# Resveratrol-Based Carbamates as Selective Butyrylcholinesterase Inhibitors: Design, Synthesis, Computational Study and Biometal Complexation Capability

**DOI:** 10.3390/molecules30020316

**Published:** 2025-01-15

**Authors:** Maja Sviben, Ilijana Odak, Danijela Barić, Milena Mlakić, Ottó Horváth, Lajos Fodor, Sunčica Roca, Ivana Šagud, Irena Škorić

**Affiliations:** 1 Department of Organic Chemistry, Faculty of Chemical Engineering and Technology, University of Zagreb, Trg Marka Marulića 19, HR-10 000 Zagreb, Croatia; mdragojev@fkit.unizg.hr (M.M.); mratajec@fkit.unizg.hr (M.S.); 2 Department of Chemistry, Faculty of Science and Education, University of Mostar, Matice Hrvatske bb, 88 000 Mostar, Bosnia and Herzegovina; ilijana.odak@fpmoz.sum.ba; 3 Group for Computational Life Sciences, Division of Physical Chemistry, Ruđer Bošković Institute, Bijenička Cesta 54, HR-10 000 Zagreb, Croatia; dbaric@irb.hr; 4 Environmental and Inorganic Photochemistry Research Group, Center for Natural Sciences, Faculty of Engineering, University of Pannonia, P.O. Box 158, H-8201 Veszprém, Hungary; horvath.otto@mk.uni-pannon.hu (O.H.); fodor.lajos@mk.uni-pannon.hu (L.F.); 5 NMR Center, Rudjer Bošković Institute, Bijenička Cesta 54, HR-10 000 Zagreb, Croatia; sroca@irb.hr; 6 Croatian Agency for Medicinal Products and Medical Devices, Ksaverska Cesta 4, HR-10 000 Zagreb, Croatia

**Keywords:** ADMET, carbamates, butyrylcholinesterase inhibition, biometal complexation, docking, molecular dynamics, synthesis

## Abstract

Considering our previous experience in the design of new cholinesterase inhibitors, especially resveratrol analogs, in this research, the basic stilbene skeleton was used as a structural unit for new carbamates designed as potentially highly selective butyrylcholinesterase (BChE) inhibitors with excellent absorption, distribution, metabolism, excretion and toxicity ADMET properties. The inhibitory activity of newly prepared carbamates **1**–**13** was tested toward the enzymes acetylcholinesterase (AChE) and BChE. In the tested group of compounds, the leading inhibitors were **1** and **7**, which achieved excellent selective inhibitory activity for BChE with IC_50_ values of 0.12 ± 0.09 μM and 0.38 ± 0.01 μM, respectively. Both were much more active than the standard inhibitor galantamine against BChE. Molecular docking of the most promising inhibitor candidates, compounds **1** and **7**, revealed that stabilizing interactions between the active site residues of BChE and the ligands involve π-stacking, alkyl-π interactions, and, when the carbamate orientation allows, H-bond formation. MD analysis confirmed the stability of the obtained complexes. Some bioactive resveratrol-based carbamates displayed complex-forming capabilities with Fe^3+^ ions as metal centers. Spectrophotometric investigation indicated that they coordinate one or two metal ions, which is in accordance with their chemical structure, offering two binding sites: an amine and a carboxylic group in the carbamate moiety. Based on the obtained in silico, experimental and computational results on biological activity in the present work, new carbamates **1** and **7** represent potential selective BChE inhibitors as new therapeutics for neurological disorders.

## 1. Introduction

Alzheimer’s disease is a progressive neurodegenerative disease characterized by the deterioration of synapses and neurons in the cortical and subcortical regions of the brain and impairment of the function of nerve impulse transmitter systems [1,2]. Alzheimer’s disease (AD) is a complex multifactorial disease featuring several pathophysiological factors linked to various hypotheses about the origin and causes of AD: the cholinergic hypothesis, the amyloid hypothesis, the tau hypothesis, and the oxidative stress hypothesis. Also, it was observed that in certain parts of the brain affected by AD, there is an increased accumulation of metals, which leads to a change in the homeostasis of biometals. Changes in the biodistribution of metals in presynaptic and postsynaptic neurons cause abnormal neurotransmission, affect the activities of metal-dependent enzymes and increase the secretion of amyloid β-peptides. Furthermore, metal ions with amyloid β-peptides form stable complexes that are more toxic than Aβ plaques and can generate reactive oxygen species that cause oxidative stress, lipid peroxidation and protein damage [3,4,5,6,7].

**Figure 1:**
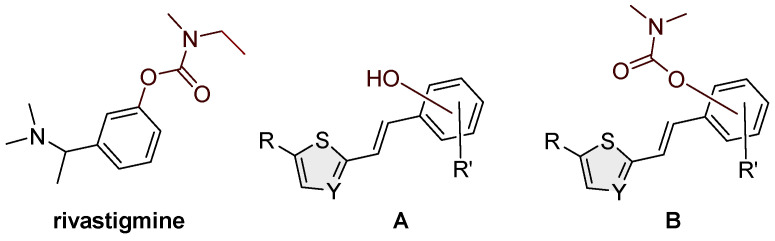
Structures of rivastigmine, a progressive non-selective inhibitor of AChE and BChE; resveratrol analogs (**A**) exhibiting significant BChE inhibitory potential [30,32] and newly designed compounds from this work (**B**).

The complex pathophysiology of AD indicates the need to develop new molecules that simultaneously act on multiple targets responsible for the onset and development of the disease. Previous investigations have shown that designed hybrid molecules such as cholinesterase (ChE) inhibitors (galantamine, donepezil and tacrine) also have additional activities depending on the fragments introduced into the structure. Today, the treatment of AD is exclusively symptomatic and mainly alleviates symptoms rather than targeting the course, development and outcome of the disease [5,8]. Treatment is based mainly on increasing the concentration of acetylcholine (ACh) by inhibiting cholinesterase; three of the five drugs currently used for the treatment of AD are ChE inhibitors: galantamine, donepezil and rivastigmine. The carbamate rivastigmine (Figure 1) is a progressive non-selective inhibitor of acetylcholinesterase (AChE) and butyrylcholinesterase (BChE) [9,10] that acts in the central nervous system and is particularly active in the cortex and hippocampus.

To date, a number of carbamates of different structures have been synthesized and tested, and they have been shown to inhibit both cholinesterases, AChE and BChE. The mechanism of action of carbamates on cholinesterase is similar to that of ACh on AChE. However, unlike the extremely fast hydrolysis of acetylcholine, the hydrolysis of carbamates is much slower [11,12,13]. The first known cholinesterase inhibitor was the first carbamate drug, physostigmine, a methylcarbamate ester, also known as eserine, which was initially used as a drug for treating AD [14]. Rivastigmine (Figure 1) is a BChE and AChE inhibitor used in the treatment of mild, moderate and severe AD, as well as mild to moderate dementia in Parkinson’s disease [15,16]. The design of new cholinesterase inhibitors is based on modifying the structure of existing carbamate cholinesterase inhibitors or synthesizing carbamate derivatives of existing AD drugs to find new, more effective drugs for the treatment of neurodegenerative diseases. To date, a number of carbamates with different structures have been synthesized and tested, which have been shown to inhibit both cholinesterases, AChE and BChE [17,18,19,20,21,22,23]. Since the selective inhibition of BChE stood out as a promising approach to treating AD, one part of the research was directed toward finding a selective BChE inhibitor with a carbamate group in its structure. So far, the carbamate cymserine, a structural analog of physostigmine with an isopropylphenyl instead of a methyl group at N-4, has proven to be an immensely selective BChE inhibitor, which is 15 times more effective as a BChE inhibitor compared to AChE. However, the use of cymserine for the treatment of AD was stopped due to the formation of the toxic metabolite eseroline.

Taking into account all our previous experience in the design of new cholinesterase inhibitors [24,25,26,27] and arylethenes [28,29], especially resveratrol analogs [30,31,32] (Figure 1, structure A), in this research, the skeletons of the previous molecules were used as a structural basis for new carbamates (Figure 1, structure B). Several of those resveratrol analogs exhibited significantly enhanced BChE inhibitory and antioxidant activity compared to established standards such as galantamine or resveratrol (Figure 1, structure A), which was an excellent starting point for this research. Based on the obtained in silico, experimental and computational results regarding biological activity in the present work, novel carbamates (Figure 1, structure B) represent potential selective BChE inhibitors as new therapeutics for neurological disorders.

## 2. Results and Discussion

### 2.1. Design, Synthesis and Characterization of New Carbamates ***1**–**13***

**Scheme 1:**
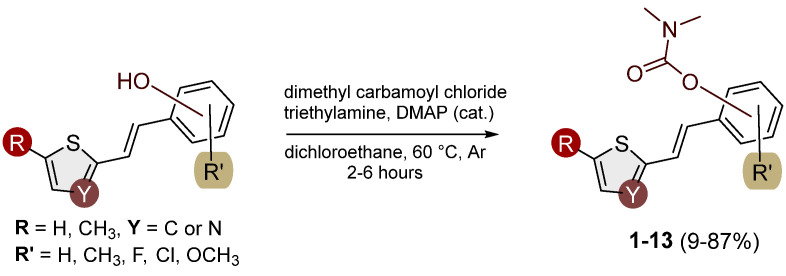
Newly designed and synthesized carbamates **1**–**13**.

Analogs of resveratrol previously synthesized by our group [30,31,32] were used as the starting material for synthesizing carbamates **1**–**13** in dichloroethane (Scheme 1). At room temperature in an inert atmosphere, 1.8 eq of triethylamine and a catalytic amount of DMAP were added. After that, the reaction mixture was stirred under argon, followed by adding 2 eq of dimethylcarbamoyl chloride. In the end, the reaction mixture was worked up (see Section 3).

**Figure 2:**
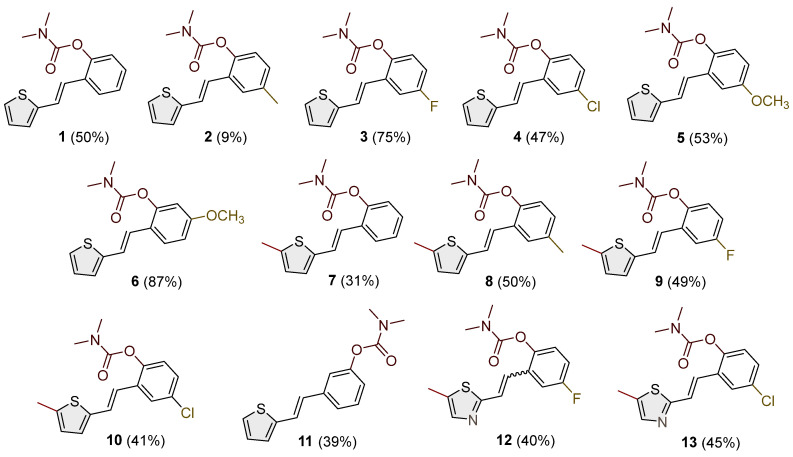
Newly synthesized carbamates **1**–**13** bearing various functionalities (isolated yields for individual compounds are shown in parentheses).

The resulting products, **1**–**13**, were purified through column chromatography, using petroleum ether and diethyl ether in differing ratios as the eluent, and isolated in a wide and different range of yields depending on the nature and position of the substituents (9–87%, Figure 2). It can be noted that the nature of the heterocyclic nucleus, the substituent on it and the position of the carbamate group did not affect the isolated yields of the carbamates, and for most of the derivatives, these yields were in the range of 40–50%. The highest yield was obtained fore derivative **6** (87%), in which the methoxy group was in the *meta*-position in relation to the carbamate group, unlike most derivatives that have substituents in the *para*-position in relation to the carbamate substituent. The lowest yields were obtained for products **2** (9%) and **7** (31%), which had one methyl group in addition to the carbamate, either on the heterocyclic or the aryl ring.

The structures of the newly synthesized *trans*-resveratrol-based carbamates **1**–**13** were confirmed via NMR (^1^H and ^13^C) and HRMS analyses (Figure 3 and Figures S1–S63). The purity of the newly synthesized carbamates was determined using the HPLC method and ranged from 85 to 99%, depending on the compound.

**Scheme 2:**
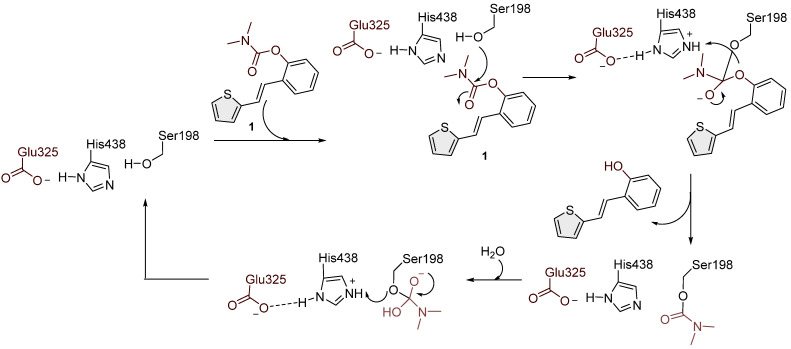
Possible mechanism of action of carbamate **1** in the BChE enzyme active site by analogy with acetylcholine and rivastigmine.

As in this research, *trans*-resveratrol-based carbamates **1**–**13** were designed as selective BChE inhibitors, it is worth briefly referring to BChE-assisted substrate (carbamate) hydrolysis (Scheme 2), which is analogous to the mechanism of the AChE-assisted hydrolysis of ACh. The mechanism includes the formation of the Michaelis complex, acylation of the enzyme, and its spontaneous deacylation with water (Scheme 2). In the first step, under the influence of Glu325, His438 acts as a base that attracts a proton from the hydroxyl group of Ser198, thus making it more nucleophilic than the other serine residues present in the enzyme. The nucleophilic oxygen of Ser198 reacts with the carbonyl carbon atom of the carbamate, resulting in a tetrahedral intermediate. After that, the serine remains acetylated. One water molecule, which, under the influence of His438, becomes more nucleophilic than other water molecules in the active site of the enzyme, reacts with the carbonyl carbon of the acylated enzyme, which leads to the formation of a new tetrahedral intermediate and ultimately to the deacylation of the enzyme [33,34].

### 2.2. Cholinesterases Inhibitory Activity of Carbamate Derivatives

Previously tested resveratrol analogs [32] with a thiophene or thiazole ring on one side and a phenol ring on the other side of the stilbene moiety were converted into carbamates (Scheme 1, Figure 2). The inhibitory activity of newly prepared carbamates **1**–**13** was tested toward acetylcholinesterase (AChE) and butyrylcholinesterase (BChE). The assays were carried out using Ellman’s method [35] with minor modifications, and the results were expressed as IC_50_ values and presented in Table 1.

The most dramatic result of translation from resveratrol analogs into carbamate derivatives is the complete loss of activity towards acetylcholinesterase; namely, none of the tested derivatives achieved more than 35% inhibition of this enzyme at the maximum tested concentrations. This is not surprising considering the presence of the carbamate group, which has been shown in various derivatives to be highly selective for BChE [36,37,38,39]. Furthermore, butyrylcholinesterase inhibition is highly successful and has better concentration values than resveratrol analogs. In the tested group of compounds, the leading inhibitors were **1** and **7**, which achieved excellent inhibitory activity for BChE, with IC_50_ values of 0.12 ± 0.09 μM and 0.38 ± 0.01 μM, respectively. Both were much more active than galantamine against BChE (Table 2). The common structural feature of these two inhibitors is that they have no substituent on the carbamate side of stilbene, and they differ only in the presence/absence of methyl on thiophene. Changing the position of the carbamate group from *ortho* (derivative **1**) to *meta* (derivative **12**) drastically reduces the activity towards the BChE by two orders of magnitude (IC_50_ for **12** is 11.37 ± 2.48 μM).

In the group of compounds without the methyl at the thiophene ring (**1**–**6**), almost all derivatives achieved excellent inhibition values, better than the standard galantamine. A slight decline in activity was found for derivative **5**, the one with methoxy substituent. The effects of the substituents on the benzene ring were similar to resveratrol derivatives [32]. Methylation of the thiophene ring in derivatives **8**–**10** reduced the inhibitory effect, but the obtained IC_50_ values were still in the moderate range. Two thiazole derivatives, **12** and **13,** were also tested, and **13** stood out with a very good inhibition value of 3.77 ± 0.13 μM, better than the galantamine. Finding a selective inhibitor of BChE continues to be one of the approaches to discovering new therapeutic treatments for AD. It is shown that the carbamates synthesized and tested here show powerful and utterly selective inhibition of BChE, which makes this structural basis interesting for the development of potent BChE inhibitors.

### 2.3. In Silico Evaluation of ADME(T) Properties and In Silico Assessment of the Possibility of a Compound Passing Through the Blood–Brain Barrier via Passive Transport

Absorption, distribution, metabolism, excretion and toxicity (ADME(T)) studies are essential in any drug development stage. They give insights into whether the compound exhibits drug-like pharmacokinetic properties and whether it has properties that will cause safety concerns in people [40]. ADMET properties are investigated in silico, in vitro, and in vivo. In early drug discovery, in silico tools are used when molecules are either not yet synthesized in a pure enough form or the quantities synthesized are insufficient, as the synthetic pathways are still developed and optimized in the early stages. This study used free in silico tools [41,42] to screen the candidate compounds.

The data presented in Table 2 show that the water solubility is very low. As these carbamate derivatives tested positive for selective BChE inhibition, they should be able to cross the BBB barrier to work as potential CNS drugs for Alzheimer*’*s disease. The in silico data that will indicate that the CNS potential are the ones under distribution in the above table.

The BBB (as log BB = the drug concentration in the brain divided by the concentration in the blood) for both compounds is approx. 0.3. This result is promising, as compounds with log BB > 0.3 readily cross the BBB. The second indicator is the Log PS (permeability surface area); here, log PS > −2 value signifies molecules that can penetrate the CNS. For compounds **1** and **7,** this value is above −0.94, indicating that they are very good candidates. As the CNS is very dependent on the general indicators of distribution in the body, the VDss and the fraction unbound (human) (Fu) were also calculated. VDss is considered the least-biased indicator of the extent of distribution. This pharmacokinetic parameter represents the hypothetical volume into which the drug dose would have to be evenly distributed to give rise to the same concentration observed in the blood plasma. When these two parameters are calculated and commented on together, they show how the decreased plasma protein binding leads to an increase in free plasma fraction, causing an increase in the volume of distribution and a shorter elimination half-life. They link distribution and elimination. For a CNS drug, the unbound fraction has a twofold effect, and the interpretation of this indicator based on in silico preliminary results is complex. Drugs highlighted as potential CNS candidates are very sensitive to the unbound portion of the drug, as a maximal concentration in the plasma is one of the key factors with the CNS candidates. Usually, the bonds to the blood proteins are reversible, but this would require further experimental results for the tested structures. If we consider that the unbound drug is the effective form, then the higher Fu can indicate better bioavailability, and the drug can better distribute in the central nervous system.

**Figure 4:**
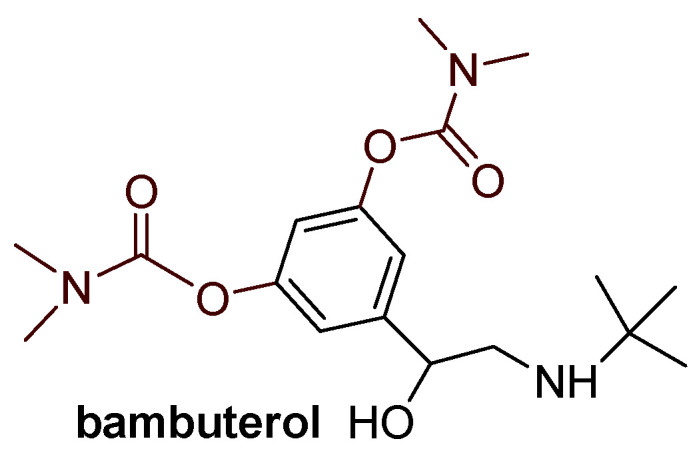
Structure of bambuterol.

Since these candidates can be regarded as analogs containing an active carbamate moiety, it was practical to compare the indicators mentioned above with well-known drugs with the same structural fragment. Bambuterol (Figure 4) was selected due to its structural resemblance to rivastigmine and its similar therapeutic indication (see Section 1).

As these are well-known drugs, the in silico prediction was made only for the distribution parameters VDss, BBB and PS. The parameter VDss is similar to the one for compounds **1** and **7**. Log BB is greater for the known drugs, but as drugs above 0.3 signify that they readily cross the BBB barrier, the lead compounds **1** and **7** still meet the criteria.

From all of the parameters tested in the range of in silico ADME(T) studies and the study of potential genotoxicity, it is safe to promote compounds **1** and **7** as promising leads that could be further developed into drug products.

### 2.4. Docking Study and Molecular Dynamics

**Figure 5:**
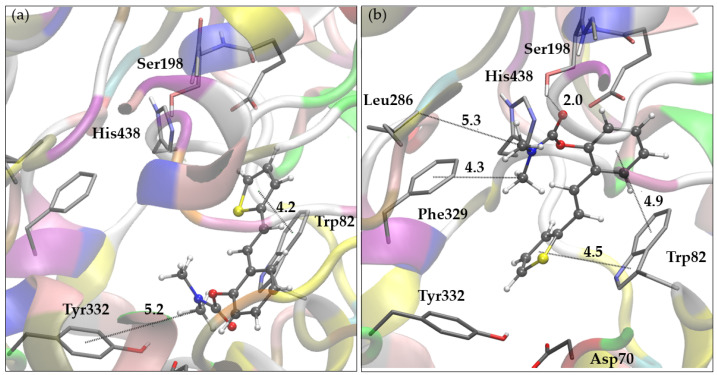
(**a**) Representative pose of the most populated cluster of molecule **1** docked into the active site of BChE. (**b**) The most stable pose of molecule **1** obtained by docking to BChE. Distances given in angstroms. Docked molecules are presented using ball-and-stick models.

**Figure 6:**
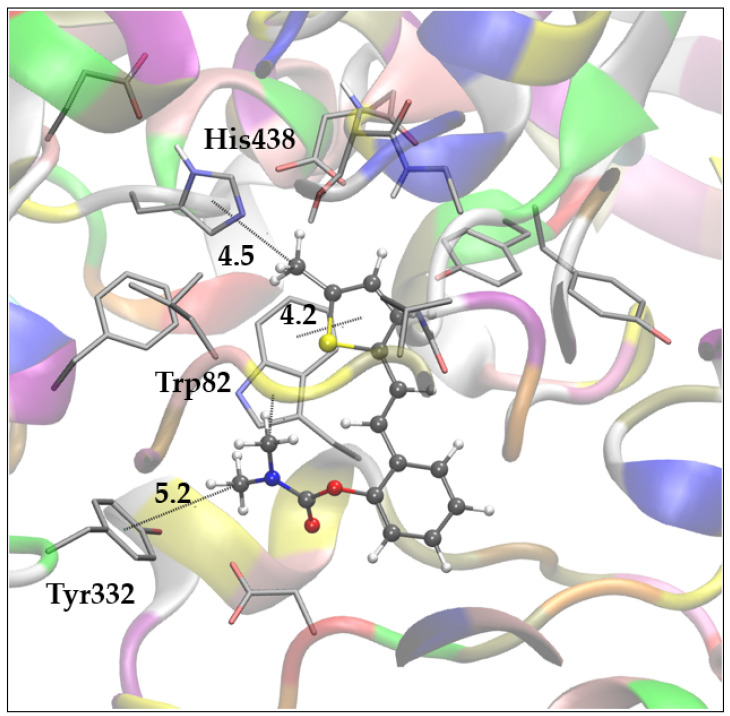
Representative pose of the most populated and most stable cluster of molecule **7** docked into the active site of BChE. Distances in angstroms. Docked molecules are presented using ball-and-stick models.

Compounds **1** and **7**, identified as the most potent inhibitors, were docked into the active site of BChE. Figure 5 and Figure 6 illustrate the molecular structures of the resulting ligand–protein complexes, highlighting key interactions that contribute to their stability.

According to the docking results for compound **1**, the representative pose of the most populated cluster, depicted in Figure 5a, shows the carbamate group oriented toward the peripheral anionic site (PAS). The methyl groups of the dimethylamino carbamate fragment engage in alkyl-π interactions with Tyr332 (PAS) and Trp82, belonging to the anionic site, while the thiophene ring interacts with Trp82. The second complex of compound **1** with BChE, illustrated in Figure 5b, belongs to a slightly more stable but significantly less-populated cluster of poses compared to the cluster represented by the pose shown in Figure 5a. In this conformation, the carbamate group is oriented toward the esteratic site, where the carbonyl oxygen forms a hydrogen bond with the hydroxyl group of Ser198. The dimethylamino fragment of the ligand is directed toward Leu286 in the acyl pocket but also engages in hydrophobic interactions with Phe329. The ligand*’*s aromatic rings (thiophene and phenyl) contribute to the stabilization of the complex through π-stacking interactions with Trp82.

The complex of compound **7** with BChE, shown in Figure 6, is representative of the most stable and most populated cluster of poses obtained via docking. Here, the ligand adopts a conformation similar to that observed for compound **1** in Figure 5a. The carbamate group is oriented toward PAS, and the methyl groups of the dimethylamino fragment participate in alkyl-π interactions with Tyr332 and Trp82. Furthermore, one additional alkyl-π interaction is noted between the -CH₃ substituent on the thiophene ring and His438 in the esteratic site, while the thiophene ring maintains its interaction with Trp82 at the anionic site.

The binding free energies estimated from docking for compounds **1** and **7** are −6.82 (−6.85) kcal mol^−1^ and −7.43 kcal mol^−1^, respectively (Table S1). For comparison, the binding free energy for the reference ligand galantamine, calculated using the same method, was −7.54 kcal mol^−1^.

**Figure 7:**
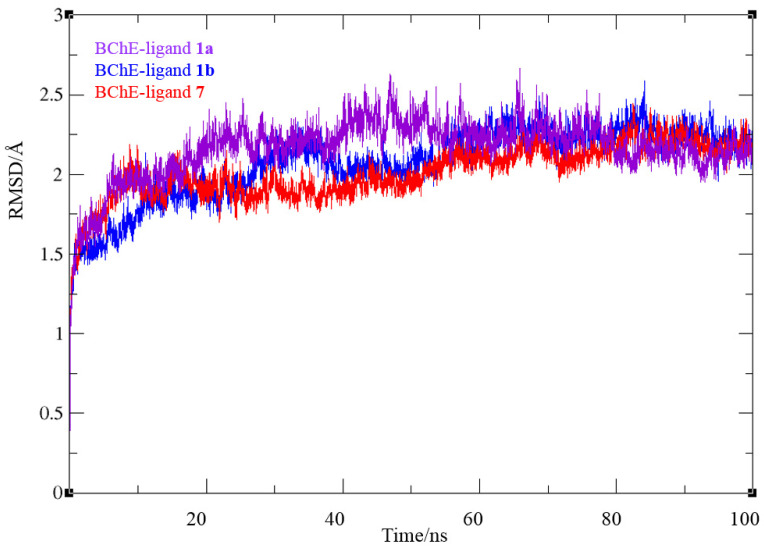
Root mean square deviation values from molecular dynamics simulation of a protein–ligand complex of BChE with compounds **1** and **7**, respectively. Complexes BChE-**1a** and BChE-**1b** correspond to the ligand conformations presented in Figure 5a and Figure 5b, respectively.

**Figure 8:**
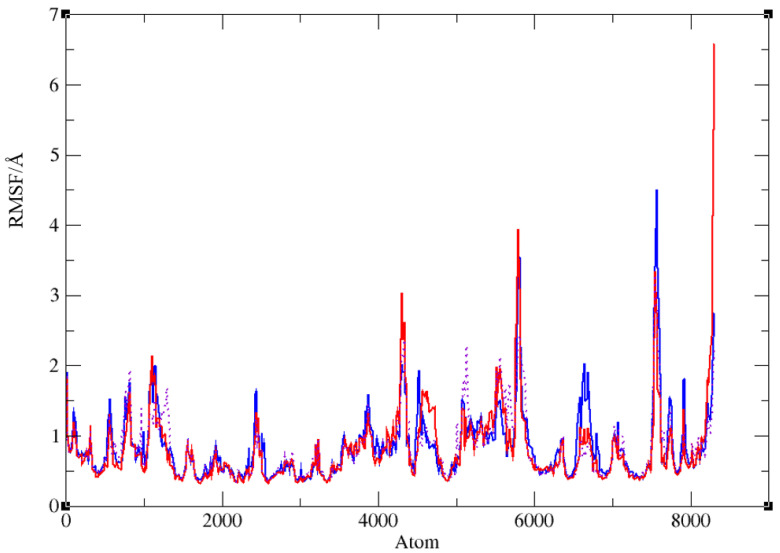
Root mean square fluctuation values from molecular dynamics simulation of a protein–ligand complex of BChE with compounds **1** (dotted violet and blue lines) and **7** (red line), respectively.

**Figure 9:**
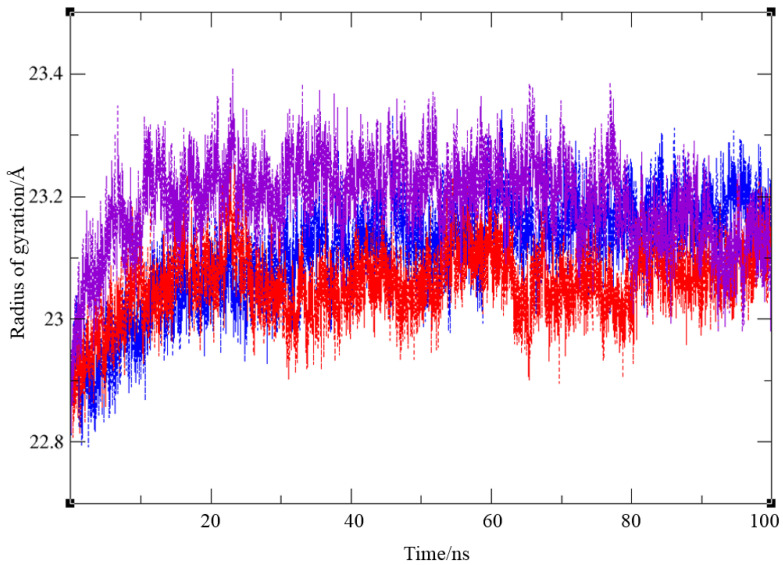
Values of a radius of gyration from molecular dynamics simulation of a protein–ligand complex of BChE with compounds **1** (violet for conformation **1a**, and blue for **1b**) and **7** (red line), respectively.

The stability of ligand–enzyme complexes obtained through docking was evaluated through molecular dynamics (MD) simulations. The production simulation lasted 100 ns, and root mean square deviation (RMSD) values were calculated to monitor structural changes in the protein–ligand complex over the simulation period. RMSD analysis was conducted on all atoms, excluding hydrogens, for all three complexes, BChE-**1a**, BChE-**1b** and BChE-**7**. Additionally, root mean square fluctuation (RMSF) values were computed as average quadratic fluctuations in the positions of α carbons over the trajectory, identifying the most mobile regions of the protein backbone. To estimate protein compactness in the complexes with ligands, the radius of gyration (Rg) was calculated as the RMS average of the distance of all atoms from the protein–ligand complex*’*s center of mass. A significant increase in Rg suggests a more expanded or flexible protein conformation in the presence of the ligand. The MD simulation results for BChE complexes with compounds **1** and **7** are shown in Figure 7, Figure 8 and Figure 9.

In all three systems, convergence was achieved after 30 ns. For the complexes with ligand **1**, the RMSD values ranged from 0.78 to 2.67 for BChE-**1a** and to 2.59 Å (BChE-**1b**), averaging 2.16 (2.07) Å, respectively, with similar values for ligand **7** (0.81 to 2.44 Å, averaging 2.02 Å). The average RMSD for the final 70 ns decreased to 1.69 (1.89) and 1.90 Å for ligands **1** and **7**, respectively. The RMSF analysis (Figure 8) revealed that in the BChE**-1a** (violet dotted line), the biggest value was 3.29 Å, corresponding to residue Gln484, while for the BChE-**1b** complex (blue line), the most mobile protein backbone regions were α carbons 7559 and 5786, corresponding to residues Asn485 and Trp376, with RMSF values of 4.50 and 3.61 Å, respectively. In the BChE–ligand **7** complex (red line), the highest RMSF (6.59 Å) was observed for Val529 (positioned at a distance of 24 Å from the active site, thus not influencing its structure), with the other residues showing significantly less mobility throughout the simulation.

Finally, the radius of gyration for three complexes, shown in Figure 9, indicates that protein compactness was maintained during the simulation, with Rg values within a narrow range of 22.81–23.41 Å for BChE–ligand **1a**, 22.79–23.34 Å for the complex with ligand **1b**, and 22.81–23.28 Å for BChE–ligand **7**, respectively.

**Figure 10:**
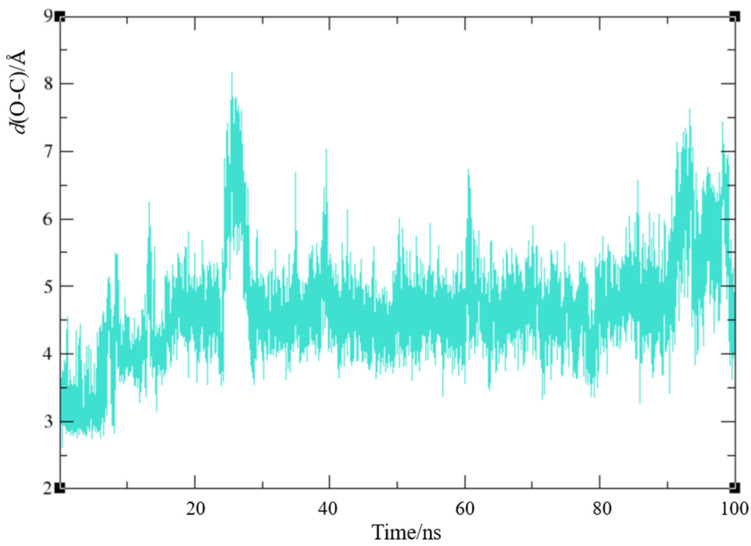
Distance between hydroxyl oxygen of the catalytic serine and carbonyl carbon of ligand **1** based on molecular dynamics simulation of a protein–ligand complex BChE-**1b**.

Although the orientation of ligand **1** depicted in Figure 5b is not the most prevalent among the poses generated through docking, it is noteworthy, as it may enhance the covalent binding of the carbamate to the catalytic serine. In this docking conformation, the initial distance between the hydroxyl oxygen of Ser198 and the carbonyl carbon of the ligand, *d*(O-C), is favorable at 3.13 Å. Therefore, we examined the trajectory of the MD simulation of complex BChE-**1b** to assess the stability of this distance over time. Figure 10 illustrates the variation in distance between the serine oxygen and the carbonyl carbon for 100 ns.

The optimal distance for the reaction to start should not exceed 4 Å [43,44]. Here, the O-C distance increases significantly after the first 25–30 ns, with initial values below 4 Å not being maintained. A favorable value of ~4 Å or less is present 14% of the time; even 60% of the time, this distance is greater than 4.5 Å. This variability may be attributed to the size of the ligand, which allows for more space within the relatively large active site. The fluctuations in the d(O-C) suggest that, despite the initial conformation appearing highly conducive for the nucleophilic attack of the serine oxygen on the ligand*’*s carbonyl carbon, the chemical reaction may not occur. However, further experimental measurements are needed to reach a definitive conclusion.

### 2.5. Complex Formation of Biometals with Bioactive Carbamates

Our previous studies on other cholinesterase inhibitory active resveratrol derivatives [45] made it reasonable to investigate the interactions of some of these resveratrol-based carbamates with Fe^3+^, a potential biometal. For this purpose, the following compounds were selected: **1**, **3**, **6** and **7**. The Fe^3+^ ions strongly hydrolyze, decreasing the pH of the aqueous system; thus, acidic solutions were used to study the possible complex formation via spectrophotometric titrations. Although generally, in these experiments, the concentration of the potential ligand (L) is increased at a constant concentration of the metal center (Fe^3+^ in this case), in our work, another type of titration was also carried out, i.e., increasing the concentration of the metal center at a constant C_L_ value. Nevertheless, the previous method provided more valuable information in our case.

**Figure 11:**
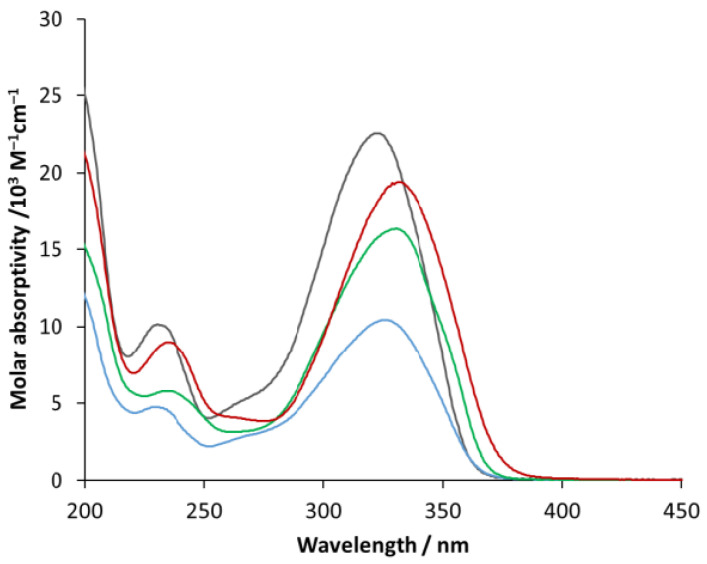
Molar absorptivity spectra of compound **1** (black), **3** (blue), **6** (green) and **7** (red).

First, the absorption spectra of the selected ligands were recorded for spectrophotometric titration. Figure 11 shows the corresponding molar absorptivity spectra, which are very similar to each other from the viewpoint of the band positions. The shorter-wavelength bands (at about 233–238 nm) may be designated as thiophene-centered ones due to a π–π* transition. The longer-wavelength bands (at about 322–331 nm) belong to the whole 2-styryl thiophene moiety due to the conjugation of the two aromatic rings through the vinyl bridge. The substituents hardly influence the band positions; instead, they affect the molar absorptivities.

The molar absorptivities at the longer-wavelength band maxima are given in Table 3.

**Figure 12:**
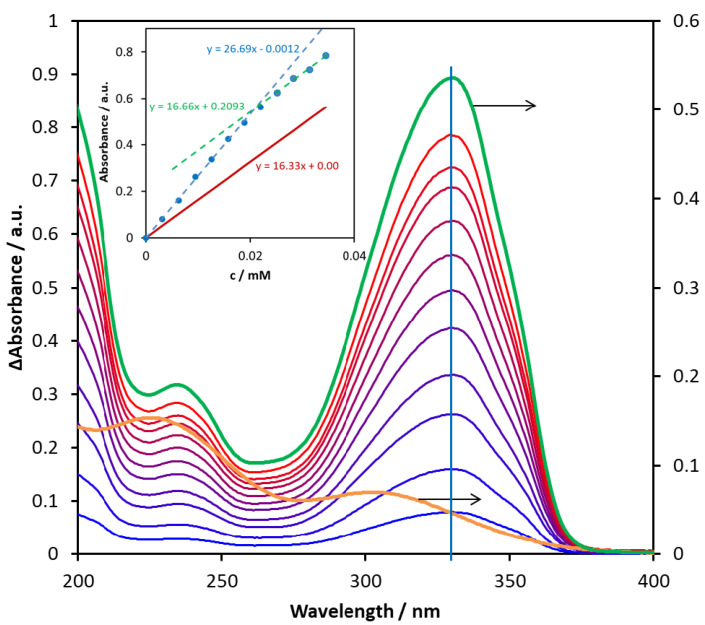
Difference absorption spectra obtained through titration of 1.93 × 10^–5^ M of Fe_2_(SO_4_)_3_ aqueous solution with compound **6** in the 0–0.35 mM concentration range. The orange curve is the spectrum of the starting pure Fe_2_(SO_4_)_3_ solution, while the green spectrum belongs to the pure ligand. The inset shows the changes in absorbance measured at 330 nm (blue) and the theoretical absorbance calculated for the ligand at the same wavelength (red) as a function of the concentration of **6**.

Figure 12 demonstrates a titration series of spectra, in the case of which the concentration of ligand (**6**) was increased up to 3.5 × 10^−5^ M. During the titration, the band position did not change, but the absorbance at the band maximum appreciably deviated from the straight line (see inset). This phenomenon unambiguously suggests an interaction between the metal ion and the potential ligand. However, the absorption spectrum of the formed associate or complex is very similar to that of the ligand; only the corresponding absorptivities are lower. Since the deviation from the straight line is relatively small, the estimation of the formation constant is very uncertain. Nevertheless, molar absorptivity and the composition of the complex may be suggested based on the absorbance vs. ligand concentration plot (Figure 11, inset). As shown in the inset, which can be seen in a much larger version in Figure S64, the absorbance (at 330 nm) vs. ligand concentration plot indicates an appreciable breaking point, before which the slope is higher than that of the red line representing the theoretical absorbance of the ligand (calculated by using its molar absorptivity at 330 nm). The slope values are the molar absorptivities of the corresponding species at 330 nm. Hence, the molar absorptivity of the complex formed in this system is about 26,700 M^−1^ cm^−1^, which is ca. 64% higher than that of the ligand, indicating a considerable interaction between the metal ion and the ligand. The composition of the complex can be estimated using the position of the breaking point. The concentration of the ligand is about half of Fe^3+^ at the breaking point (0.02 mM vs. 0.038 mM), which suggests a 2:1 (M_2_L) composition. This result indicates that metal ions may be bound to both coordination sites of this carbamate ligand, i.e., to both the carboxylic and the amine moiety. Nevertheless, the carboxylic group is preferred in this respect. Since the slope of the absorbance vs. ligand concentration plot after the breaking point agrees with that of the absorptivity of the ligand, i.e., the slope of the red straight line, no further complex formation occurred in this range.

A similar phenomenon was observed for compound (**3**) (Figure S65). In this case, the absorptivity of the complex (13,200 M^−1^ cm^−1^) just moderately (by about 26%) exceeded that of the ligand (10,400 M^−1^cm^−1^). The position of the breaking point, i.e., the ligand-to-metal concentration ratio, suggested a 1:1 composition. This finding agrees with the lower absorptivity of the complex, indicating a weaker interaction. The absorbance vs. ligand concentration plot shows no deviation from the straight line for the compounds with no substituent on the benzene ring (apart from the common carbamate group), i.e., for **1** and **7**. Since the absorption spectra of the complexes, when they formed, were very similar to those of the ligands, the spectral series of titration with the metal center did not prove to be as informative as those obtained by increasing the ligand concentration.

### 2.6. In Silico Genotoxicity Evaluation

Determination of impurities is a vital part of active substance and drug development. Within the scope of impurities of drug substances and drug products, possible mutagenic/cancerogenic impurities take a special role, as they are controlled with much lower limits (ICH M7 Guideline). It is common when developing new drug substances and products that the impurities will also be new compounds, and there will be no experimental data available for them in the literature databases. In these cases, the Q(SAR) approach is vital. (Q)SAR models make predictions based on structural components [46]. This approach for evaluation of the mutagenic potential of compounds can also be used to determine the mutagenic potential during the early stages of searching for potentially active drug substances. This is extremely important, as compounds with biological activity and mutagenic potential can be eliminated early on. The Lhasa software (https://www.lhasalimited.org/) is the most commonly used tool because two complementary models (one rule based and one statistics based) are used, and their predictions are reviewed one more time by an expert.

In the case of compounds **1**–**13** (Table 4) that were investigated for their potential biological activity with an emphasis on the selective inhibition of enzyme BChE, only one structure was identified by the software to have a potential for mutagenicity. This was compound **11**. Here, the Sarah prediction gives a 9% chance of some of the structural features in this precise structural environment being positive. It is important to note that the most active molecules, **1** and **7**, were reviewed as negative and can be promising future leads for further early drug product development stages.

## 3. Materials and Methods

### 3.1. General Procedure

NMR spectra were recorded using a Bruker AV300 or AV600 spectrometer (Bruker BioSpin GmbH, Rheinstetten, Germany) with a 5 mm probe head at the Ruđer Bošković Institute. Standard 1H and proton-decoupled ^13^C{^1^H} NMR spectra were recorded at 300.000 and 600.130 MHz and 75.432 and 150.903 MHz, respectively. The chemical shifts (*δ*/ppm) of the ^1^H and ^13^C spectra were related to the tetramethylsilane signal (TMS). All spectra were recorded in deuterated chloroform (CDCl_3_-d) at 25 °C. The signal at 2.36 ppm in the proton spectra belongs to toluene.

The reactions were monitored using thin-layer chromatography performed on silica gel-coated plates (0.2 mm, 60/Kieselguhr F_254_) immersed in 10 mL of the dissolution system. Workup after each synthesis included an extraction between water and DCE, with a single addition of 2.4 M HCl. After separating the organic and aqueous layers, the organic layer was dried over anhydrous magnesium sulfate, MgSO_4_. The obtained compounds were purified via column chromatography performed in glass columns of different diameters, filled with silica gel (60 Å, technical grade) of different heights. The abbreviations used in the experimental part are as follows: TEA—triethylamine, DMAP—4-dimethylaminopyridine, DCE—1,2-dichloroethane, PE—petroleum ether, E—diethyl ether, HCl—hydrochloric acid, NMR—nuclear magnetic resonance, s—singlet, d—doublet, m—multiplet, t—triplet, dd—doublet of doublets. High-resolution mass spectrometry (HRMS) analyses were performed using a MALDI TOF/TOF analyzer mass spectrometer fitted with an Nd:YAG laser at 355 nm (fitting rate of 200 Hz).

### 3.2. Synthesis of Carbamates ***1**–**13***

Analogues of resveratrol previously synthesized by our group [30,31,32] were used as the starting material in the synthesis of carbamates **1**–**13**. In a 25 mL round-bottomed flask, depending on the starting resveratrol, 50–100 mg was dissolved in 1 mL of DCE. While stirring at room temperature in an inert atmosphere, 1.8 eq of TEA and a catalytic amount of DMAP were added. The reaction flask was then submerged in an oil bath, and the mixture was stirred at 60 °C for 15 min under argon. Afterwards, 2 eq of dimethylcarbamoyl chloride was added dropwise, and stirring continued at 60 °C in an inert atmosphere for 2 h. At the end of the reaction, the reaction mixture was extracted three times between DCE and distilled water, with a single addition of 1 mL of 2.4 M HCl. The organic layer was dried over anhydrous magnesium sulfate, MgSO_4_, filtered, and evaporated to dryness using a rotary evaporator. The resulting products, **1**–**13**, were purified through column chromatography, using petroleum ether and diethyl ether in differing ratios as eluents. NMR analyses indicated carbamates **1**–**13** were obtained as *trans*-isomers (with traces of *cis*-isomer in some samples), with the exception of **12**, which was obtained with a prevalence of the *cis-*isomer (*trans*:*cis* = 0.3:1).


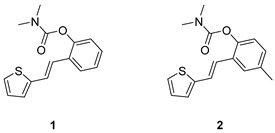


(*E*)-2-(2-(thiophen-2-yl)vinyl)phenyl dimethylcarbamate (**1**): 67 mg (isolated 50%), brown oil; *Rf* (40% PE/E) = 0.50; ^1^H NMR (CDCl_3_, 300 MHz) *δ*/ppm: 7.61 (dd, *J* = 7.6, 1.3 Hz, 1H), 7.25–7.11 (m, 5H), 7.08–6.97 (m, 3H), 3.20 (s, 3H), 3.04 (s, 3H); ^13^C NMR (CDCl_3_, 75 MHz) *δ*/ppm: 154.6, 148.8, 143.1, 129.7, 128.3, 127.6, 126.4, 125.9, 125.6, 124.5, 123.4, 123.1, 121.9, 36.8, 36.4; HRMS (ESI) (*m*/*z*) for C_15_H_15_NO_2_S: [M + H]^+^_calcd_ = 273.0823, and [M + H]^+^_measured_ = 273.0824.

(*E*)-4-methyl-2-(2-(thiophen-2-yl)vinyl)phenyl dimethylcarbamate (**2**): 12 mg (isolated 9%), yellow oil; *Rf* (40% PE/E) = 0.59; ^1^H NMR (CDCl_3_, 300 MHz) *δ*/ppm: 7.33 (br s, 1H), 7.13 (d, *J* = 8.6 Hz, 1H), 7.11–7.08 (m, 1H), 7.01–6.89 (m, 5H), 3.12 (s, 3H), 2.96 (s, 3H), 2.28 (s, 3H); ^13^C NMR (CDCl_3_, 75 MHz) *δ*/ppm: 153.8, 145.7, 142.2, 134.0, 128.1, 126.6, 125.3, 125.2, 123.4, 122.1, 121.8, 121.1, 35.8, 35.4, 19.9; HRMS (ESI) (*m*/*z*) for C_16_H_17_NO_2_S: [M + H]^+^_calcd_ = 287.0980, and [M + H]^+^_measured_ = 287.0982.


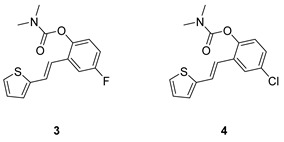


(*E*)-4-fluoro-2-(2-(thiophen-2-yl)vinyl)phenyl dimethylcarbamate (**3**): 99 mg (isolated 75%), yellow oil; *Rf* (40% PE/E) = 0.59; ^1^H NMR (CDCl_3_, 300 MHz) *δ*/ppm: 7.42–7.27 (m, 1H), 7.23–6.91 (m, 6H), 3.19 (s, 3H), 3.04 (s, 3H); ^13^C NMR (CDCl_3_, 75 MHz) *δ*/ppm: 160.1 (d, *J_CF_* = 244.2 Hz), 154.5, 146.7, 144.7 (d, *J_CF_* = 3.2 Hz), 142.5, 127.7, 127.0, 126.2 (d, *J_CF_* = 7.7 Hz), 125.0, 124.5 (d, *J_CF_* = 9.9 Hz), 122.1, 114.9 (d, *J_CF_* = 24.1 Hz), 111.8 (d, *J_CF_* = 24.1 Hz), 36.9, 36.5; HRMS (ESI) (*m*/*z*) for C_15_H_14_FNO_2_S: [M + H]^+^_calcd_ = 291.0729, and [M + H]^+^_measured_ = 291.0735.

(*E*)-4-chloro-2-(2-(thiophen-2-yl)vinyl)phenyl dimethylcarbamate (**4**): 40 mg (isolated 47%), brown oil; *Rf* (40% PE/E) = 0.59; ^1^H NMR (CDCl_3_, 300 MHz) *δ*/ppm: 7.58 (d, *J* = 2.2 Hz, 1H), 7.24–7.16 (m, 3H), 7.11–7.06 (m, 2H), 7.03–6.99 (m, 1H), 6.93 (d, *J* = 16.1 Hz, 1H), 3.19 (s, 3H), 3.04 (s, 3H); ^13^C NMR (CDCl_3_, 75 MHz) *δ*/ppm: 154.3, 147.2, 142.5, 131.3, 131.0, 128.0, 127.8, 127.1, 124.6, 124.5, 36.9, 36.5; HRMS (ESI) (*m*/*z*) for C_15_H_14_ClNO_2_S: [M + H]^+^_calcd_ = 307.0434, and [M + H]^+^_measured_ = 307.0439.


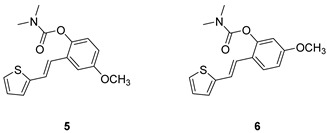


(*E*)-4-methoxy-2-(2-(thiophen-2-yl)vinyl)phenyl dimethylcarbamate (**5**): 69 mg (isolated 53%), brown oil; *Rf* (40% PE/E) = 0.34; ^1^H NMR (CDCl_3_, 300 MHz) *δ*/ppm: 7.23–7.13 (m, 2H), 7.09 (d, *J* = 2.8 Hz, 1H), 7.06–7.03 (m, 2H), 7.02–6.94 (m, 2H), 6.81 (dd, *J* = 8.7, 2.8 Hz, 1H), 3.83 (s, 3H), 3.19 (s, 3H), 3.03 (s, 3H); ^13^C NMR (CDCl_3_, 75 MHz) *δ*/ppm: 157.0, 155.0, 142.8, 132.7, 130.4, 127.6, 126.5, 124.6, 123.9, 123.6, 121.9, 114.1, 110.3, 55.7, 36.9, 36.5; HRMS (ESI) (*m*/*z*) for C_16_H_17_ClNO_3_S: [M + H]^+^_calcd_ = 303.0929, and [M + H]^+^_measured_ = 303.0936.

(*E*)-5-methoxy-2-(2-(thiophen-2-yl)vinyl)phenyl dimethylcarbamate (**6**): 57 mg (isolated 87%), colourless oil; *Rf* (40% PE/E) = 0.24; ^1^H NMR (CDCl_3_, 300 MHz) *δ*/ppm: 7.52 (d, *J =* 8.6 Hz, 1H), 7.16 (d, *J =* 4.8 Hz, 1H), 7.09 (d, *J =* 16.3 Hz, 1H), 7.03–6.97 (m, 3H), 6.78 (dd, *J =* 9.2, 2.1 Hz, 1H), 6.71 (d, *J =* 2.4 Hz, 1H), 3.81 (s, 3H), 3.20 (s, 3H), 3.05 (s, 3H); ^13^C NMR (CDCl_3_, 75 MHz) *δ*/ppm: 159.8, 154.4, 149.7, 143.4, 127.6, 126.6, 125.6, 123.9, 122.3, 121.9, 121.4, 112.5, 108.3, 55.5, 36.9, 36.5; HRMS (ESI) (*m*/*z*) for C_16_H_17_ClNO_3_S: [M + H]^+^_calcd_ = 303.0929, and [M + H]^+^_measured_ = 303.0933.


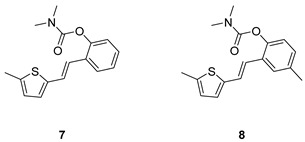


(*E*)-2-(2-(5-methylthiophen-2-yl)vinyl)phenyl dimethylcarbamate (**7**): 26 mg (isolated 31%), yellow oil; *Rf* (40% PE/E) = 0.43; ^1^H NMR (CDCl_3_, 300 MHz) *δ*/ppm: 7.58 (dd, *J* = 7.4, 1.1 Hz, 1H), 7.24–7.08 (m, 4H), 6.93–6.81 (m, 2H), 6.67–6.62 (m, 1H), 3.20 (s, 3H), 3.05 (s, 3H), 2.48 (s, 3H); ^13^C NMR (CDCl_3_, 75 MHz) *δ*/ppm: 154.6, 148.7, 141.0, 139.5, 129.9, 128.0, 126.7, 125.8, 125.8, 125.6, 123.8, 123.1, 120.6, 36.9, 36.5, 15.6; HRMS (ESI) (*m*/*z*) for C_16_H_18_NO_2_S: [M + H]^+^_calcd_ = 287.0980, and [M + H]^+^_measured_ = 287.0982.

(*E*)-4-methyl-2-(2-(5-methylthiophen-2-yl)vinyl)phenyl dimethylcarbamate (**8**): 37mg (isolated 50%), yellow oil; *Rf* (40% PE/E) = 0.50; ^1^H NMR (CDCl_3_, 300 MHz) *δ*/ppm: 7.38 (s, 1H), 7.10 (d, *J =* 16.1 Hz, 1H), 7.06–6.96 (m, 2H), 6.89–6.80 (m, 2H), 6.66–6.61 (m, 1H), 3.19 (s, 3H), 3.03 (s, 3H), 2.48 (s, 3H), 2.34 (s, 3H); ^13^C NMR (CDCl_3_, 75 MHz) *δ*/ppm: 154.8, 146.6, 141.1, 139.4, 135.0, 129.4, 128.8, 126.6, 126.2, 125.8, 123.5, 122.8, 120.8, 36.9, 36.5, 20.9, 15.6; HRMS (ESI) (*m*/*z*) for C_17_H_19_NO_2_S: [M + H]^+^_calcd_ = 301.1136, and [M + H]^+^_measured_ = 301.1142.


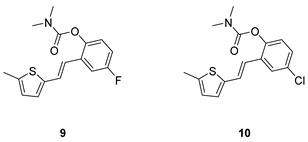


(*E*)-4-fluoro-2-(2-(5-methylthiophen-2-yl)vinyl)phenyl dimethylcarbamate (**9**): 32 mg (isolated 49%), yellow oil; *Rf* (40% PE/E) = 0.42; ^1^H NMR (CDCl_3_, 300 MHz) *δ*/ppm: 7.22–7.15 (m, 1H), 7.06–6.97 (m, 2H), 6.88–6.83 (m, 1H), 6.80 (d, *J* = 3.6 Hz, 1H), 6.73 (d, *J* = 16.0 Hz, 1H), 6.60–6.56 (m, 1H), 3.12 (s, 3H), 2.97 (s, 3H), 2.41 (s, 3H); ^13^C NMR (CDCl_3_, 75 MHz) *δ*/ppm: 160.29 (d, *J_CF_* = 243.1 Hz), 157.7, 144.7, 140.5 (d, *J_CF_* = 25.4 Hz), 131.7 (d, *J_CF_* = 8.7 Hz), 129.2, 127.6, 126.1, 125.0, 124.6 (d, *J_CF_* = 8.9 Hz), 119.6, 114.7 (d, *J_CF_* = 23.8 Hz), 111.8 (d, *J_CF_* = 24.5 Hz), 37.1, 36.6, 15.8; HRMS (ESI) (*m*/*z*) for C_16_H_16_FNO_2_S: [M + H]^+^_calcd_ = 305.0886, and [M + H]^+^_measured_ = 305.0890.

(*E*)-4-chloro-2-(2-(5-methylthiophen-2-yl)vinyl)phenyl dimethylcarbamate (**10**): 26 mg (isolated 41%), yellow oil; *Rf* (40% PE/E) = 0.41; ^1^H NMR (CDCl_3_, 600 MHz) *δ*/ppm: 7.22–7.06 (m, 4H), 6.91–6.82 (m, 2H), 6.66–6.61 (m, 1H), 3.21 (s, 3H), 3.04 (s, 3H), 2.47 (s, 3H); ^13^C NMR (CDCl_3_, 75 MHz) *δ*/ppm: 148.8, 141.0, 139.4, 129.9, 128.1, 126.7, 125.9, 125.8, 125.6, 123.8, 123.0, 120.7, 36.8, 36.4, 15.7 (1 quaternary C is missing); HRMS (ESI) (*m*/*z*) for C_16_H_16_ClNO_2_S: [M + H]^+^_calcd_ = 321.0590, and [M + H]^+^_measured_ = 321.0594.


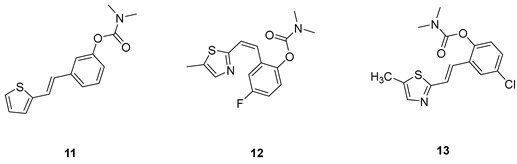


(*E*)-3-(2-(thiophen-2-yl)vinyl)phenyl dimethylcarbamate (**11**): 19 mg (isolated 39%), yellow oil; *Rf* (40% PE/E) = 0.45; ^1^H NMR (CDCl_3_, 600 MHz) *δ*/ppm: 7.24 (t, *J* =7.6, 7.2 Hz, 1H), 7.21–7.18 (m, 1H), 7.18–7.16 (m, 1H), 7.14 (d, *J* = 16.1 Hz, 1H), 7.12 (d, *J* = 5.2 Hz, 1H), 6.99 (d, *J* = 3.3 Hz, 1H), 6.94–6.91 (m, 2H), 6.82 (d, *J* = 15.9 Hz, 1H), 3.04 (s, 3H), 2.95 (s, 3H); ^13^C NMR (CDCl_3_, 150 MHz) *δ*/ppm: 153.8, 150.9, 141.6, 137.4, 128.4, 126.6, 126.5, 125.3, 123.5, 122.4, 121.5, 119.8, 118.2, 35.7, 35.4; HRMS (ESI) (*m*/*z*) for C_15_H_16_NO_2_S: [M + H]^+^_calcd_ = 273.0824, and [M + H]^+^_measured_ = 273.0826.

(*E*)-4-fluoro-2-(2-(5-methylthiazol-2-yl)vinyl)phenyl dimethylcarbamate (**12**): 30 mg (isolated 4 %), yellow oil; *Rf* (40% PE/E) = 0.13; ^1^H NMR (CDCl_3_, 300 MHz) *δ*/ppm: 7.52 (s, 1H), 7.32 (dd, *J* = 8.6, 2.5 Hz, 1H), 7.28 (d, *J* = 2.5 Hz, 1H), 7.14 (d, *J* = 8.7 Hz, 1H), 6.76 (d, *J* = 11.8 Hz, 1H), 6.38 (d, *J* = 11.8 Hz, 1H), 2.95 (s, 3H), 2.91 (s, 3H), 2.58 (s, 3H); ^13^C NMR (CDCl_3_, 75 MHz) *δ*/ppm: 157.8, 146.8, 142.9, 140.7, 132.5, 130.6 129.7, 129.0, 128.0, 127.4, 124.6, 123.3, 122.4, 122.0, 35.7, 35.1, 18.6; MS (ESI) *m*/*z* (%, fragment): 304 (100).

(*E*)-4-chloro-2-(2-(5-methylthiazol-2-yl)vinyl)phenyl dimethylcarbamate (**13**): 29 mg (isolated 45%), yellow oil; *Rf* (40% PE/E) = 0.39; ^1^H NMR (CDCl_3_, 600 MHz) *δ*/ppm: 7.09 (d, *J* = 3.0 Hz, 1H), 7.55 (m, 2H), 7.01–6.99 (m, 1H), 6.97 (d, *J* = 16.0 Hz, 1H), 6.81 (dd, *J* = 8.5, 2.8 Hz, 1H), 3.19 (s, 3H), 3.03 (s, 3H), 2,61 (s, 3H); ^13^C NMR (CDCl_3_, 150 MHz) *δ*/ppm: 157.0, 154.9, 142.9, 142.7, 127.6, 126.5, 124.6, 123.9, 123.6, 121.9, 114.1, 110.3, 55.7, 36.9, 36.5, 20.2; HRMS (ESI) (*m*/*z*) for C_15_H_15_ClN_2_O_2_S: [M + H]^+^_calcd_ = 322.0543, and [M + H]^+^_measured_ = 322.0545.

### 3.3. Cholinesterase Inhibitory Activity

The cholinesterase inhibitory activity of the tested compounds was measured using the modified Ellman’s method. Acetylcholinesterase (AChE, E.C. from electric eel) and butyrylcholinesterase (BChE, E.C. from equine serum) were purchased from Sigma Aldrich (St. Louis, MO, USA). The substrates used in this assay, including acetylthiocholine iodide (ATChI) and *S*-butyrylthiocholiniodide (BTChI), as well as trisma base for buffer preparation, were purchased from Sigma Aldrich. The reagent used for colorimetric detection, 5,5’-dithiobis-2-nitrobenzoic acid (DTNB, Ellman’s reagnt, Zwijndrecht, Netherland). Galantamine was used as a reference standard (Sigma Aldrich). The reaction mixture was composed of 180 µL of Tris buffer (50 mM, pH 8.0), 10 µL of AChE/BChE enzyme (concentration in reaction mixture: 0.03 U/mL, prepared in 20 mM Tris buffer, pH 7.5), 10 µL of tested compound dissolved in ethanol (concentration in reaction mixture was in the range of 100 nM–250 µM, depending on solubility) and 10 µL of DTNB (concentration of 0.3 mM in the reaction mixture, prepared in Tris buffer). The reaction started with the addition of 10 µL of ATChI/BTChI substrate Caco2 permeability (concentration in reaction mixture: 0.5 mM, prepared in Tris buffer), and the developing yellow color was measured at 405 nm over 6 min using a 96-well microplate reader (Bio Tek 800TSUV Absorbance Reader, Agilent, Santa Clara, CA, USA). A control measurement was taken without the inhibitor, which was replaced by 10 µL of buffer. For each measurement, non-enzymatic hydrolysis was measured as a blank, where a 10 µL of buffer replaced the enzyme. The percentage enzyme inhibition was calculated from the measured data using the following equation: Inhibition (%) = [(*A*_C_–*A*_T_)/*A*_C_]·100.

A_C_ represents the enzyme activity in the control measurement (without inhibitor), and *A*_T_ is the enzyme activity in the test sample. Inhibition data were used to calculate the IC_50_ values via nonlinear fit of the inhibitor concentration vs. response. The inhibitory activity of ethanol was also measured, and its contribution to inhibition was subtracted. All experiments were run in triplicate. The IC_50_ values are represented as mean values of three measurements ± standard deviations.

### 3.4. Metal–Chelate or Metal–Associate Formation

The absorption spectra used to determine the molar absorptivity and to evaluate the complex formation constant were recorded with a Specord S600 spectrophotometer (Analytic Jena GmbH, Jena, Germany) in the 190–900 nm wavelength range. During the titration, the ligand concentration was increased at a constant Fe(III) content. Also, the iron(III) concentration was increased at a constant ligand content, but this method proved to be less informative than the other.

### 3.5. Computational Study

The geometries of ligands **1** and **7** were prepared for the docking study via geometry optimization using the M062X/6-31+G(d,p) model with the Gaussian16 program package [47], followed by adding Gasteiger charges, detecting rotatable bonds, setting torsional degrees of freedom and identifying aromatic carbons. Crystallographic data for BChE, PDB ID: 3DJY, were obtained from the Protein Data Bank [48] and prepared for docking by removing non-amino acid residues, adding polar hydrogens, and assigning Kollman charges. Molecular docking was performed using Autodock4.0 [49], utilizing the Lamarckian Genetic Algorithm, generating 25 docking poses for each ligand and with enzyme residues kept rigid throughout the process.

The most stable complexes obtained through docking were used as starting structures for the molecular dynamic (MD) study. The protein–ligand complexes were solvated in a truncated octahedron OPC water box and neutralized with Cl^−^ ions using the Amber16 software package [50], applying the ff14SB force field [51] for the protein and the GAFF force field [52] for the ligands. Ligand partial charges were derived using the RESP procedure. Equilibration of all three systems included energy minimizations and short (20 ps) MD simulations, with harmonic restraints gradually reduced to zero and adjustments made to volume and temperature. The target values for temperature and pressure were set to 300 K and 1 atm, respectively. Production MD simulations were then conducted without constraints for 100 nanoseconds under NPT conditions (300 K and 1 atm).

## 4. Conclusions

Considering our previous experience in the design of new cholinesterase inhibitors, especially resveratrol analogs, the basic stilbene skeleton was used as a structural unit for new carbamates in this research. Inhibitory activity was tested toward the enzymes acetylcholinesterase (AChE) and butyrylcholinesterase (BChE) via newly prepared carbamates **1**–**13**. In the tested group of compounds, the leading inhibitors were **1** and **7**, which achieved excellent selective inhibitory activity for BChE, with IC_50_ values of 0.12 ± 0.09 μM and 0.38 ± 0.01 μM, respectively. Both were much more active than the standard inhibitor, galantamine against BChE. Molecular docking of the most promising inhibitor candidates, compounds **1** and **7**, revealed that stabilizing interactions between the active site residues of BChE and the ligands involve π-stacking, alkyl-π interactions and, depending on the orientation of the carbamate group, either an H-bond between the ligand’s carbonyl oxygen and the catalytic serine or another alkyl-π attraction, this time between the dimethylamino fragment of carbamate group with Trp82 and Tyr332. MD analysis confirmed the stability of the obtained complexes. Based on the obtained in silico, experimental and computational results on biological activity in the present work, novel carbamates **1**–**13** represent potential selective BChE inhibitors as new therapeutics for neurological disorders, and they can be incorporated into the chemical libraries used in high-throughput screening and drug development. Complex formation studies indicated that some selected representatives of these compounds bind to Fe^3+^ ions, which confirmed their coordination abilities, mainly due to the carboxylic moieties of the carbamate part.

## Figures and Tables

**Figure 3:**
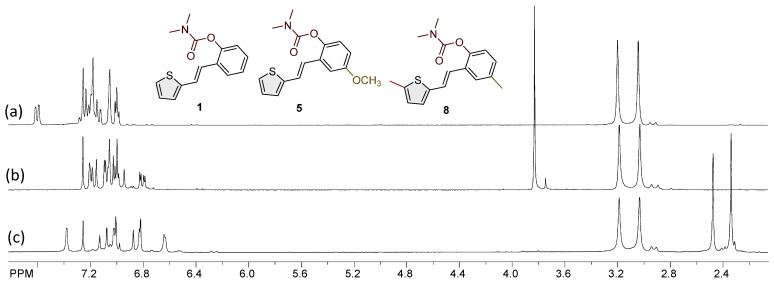
The portions of the ^1^H NMR spectra of the most active carbamates as selective BChE inhibitors were compared: (**a**) **1**, (**b**) **5**, and (**c**) **8**.

**Table 1:** Cholinesterase inhibition (AChE and BChE) for carbamate derivatives **1**–**13**.

Carbamate Derivative	AChE Inhibition * (%)	AChE IC_50_ (μM)	BChE Inhibition * (%)	BChE IC_50_ (μM)
**1**	30.0 (100)	-	85.0 (50)	0.12 ± 0.09
**2**	16.7 (100)	-	85.0 (10)	1.05 ± 0.09
**3**	12.6 (100)	-	85.0 (100)	1.48 ± 0.36
**4**	35.0 (100)	-	85.3 (25)	2.83 ± 0.03
**5**	11.0 (100)	-	84.0 (100)	11.59 ± 0.18
**6**	1.0 (100)	-	84.4 (25)	0.90 ± 0.14
**7**	17.7 (100)	-	86.3 (25)	0.38 ± 0.01
**8**	25.2 (100)	-	72.5 (100)	25.50 ± 0.21
**9**	23.6 (100)	-	85.2 (100)	15.05 ± 2.32
**10**	27.0 (100)	-	85.7 (100)	23.17 ± 3.78
**11**	19.0 (100)	-	83.4 (100)	11.37 ± 2.48
**12**	23.9 (100)	-	81.4 (100)	23.39 ± 2.43
**13**	37.5 (100)	-	84.1 (50)	3.77 ± 0.13
Galantamine	90.0 (4.5)	0.15 ± 0.07	90.1 (25)	7.54 ± 1.64

* The numbers shown in parentheses represent the maximal concentrations tested in μM.

**Table 2:** In silico study for more indicators of absorption, distribution, metabolism, excretion and toxicity (ADME(T)) in the human body of carbamates **1** and **7**.

Property	Model Name	1	7	Bambuterol	Rivastigmine	Unit
**Absorption**	Water solubility	−4.1	−4.43			log mol/L
	Caco2 permeability	1.911	1.843			log Papp in 10^−6^ cm/s
	Intestinal absorption	90.749	91.133			% Absorbed
	Skin permeability	−2.687	−2.689			log Kp
	P−glycoprotein substrate	No	No			
	P−glycoprotein I inhibitor	No	No			
	P−glycoprotein II inhibitor	No	No			
**Distribution**	VDss (human)	0.313	0.412	0.401	0.451	log L/kg
	Fraction unbound (human)	0.033	0.023	−	−	Fu
	BBB permeability	0.31	0.322	0.707	0.968	log BB
	CNS permeability	−0.941	−0.955	−1.95	−0.801	log PS
**Metabolism**	CYP2D6 substrate	No	No			
	CYP3A4 substrate	Yes	Yes			
	CYP1A2 inhibitor	Yes	Yes			
	CYP2C19 inhibitor	Yes	Yes			
	CYP2C9 inhibitor	Yes	No			
	CYP2D6 inhibitor	No	No			
	CYP3A4 inhibitor	No	No			
**Excretion**	Total clearance	0.167	0.111			log mL/min/kg
	Renal OCT2 substrate	No	No			Yes/No
**Toxicity**	AMES toxicity	No	No			Yes/No
	Max. tolerated dose (human)	0.245	0.166			log mg/kg/day
	hERG I inhibitor	No	No			
	hERG II inhibitor	No	Yes			
	Oral rat acute toxicity (LD50)	2.579	2.618			mol/kg
	Oral rat chronic toxicity (LOAEL)	1.874	1.855			log mg/kg_bw/day
	Hepatotoxicity	No	Yes			
	Skin sensitization	No	No			
	T. pyriformis toxicity	1.785	1.927			log ug/L
	Minnow toxicity	0.379	−0.096			log mM

**Table 3:** Molar absorptivities at the longer-wavelength band maxima of the selected compounds.

Compound	*λ*/nm	*ε*/M^–1^cm^–1^
**1**	322	22,570
**3**	325	10,440
**6**	330	16,330
**7**	331	19,410

**Table 4:** The mutagenic potential of carbamates **1**–**13** through Lhasa M7 evaluation (green square—negative, red square—positive, white square—no data available); grey highlight—negative, orange highlight—positive, white—strongly negative).

Compound	ICH M7 Class	Derek Prediction	Sarah Prediction	Overall In Silico
**1**	Class 5	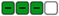	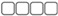	Negative
**2**	Class 5	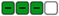	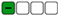	Negative
**3**	Inconclusive	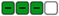	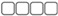	Inconclusive
**4**	Inconclusive	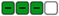	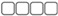	Inconclusive
**5**	Class 5	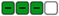	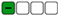	Negative
**6**	Class 5	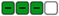	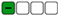	Negative
**7**	Class 5	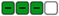	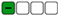	Negative
**8**	Class 5	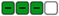	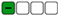	Negative
**9**	Class 5	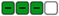	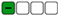	Negative
**10**	Class 5	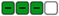	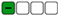	Negative
**11**	Class 3	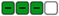	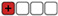	Positive
**12**	Class 5	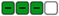	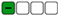	Negative
**13**	Class 5	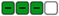	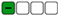	Negative

## Data Availability

The data presented in this study are available upon request from the corresponding authors. The data are not publicly available due to privacy.

## Supplementary Materials

The following supporting information can be downloaded at: https://www.mdpi.com/article/10.3390/molecules30020316/s1: ^1^H and ^13^C NMR spectra of carbamates **1**–**13** (Figures S1–S50). Mass spectra and HRMS analyses of carbamates **1**–**13** (Figures S51–S63). Table S1, free energies of binding obtained via docking. Cartesian coordinates of docked ligands and enzyme. Table S2, RMSD, RMSF, and Rg for protein–ligand complexes from MD simulations. Complex formation of biometals with bioactive carbamates **3** and **6** (Figures S64–S65). Dose–response curves for BChE inhibition by compounds **1**–**13** (Figures S66–S78).
