# Supplementary material for: Resveratrol-Based Carbamates as Selective Butyrylcholinesterase Inhibitors: Design, Synthesis, Computational Study and Biometal Complexation Capability
Source: Molecules. 2025 Jan 15;30(2):316. doi: 10.3390/molecules30020316 (PMC11767309; doi:10.3390/molecules30020316)
Supplement: Supplementary file 1 [file molecules-30-00316-s001.zip › molecules-3346430-supplementary.pdf]

Electronic Supporting Information

Article

Resveratrol-Based Carbamates as Selective Butyrylcholinesterase Inhibitors: Design, Synthesis, Computational Study and Biometal Complexation Capability

Maja Sviben [1], Ilijana Odak [2], Danijela Barić [3], Milena Mlakić [1], Ottó Horváth [4], Lajos Fodor [4], Sunčica Roca [5], Ivana Šagud [6,*] and Irena Škorić [1,*]

1 Department of Organic Chemistry, Faculty of Chemical Engineering and Technology, University of Zagreb, Trg Marka Marulića 19, HR-10 000 Zagreb, Croatia; mdragojev@fkit.unizg.hr (M.M.); mratajec@fkit.unizg.hr (M.S.)

2 Department of Chemistry, Faculty of Science and Education, University of Mostar, Matice Hrvatske bb, 88 000 Mostar, Bosnia and Herzegovina; ilijana.odak@fpmoz.sum.ba

3 Group for Computational Life Sciences, Division of Physical Chemistry, Ruđer Bošković Institute, Bijenička Cesta 54, HR-10 000 Zagreb, Croatia; dbaric@irb.hr

4 Environmental and Inorganic Photochemistry Research Group, Center for Natural Sciences, Faculty of Engineering, University of Pannonia, P.O. Box 158, H-8201 Veszprém, Hungary; horvath.otto@mk.uni-pannon.hu (O.H.); fodor.lajos@mk.uni-pannon.hu (L.F.)

5 NMR Center, Rudjer Bošković Institute, Bijenička Cesta 54, HR-10 000 Zagreb, Croatia; sroca@irb.hr

6 Croatian Agency for Medicinal Products and Medical Devices, Ksaverska Cesta 4, HR-10 000 Zagreb, Croatia

* Correspondence: ivana.sagud@halmed.hr (I.Š.); iskoric@fkit.unizg.hr (I.Š.)

<u>**Table of contents:**</u>

1. **^{1}H and ^{13}C NMR spectra of carbamates 1–13**
2. **Mass spectra and HRMS analyses of carbamates 1–13**
3. **Table S1, free energies of binding obtained by docking**
4. **Cartesian coordinates of docked ligands and enzyme**
5. **Table S2, RMSD, RMSF, and Rg for protein-ligand complexes from MD simulations**
6. **Complex formation of biometals with bioactive carbamates 3 and 6**
7. **Dose – response curves for three measurements of BChE inhibiton by compounds 1–13**

1. ^{1}H and ^{13}C NMR spectra of carbamates 1 – 13

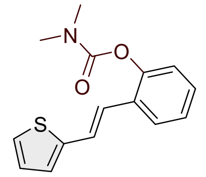

1

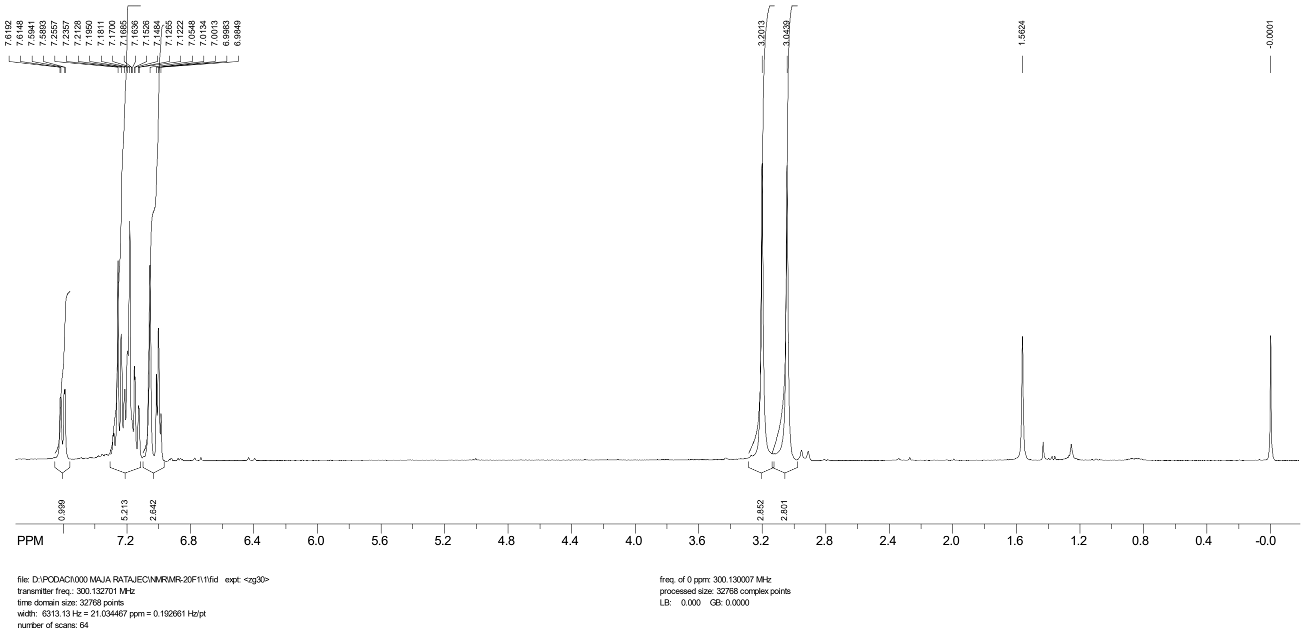

Figure S1. ^{1}H NMR spectrum ($CDCl_3$) of carbamate **1**.

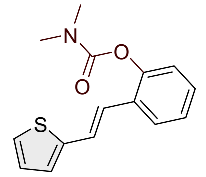

1

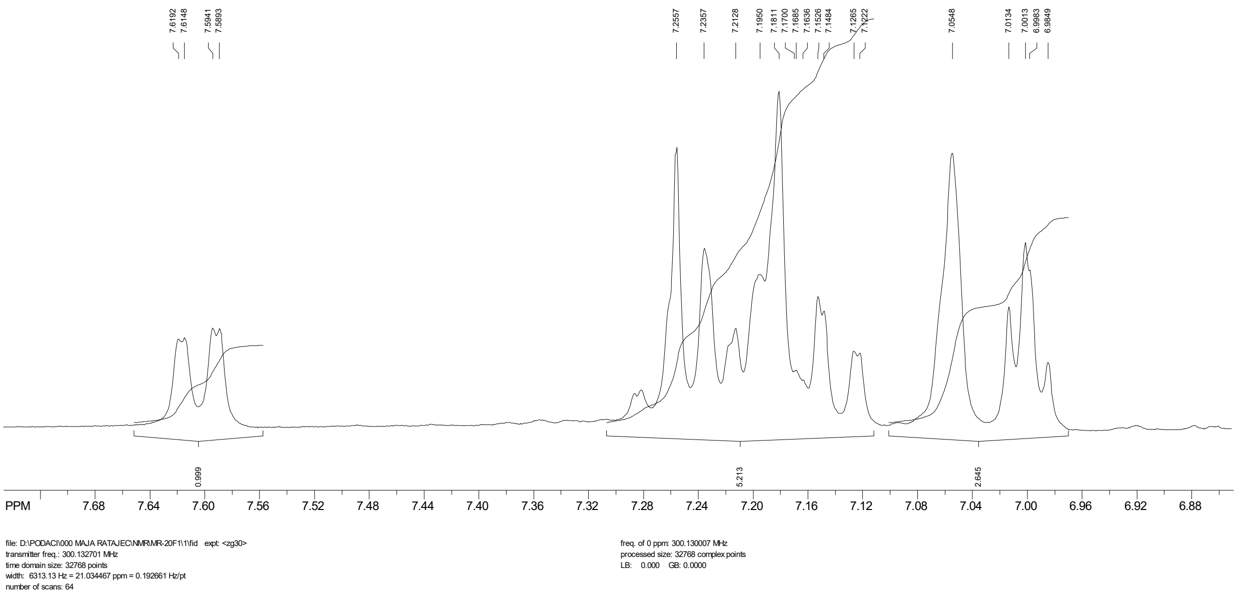

Figure S2. Aromatic part of the ^{1}H NMR spectrum ($CDCl_3$) of carbamate **1**.

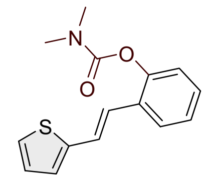

1

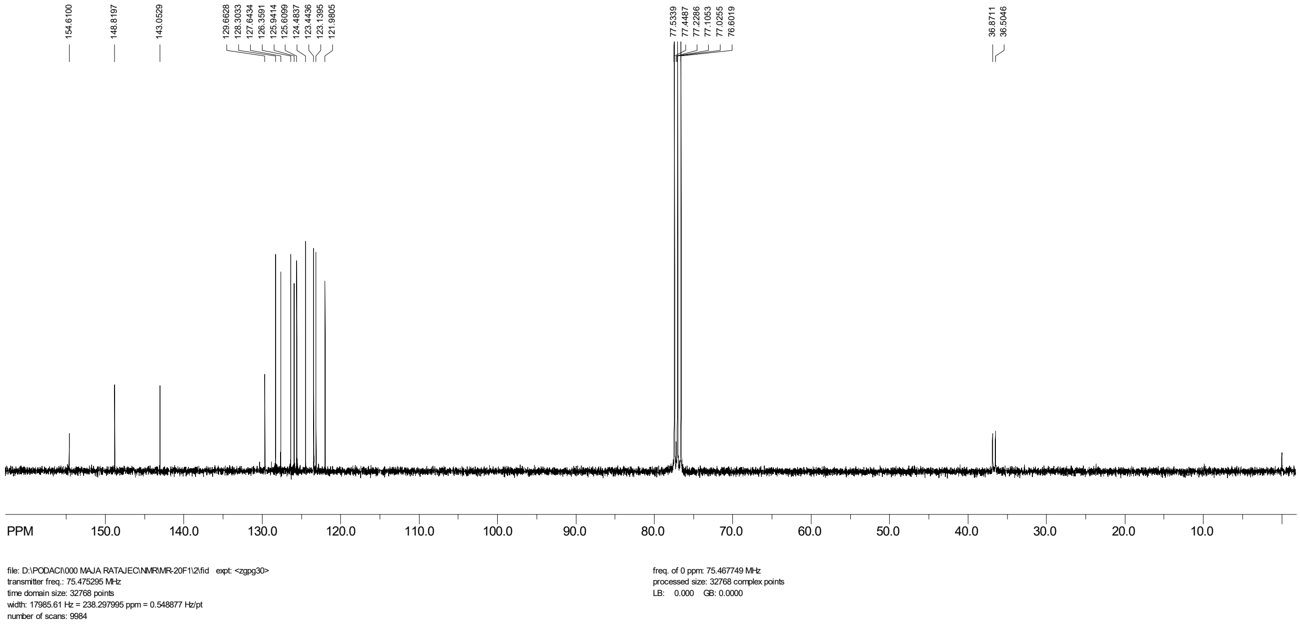

Figure S3. ^{13}C NMR spectrum ($CDCl_3$) of carbamate **1**.

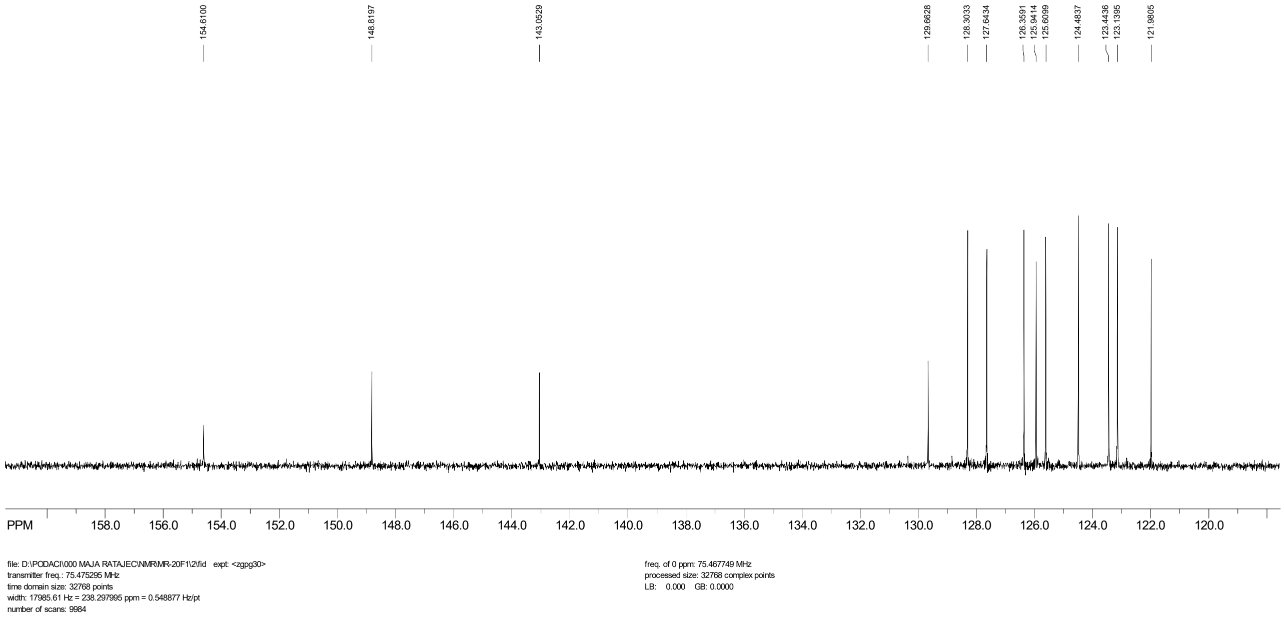

Figure S4. Aromatic part of the ^{13}C NMR spectrum ($CDCl_3$) of carbamate **1**.

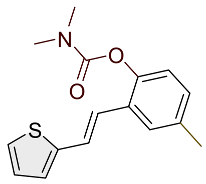

2

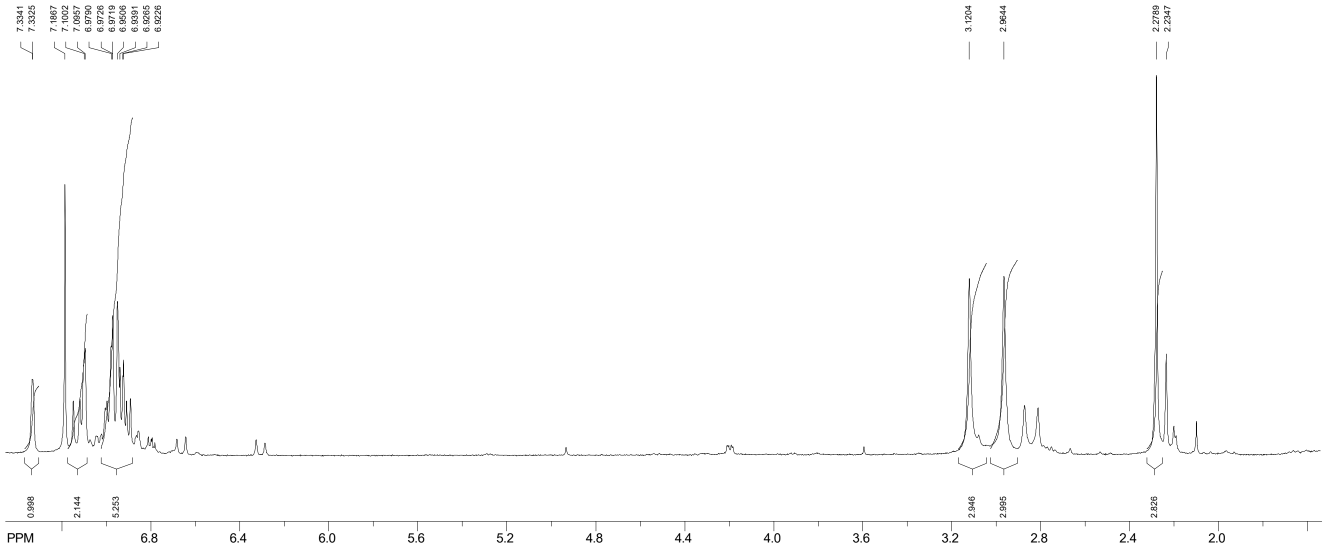

Figure S5. ^{1}H NMR spectrum ($CDCl_3$) of carbamate **2**.

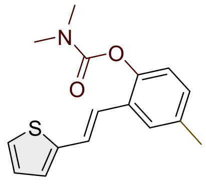

2

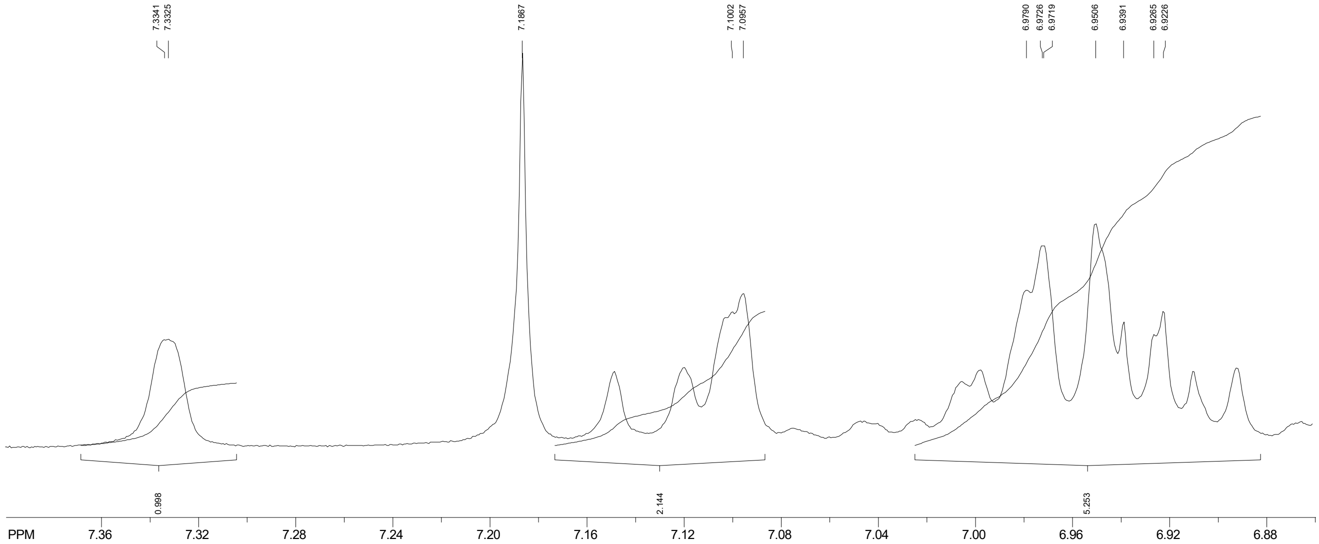

Figure S6. Aromatic part of the ^{1}H NMR spectrum ($CDCl_3$) of carbamate **2**.

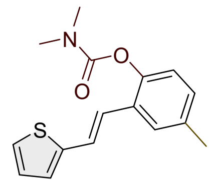

2

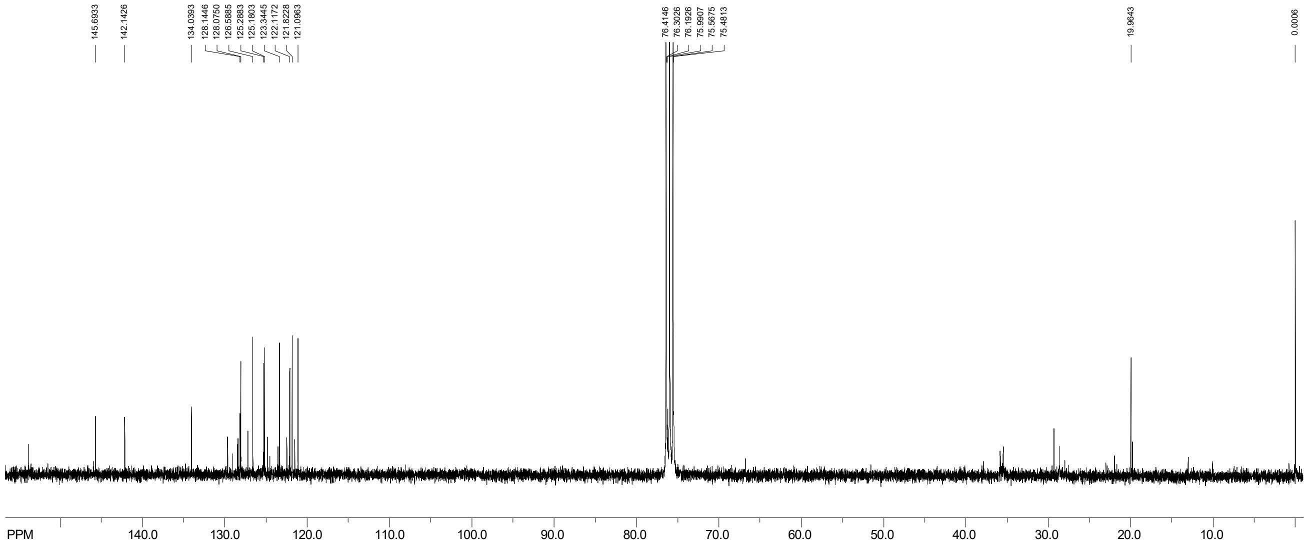

Figure S7. ^{13}C NMR spectrum ($CDCl_3$) of carbamate **2**.

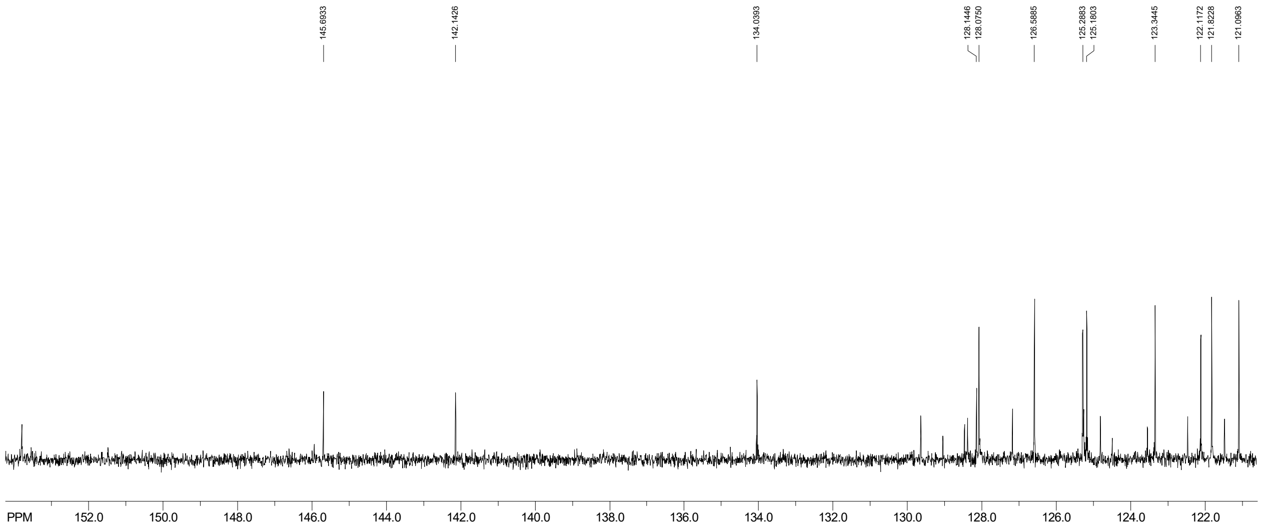

Figure S8. Part of the ^{13}C NMR spectrum ($CDCl_3$) of carbamate **2**.

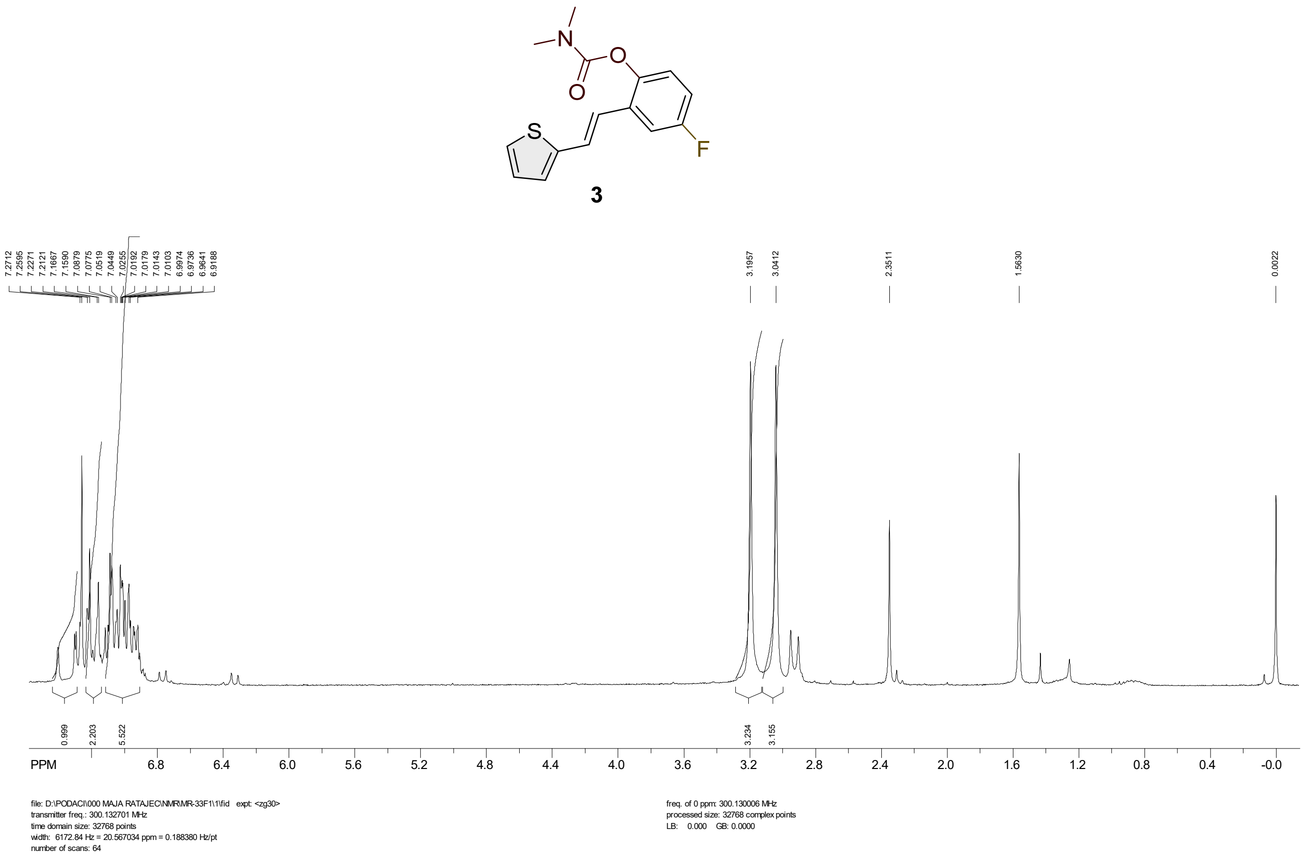

Figure S9. ^{1}H NMR spectrum ($CDCl_3$) of carbamate **3**.

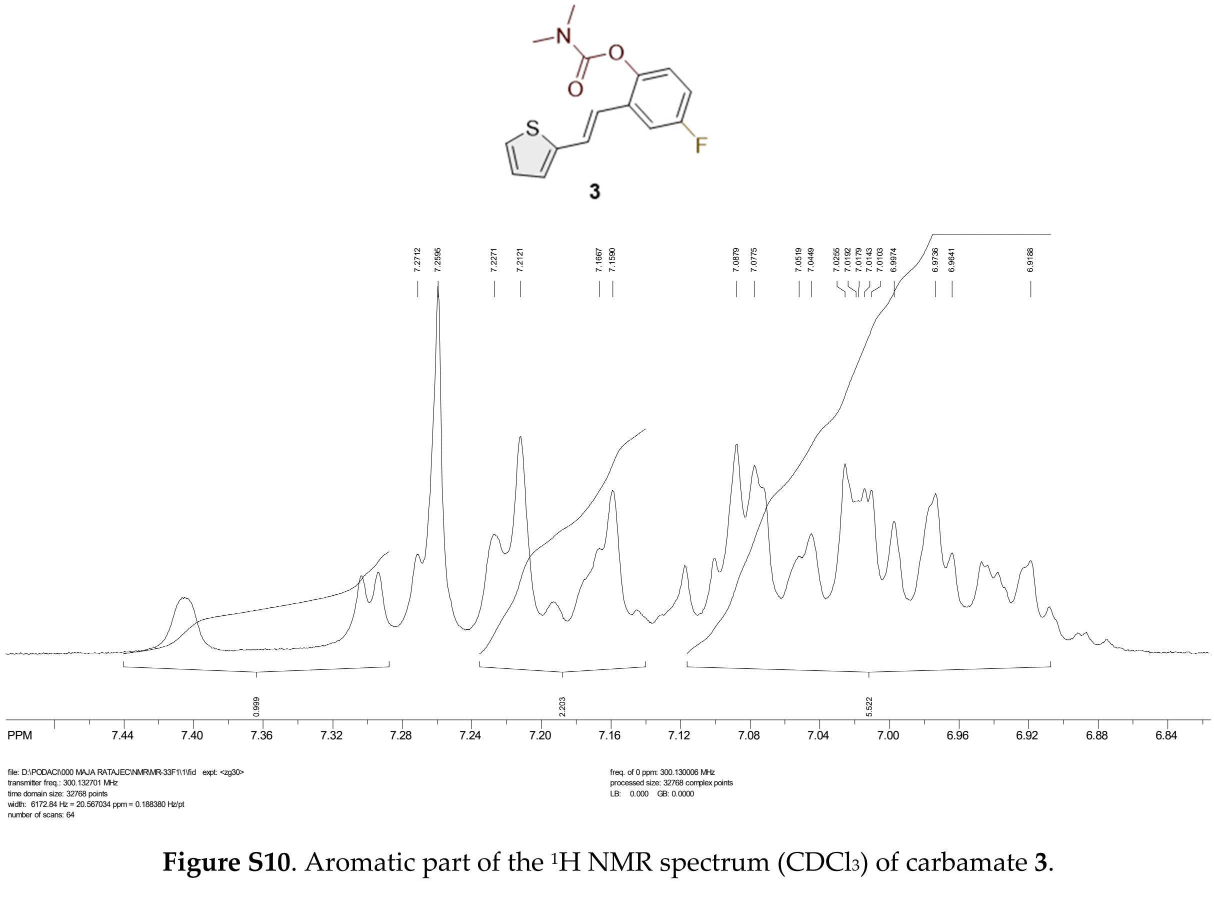

Figure S10. Aromatic part of the ^{1}H NMR spectrum ($CDCl_3$) of carbamate **3**.

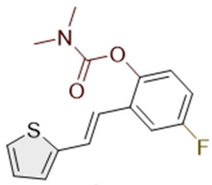

3

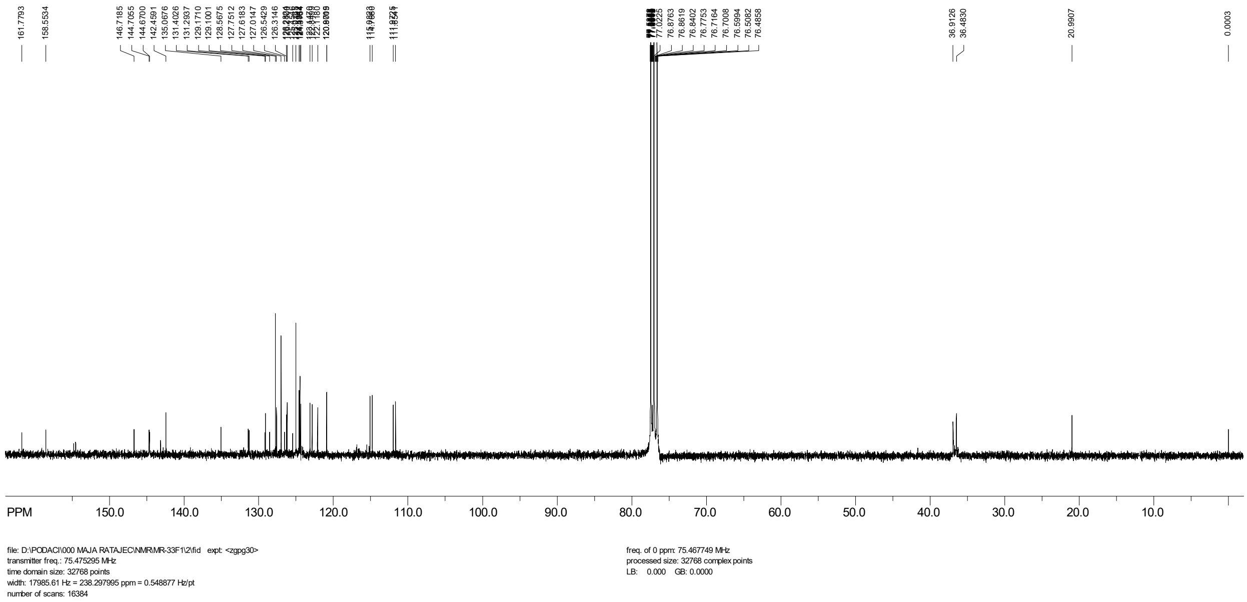

Figure S11. ^{13}C NMR spectrum ($CDCl_3$) of carbamate **3**.

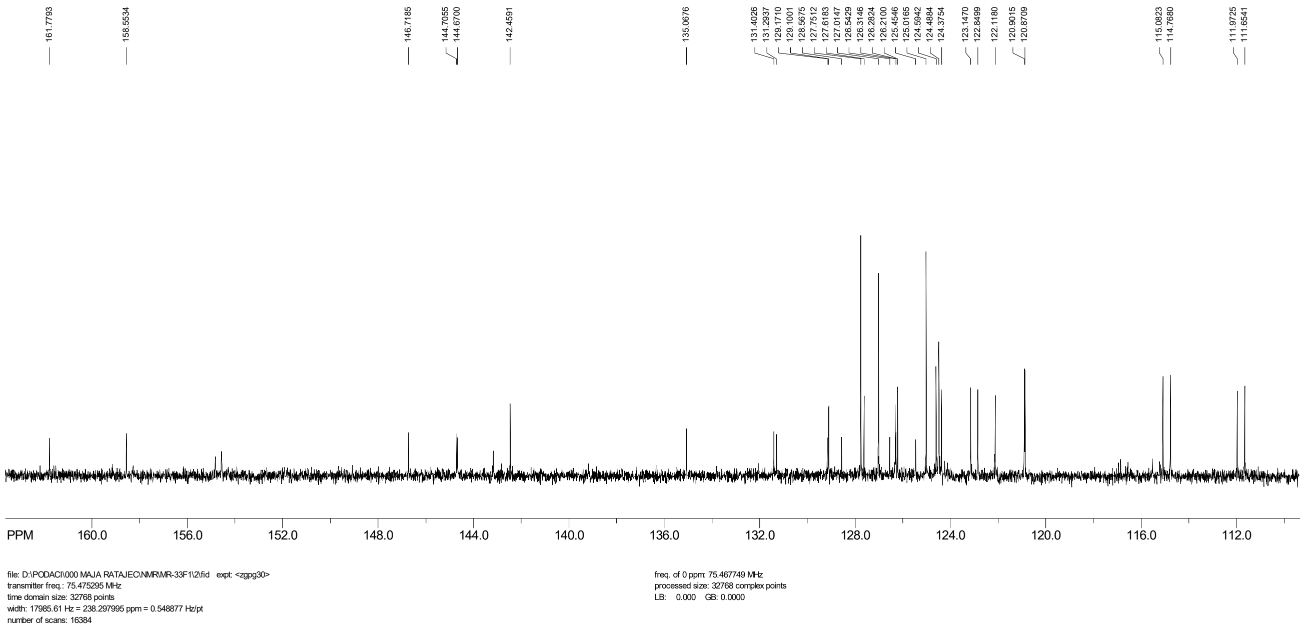

Figure S12. Aromatic part of the ^{13}C NMR spectrum ($CDCl_3$) of carbamate **3**.

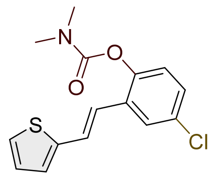

4

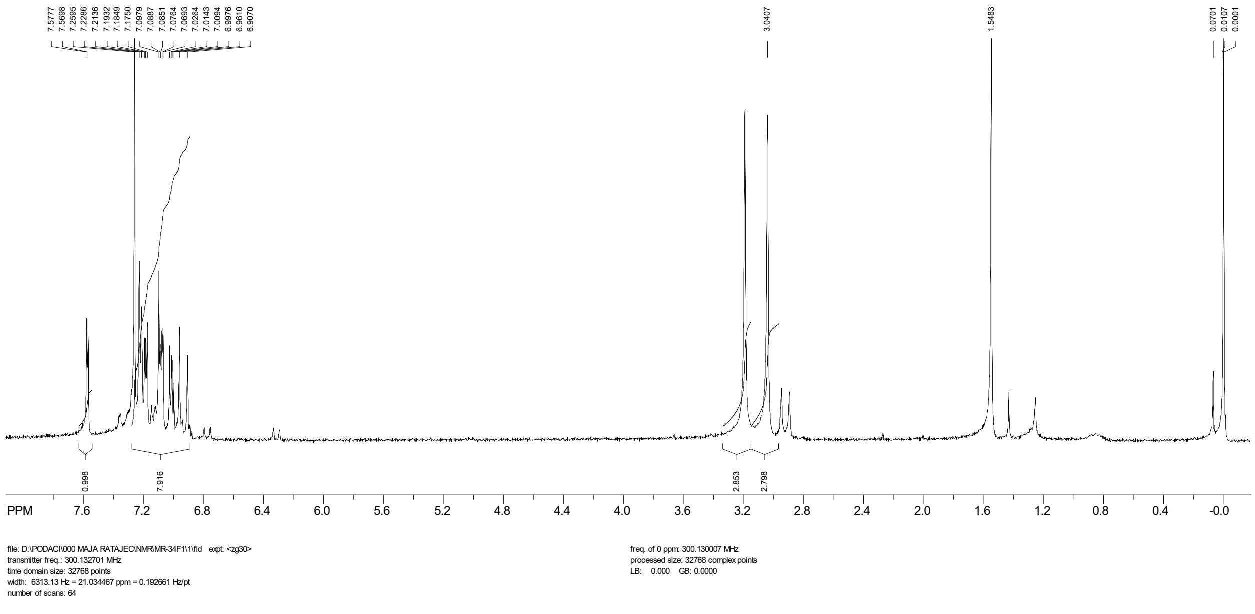

Figure S13. ^{1}H NMR spectrum ($CDCl_3$) of carbamate **4**.

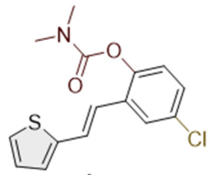

4

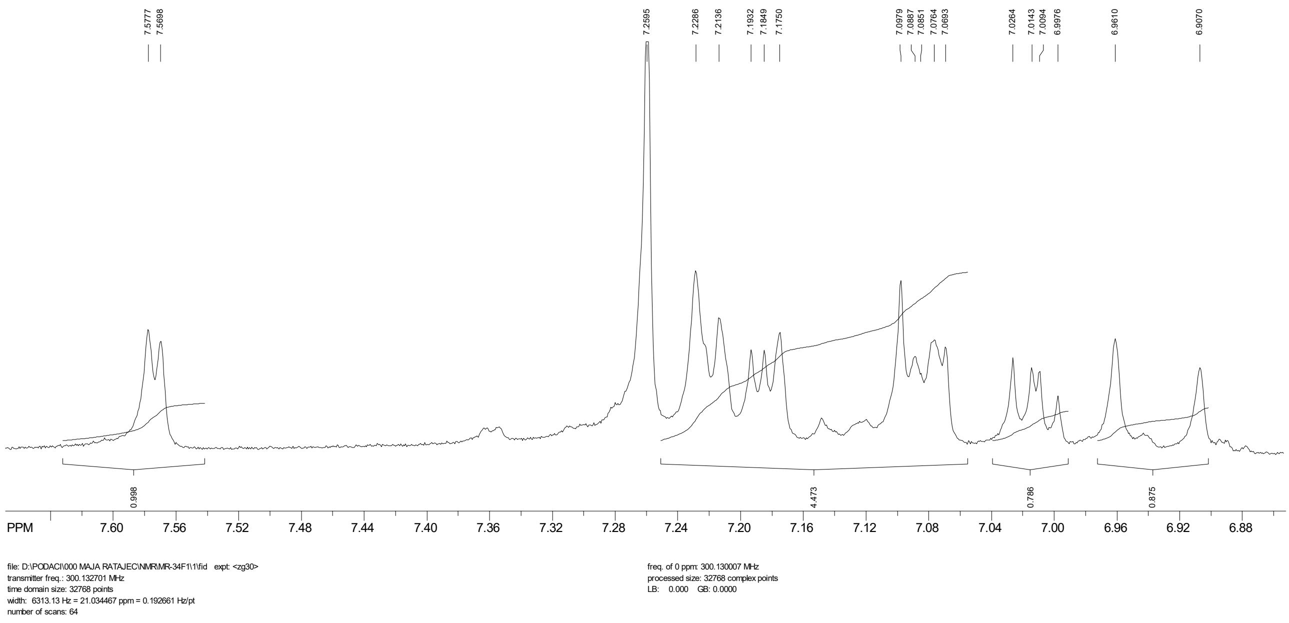

Figure S14. Aromatic part of the ^{1}H NMR spectrum ($CDCl_3$) of carbamate **4**.

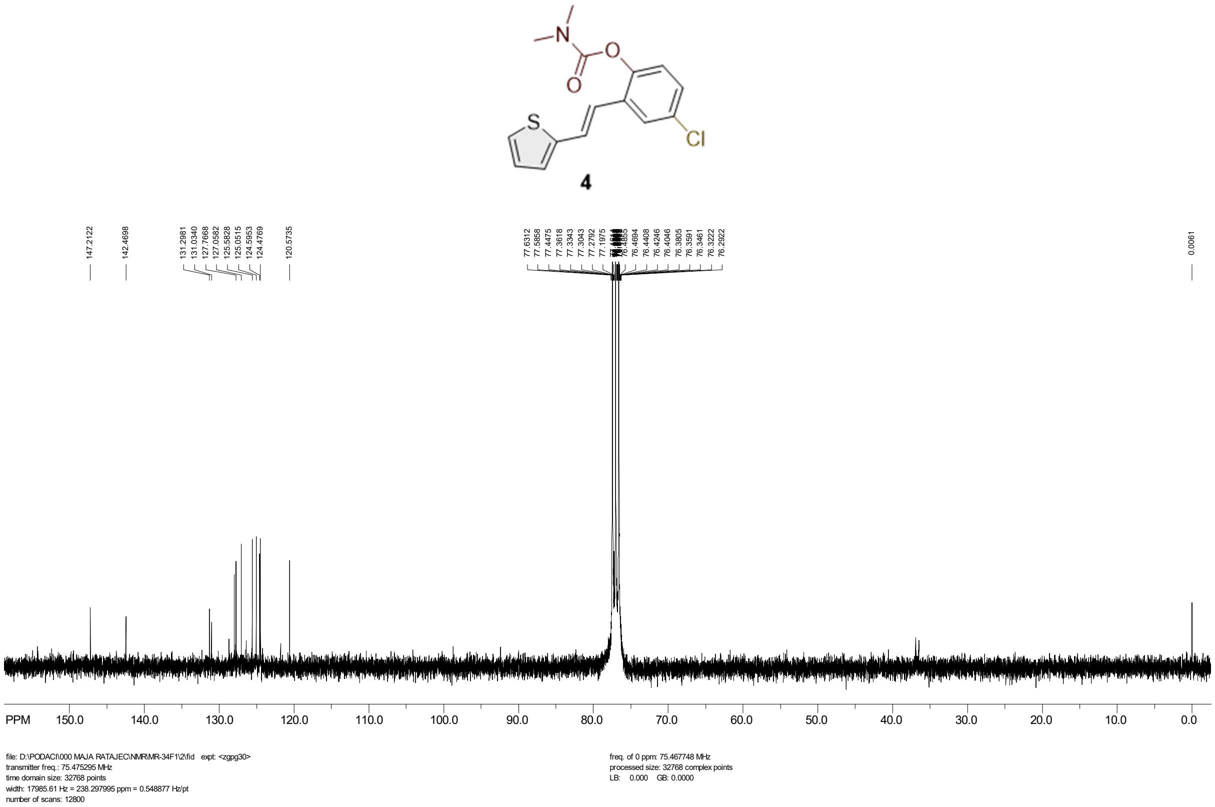

Figure S15. ^{13}C NMR spectrum ($CDCl_3$) of carbamate **4**.

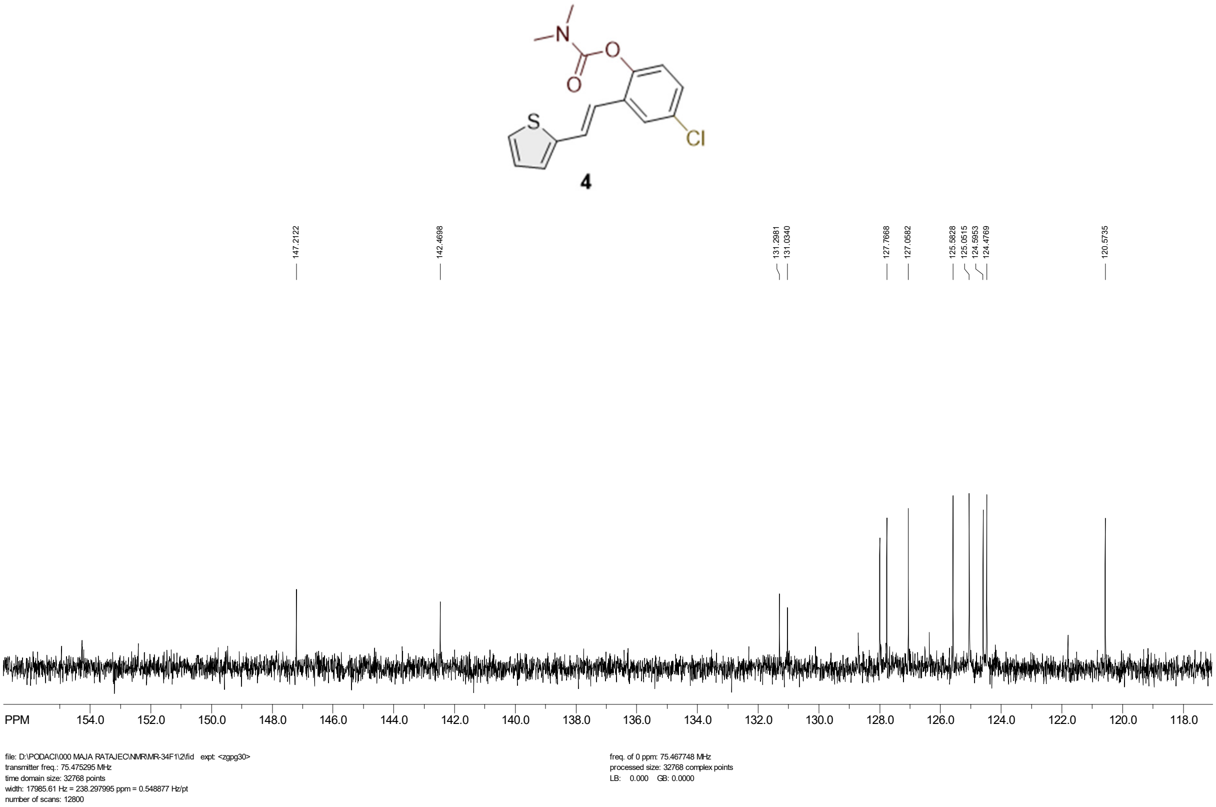

Figure S16. Aromatic part of the ^{13}C NMR spectrum ($CDCl_3$) of carbamate **4**.

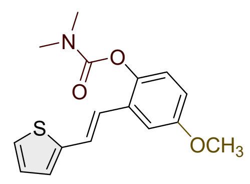

5

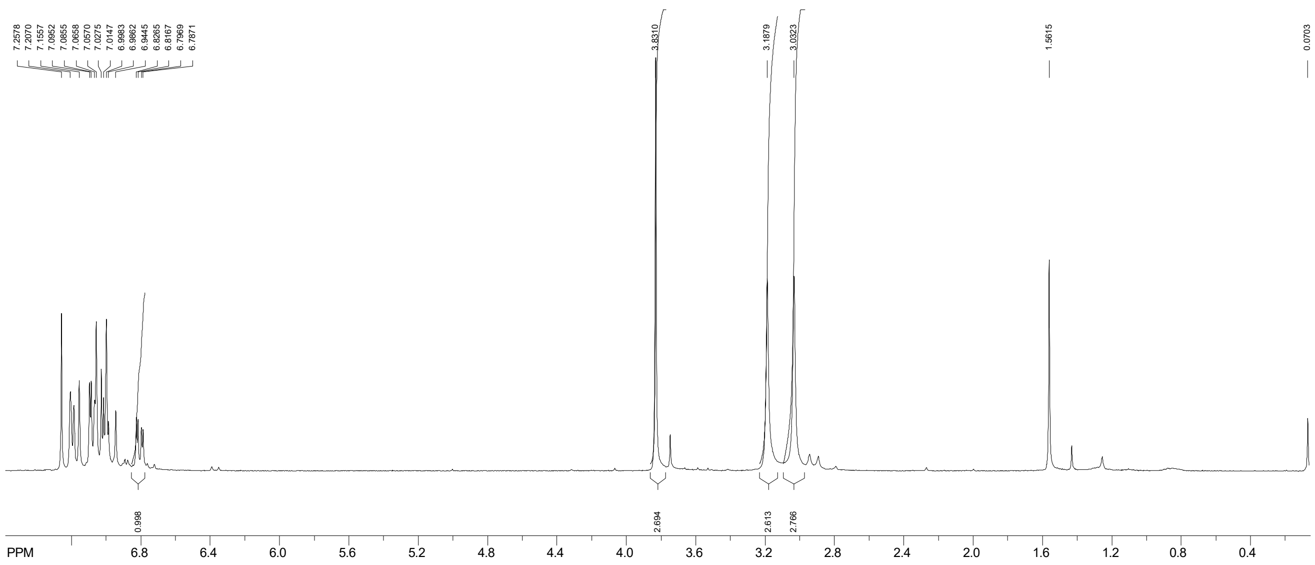

Figure S17. ^1H NMR spectrum ($CDCl_3$) of carbamate **5**.

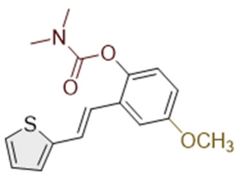

5

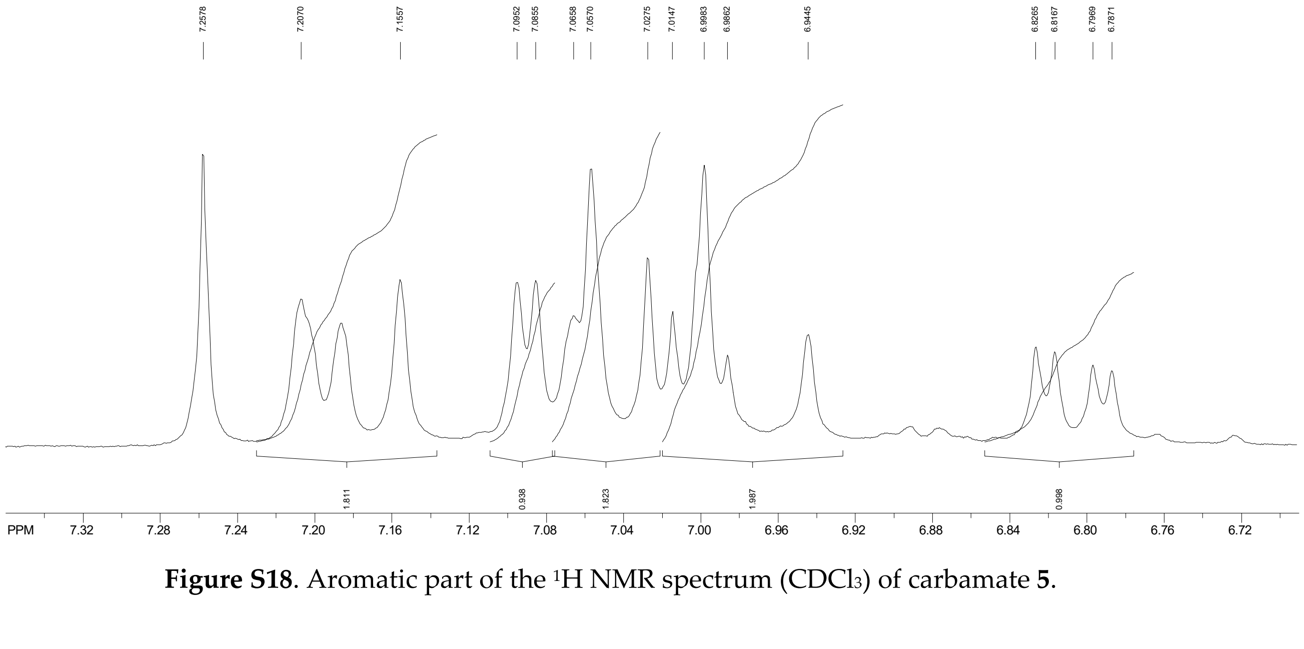

Figure S18. Aromatic part of the ^1H NMR spectrum ($CDCl_3$) of carbamate **5**.

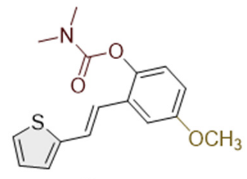

5

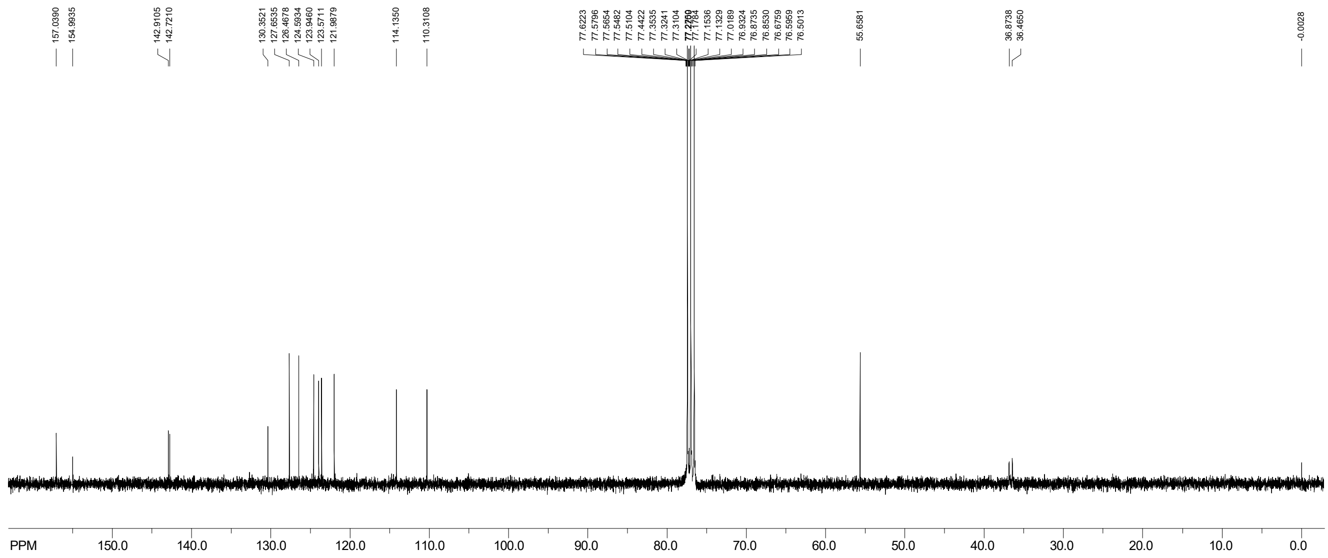

Figure S19. ^{13}C NMR spectrum ($CDCl_3$) of carbamate **5**.

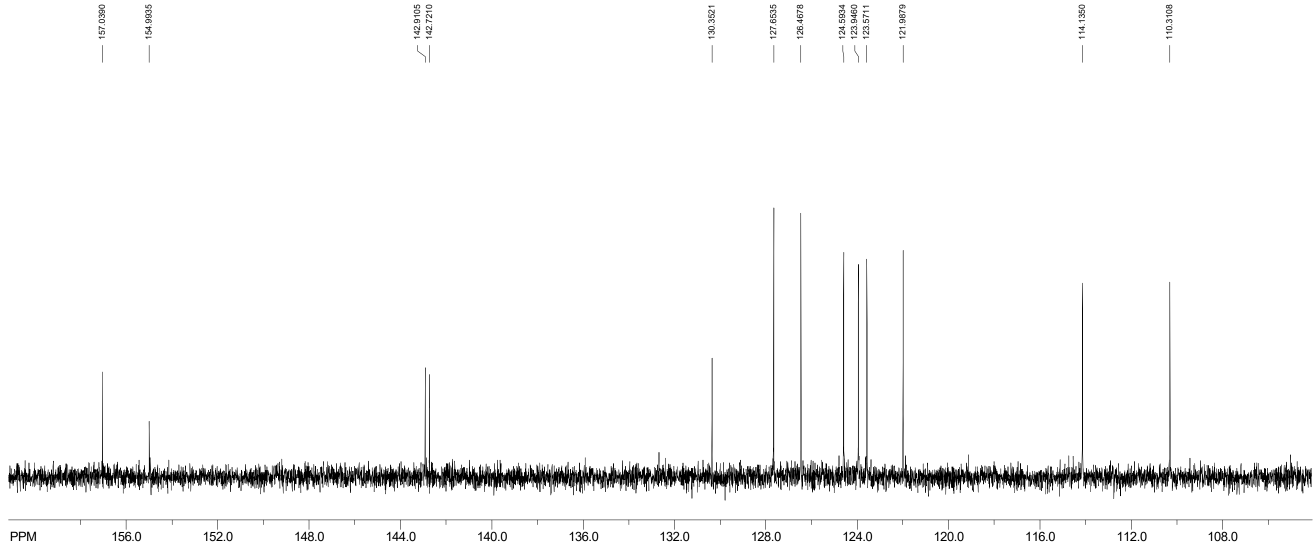

Figure S20. Aromatic part of the ^{13}C NMR spectrum ($CDCl_3$) of carbamate **5**.

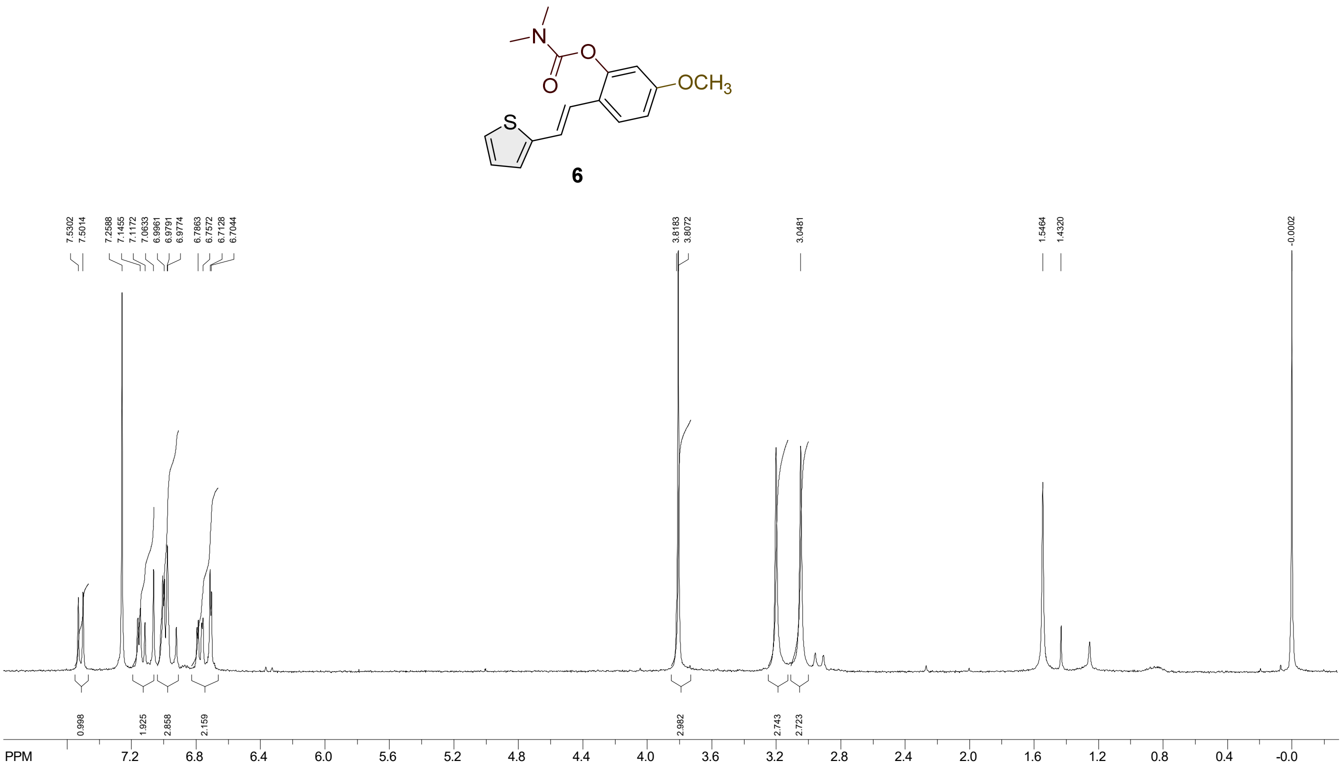

Figure S21. ^{1}H NMR spectrum ($CDCl_3$) of carbamate **6**.

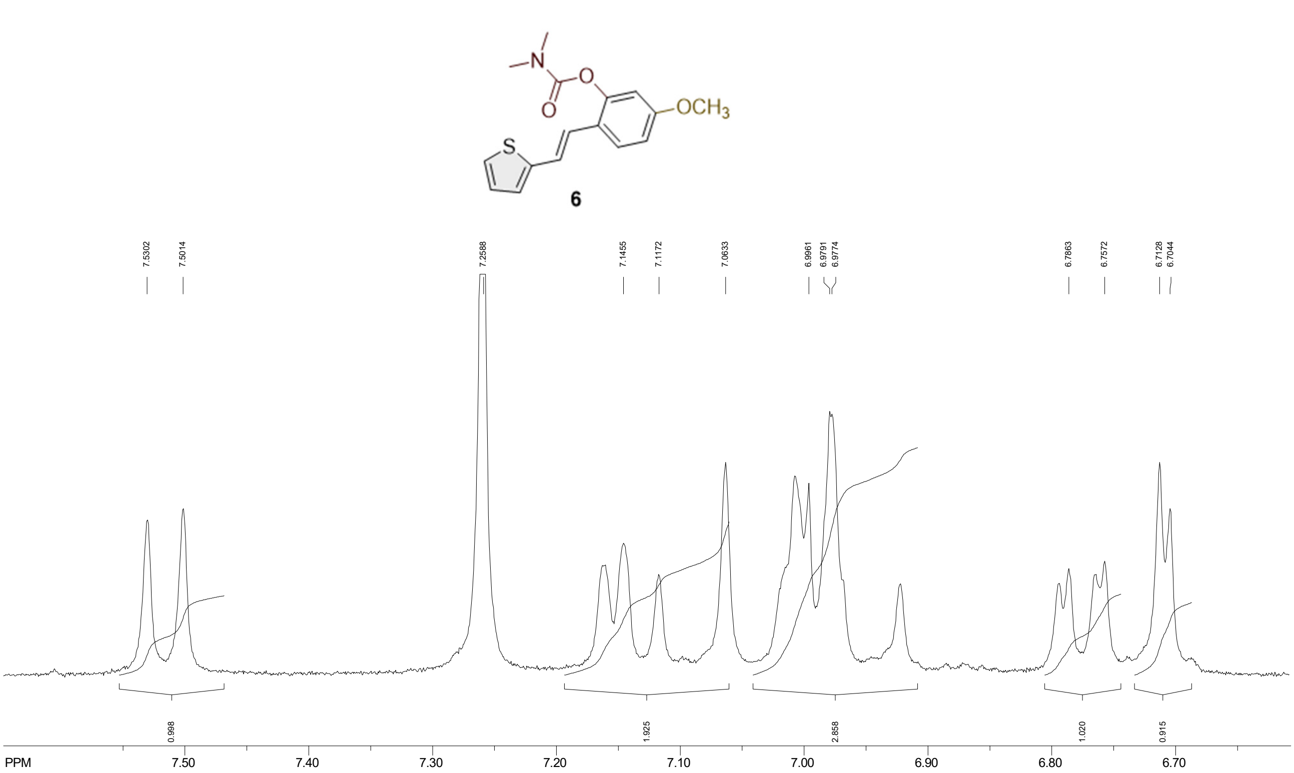

Figure S22. Aromatic part of the ^{1}H NMR spectrum ($CDCl_3$) of carbamate **6**.

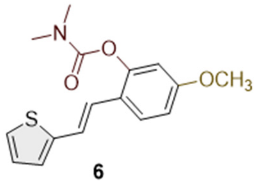

6

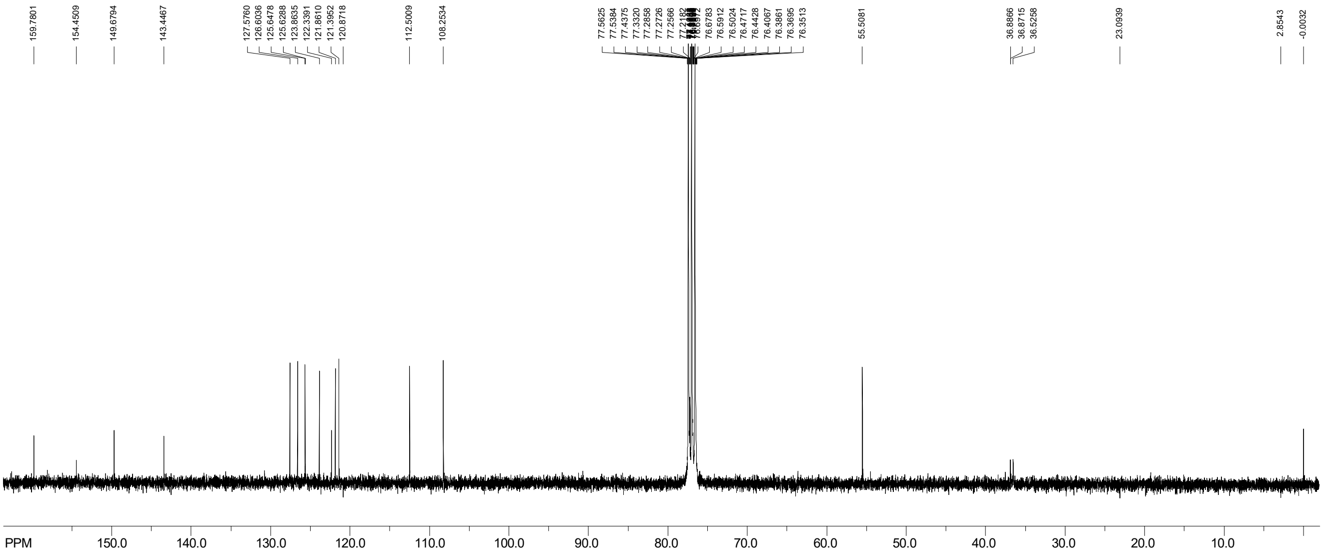

Figure S23. ^{13}C NMR spectrum ($CDCl_3$) of carbamate **6**.

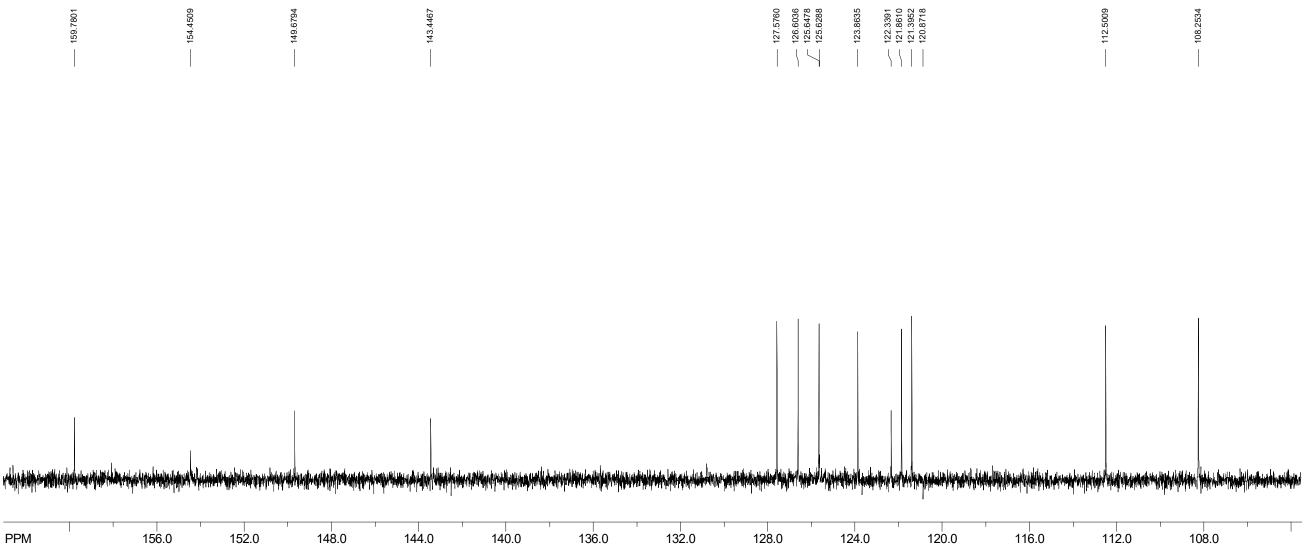

Figure S24. Aromatic part of the ^{13}C NMR spectrum ($CDCl_3$) of carbamate **6**.

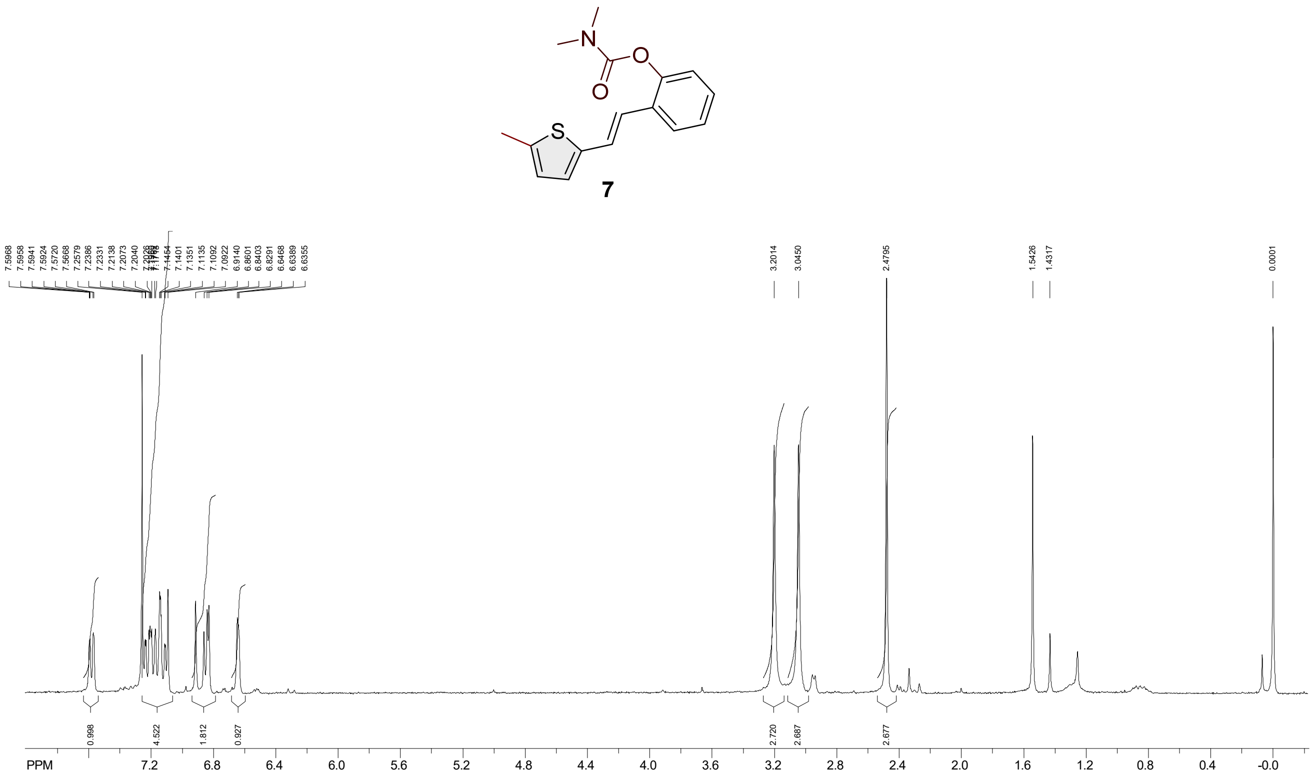

Figure S25. ¹H NMR spectrum ($CDCl_3$) of carbamate **7**.

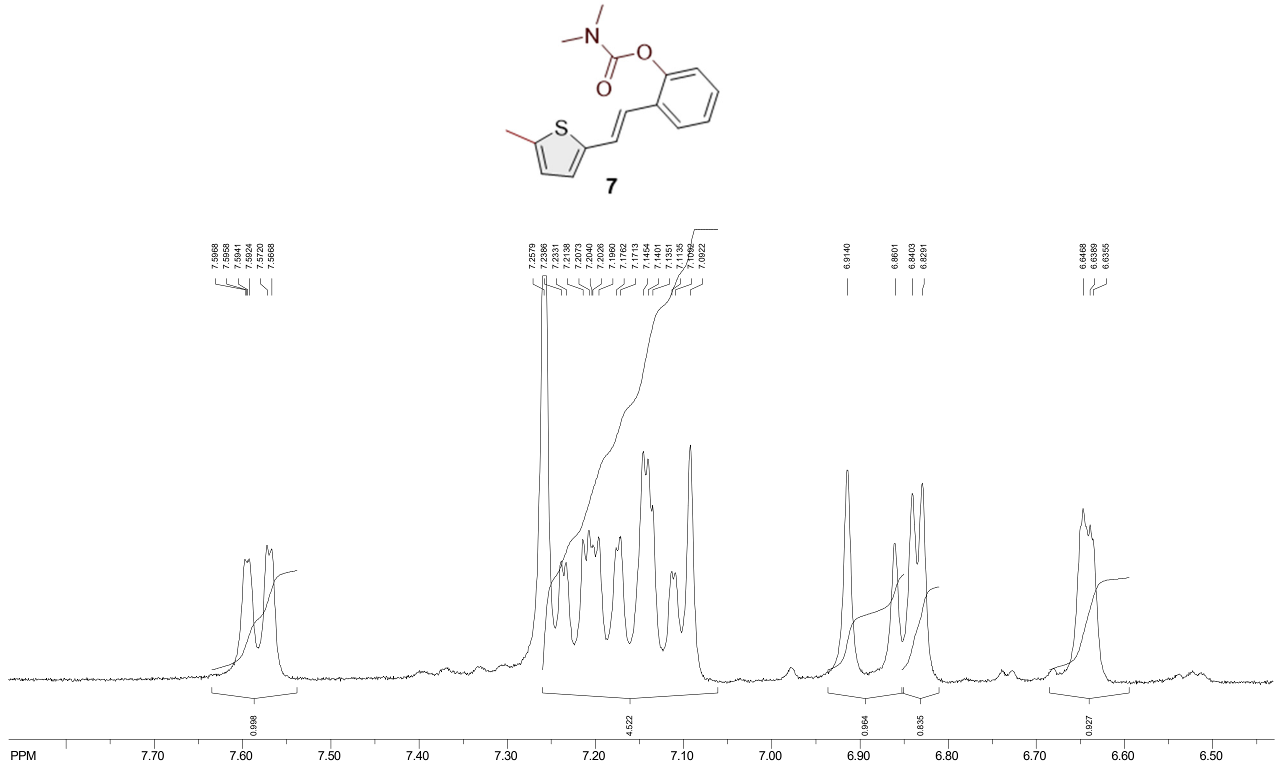

Figure S26. Aromatic part of the ¹H NMR spectrum ($CDCl_3$) of carbamate **7**.

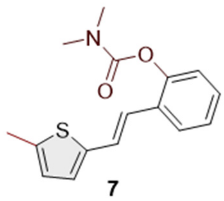

7

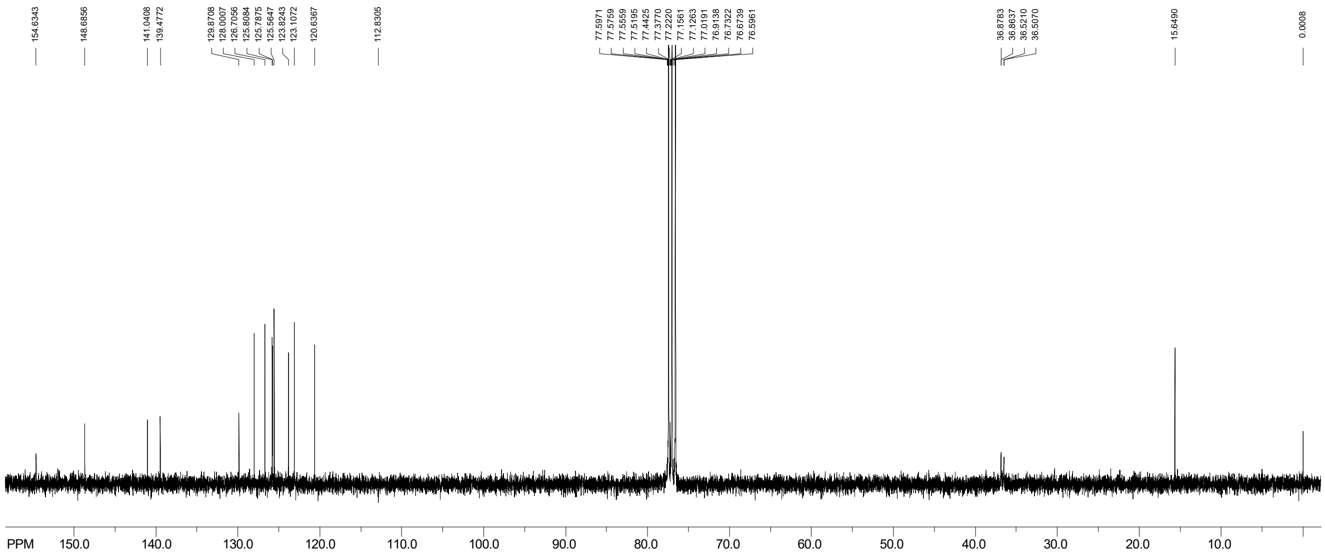

Figure S27. ^{13}C NMR spectrum ($CDCl_3$) of carbamate **7**.

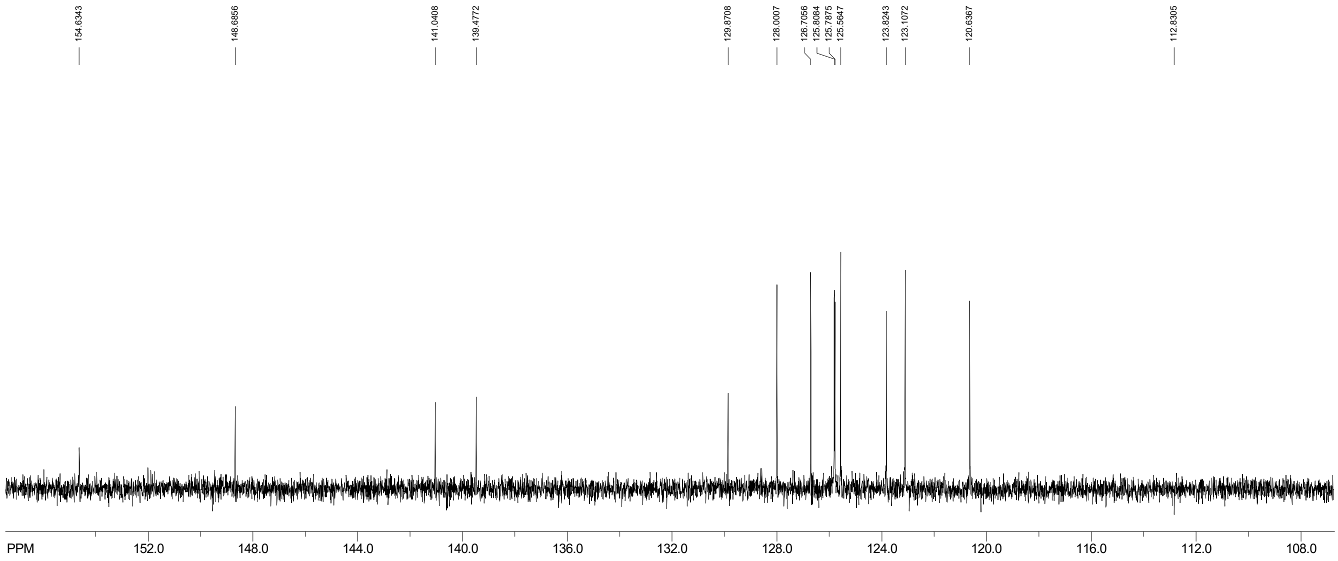

Figure S28. Aromatic part of the ^{13}C NMR spectrum ($CDCl_3$) of carbamate **7**.

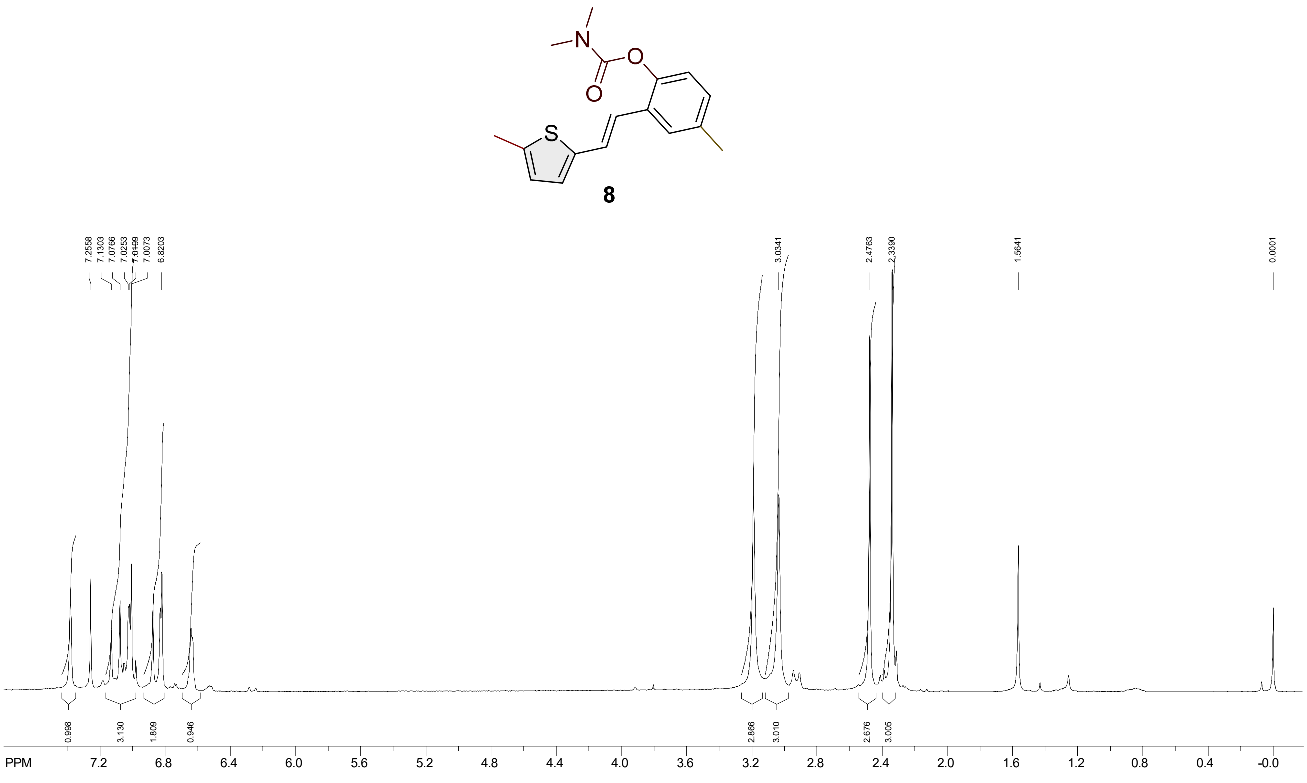

Figure S29. ^{1}H NMR spectrum ($CDCl_3$) of carbamate **8**.

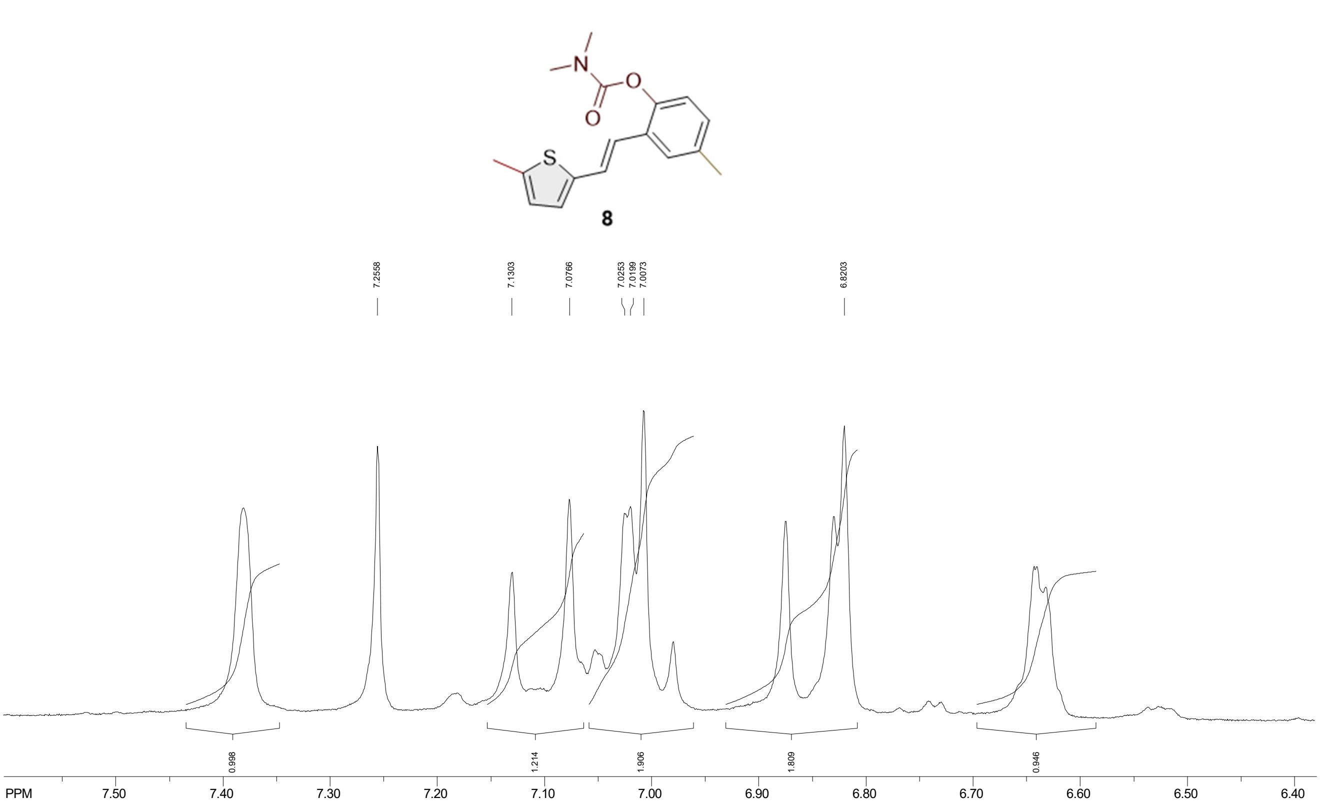

Figure S30. Aromatic part of the ^{1}H NMR spectrum ($CDCl_3$) of carbamate **8**.

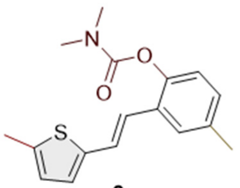

8

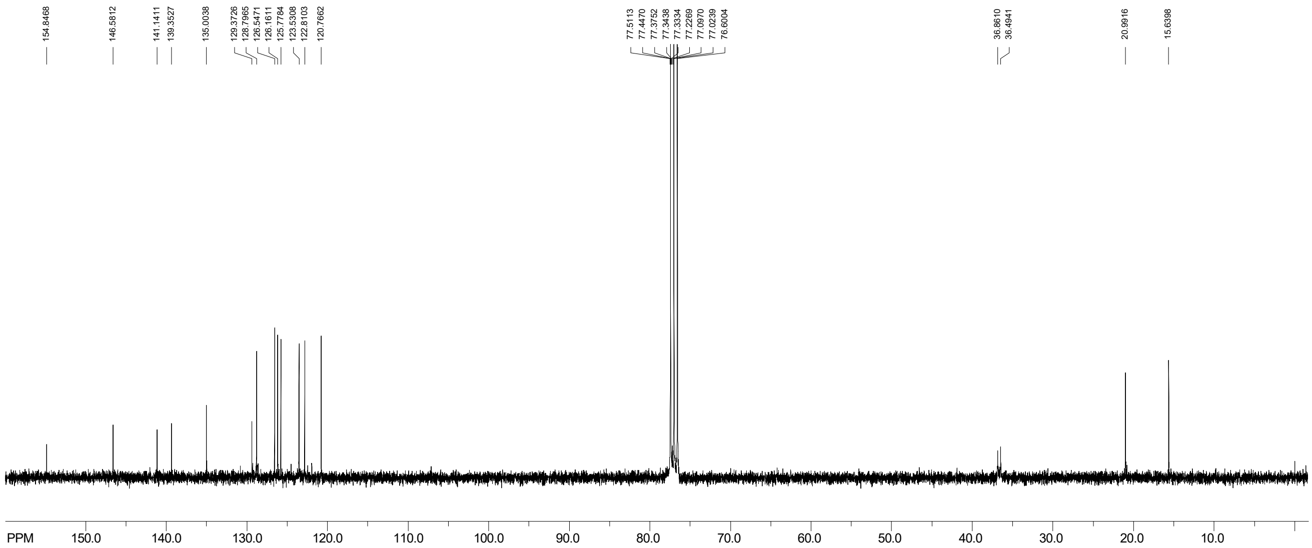

Figure S31. ^{13}C NMR spectrum ($CDCl_3$) of carbamate **8**.

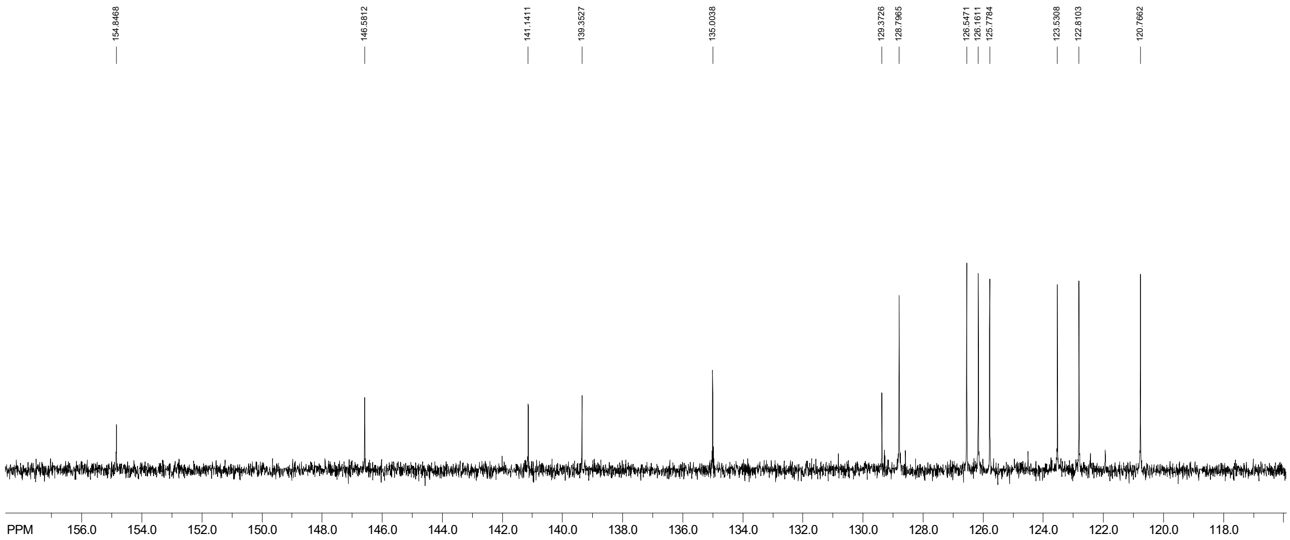

Figure S32. Aromatic part of the ^{13}C NMR spectrum ($CDCl_3$) of carbamate **8**.

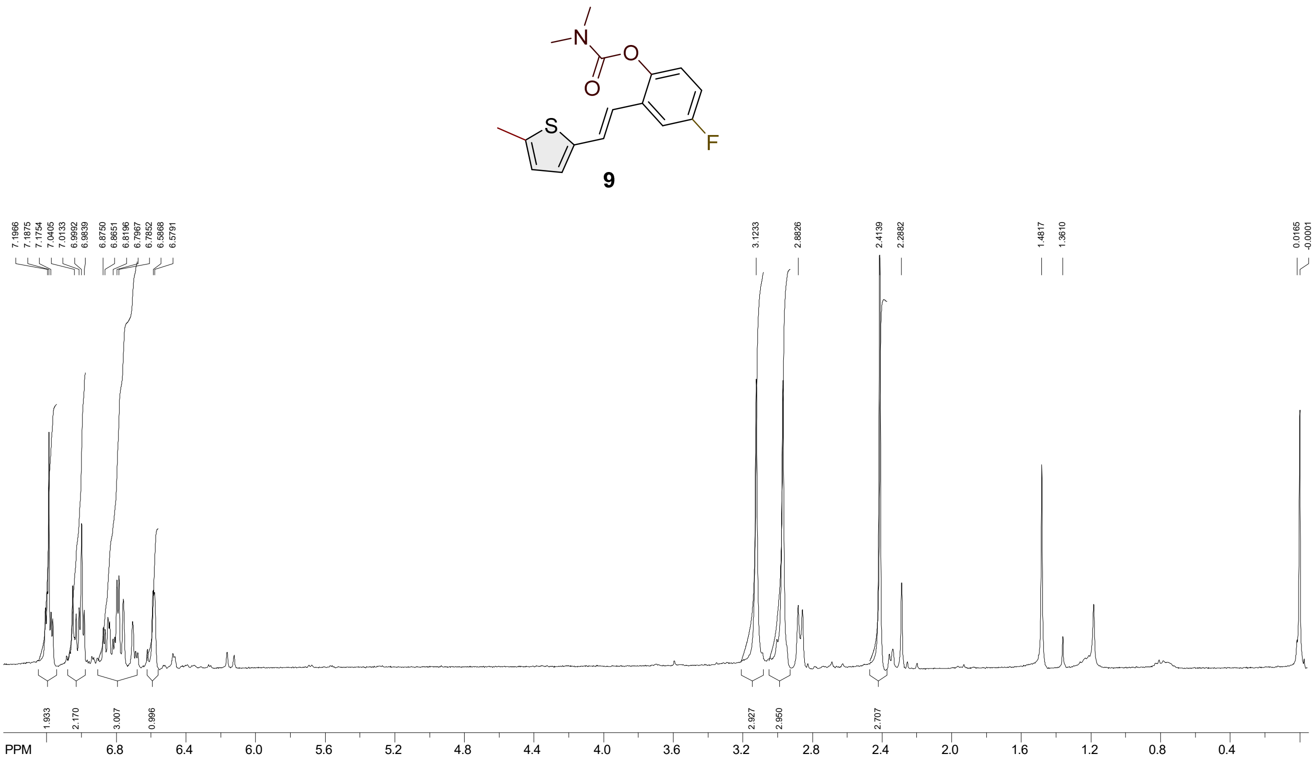

Figure S33. ^{1}H NMR spectrum ($CDCl_3$) of carbamate **9**.

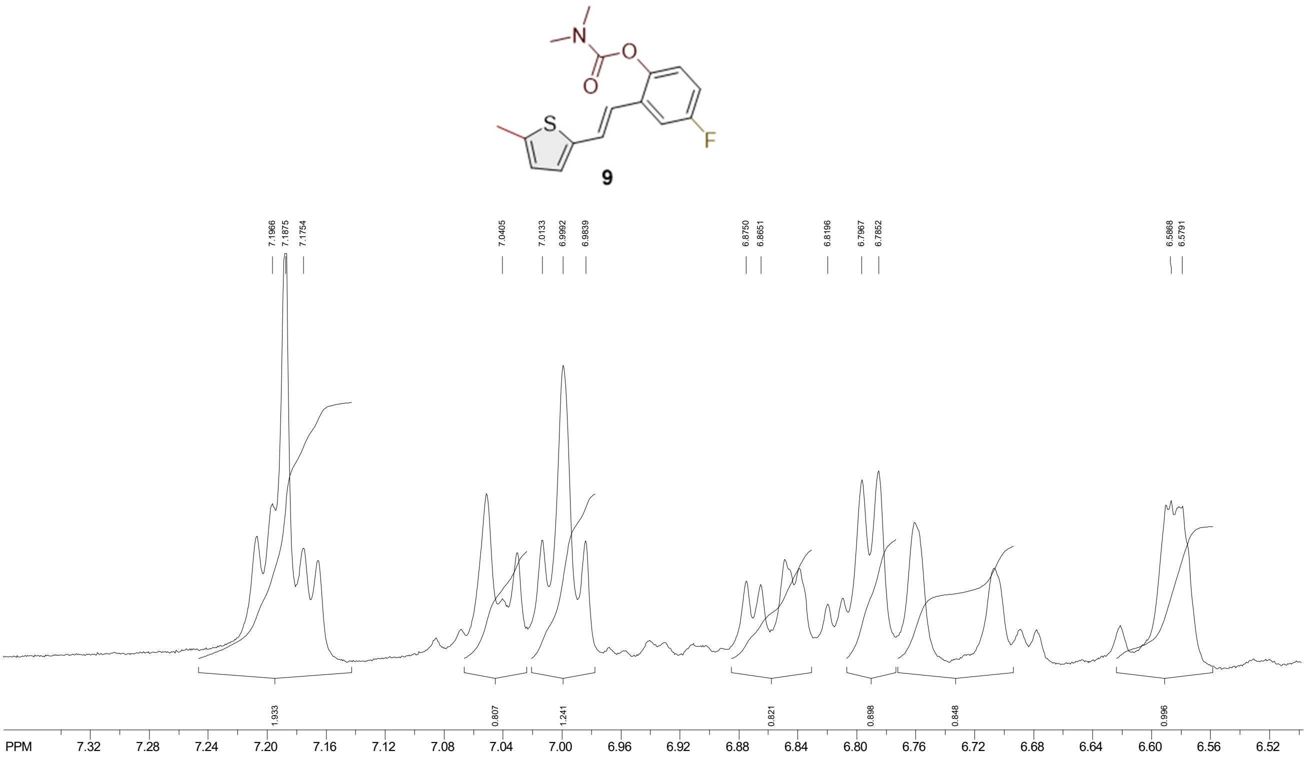

Figure S34. Aromatic part of the ^{1}H NMR spectrum ($CDCl_3$) of carbamate **9**.

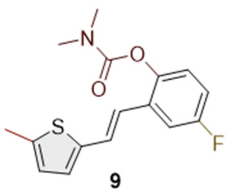

9

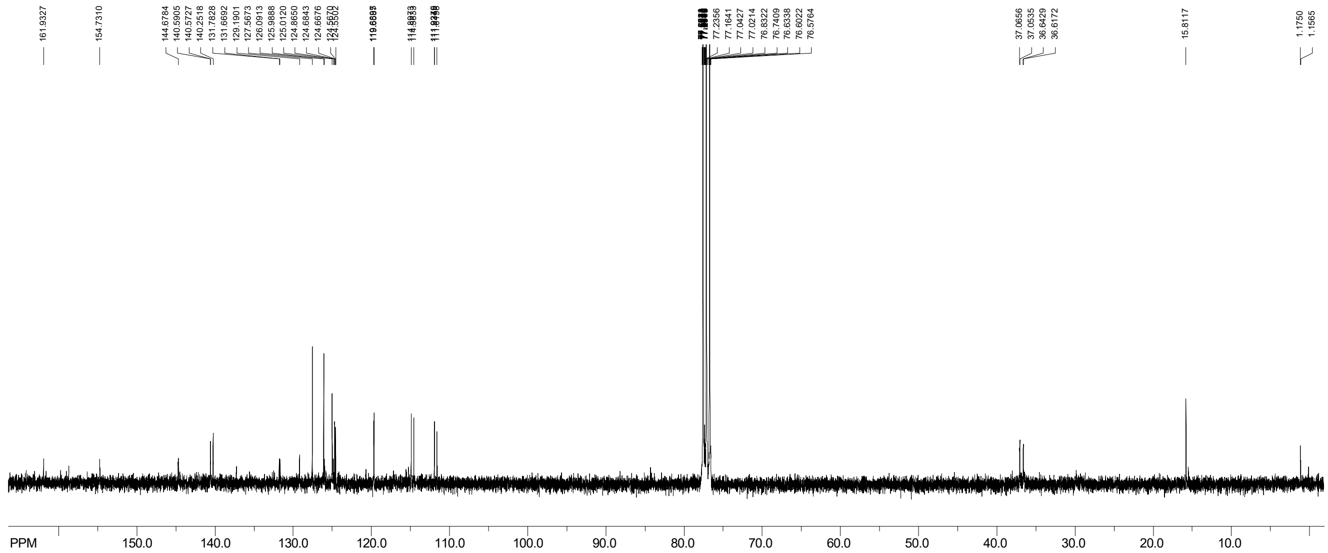

Figure S35. ^{13}C NMR spectrum ($CDCl_3$) of carbamate **9**.

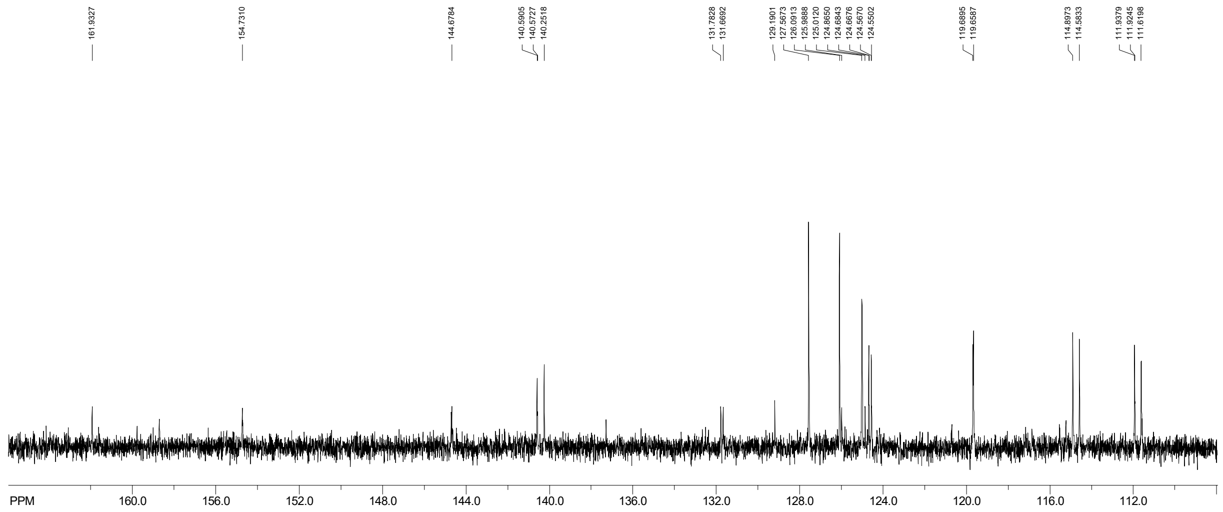

Figure S36. Aromatic part of the ^{13}C NMR spectrum ($CDCl_3$) of carbamate **9**.

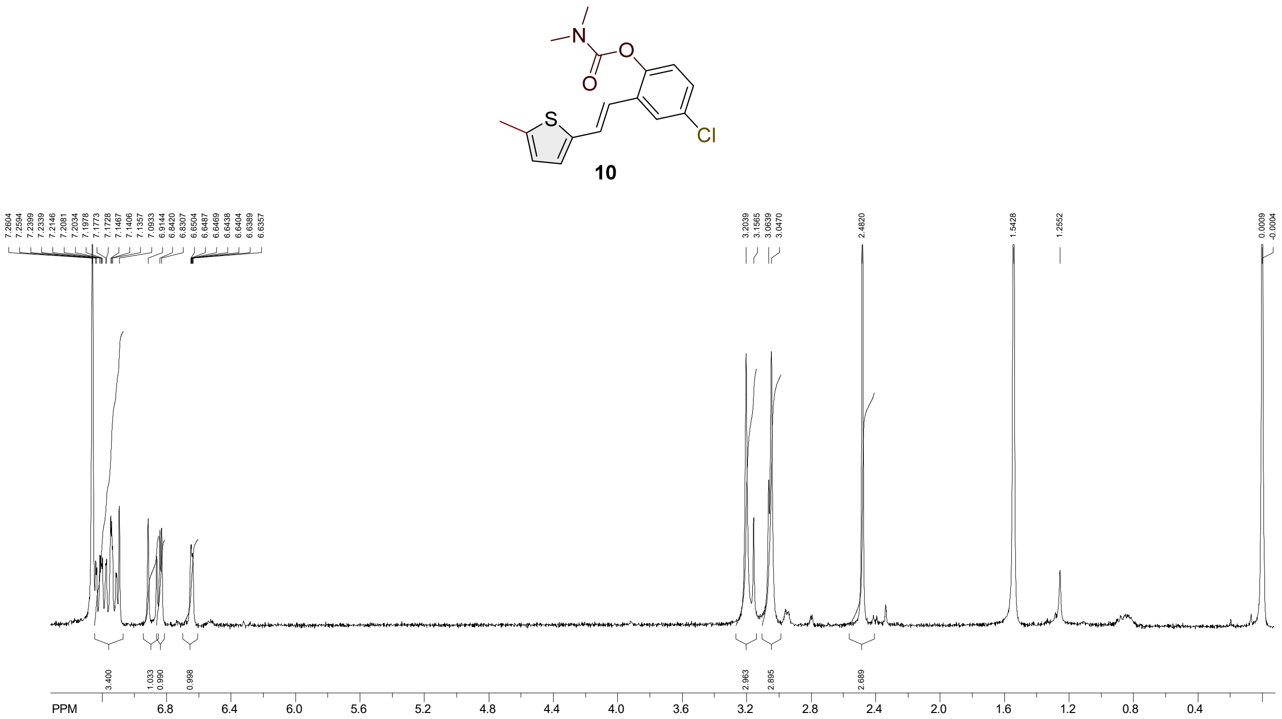

Figure S37. ^{1}H NMR spectrum ($CDCl_3$) of carbamate **10**.

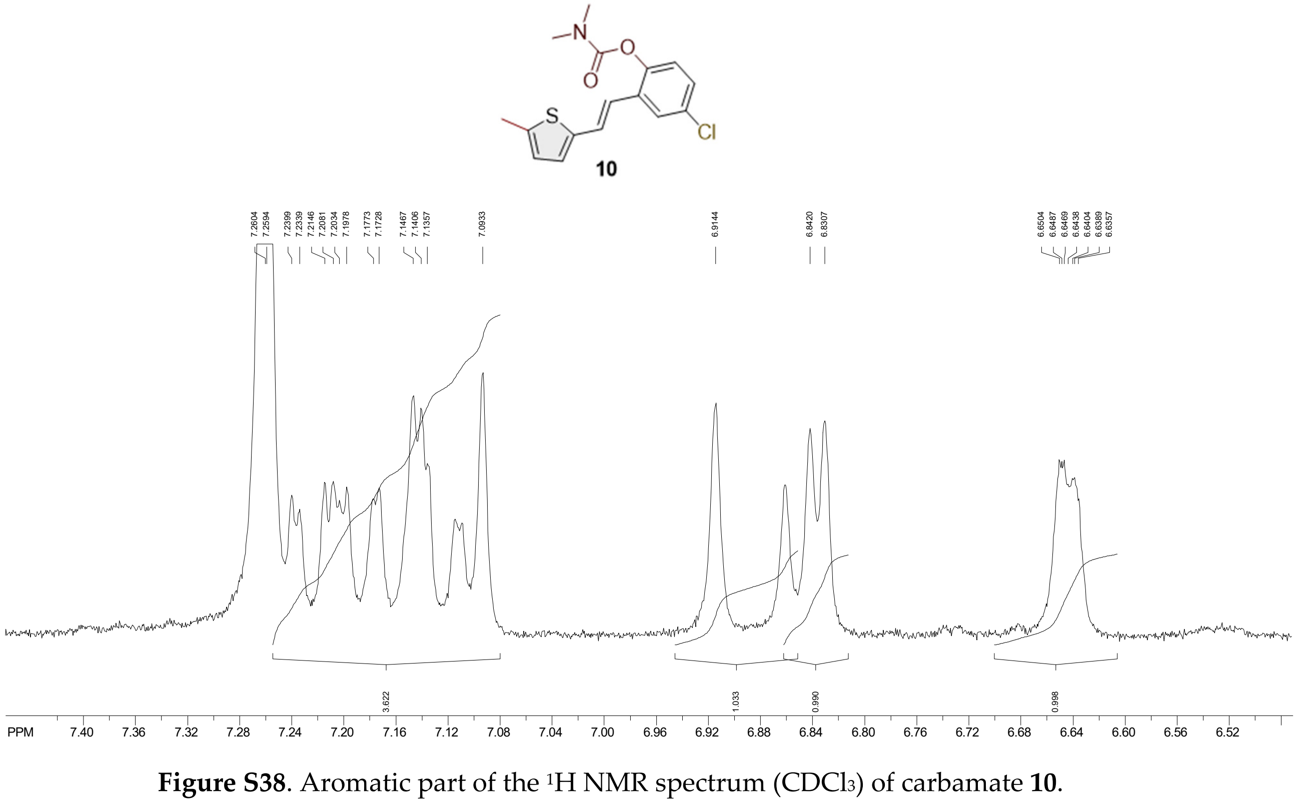

Figure S38. Aromatic part of the ^{1}H NMR spectrum ($CDCl_3$) of carbamate **10**.

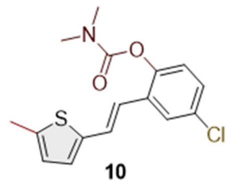

10

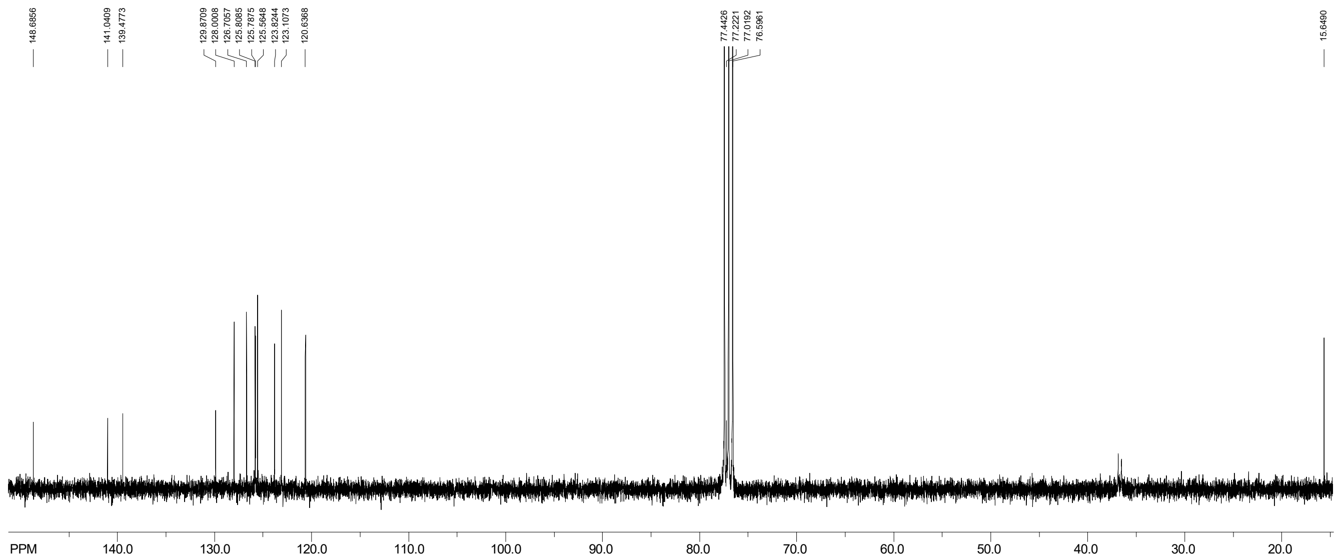

Figure S39. ^{13}C NMR spectrum ($CDCl_3$) of carbamate **10**.

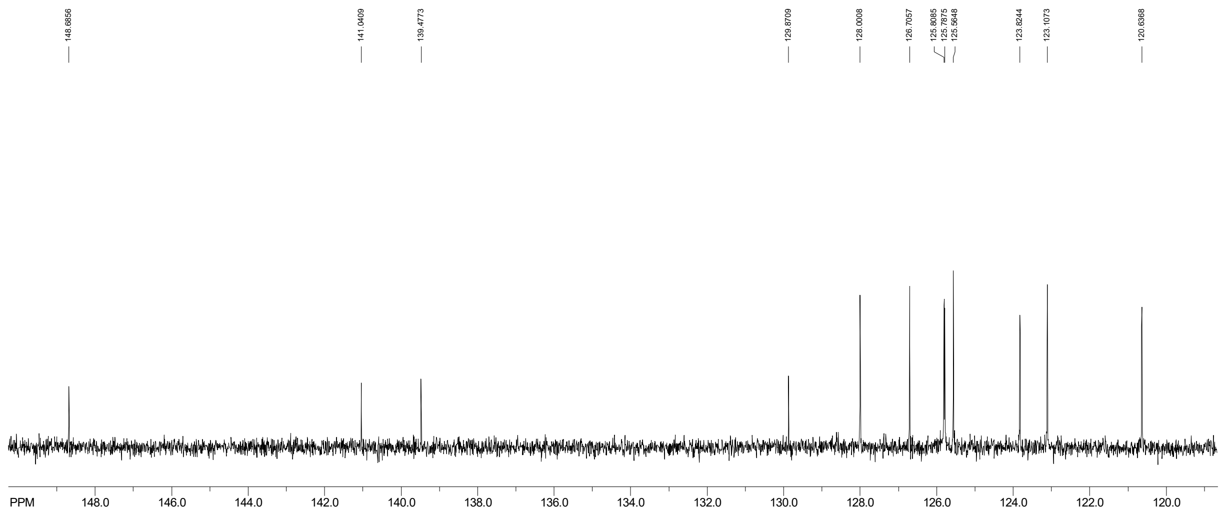

Figure S40. ^{13}C NMR spectrum ($CDCl_3$) of carbamate **10**.

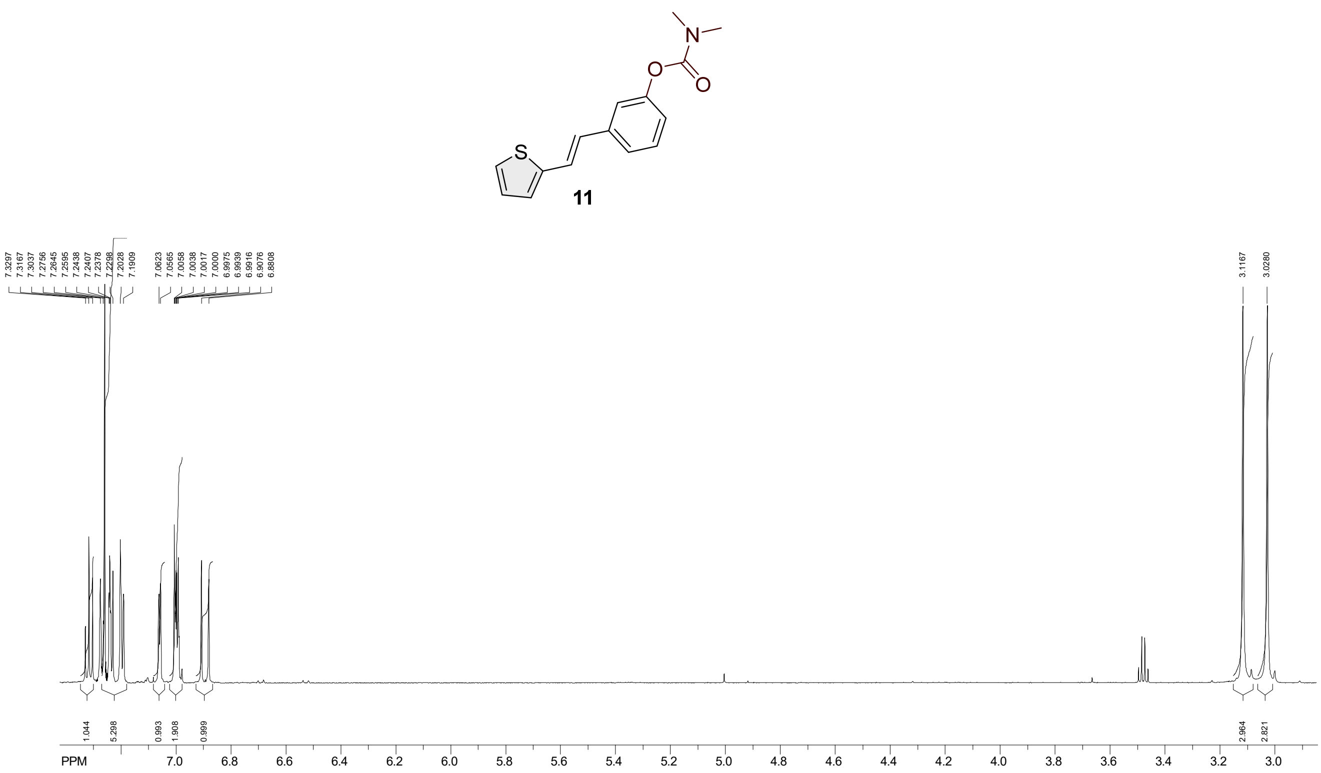

Figure S41. ^{1}H NMR spectrum ($CDCl_3$) of carbamate **11**.

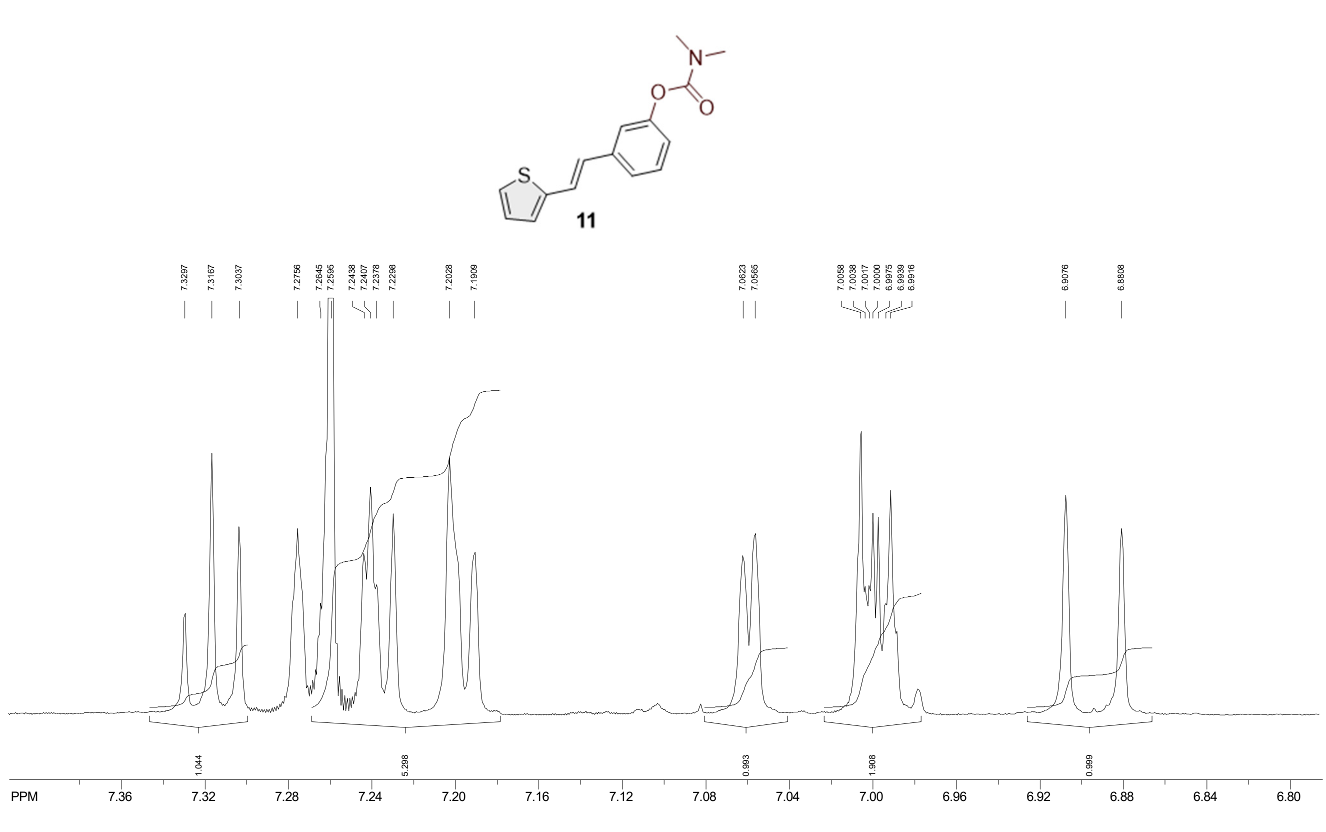

Figure S42. Aromatic part of the ^{1}H NMR spectrum ($CDCl_3$) of carbamate **11**.

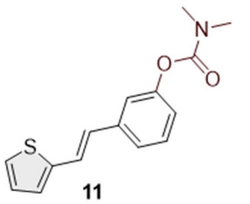

11

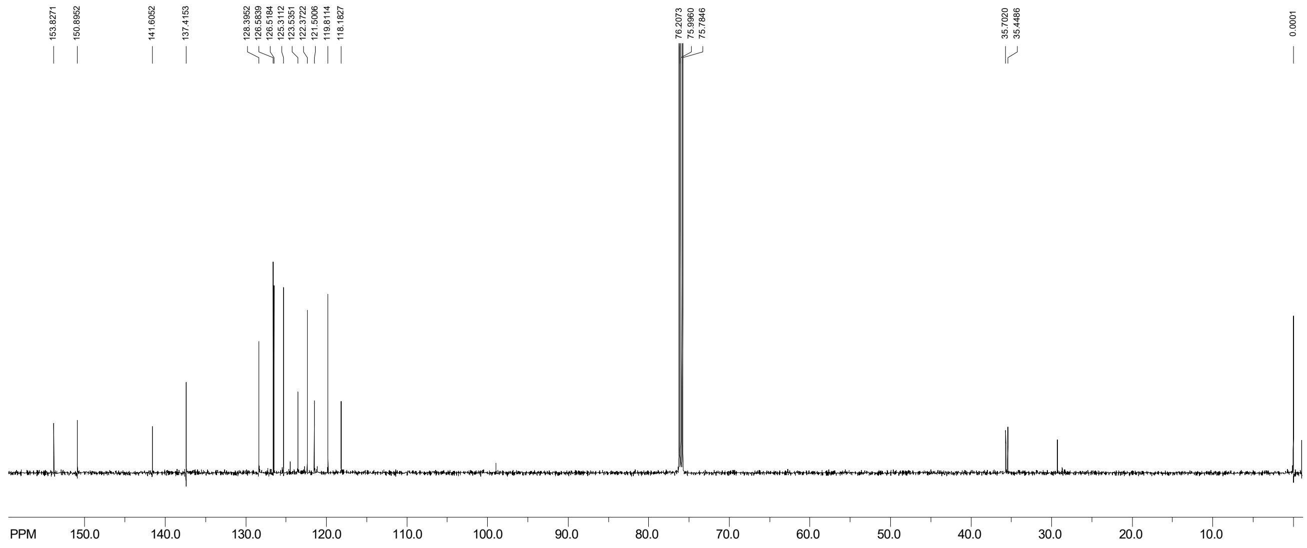

Figure S43. ^{13}C NMR spectrum ($CDCl_3$) of carbamate **11**.

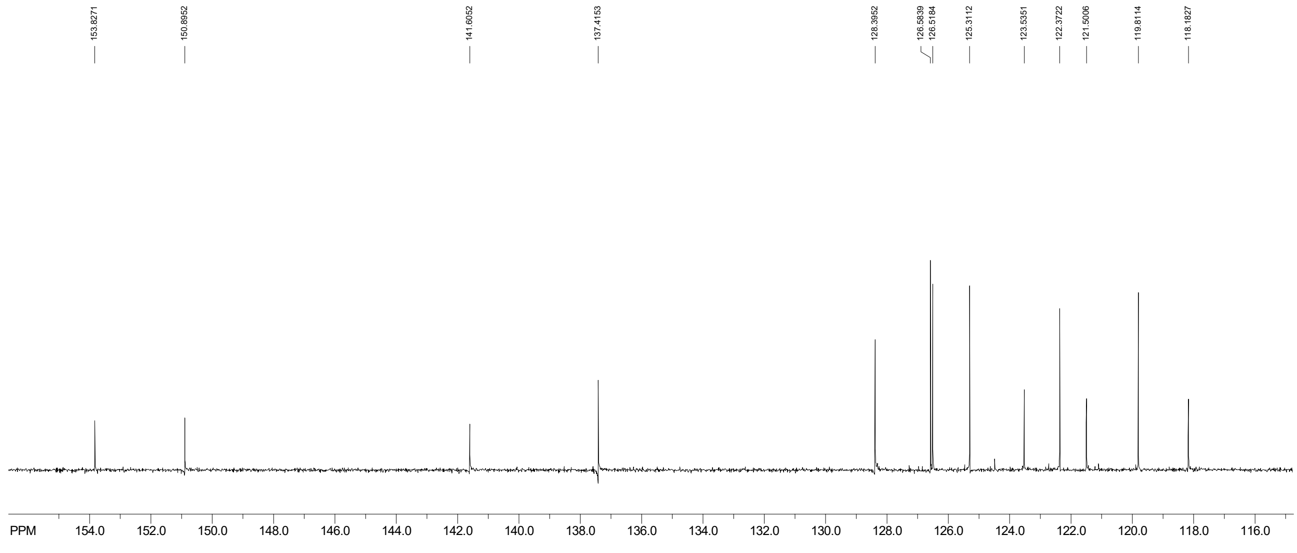

Figure S44. Aromatic part of the ^{13}C NMR spectrum ($CDCl_3$) of carbamate **11**.

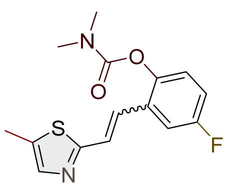

12

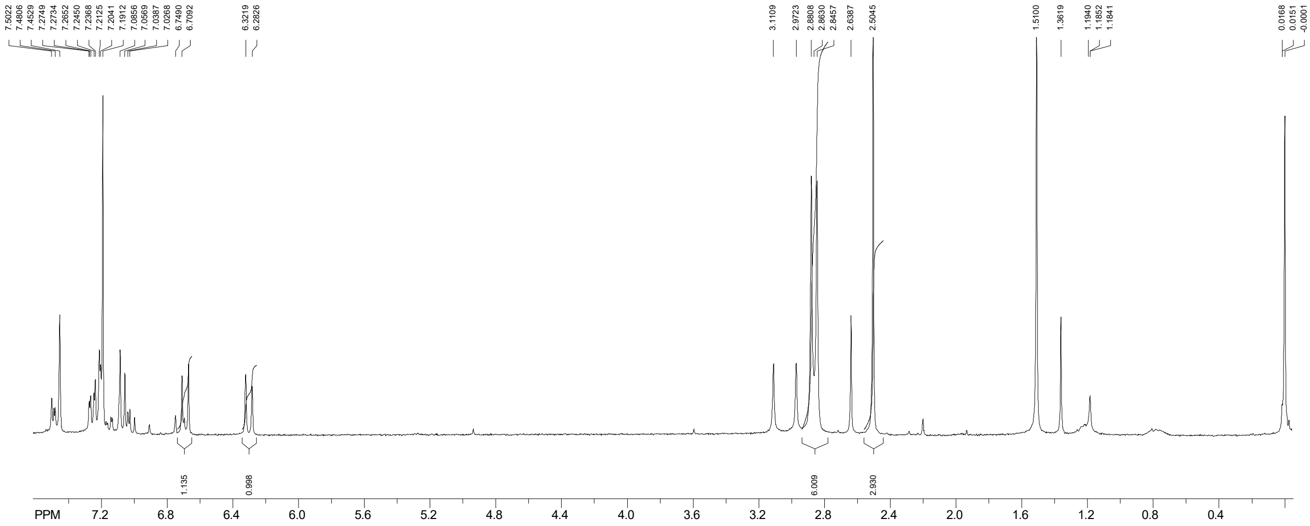

Figure S45. ^{1}H NMR spectrum ($CDCl_3$) of carbamate **12**.

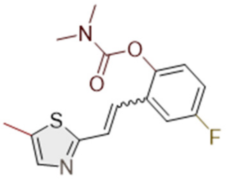

12

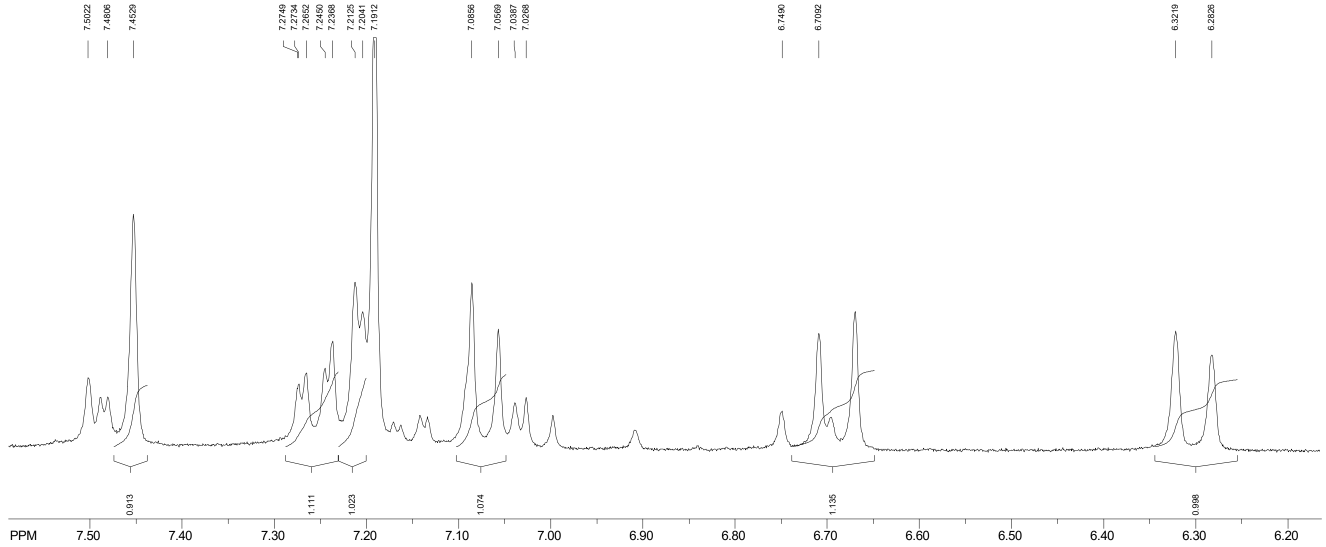

Figure S46. Aromatic part of the ^{1}H NMR spectrum ($CDCl_3$) of carbamate **12**.

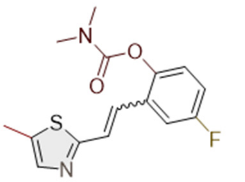

12

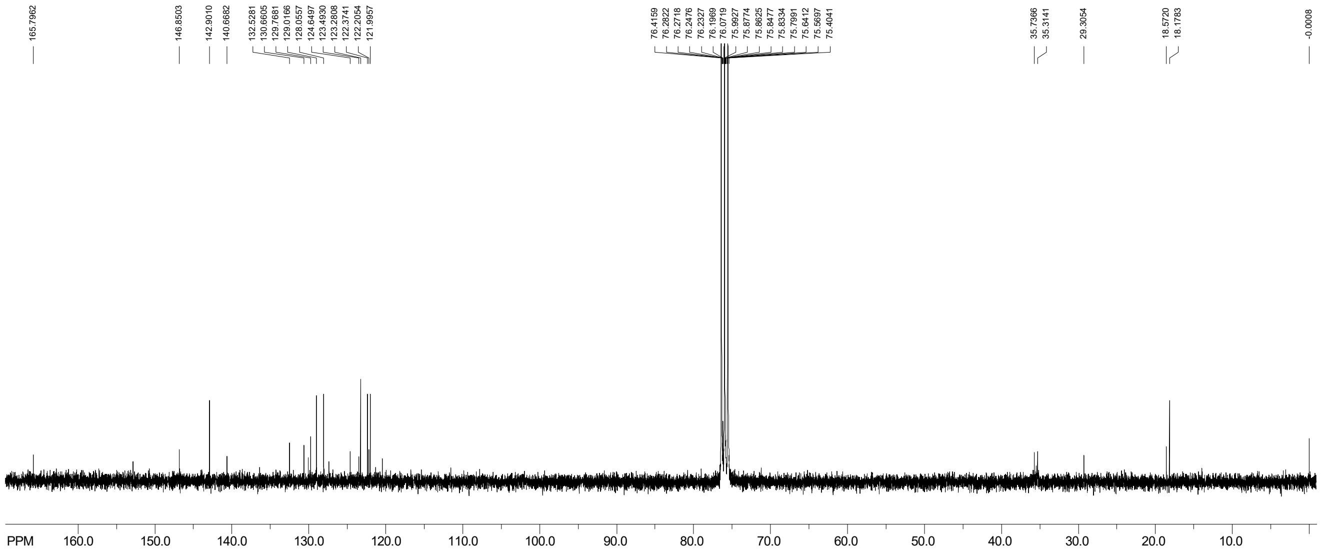

Figure S47. ^{13}C NMR spectrum ($CDCl_3$) of carbamate **12**.

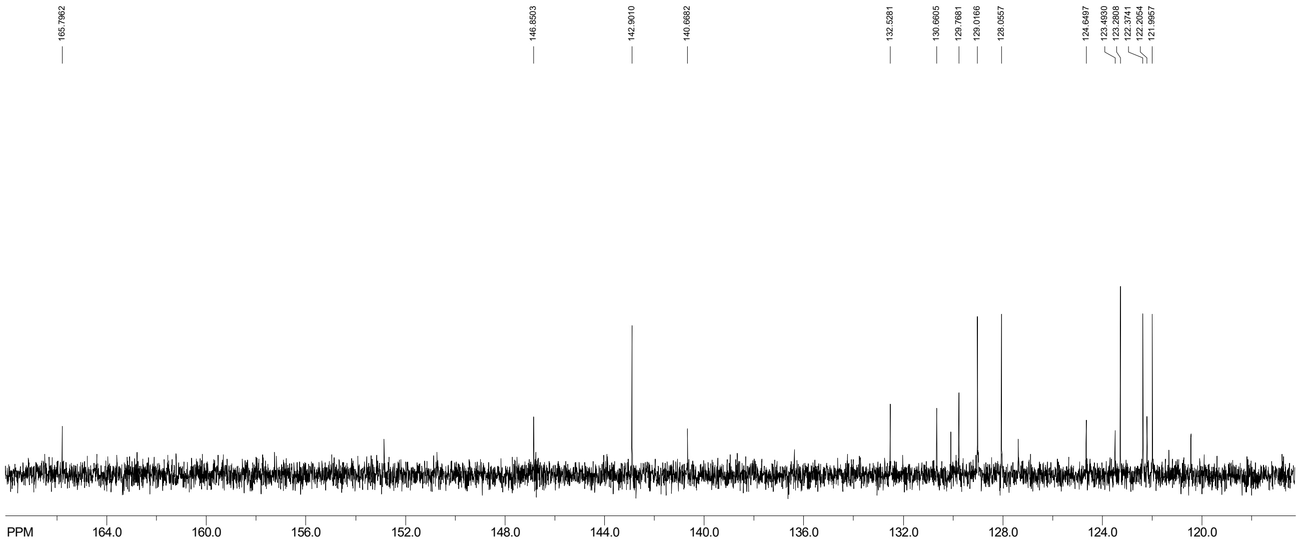

Figure S48. Aromatic part of the ^{13}C NMR spectrum ($CDCl_3$) of carbamate **12**.

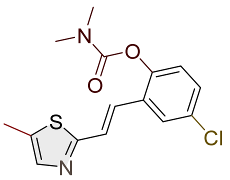

13

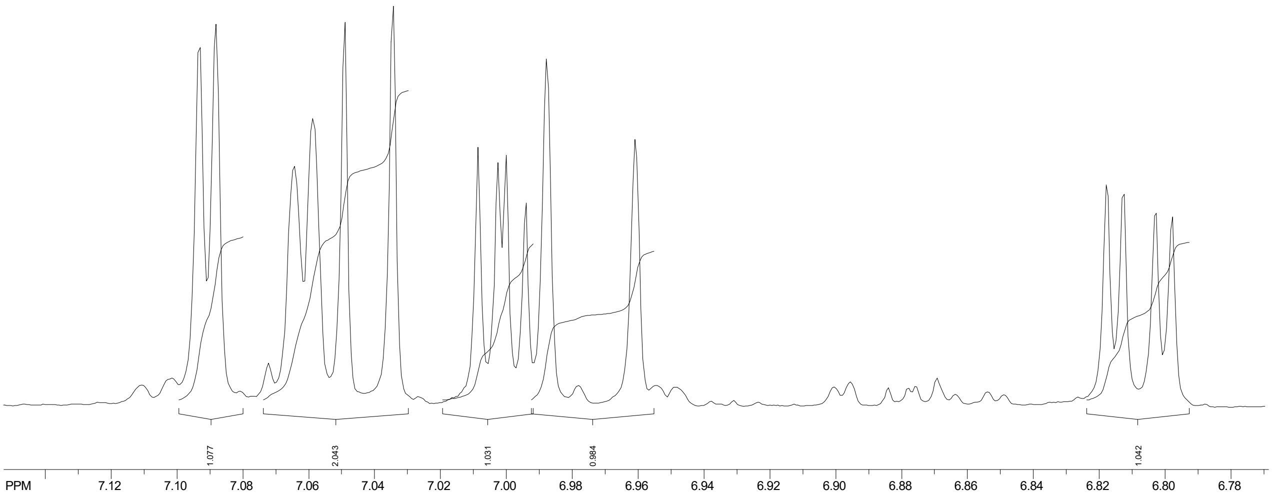

Figure S49. Aromatic part of the ^{1}H NMR spectrum ($CDCl_3$) of carbamate **13**.

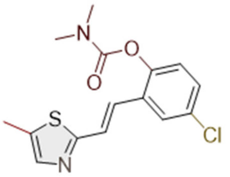

13

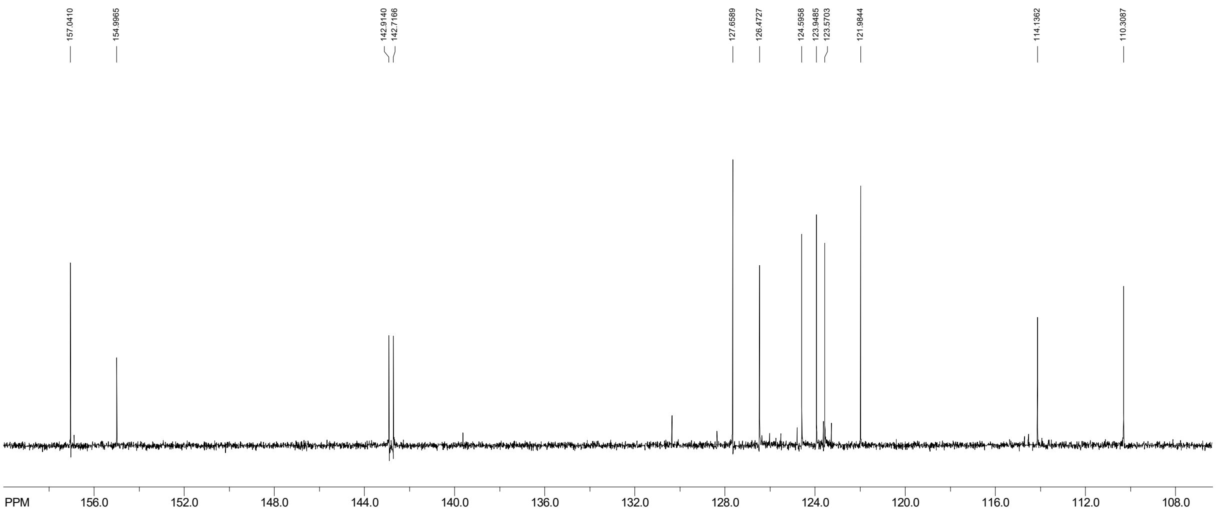

Figure S50. Aromatic part of the ^{13}C NMR spectrum ($CDCl_3$) of carbamate **13**.

2. Mass spectra and HRMS analyses of carbamates 1 – 13

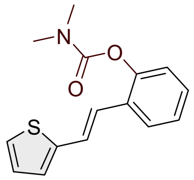

1

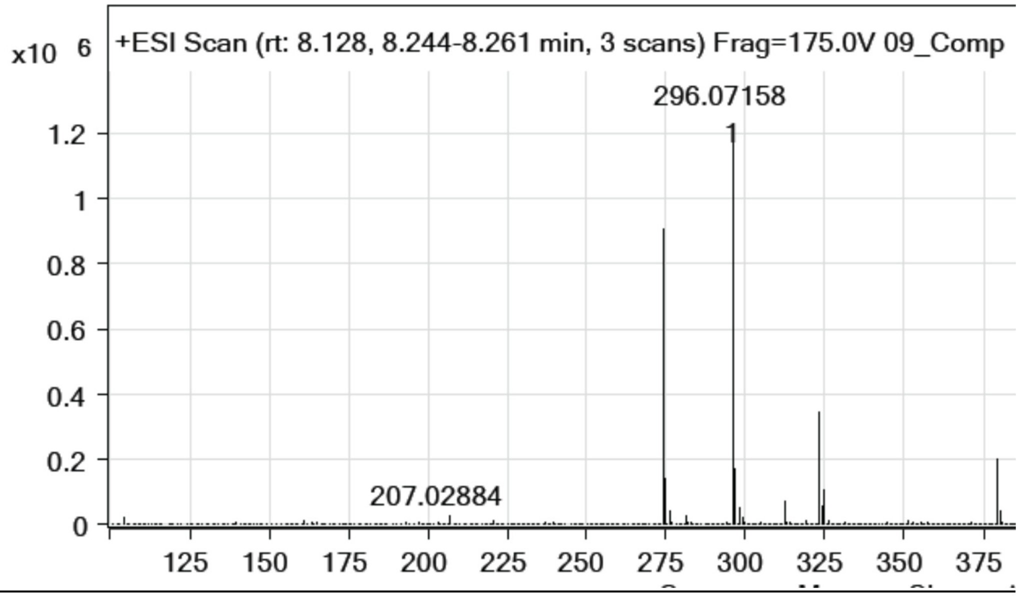

Formula Calculator Results

Formula	Best	Mass	Tgt Mass	Diff (ppm)	Ion Species	Score
C15 H15 N O2 S	True	273.08242	273.08235	-0.27	C15 H16 N O2 S	98.1

Figure S51. Mass spectra and HRMS analysis of carbamate **1**.

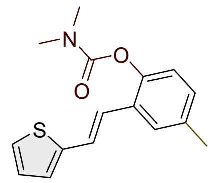

2

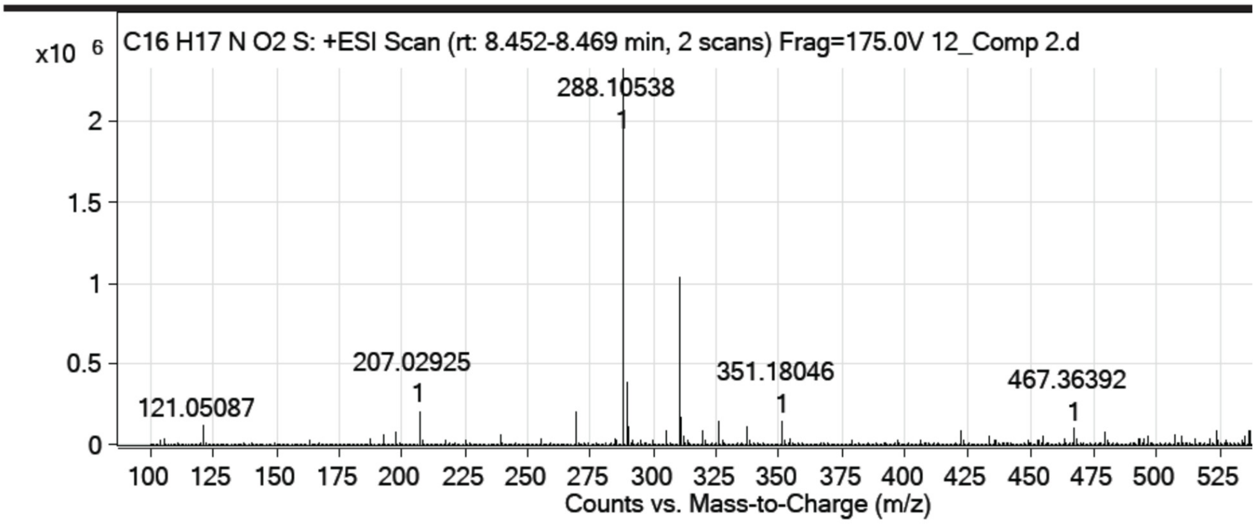

Formula Calculator Results

Formula	Best	Mass	Tgt Mass	Diff (ppm)	Ion Species	Score
C16 H17 N O2 S	True	287.0982	287.098	-0.7	C16 H18 N O2 S	97.4

Figure S52. Mass spectra and HRMS analysis of carbamate **2**.

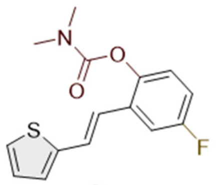

3

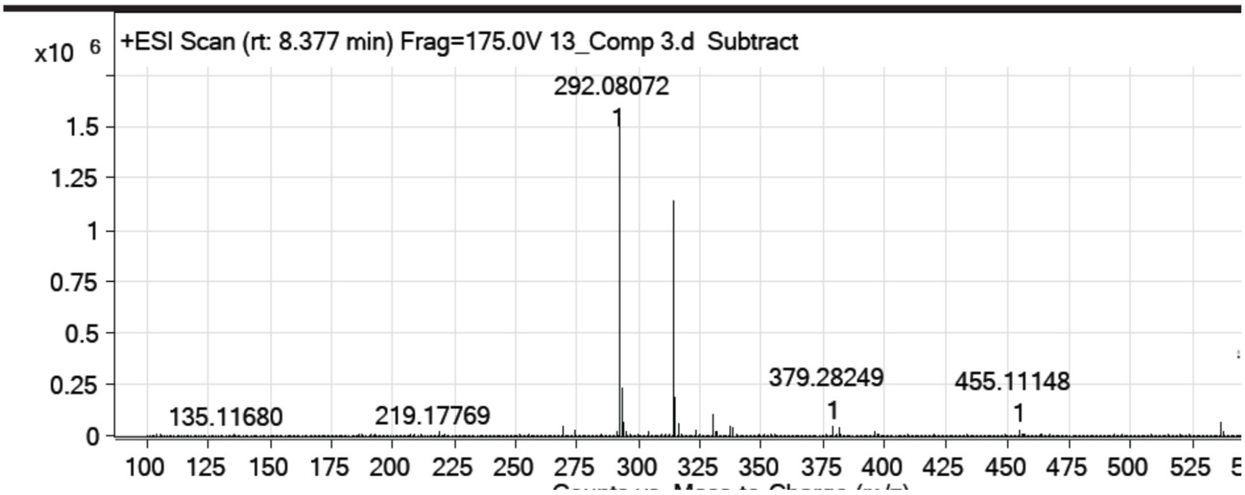

Formula Calculator Results

Formula	Best	Mass	Tgt Mass	Diff (ppm)	Ion Species	Score
C15 H14 F N O2 S	True	291.07346	291.07293	-1.83	C15 H15 F N O2 S	96.72

Figure S53. Mass spectra and HRMS analysis of carbamate **3**.

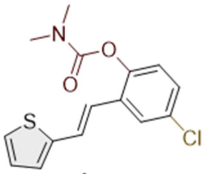

4

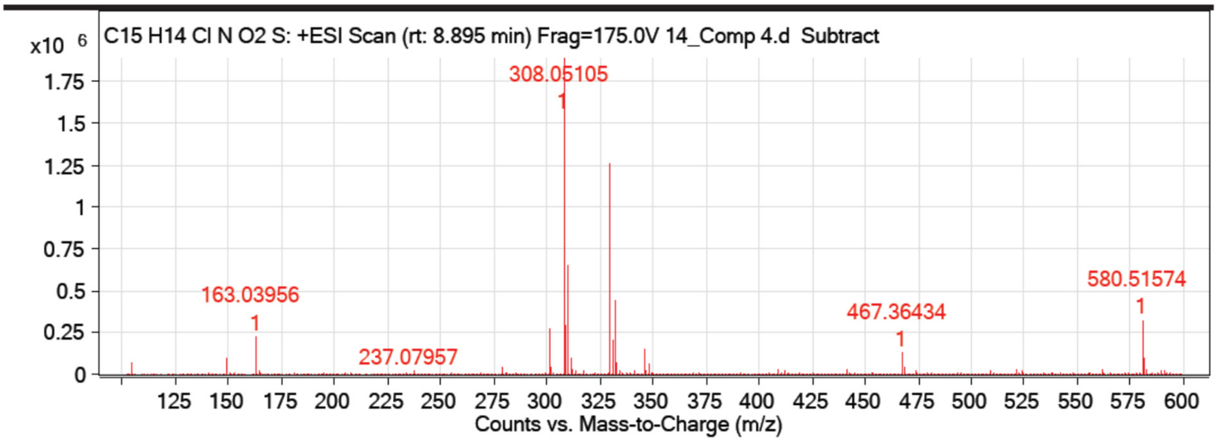

Formula Calculator Results

Formula	Best	Mass	Tgt Mass	Diff (ppm)	Ion Species	Score
C15 H14 Cl N O2 S	True	307.04388	307.04338	-1.63	C15 H15 Cl N O2 S	97.58

Figure S54. Mass spectra and HRMS analysis of carbamate **4**.

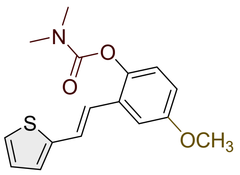

5

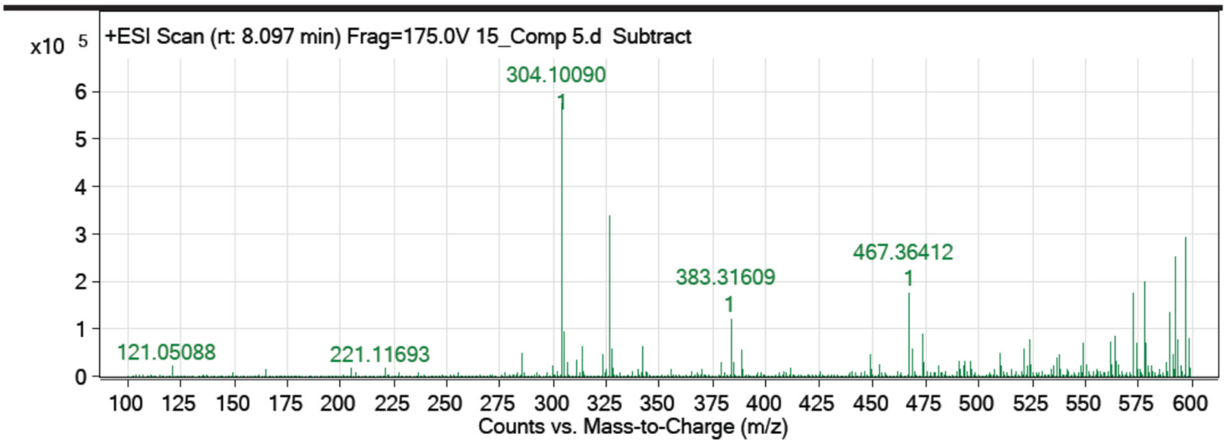

Formula Calculator Results

Formula	Best	Mass	Tgt Mass	Diff (ppm)	Ion Species	Score
C16 H17 N O3 S	True	303.09357	303.09291	-2.16	C16 H18 N O3 S	96.3

Figure S55. Mass spectra and HRMS analysis of carbamate **5**.

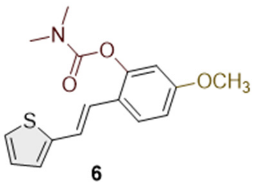

6

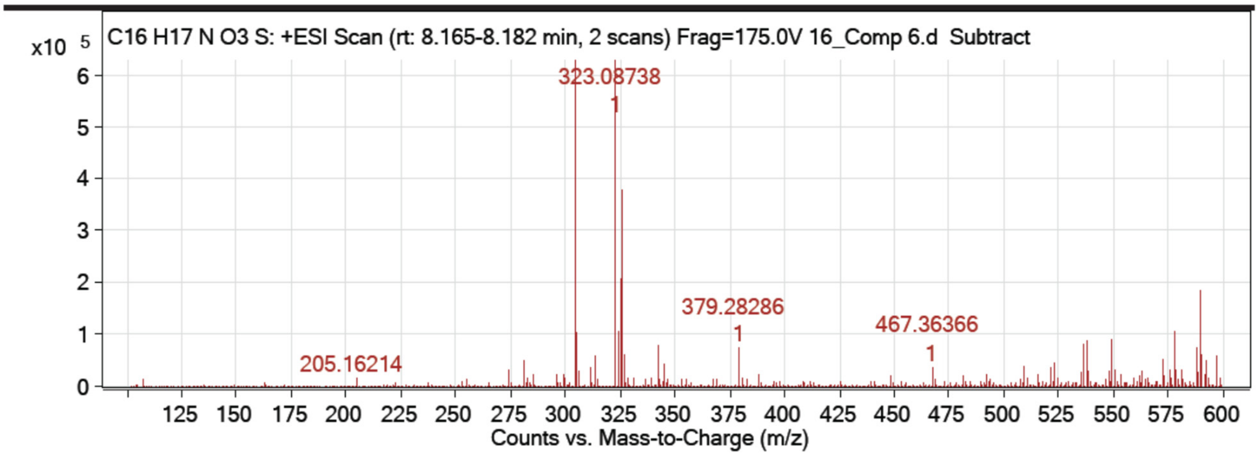

Formula Calculator Results

Formula	Best	Mass	Tgt Mass	Diff (ppm)	Ion Species	Score
C16 H17 N O3 S	True	303.09329	303.09291	-1.25	C16 H18 N O3 S	97.46

Figure S56. Mass spectra and HRMS analysis of carbamate **6**.

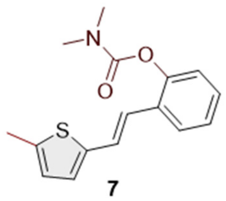

7

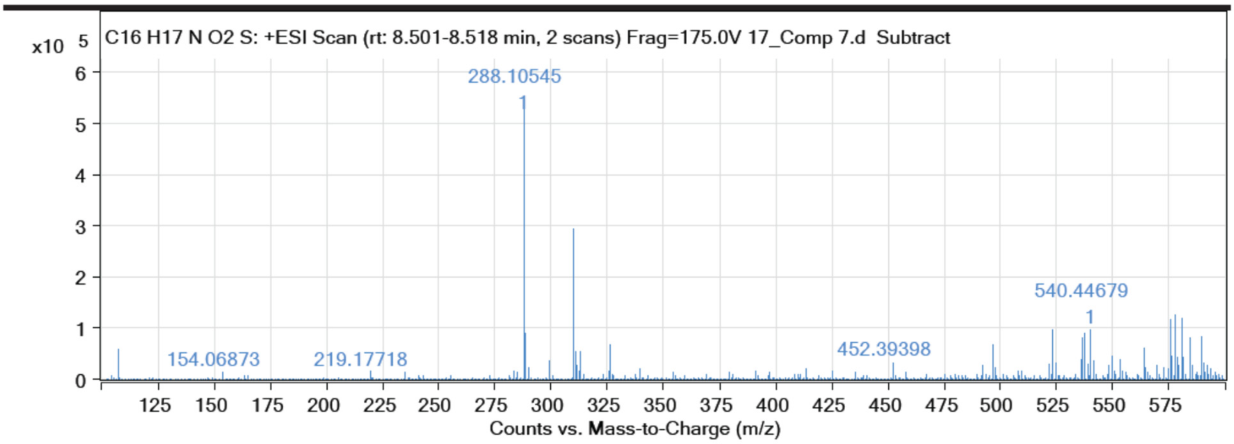

Formula Calculator Results

Formula	Best	Mass	Tgt Mass	Diff (ppm)	Ion Species	Score
C16 H17 N O2 S	True	287.09819	287.098	-0.68	C16 H18 N O2 S	97.71

Figure S57. Mass spectra and HRMS analysis of carbamate **7**.

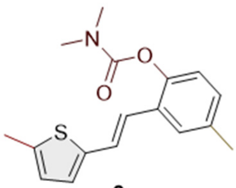

8

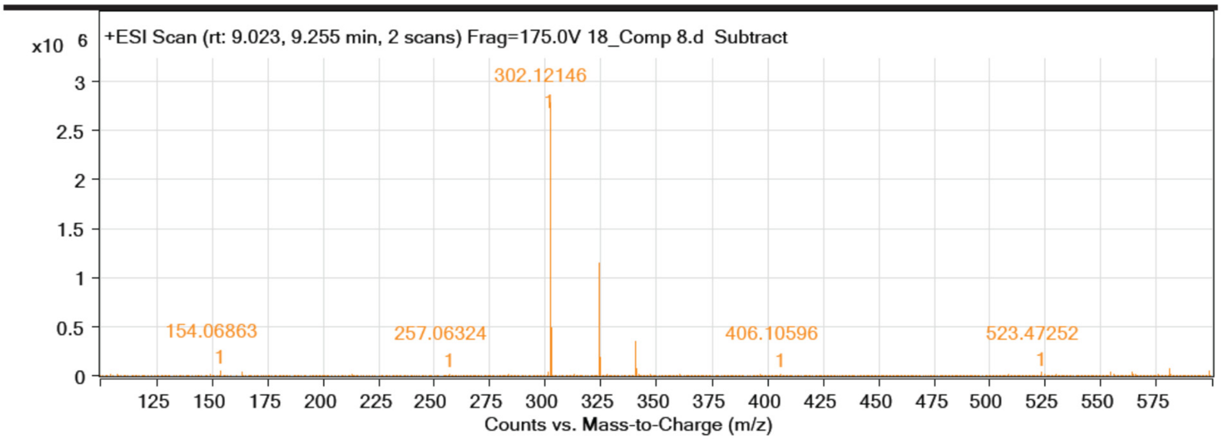

Formula Calculator Results

Formula	Best	Mass	Tgt Mass	Diff (ppm)	Ion Species	Score
C17 H19 N O2 S	True	301.11425	301.11365	-2	C17 H20 N O2 S	74.93

Figure S58. Mass spectra and HRMS analysis of carbamate **8**.

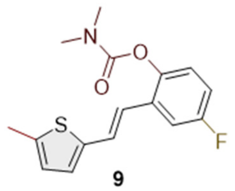

9

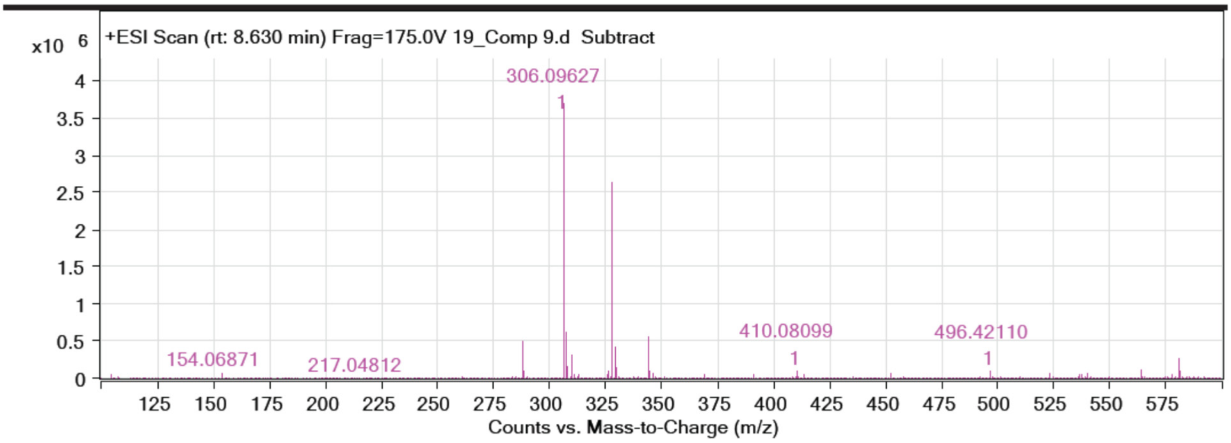

Formula Calculator Results

Formula	Best	Mass	Tgt Mass	Diff (ppm)	Ion Species	Score
C16 H16 F N O2 S	True	305.08904	305.08858	-1.51	C16 H17 F N O2 S	97.26

Figure S59. Mass spectra and HRMS analysis of carbamate **9**.

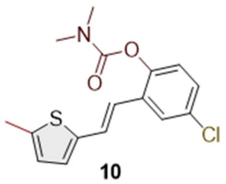

10

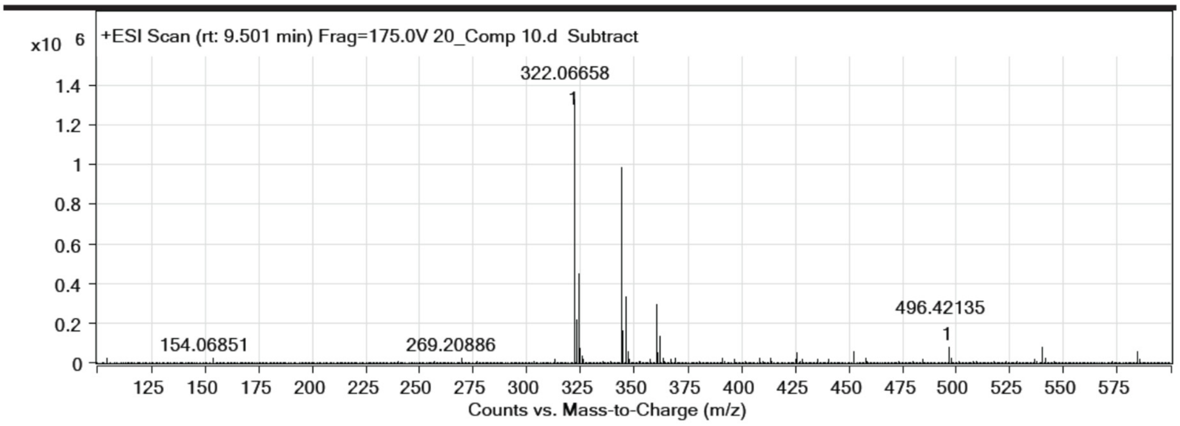

Formula Calculator Results

Formula	Best	Mass	Tgt Mass	Diff (ppm)	Ion Species	Score
C16 H16 Cl N O2 S	True	321.05942	321.05903	-1.22	C16 H17 Cl N O2 S	97.06

Figure S60. Mass spectra and HRMS analysis of carbamate **10**.

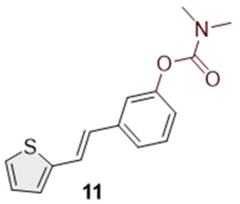

11

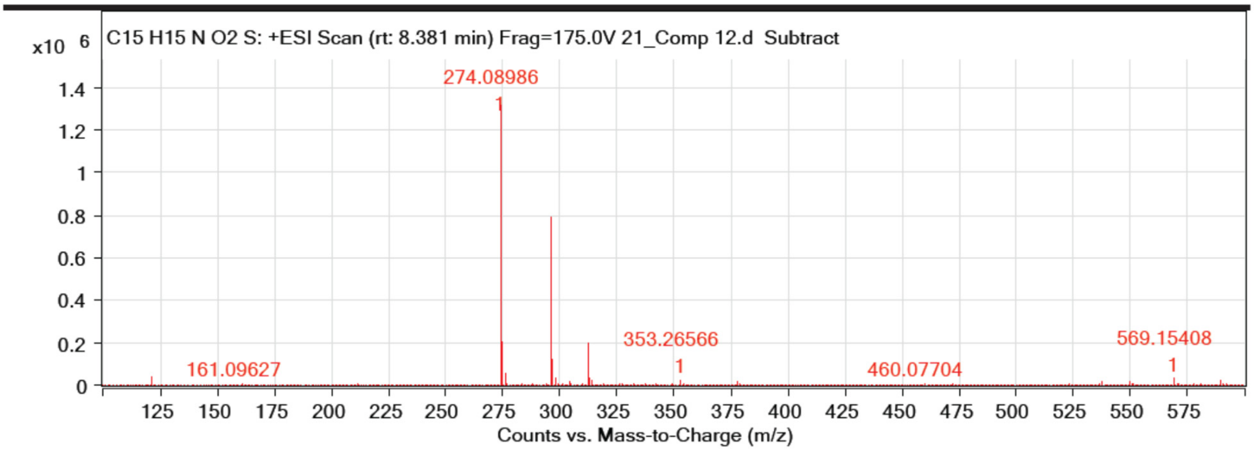

Formula Calculator Results

Formula	Best	Mass	Tgt Mass	Diff (ppm)	Ion Species	Score
C15 H15 N O2 S	True	273.08264	273.08235	-1.08	C15 H16 N O2 S	97.34

Figure S61. Mass spectra and HRMS analysis of carbamate **11**.

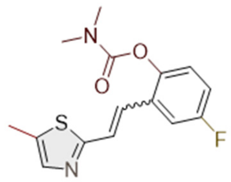

12

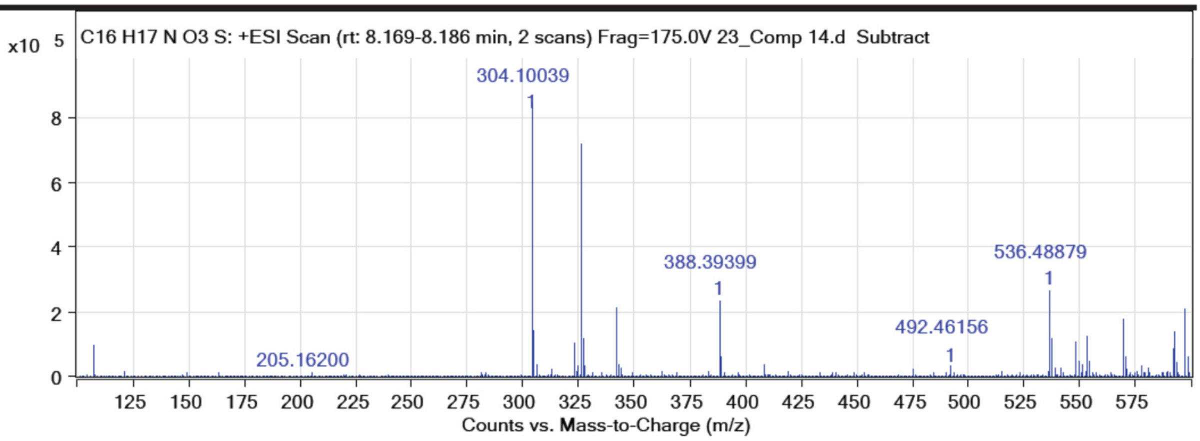

Figure S62. Mass spectra of carbamate **12**.

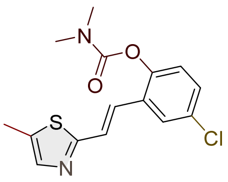

13

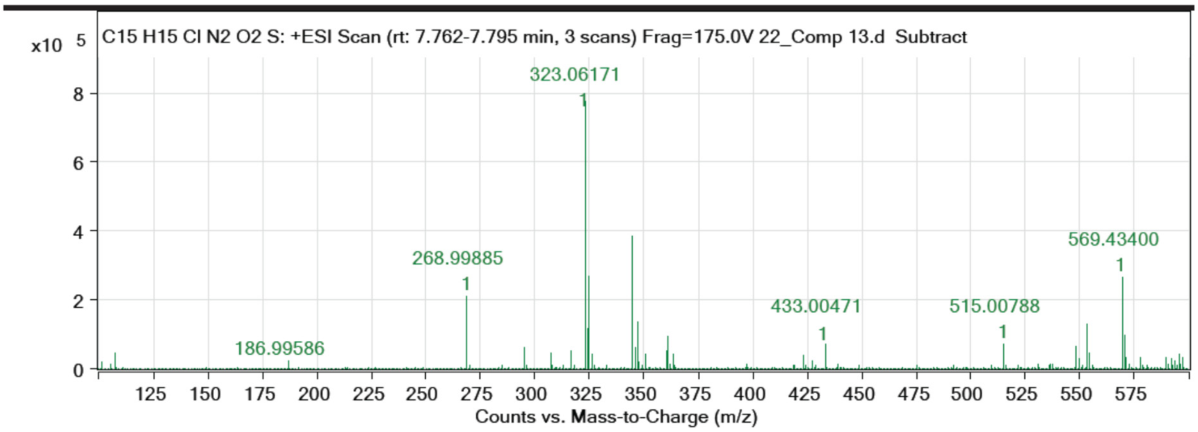

Formula Calculator Results

Formula	Best	Mass	Tgt Mass	Diff (ppm)	Ion Species	Score
C15 H15 Cl N2 O2 S	True	322.05451	322.05428	-0.72	C15 H16 Cl N2 O2 S	98.27

Figure S63. Mass spectra and HRMS analysis of carbamate **13**.

3. Table S1, free energies of binding obtained by docking

Table S1. Free energies of binding, ΔG_{bind} obtained by molecular docking of listed molecules into the active site of BChE (3DJY.pdb), along with the number of conformational clusters and distribution of conformations.

Ligand	ΔG_{bind}/kcal mol^{-1}		Number of distinctive conformational clusters	Distribution of conformations within clusters with $n > 1$ (n = cluster population)	Experiment IC$_{50}$/μM
	lowest	highest			
**1**	−6.85	−6.12	3	4, 11, 5	0.12 ± 0.09
**7**	−7.43	−6.52	4	17, 3, 4	0.38 ± 0.01
Galantamine	−7.31	−7.26	1	25	7.54 ± 1.64

4. Cartesian coordinates of docked ligands and enzyme

Ligand **1**, docked conformation **1a**

C	8.934000	-4.182000	-6.441000
H	8.613000	-3.133000	-6.330000
C	7.976000	-5.169000	-6.713000
C	8.433000	-6.486000	-6.865000
C	9.775000	-6.824000	-6.733000
H	10.102000	-7.871000	-6.849000
C	10.700000	-5.823000	-6.452000
H	11.768000	-6.076000	-6.343000
C	10.279000	-4.499000	-6.308000
H	11.014000	-3.706000	-6.089000
C	6.544000	-4.874000	-6.846000
H	5.859000	-5.728000	-6.984000
C	6.003000	-3.644000	-6.813000
H	6.691000	-2.795000	-6.660000
C	4.591000	-3.336000	-6.961000
S	3.411000	-4.512000	-7.459000
C	2.133000	-3.359000	-7.355000
H	1.077000	-3.585000	-7.580000
C	2.584000	-2.133000	-6.961000
H	1.933000	-1.252000	-6.831000
C	3.991000	-2.120000	-6.737000
H	4.545000	-1.222000	-6.415000
O	7.457000	-7.450000	-7.060000
C	7.165000	-7.878000	-8.329000
O	7.917000	-7.757000	-9.271000
N	5.938000	-8.462000	-8.363000
C	5.404000	-8.857000	-9.654000
H	6.128000	-8.891000	-10.501000
H	4.544000	-8.200000	-9.922000
H	4.887000	-9.841000	-9.562000
C	4.993000	-8.417000	-7.258000
H	5.403000	-8.114000	-6.267000
H	4.476000	-9.401000	-7.166000
H	4.133000	-7.760000	-7.526000

Ligand **1**, docked conformation **1b**

C	1.974000	-3.152000	-7.966000
C	1.551000	-4.272000	-7.234000
C	1.396000	-4.115000	-5.849000
C	1.624000	-2.901000	-5.211000
C	2.033000	-1.806000	-5.966000
C	2.210000	-1.933000	-7.345000
C	1.278000	-5.573000	-7.857000
C	1.540000	-5.886000	-9.137000
C	1.291000	-7.175000	-9.761000
S	2.357000	-8.537000	-9.580000
C	1.348000	-9.530000	-10.566000
C	0.251000	-8.854000	-11.015000
C	0.219000	-7.506000	-10.555000
O	0.907000	-5.216000	-5.164000

C	1.345000	-5.478000	-3.892000
O	1.424000	-4.639000	-3.021000
N	1.653000	-6.794000	-3.756000
C	2.261000	-7.231000	-2.512000
C	1.725000	-7.725000	-4.872000

Ligand **7**, docked

C	8.961000	-4.365000	-6.368000
C	7.985000	-5.296000	-6.757000
C	8.399000	-6.624000	-6.934000
C	9.713000	-7.027000	-6.717000
C	10.653000	-6.081000	-6.323000
C	10.277000	-4.749000	-6.149000
C	6.582000	-4.932000	-6.985000
C	6.026000	-3.754000	-6.655000
C	4.645000	-3.373000	-6.893000
C	4.096000	-2.128000	-6.718000
C	2.706000	-2.074000	-7.032000
C	2.199000	-3.273000	-7.446000
S	3.435000	-4.493000	-7.456000
C	0.796000	-3.596000	-7.857000
O	7.402000	-7.536000	-7.245000
C	7.260000	-7.976000	-8.535000
O	8.143000	-7.933000	-9.363000
N	6.010000	-8.473000	-8.732000
C	5.635000	-8.869000	-10.078000
C	4.925000	-8.330000	-7.775000

Cartesian coordinates of BChE used for docking

N	16.801000	26.757000	-18.796000
C	17.444000	26.380000	-17.498000
C	18.808000	27.060000	-17.357000
C	19.689000	26.386000	-16.314000
O	19.784000	25.134000	-16.332000
O	20.287000	27.106000	-15.485000
C	16.573000	26.765000	-16.299000
O	16.268000	27.957000	-16.108000
N	16.179000	25.776000	-15.485000
C	15.237000	26.049000	-14.382000
C	13.937000	25.280000	-14.564000
C	12.732000	26.155000	-14.115000
C	13.862000	24.739000	-15.998000
C	15.082000	23.847000	-16.400000
C	15.776000	25.738000	-12.992000
O	15.995000	24.579000	-12.653000
N	15.971000	26.777000	-12.190000
C	16.472000	26.608000	-10.847000
C	17.821000	27.327000	-10.629000
C	18.110000	27.509000	-9.154000
C	18.963000	26.509000	-11.212000
C	19.342000	26.894000	-12.615000

C	15.433000	27.112000	-9.870000
O	14.884000	28.199000	-10.021000
N	15.130000	26.296000	-8.877000
C	14.149000	26.683000	-7.898000
C	13.009000	25.663000	-7.813000
C	12.093000	25.990000	-6.660000
C	12.210000	25.683000	-9.105000
C	11.436000	26.984000	-9.309000
C	14.876000	26.751000	-6.592000
O	15.712000	25.907000	-6.312000
N	14.590000	27.774000	-5.804000
C	15.201000	27.892000	-4.497000
C	15.452000	29.363000	-4.170000
C	14.326000	27.239000	-3.417000
O	13.192000	27.660000	-3.208000
N	14.856000	26.221000	-2.736000
C	14.125000	25.556000	-1.653000
C	14.076000	24.017000	-1.827000
C	13.993000	23.655000	-3.298000
O	15.235000	23.406000	-1.229000
C	14.754000	25.879000	-0.305000
O	15.869000	26.384000	-0.239000
N	14.063000	25.560000	0.781000
C	14.580000	25.906000	2.093000
C	13.527000	25.627000	3.168000
C	12.146000	26.149000	2.792000
C	11.303000	26.473000	4.006000
C	11.244000	27.967000	4.211000
N	10.922000	28.636000	2.926000
C	15.908000	25.210000	2.426000
O	16.606000	25.629000	3.351000
N	16.271000	24.158000	1.690000
C	17.531000	23.458000	1.959000
C	17.307000	21.950000	2.109000
C	16.288000	21.617000	3.197000
O	16.662000	21.320000	4.339000
N	14.990000	21.677000	2.848000
C	18.599000	23.726000	0.907000
O	19.731000	23.274000	1.035000
N	18.239000	24.479000	-0.121000
C	19.172000	24.819000	-1.172000
C	18.450000	24.898000	-2.499000
O	17.222000	24.726000	-2.564000
N	19.220000	25.145000	-3.552000
C	18.707000	25.288000	-4.892000
C	19.595000	26.238000	-5.683000
C	19.512000	27.681000	-5.283000
C	20.271000	28.514000	-6.320000
C	20.019000	30.021000	-6.168000
N	20.614000	30.782000	-7.330000
C	18.699000	23.952000	-5.613000
O	19.548000	23.081000	-5.360000
N	17.764000	23.789000	-6.538000
C	17.771000	22.588000	-7.329000
C	16.698000	21.563000	-6.830000
C	16.837000	21.351000	-5.349000
C	15.318000	22.054000	-7.147000
C	17.552000	22.936000	-8.773000

O	16.790000	23.841000	-9.085000
N	18.218000	22.211000	-9.664000
C	17.994000	22.368000	-11.095000
C	19.346000	22.553000	-11.805000
C	19.322000	22.259000	-13.293000
C	20.731000	22.276000	-13.883000
N	21.182000	23.639000	-14.156000
C	22.148000	24.278000	-13.489000
N	22.809000	23.673000	-12.494000
N	22.451000	25.538000	-13.833000
C	17.217000	21.167000	-11.687000
O	17.550000	20.008000	-11.448000
N	16.186000	21.448000	-12.472000
C	15.383000	20.397000	-13.087000
C	15.659000	20.314000	-14.571000
O	16.637000	20.872000	-15.047000
N	14.807000	19.621000	-15.307000
C	15.005000	19.427000	-16.727000
C	15.654000	18.068000	-16.977000
C	14.709000	16.861000	-16.897000
S	15.676000	15.351000	-16.855000
C	14.589000	14.235000	-15.985000
C	13.684000	19.507000	-17.480000
O	12.626000	19.225000	-16.931000
N	13.736000	19.883000	-18.748000
C	12.507000	20.112000	-19.475000
C	12.649000	21.329000	-20.382000
C	12.940000	22.614000	-19.624000
C	11.751000	23.090000	-18.775000
O	11.605000	24.290000	-18.505000
N	10.894000	22.149000	-18.358000
C	12.204000	18.899000	-20.293000
O	13.117000	18.292000	-20.824000
N	10.926000	18.546000	-20.397000
C	10.520000	17.377000	-21.156000
C	9.951000	16.285000	-20.231000
C	10.704000	15.912000	-18.956000
C	9.862000	15.019000	-18.043000
C	12.020000	15.242000	-19.344000
C	9.433000	17.810000	-22.094000
O	8.589000	18.611000	-21.721000
N	9.411000	17.262000	-23.293000
C	8.307000	17.532000	-24.201000
C	8.762000	17.535000	-25.676000
C	7.596000	17.850000	-26.629000
O	9.777000	18.529000	-25.830000
C	7.300000	16.428000	-23.999000
O	7.645000	15.242000	-24.008000
N	6.055000	16.820000	-23.795000
C	4.992000	15.845000	-23.612000
C	4.530000	15.776000	-22.137000
C	3.553000	14.594000	-21.933000
C	5.740000	15.727000	-21.165000
C	3.850000	16.364000	-24.438000
O	3.406000	17.489000	-24.220000
N	3.372000	15.557000	-25.376000
C	2.225000	15.951000	-26.200000
C	0.902000	15.870000	-25.429000

C	0.667000	14.571000	-24.701000
C	0.003000	14.571000	-23.480000
C	-0.242000	13.394000	-22.794000
C	0.159000	12.209000	-23.319000
C	0.821000	12.187000	-24.538000
C	1.069000	13.374000	-25.231000
C	2.361000	17.396000	-26.649000
O	1.479000	18.206000	-26.353000
N	3.451000	17.731000	-27.331000
C	3.627000	19.077000	-27.883000
C	3.587000	20.220000	-26.890000
O	3.153000	21.326000	-27.214000
N	4.040000	19.979000	-25.674000
C	3.981000	21.014000	-24.654000
C	5.179000	20.729000	-23.811000
O	5.935000	19.832000	-24.148000
N	5.361000	21.466000	-22.722000
C	6.472000	21.210000	-21.826000
C	7.420000	22.389000	-21.824000
C	8.602000	22.087000	-20.923000
O	7.868000	22.606000	-23.169000
C	6.117000	20.961000	-20.358000
O	5.407000	21.750000	-19.720000
N	6.690000	19.900000	-19.806000
C	6.638000	19.686000	-18.385000
C	6.038000	18.291000	-18.075000
C	6.164000	17.995000	-16.606000
C	4.567000	18.214000	-18.525000
C	8.061000	19.785000	-17.862000
O	8.964000	19.308000	-18.515000
N	8.254000	20.402000	-16.693000
C	9.538000	20.384000	-15.945000
C	9.799000	21.741000	-15.275000
C	11.226000	21.851000	-14.784000
O	9.504000	22.786000	-16.203000
C	9.599000	19.324000	-14.808000
O	8.715000	19.274000	-13.930000
N	10.660000	18.502000	-14.822000
C	10.835000	17.368000	-13.906000
C	11.113000	16.105000	-14.680000
C	11.997000	17.640000	-12.995000
O	13.084000	18.050000	-13.468000
N	11.791000	17.433000	-11.701000
C	12.905000	17.425000	-10.743000
C	12.713000	18.447000	-9.629000
C	12.538000	19.868000	-10.130000
C	11.334000	20.281000	-10.693000
C	11.159000	21.596000	-11.165000
C	12.196000	22.512000	-11.035000
C	13.408000	22.106000	-10.447000
C	13.568000	20.795000	-10.001000
C	12.960000	16.047000	-10.138000
O	12.203000	15.749000	-9.197000
N	13.813000	15.201000	-10.705000
C	13.933000	13.822000	-10.265000
C	14.220000	12.936000	-11.459000
C	13.271000	13.069000	-12.627000
C	13.625000	12.082000	-13.703000

C	11.832000	12.813000	-12.158000
C	15.038000	13.646000	-9.210000
O	16.200000	14.077000	-9.408000
N	14.692000	13.006000	-8.101000
C	15.643000	12.737000	-7.040000
C	16.101000	13.872000	-6.142000
O	17.317000	14.011000	-5.899000
N	15.179000	14.680000	-5.607000
C	15.554000	15.667000	-4.565000
C	14.529000	16.832000	-4.500000
C	15.019000	17.948000	-3.593000
C	14.138000	17.335000	-5.896000
C	13.011000	18.384000	-5.863000
C	15.622000	14.988000	-3.188000
O	14.708000	14.204000	-2.831000
N	16.709000	15.237000	-2.409000
C	17.877000	16.134000	-2.611000
C	18.640000	16.035000	-1.313000
C	18.215000	14.708000	-0.667000
C	16.754000	14.526000	-1.140000
C	15.797000	15.193000	-0.164000
O	15.627000	16.409000	-0.228000
N	15.224000	14.427000	0.761000
C	14.304000	15.003000	1.731000
C	12.825000	14.658000	1.429000
C	12.425000	13.204000	1.625000
C	12.494000	12.312000	0.590000
C	12.103000	10.968000	0.757000
C	11.598000	10.535000	1.955000
O	11.217000	9.205000	2.119000
C	11.499000	11.412000	2.998000
C	11.929000	12.756000	2.829000
C	14.644000	14.626000	3.158000
O	13.984000	15.082000	4.088000
N	15.681000	13.803000	3.338000
C	16.166000	13.438000	4.691000
C	15.385000	12.232000	5.207000
C	17.687000	13.137000	4.731000
O	18.321000	12.930000	3.711000
N	18.278000	13.084000	5.911000
C	19.655000	12.601000	6.005000
C	20.213000	12.783000	7.429000
C	20.752000	14.213000	7.709000
C	20.886000	14.506000	9.209000
O	20.965000	13.577000	10.036000
N	20.906000	15.805000	9.574000
C	19.644000	11.125000	5.618000
O	18.745000	10.397000	6.045000
N	20.630000	10.680000	4.806000
C	21.622000	11.496000	4.065000
C	22.225000	10.513000	3.093000
C	22.047000	9.171000	3.712000
C	20.705000	9.266000	4.458000
C	20.685000	8.427000	5.760000
O	21.392000	8.757000	6.704000
N	19.835000	7.392000	5.834000
C	18.831000	6.984000	4.828000
C	18.098000	5.866000	5.501000

C	18.269000	6.086000	6.998000
C	19.690000	6.650000	7.099000
C	20.694000	5.516000	7.244000
O	20.316000	4.339000	7.292000
N	21.976000	5.870000	7.331000
C	23.065000	4.897000	7.299000
C	24.079000	5.290000	6.233000
C	23.503000	5.768000	4.920000
C	24.546000	6.523000	4.095000
C	22.940000	4.601000	4.135000
C	23.794000	4.816000	8.612000
O	23.847000	5.780000	9.371000
N	24.394000	3.672000	8.885000
C	25.254000	3.589000	10.062000
C	24.471000	3.505000	11.337000
O	23.759000	2.511000	11.566000
N	24.593000	4.528000	12.187000
C	23.846000	4.561000	13.456000
C	24.577000	5.414000	14.518000
C	24.120000	6.860000	14.622000
C	25.254000	7.860000	14.750000
N	25.501000	8.588000	13.499000
C	24.901000	9.729000	13.152000
N	23.999000	10.286000	13.963000
N	25.199000	10.320000	11.995000
C	22.378000	5.035000	13.264000
O	21.570000	4.957000	14.202000
N	22.058000	5.518000	12.051000
C	20.706000	5.937000	11.683000
C	20.739000	7.074000	10.645000
C	21.415000	8.356000	11.163000
C	21.422000	9.496000	10.151000
C	20.773000	8.787000	12.499000
C	19.853000	4.774000	11.179000
O	18.612000	4.898000	11.081000
N	20.504000	3.652000	10.861000
C	19.786000	2.475000	10.443000
C	20.735000	1.304000	10.159000
C	20.018000	0.130000	9.508000
C	20.835000	-1.158000	9.448000
N	21.651000	-1.217000	8.243000
C	22.525000	-2.185000	7.963000
N	22.678000	-3.210000	8.806000
N	23.219000	-2.129000	6.827000
C	18.753000	2.071000	11.492000
O	19.074000	1.978000	12.691000
N	17.525000	1.809000	11.028000
C	16.353000	1.456000	11.866000
C	16.618000	0.339000	12.868000
C	17.160000	-0.917000	12.273000
C	16.489000	-1.574000	11.269000
C	17.006000	-2.748000	10.731000
C	18.190000	-3.270000	11.213000
C	18.852000	-2.628000	12.231000
C	18.347000	-1.473000	12.763000
C	15.711000	2.601000	12.643000
O	14.714000	2.373000	13.355000
N	16.249000	3.811000	12.520000

C	15.692000	4.931000	13.255000
C	16.804000	5.829000	13.809000
C	17.561000	5.168000	14.957000
C	18.674000	6.051000	15.449000
C	18.158000	7.218000	16.249000
N	18.107000	6.903000	17.715000
C	14.770000	5.776000	12.379000
O	14.848000	5.707000	11.159000
N	13.921000	6.575000	13.031000
C	13.078000	7.549000	12.366000
C	12.262000	8.353000	13.419000
C	11.069000	7.546000	13.996000
C	10.440000	8.180000	15.218000
C	9.981000	9.617000	15.000000
N	8.902000	10.104000	15.959000
C	14.015000	8.425000	11.520000
O	15.185000	8.600000	11.910000
N	13.526000	8.937000	10.359000
C	12.113000	8.824000	9.937000
C	11.987000	9.837000	8.822000
C	13.389000	9.903000	8.214000
C	14.325000	9.726000	9.425000
C	14.661000	11.074000	10.046000
O	13.844000	11.634000	10.782000
N	15.860000	11.587000	9.778000
C	16.298000	12.832000	10.404000
C	17.723000	12.718000	10.992000
C	17.984000	11.506000	11.917000
C	17.135000	11.479000	13.190000
O	17.029000	12.492000	13.887000
N	16.542000	10.307000	13.516000
C	16.220000	13.927000	9.346000
O	16.502000	13.695000	8.170000
N	15.817000	15.128000	9.758000
C	15.526000	16.187000	8.791000
C	14.682000	17.317000	9.416000
O	15.448000	18.110000	10.298000
C	16.805000	16.674000	8.092000
O	17.915000	16.357000	8.530000
N	16.653000	17.434000	7.013000
C	17.762000	17.641000	6.096000
C	17.259000	17.724000	4.660000
C	18.289000	17.341000	3.609000
C	19.248000	16.277000	4.115000
C	17.549000	16.896000	2.371000
C	18.588000	18.862000	6.423000
O	18.049000	19.955000	6.565000
N	19.905000	18.627000	6.494000
C	20.948000	19.590000	6.862000
C	22.398000	18.945000	6.819000
C	22.888000	18.471000	8.220000
O	22.425000	17.835000	5.901000
C	20.988000	20.822000	5.978000
O	21.002000	21.936000	6.500000
N	21.058000	20.636000	4.656000
C	21.118000	21.780000	3.722000
C	20.817000	23.095000	4.439000
C	21.351000	24.335000	3.719000

C	20.635000	25.611000	4.217000
C	21.425000	26.904000	3.913000
N	22.638000	27.087000	4.795000
C	22.438000	21.935000	2.958000
O	23.519000	21.879000	3.547000
N	22.330000	22.151000	1.646000
C	23.487000	22.300000	0.772000
C	23.427000	21.289000	-0.381000
C	22.260000	21.505000	-1.282000
C	22.235000	22.240000	-2.436000
N	20.976000	22.212000	-2.990000
C	20.161000	21.463000	-2.185000
C	18.812000	21.150000	-2.320000
C	18.254000	20.355000	-1.359000
C	18.996000	19.879000	-0.282000
C	20.327000	20.199000	-0.136000
C	20.935000	21.003000	-1.102000
C	23.573000	23.727000	0.221000
O	22.641000	24.532000	0.349000
N	24.722000	24.046000	-0.357000
C	24.857000	25.258000	-1.138000
C	25.821000	26.268000	-0.497000
O	27.143000	26.099000	-0.967000
C	25.317000	24.774000	-2.501000
O	25.598000	23.573000	-2.686000
N	25.378000	25.695000	-3.455000
C	25.466000	25.308000	-4.860000
C	26.654000	24.354000	-5.127000
C	28.027000	25.043000	-4.948000
O	28.061000	26.306000	-4.874000
O	29.072000	24.327000	-4.879000
C	24.130000	24.654000	-5.256000
O	23.197000	24.555000	-4.438000
N	24.065000	24.214000	-6.512000
C	22.843000	23.742000	-7.122000
C	22.730000	24.262000	-8.551000
C	21.433000	23.721000	-9.240000
C	22.809000	25.793000	-8.552000
C	22.673000	26.416000	-9.925000
C	22.791000	22.224000	-7.158000
O	23.626000	21.602000	-7.815000
N	21.821000	21.635000	-6.443000
C	21.542000	20.202000	-6.553000
C	20.648000	19.717000	-5.417000
C	20.432000	18.251000	-5.486000
C	19.689000	17.568000	-6.409000
N	19.764000	16.216000	-6.170000
C	20.569000	16.001000	-5.081000
C	20.942000	14.810000	-4.460000
C	21.775000	14.890000	-3.371000
C	22.232000	16.129000	-2.886000
C	21.858000	17.329000	-3.513000
C	21.013000	17.266000	-4.627000
C	20.885000	19.901000	-7.897000
O	19.854000	20.461000	-8.243000
N	21.492000	19.014000	-8.662000
C	20.982000	18.671000	-9.972000
C	22.142000	18.286000	-10.887000

C	22.931000	19.501000	-11.377000
O	22.374000	20.601000	-11.531000
N	24.240000	19.309000	-11.610000
C	19.989000	17.511000	-9.875000
O	20.399000	16.388000	-9.629000
N	18.695000	17.786000	-10.056000
C	17.649000	16.753000	-9.923000
C	16.433000	17.308000	-9.204000
C	17.271000	16.310000	-11.315000
O	16.203000	16.651000	-11.831000
N	18.161000	15.551000	-11.940000
C	18.046000	15.362000	-13.359000
C	19.186000	16.117000	-14.073000
C	18.940000	17.617000	-13.961000
O	20.413000	15.851000	-13.377000
C	18.005000	13.882000	-13.689000
O	17.837000	13.495000	-14.843000
N	18.107000	13.053000	-12.664000
C	17.863000	11.609000	-12.825000
C	19.155000	10.859000	-13.215000
C	20.324000	11.006000	-12.247000
C	21.352000	9.856000	-12.487000
C	22.709000	10.080000	-11.778000
N	22.636000	10.069000	-10.259000
C	17.203000	10.993000	-11.579000
O	17.295000	11.554000	-10.499000
N	16.522000	9.859000	-11.729000
C	15.936000	9.210000	-10.555000
C	15.179000	7.978000	-10.990000
C	13.858000	8.229000	-11.672000
C	12.756000	8.648000	-10.947000
C	11.512000	8.846000	-11.566000
C	11.391000	8.590000	-12.887000
O	10.173000	8.761000	-13.485000
C	12.479000	8.162000	-13.638000
C	13.700000	7.983000	-13.021000
C	16.996000	8.761000	-9.562000
O	18.070000	8.356000	-9.967000
N	16.697000	8.791000	-8.269000
C	17.660000	8.334000	-7.267000
C	17.455000	9.046000	-5.936000
C	17.687000	6.825000	-7.065000
O	16.951000	6.086000	-7.746000
N	18.553000	6.369000	-6.150000
C	18.600000	4.967000	-5.779000
C	19.584000	4.725000	-4.640000
C	21.059000	5.087000	-4.988000
O	21.499000	4.982000	-6.127000
N	21.815000	5.443000	-3.964000
C	17.244000	4.544000	-5.195000
O	16.639000	5.321000	-4.465000
N	16.842000	3.292000	-5.456000
C	15.732000	2.657000	-4.743000
C	15.166000	1.537000	-5.614000
O	14.801000	2.089000	-6.844000
C	16.220000	2.083000	-3.406000
O	17.425000	1.755000	-3.266000
N	15.320000	1.936000	-2.433000

C	15.697000	1.350000	-1.160000
C	14.597000	1.592000	-0.082000
S	14.199000	3.373000	0.183000
C	16.002000	-0.139000	-1.315000
O	15.528000	-0.782000	-2.243000
N	16.772000	-0.688000	-0.379000
C	17.128000	-2.093000	-0.439000
C	18.052000	-2.477000	0.720000
S	19.584000	-1.540000	0.704000
C	15.901000	-2.971000	-0.379000
O	14.981000	-2.720000	0.421000
N	15.915000	-4.039000	-1.163000
C	14.766000	-4.912000	-1.212000
C	13.589000	-4.164000	-1.853000
C	13.910000	-3.624000	-3.241000
C	12.798000	-2.766000	-3.810000
O	11.899000	-3.262000	-4.524000
N	12.844000	-1.468000	-3.505000
C	15.084000	-6.146000	-2.035000
O	15.885000	-6.072000	-2.976000
N	14.444000	-7.252000	-1.665000
C	14.448000	-8.463000	-2.436000
C	13.819000	-9.604000	-1.626000
C	14.702000	-10.031000	-0.470000
O	15.896000	-10.297000	-0.656000
N	14.142000	-10.083000	0.736000
C	13.684000	-8.212000	-3.701000
O	12.872000	-7.305000	-3.747000
N	13.949000	-9.008000	-4.726000
C	13.454000	-8.762000	-6.069000
C	14.653000	-8.477000	-7.058000
C	14.302000	-8.843000	-8.480000
C	15.068000	-6.988000	-7.058000
C	15.847000	-6.590000	-5.866000
C	12.671000	-10.002000	-6.483000
O	12.966000	-11.082000	-6.036000
N	11.662000	-9.845000	-7.329000
C	10.876000	-10.992000	-7.823000
C	9.445000	-10.497000	-8.176000
C	8.620000	-11.529000	-8.939000
O	7.567000	-11.154000	-9.507000
O	9.009000	-12.712000	-8.961000
C	11.600000	-11.747000	-9.010000
O	11.732000	-11.201000	-10.116000
N	12.083000	-12.973000	-8.746000
C	12.882000	-13.784000	-9.709000
C	14.156000	-14.365000	-9.090000
C	15.070000	-13.382000	-8.343000
C	16.046000	-14.109000	-7.372000
O	16.571000	-15.173000	-7.696000
N	16.291000	-13.515000	-6.200000
C	12.112000	-14.991000	-10.180000
O	12.716000	-15.933000	-10.703000
N	10.792000	-14.987000	-9.992000
C	9.987000	-16.115000	-10.445000
C	8.564000	-16.077000	-9.837000
O	8.654000	-16.044000	-8.425000
C	9.916000	-16.259000	-11.942000

O	9.857000	-17.365000	-12.430000
N	9.890000	-15.166000	-12.691000
C	9.768000	-15.294000	-14.149000
C	8.351000	-14.903000	-14.613000
C	7.247000	-15.569000	-13.815000
C	6.880000	-16.893000	-14.071000
C	5.877000	-17.514000	-13.341000
C	5.234000	-16.816000	-12.341000
C	5.591000	-15.482000	-12.077000
C	6.576000	-14.873000	-12.811000
C	10.821000	-14.464000	-14.887000
O	10.498000	-13.486000	-15.558000
N	12.104000	-14.852000	-14.779000
C	12.680000	-16.061000	-14.159000
C	14.153000	-15.690000	-14.080000
C	14.391000	-14.851000	-15.303000
C	13.130000	-14.006000	-15.432000
C	12.810000	-13.817000	-16.897000
O	12.389000	-14.752000	-17.541000
N	12.973000	-12.616000	-17.424000
C	12.704000	-12.385000	-18.835000
C	11.255000	-12.057000	-19.161000
O	10.939000	-11.717000	-20.286000
N	10.366000	-12.178000	-18.168000
C	8.914000	-11.912000	-18.346000
C	8.102000	-12.953000	-17.564000
C	6.610000	-12.824000	-17.748000
C	6.059000	-12.749000	-19.013000
C	4.673000	-12.632000	-19.183000
C	3.862000	-12.573000	-18.072000
C	4.404000	-12.637000	-16.812000
C	5.768000	-12.776000	-16.643000
C	8.525000	-10.476000	-17.938000
O	8.690000	-10.083000	-16.769000
N	8.040000	-9.686000	-18.902000
C	7.655000	-8.294000	-18.648000
C	7.237000	-7.573000	-19.941000
C	7.006000	-6.102000	-19.747000
N	7.989000	-5.254000	-19.274000
C	7.498000	-4.030000	-19.175000
N	6.234000	-4.053000	-19.568000
C	5.903000	-5.335000	-19.935000
C	6.561000	-8.143000	-17.536000
O	6.597000	-7.178000	-16.725000
N	5.631000	-9.101000	-17.479000
C	4.529000	-9.049000	-16.494000
C	4.985000	-8.915000	-15.050000
O	4.391000	-8.165000	-14.271000
N	6.039000	-9.635000	-14.662000
C	6.570000	-9.527000	-13.313000
C	7.085000	-10.888000	-12.848000
O	8.064000	-11.430000	-13.732000
C	7.712000	-8.529000	-13.210000
O	7.862000	-7.804000	-12.209000
N	8.554000	-8.539000	-14.238000
C	9.740000	-7.719000	-14.219000
C	10.722000	-8.148000	-15.328000
C	11.275000	-9.604000	-15.160000

C	12.581000	-9.813000	-15.964000
O	13.416000	-10.636000	-15.547000
O	12.763000	-9.118000	-16.995000
C	9.444000	-6.232000	-14.279000
O	10.231000	-5.419000	-13.785000
N	8.343000	-5.841000	-14.902000
C	7.947000	-4.419000	-14.833000
C	6.621000	-4.178000	-15.547000
C	5.465000	-4.904000	-14.938000
S	3.992000	-4.540000	-15.935000
C	3.626000	-2.895000	-15.290000
C	7.804000	-3.814000	-13.430000
O	7.762000	-2.580000	-13.302000
N	7.621000	-4.658000	-12.413000
C	7.481000	-4.191000	-11.010000
C	6.459000	-5.060000	-10.238000
C	5.132000	-5.135000	-10.967000
C	4.652000	-6.177000	-11.716000
N	3.410000	-5.862000	-12.235000
C	3.091000	-4.584000	-11.847000
C	1.950000	-3.824000	-12.110000
C	1.883000	-2.558000	-11.580000
C	2.929000	-2.041000	-10.781000
C	4.058000	-2.808000	-10.503000
C	4.151000	-4.100000	-11.046000
C	8.828000	-4.178000	-10.269000
O	8.953000	-3.629000	-9.154000
N	9.859000	-4.774000	-10.865000
C	11.149000	-4.786000	-10.172000
C	12.078000	-5.865000	-10.747000
C	11.664000	-7.288000	-10.356000
O	11.162000	-7.550000	-9.244000
N	11.893000	-8.220000	-11.268000
C	11.781000	-3.389000	-10.223000
O	11.522000	-2.605000	-11.137000
N	12.635000	-3.076000	-9.255000
C	13.044000	-3.985000	-8.176000
C	13.758000	-3.083000	-7.230000
C	14.294000	-1.953000	-8.040000
C	13.334000	-1.795000	-9.212000
C	14.145000	-1.502000	-10.469000
O	14.698000	-2.405000	-11.075000
N	14.209000	-0.234000	-10.867000
C	14.963000	0.129000	-12.033000
C	14.015000	0.576000	-13.156000
C	13.226000	1.826000	-12.801000
O	13.199000	2.253000	-11.657000
N	12.552000	2.396000	-13.782000
C	16.006000	1.224000	-11.759000
O	16.382000	1.936000	-12.659000
N	16.404000	1.408000	-10.510000
C	17.614000	2.157000	-10.186000
C	17.340000	3.584000	-9.609000
C	16.501000	4.410000	-10.533000
O	16.726000	3.488000	-8.327000
C	18.405000	1.307000	-9.192000
O	17.844000	0.395000	-8.587000
N	19.698000	1.574000	-9.027000

C	20.499000	0.891000	-8.004000
C	21.823000	1.612000	-7.755000
C	22.841000	1.262000	-8.754000
O	23.270000	0.081000	-8.779000
O	23.189000	2.171000	-9.511000
C	19.825000	0.916000	-6.683000
O	19.324000	1.960000	-6.324000
N	19.914000	-0.206000	-5.953000
C	19.472000	-0.328000	-4.580000
C	19.265000	-1.796000	-4.203000
C	18.369000	-2.633000	-5.125000
C	18.078000	-3.985000	-4.488000
C	17.060000	-1.897000	-5.449000
C	20.529000	0.271000	-3.661000
O	21.729000	0.225000	-3.961000
N	20.116000	0.810000	-2.529000
C	21.077000	1.409000	-1.628000
C	21.654000	2.633000	-2.314000
O	22.296000	3.436000	-1.344000
C	20.321000	1.826000	-0.379000
O	19.160000	2.176000	-0.474000
N	20.950000	1.787000	0.788000
C	20.301000	2.330000	1.982000
C	21.113000	2.040000	3.220000
C	21.083000	0.593000	3.631000
C	21.718000	0.438000	4.952000
O	22.925000	0.121000	4.997000
O	21.048000	0.705000	5.958000
C	20.107000	3.838000	1.861000
O	19.315000	4.455000	2.587000
N	20.859000	4.424000	0.936000
C	20.793000	5.843000	0.683000
C	22.189000	6.346000	0.318000
C	22.197000	7.797000	-0.080000
O	21.153000	8.484000	0.079000
O	23.254000	8.258000	-0.582000
C	19.738000	6.028000	-0.433000
O	20.059000	6.090000	-1.619000
N	18.452000	6.045000	-0.020000
C	17.336000	6.022000	-0.941000
C	16.739000	4.614000	-0.931000
S	16.043000	4.195000	0.665000
C	16.242000	7.104000	-0.600000
O	15.182000	7.177000	-1.260000
N	16.495000	7.974000	0.385000
C	15.436000	8.906000	0.801000
C	15.557000	9.251000	2.290000
C	15.393000	8.042000	3.222000
C	15.344000	8.435000	4.693000
C	14.145000	7.199000	2.828000
C	15.338000	10.155000	-0.063000
O	15.760000	11.229000	0.363000
N	14.747000	9.995000	-1.262000
C	14.547000	11.043000	-2.279000
C	15.476000	10.822000	-3.482000
C	16.939000	10.756000	-3.033000
C	17.482000	9.580000	-2.518000
C	18.802000	9.531000	-2.076000

C	19.571000	10.673000	-2.135000
O	20.894000	10.653000	-1.684000
C	19.047000	11.833000	-2.642000
C	17.745000	11.872000	-3.093000
C	13.112000	11.062000	-2.792000
O	12.357000	10.049000	-2.623000
N	12.763000	12.190000	-3.413000
C	11.461000	12.402000	-4.046000
C	10.513000	13.186000	-3.112000
C	10.990000	14.514000	-2.514000
C	10.751000	15.677000	-3.451000
C	10.266000	14.750000	-1.203000
C	11.572000	13.085000	-5.400000
O	12.648000	13.599000	-5.776000
N	10.471000	13.068000	-6.144000
C	10.385000	13.631000	-7.483000
C	10.102000	12.538000	-8.510000
C	11.002000	11.347000	-8.347000
O	12.239000	11.473000	-8.354000
N	10.394000	10.170000	-8.161000
C	9.216000	14.608000	-7.520000
O	8.185000	14.405000	-6.825000
N	9.363000	15.655000	-8.327000
C	8.303000	16.632000	-8.523000
C	8.654000	17.973000	-7.908000
C	7.491000	18.945000	-8.114000
C	8.955000	17.838000	-6.450000
C	8.166000	16.848000	-10.024000
O	9.182000	16.999000	-10.717000
N	6.943000	16.847000	-10.548000
C	6.733000	17.255000	-11.932000
C	6.067000	16.159000	-12.745000
C	6.895000	14.934000	-13.078000
C	7.597000	14.716000	-14.245000
N	8.181000	13.451000	-14.205000
C	7.841000	12.818000	-13.030000
C	8.182000	11.545000	-12.551000
C	7.688000	11.183000	-11.314000
C	6.901000	12.041000	-10.568000
C	6.535000	13.279000	-11.050000
C	7.018000	13.698000	-12.298000
C	5.775000	18.418000	-11.918000
O	4.723000	18.332000	-11.276000
N	6.110000	19.489000	-12.633000
C	5.268000	20.644000	-12.677000
C	5.868000	21.830000	-11.876000
C	6.373000	21.346000	-10.514000
C	6.980000	22.519000	-12.655000
C	7.513000	23.776000	-11.974000
C	4.980000	21.018000	-14.126000
O	5.780000	20.734000	-15.026000
N	3.809000	21.608000	-14.365000
C	2.787000	21.919000	-13.346000
C	1.604000	22.259000	-14.147000
C	2.020000	22.596000	-15.538000
C	3.466000	22.102000	-15.692000
C	4.375000	23.297000	-16.055000
O	4.842000	23.995000	-15.149000

N	4.631000	23.487000	-17.356000
C	5.287000	24.686000	-17.878000
C	6.486000	24.316000	-18.655000
C	4.286000	25.394000	-18.787000
O	3.694000	24.760000	-19.642000
N	4.092000	26.714000	-18.604000
C	3.257000	27.547000	-19.497000
C	3.181000	28.856000	-18.757000
C	4.477000	28.955000	-17.999000
C	4.815000	27.512000	-17.610000
C	4.375000	27.240000	-16.178000
O	3.235000	26.826000	-15.935000
N	5.305000	27.457000	-15.256000
C	5.082000	27.357000	-13.838000
C	6.059000	28.272000	-13.138000
C	6.404000	27.874000	-11.752000
C	6.837000	29.110000	-11.016000
C	7.654000	28.786000	-9.792000
N	7.556000	29.957000	-8.883000
C	3.680000	27.820000	-13.531000
O	3.330000	28.928000	-13.885000
N	2.873000	26.959000	-12.874000
C	3.286000	25.645000	-12.338000
C	2.153000	25.268000	-11.446000
C	0.962000	25.925000	-12.012000
C	1.506000	27.278000	-12.512000
C	1.557000	28.269000	-11.374000
O	2.642000	28.524000	-10.857000
N	0.401000	28.798000	-10.968000
C	0.370000	29.818000	-9.938000
C	-0.590000	30.946000	-10.337000
C	-0.012000	31.915000	-11.388000
C	0.558000	33.183000	-10.752000
C	1.158000	34.101000	-11.844000
N	0.535000	33.690000	-13.157000
C	0.050000	29.308000	-8.523000
O	0.598000	29.807000	-7.520000
N	-0.807000	28.310000	-8.405000
C	-1.139000	27.857000	-7.077000
C	-2.062000	28.902000	-6.447000
C	-2.176000	28.793000	-4.944000
O	-1.188000	28.586000	-4.232000
N	-3.425000	28.955000	-4.439000
C	-1.857000	26.539000	-7.360000
O	-3.050000	26.393000	-7.111000
N	-1.111000	25.599000	-7.934000
C	-1.674000	24.384000	-8.505000
C	-0.700000	23.777000	-9.480000
C	-1.979000	23.396000	-7.396000
O	-1.333000	23.400000	-6.327000
N	-2.959000	22.544000	-7.653000
C	-3.243000	21.409000	-6.754000
C	-4.618000	20.820000	-7.083000
C	-4.717000	19.387000	-6.605000
O	-5.626000	21.627000	-6.453000
C	-2.149000	20.320000	-6.850000
O	-1.636000	20.016000	-7.947000
N	-1.811000	19.736000	-5.700000

C	-0.751000	18.737000	-5.609000
C	0.197000	19.114000	-4.443000
C	1.324000	18.148000	-4.322000
C	0.760000	20.537000	-4.678000
C	-1.285000	17.297000	-5.423000
O	-2.090000	17.055000	-4.557000
N	-0.824000	16.365000	-6.260000
C	-1.058000	14.918000	-6.111000
C	-1.523000	14.352000	-7.458000
C	-3.046000	14.315000	-7.629000
C	-3.656000	15.664000	-7.289000
C	-3.472000	13.903000	-8.995000
C	0.243000	14.216000	-5.618000
O	1.273000	14.233000	-6.310000
N	0.229000	13.640000	-4.402000
C	1.372000	12.853000	-3.887000
C	1.681000	13.252000	-2.457000
C	2.898000	12.487000	-1.928000
C	1.865000	14.767000	-2.375000
C	2.160000	15.219000	-1.031000
C	1.151000	11.348000	-3.944000
O	0.227000	10.801000	-3.253000
N	1.949000	10.665000	-4.775000
C	1.824000	9.166000	-4.957000
C	2.264000	8.785000	-6.380000
C	2.263000	7.318000	-6.668000
C	3.353000	6.496000	-6.789000
N	2.923000	5.196000	-7.002000
C	1.550000	5.180000	-7.071000
C	0.681000	4.125000	-7.328000
C	-0.648000	4.404000	-7.349000
C	-1.127000	5.686000	-7.129000
C	-0.274000	6.728000	-6.872000
C	1.099000	6.479000	-6.844000
C	2.632000	8.355000	-3.931000
O	3.813000	8.712000	-3.640000
N	2.023000	7.310000	-3.349000
C	2.726000	6.396000	-2.463000
C	2.133000	6.403000	-0.984000
C	2.947000	5.431000	-0.063000
C	2.045000	7.849000	-0.383000
C	1.483000	7.939000	1.045000
C	2.610000	4.994000	-3.059000
O	1.485000	4.461000	-3.186000
N	3.746000	4.394000	-3.448000
C	3.732000	3.096000	-4.156000
C	5.058000	2.854000	-4.917000
C	6.310000	2.869000	-4.027000
C	6.590000	1.837000	-3.140000
C	7.750000	1.883000	-2.318000
C	8.642000	2.938000	-2.473000
O	9.794000	3.038000	-1.725000
C	8.390000	3.924000	-3.321000
C	7.253000	3.894000	-4.134000
C	3.420000	1.891000	-3.282000
O	3.724000	1.863000	-2.067000
N	2.838000	0.857000	-3.899000
C	2.628000	-0.406000	-3.206000

C	3.829000	-1.366000	-3.387000
O	4.875000	-1.002000	-3.951000
N	3.693000	-2.590000	-2.888000
C	4.798000	-3.515000	-2.846000
C	4.863000	-4.305000	-1.561000
O	5.929000	-4.769000	-1.164000
N	3.721000	-4.458000	-0.894000
C	3.663000	-5.315000	0.311000
C	4.399000	-4.781000	1.536000
O	4.583000	-5.507000	2.503000
N	4.799000	-3.506000	1.497000
C	5.749000	-2.917000	2.473000
C	5.219000	-2.909000	3.919000
C	3.897000	-2.109000	4.113000
C	3.906000	-0.720000	4.179000
C	2.733000	0.024000	4.366000
C	1.491000	-0.666000	4.488000
C	1.470000	-2.104000	4.434000
C	2.671000	-2.802000	4.242000
C	7.180000	-3.542000	2.417000
O	8.033000	-3.220000	3.285000
N	7.451000	-4.370000	1.394000
C	8.700000	-5.165000	1.337000
C	8.449000	-6.683000	1.265000
C	7.529000	-7.336000	2.329000
C	7.789000	-6.883000	3.718000
O	8.865000	-7.124000	4.274000
N	6.808000	-6.187000	4.311000
C	9.491000	-4.776000	0.107000
O	10.695000	-5.028000	0.054000
N	8.827000	-4.133000	-0.855000
C	9.379000	-3.777000	-2.144000
C	9.136000	-4.896000	-3.172000
C	9.719000	-6.251000	-2.661000
O	7.712000	-5.005000	-3.438000
C	8.747000	-2.497000	-2.702000
O	7.753000	-2.005000	-2.167000
N	9.322000	-1.963000	-3.765000
C	8.703000	-0.928000	-4.573000
C	9.631000	0.224000	-4.817000
O	10.620000	0.353000	-4.112000
N	9.308000	1.087000	-5.781000
C	10.132000	2.297000	-5.949000
C	11.481000	1.958000	-6.667000
C	11.226000	1.399000	-8.061000
O	12.275000	3.141000	-6.789000
C	9.357000	3.359000	-6.736000
O	8.467000	3.007000	-7.548000
N	9.638000	4.642000	-6.516000
C	8.917000	5.677000	-7.269000
C	8.968000	7.034000	-6.571000
O	10.278000	7.361000	-6.162000
C	9.265000	5.852000	-8.741000
O	8.569000	6.578000	-9.473000
N	10.315000	5.184000	-9.220000
C	10.794000	5.482000	-10.554000
C	12.327000	5.523000	-10.590000
O	12.871000	4.467000	-9.828000

C	10.284000	4.499000	-11.583000
O	10.704000	4.535000	-12.716000
N	9.361000	3.619000	-11.223000
C	8.727000	2.811000	-12.251000
C	7.721000	1.788000	-11.672000
C	8.183000	0.842000	-10.571000
C	7.123000	-0.302000	-10.326000
C	9.575000	0.212000	-10.907000
C	8.056000	3.666000	-13.315000
O	7.578000	4.790000	-13.077000
N	8.005000	3.098000	-14.514000
C	7.458000	3.762000	-15.697000
C	7.754000	2.818000	-16.903000
C	7.043000	3.175000	-18.173000
N	7.078000	4.448000	-18.715000
C	6.349000	4.475000	-19.826000
N	5.874000	3.257000	-20.040000
C	6.289000	2.427000	-19.018000
C	5.939000	4.012000	-15.510000
O	5.372000	4.983000	-16.033000
N	5.263000	3.102000	-14.801000
C	3.802000	3.240000	-14.636000
C	3.100000	1.881000	-14.317000
C	3.098000	0.967000	-15.572000
C	3.719000	1.222000	-13.106000
C	3.442000	4.312000	-13.590000
O	2.265000	4.686000	-13.481000
N	4.443000	4.809000	-12.835000
C	4.266000	5.916000	-11.899000
C	4.945000	5.640000	-10.561000
C	4.712000	4.289000	-9.922000
C	3.526000	3.582000	-10.112000
C	3.304000	2.334000	-9.473000
C	4.240000	1.826000	-8.641000
O	4.006000	0.594000	-8.052000
C	5.449000	2.522000	-8.426000
C	5.647000	3.750000	-9.065000
C	4.862000	7.269000	-12.413000
O	5.146000	8.183000	-11.632000
N	5.057000	7.397000	-13.705000
C	5.641000	8.645000	-14.254000
C	6.008000	8.407000	-15.705000
C	6.970000	9.457000	-16.257000
O	6.997000	10.604000	-15.757000
O	7.687000	9.102000	-17.219000
C	4.642000	9.777000	-14.257000
O	3.714000	9.764000	-15.036000
N	4.858000	10.799000	-13.436000
C	3.849000	11.839000	-13.303000
C	3.775000	12.887000	-14.400000
O	3.021000	13.860000	-14.289000
N	4.556000	12.731000	-15.463000
C	4.629000	13.828000	-16.447000
C	5.855000	13.664000	-17.354000
C	5.658000	12.675000	-18.445000
C	6.955000	12.369000	-19.155000
C	6.729000	11.341000	-20.276000
N	8.032000	10.850000	-20.819000

C	3.345000	13.981000	-17.294000
O	3.047000	15.065000	-17.784000
N	2.586000	12.912000	-17.501000
C	1.325000	13.073000	-18.230000
C	0.776000	11.708000	-18.619000
C	1.788000	10.852000	-19.319000
C	2.125000	11.105000	-20.656000
C	3.089000	10.336000	-21.325000
C	3.719000	9.317000	-20.656000
C	3.402000	9.060000	-19.301000
C	2.455000	9.850000	-18.642000
C	0.282000	13.873000	-17.465000
O	-0.404000	14.692000	-18.052000
N	0.141000	13.606000	-16.161000
C	-0.841000	14.275000	-15.303000
C	-0.862000	13.678000	-13.887000
C	-1.608000	12.338000	-13.726000
C	-1.040000	11.531000	-12.541000
C	-3.142000	12.520000	-13.614000
C	-0.552000	15.772000	-15.234000
O	-1.458000	16.595000	-15.390000
N	0.715000	16.120000	-15.011000
C	1.131000	17.530000	-14.992000
C	2.618000	17.622000	-14.682000
C	0.796000	18.193000	-16.354000
O	0.121000	19.234000	-16.429000
N	1.198000	17.550000	-17.441000
C	0.947000	18.111000	-18.763000
C	1.544000	17.175000	-19.807000
C	1.191000	17.458000	-21.259000
C	1.745000	18.813000	-21.787000
N	1.332000	18.962000	-23.181000
C	0.409000	19.819000	-23.616000
N	-0.173000	20.669000	-22.774000
N	0.086000	19.842000	-24.910000
C	-0.561000	18.339000	-19.024000
O	-0.962000	19.423000	-19.429000
N	-1.379000	17.308000	-18.772000
C	-2.791000	17.248000	-19.190000
C	-3.235000	15.780000	-19.361000
C	-4.725000	15.658000	-19.595000
C	-2.486000	15.160000	-20.543000
C	-3.751000	17.981000	-18.261000
O	-4.681000	18.616000	-18.734000
N	-3.529000	17.905000	-16.943000
C	-4.439000	18.506000	-15.975000
C	-4.936000	17.431000	-14.994000
C	-5.791000	16.354000	-15.644000
C	-7.202000	16.821000	-16.086000
O	-7.800000	17.708000	-15.449000
O	-7.730000	16.280000	-17.095000
C	-3.791000	19.655000	-15.197000
O	-4.412000	20.280000	-14.314000
N	-2.514000	19.904000	-15.476000
C	-1.858000	21.059000	-14.869000
C	-2.550000	22.367000	-15.294000
C	-2.799000	22.523000	-16.804000
C	-1.648000	23.140000	-17.566000

N	-1.143000	24.369000	-16.948000
C	-0.052000	25.012000	-17.369000
N	0.630000	24.540000	-18.420000
N	0.352000	26.120000	-16.751000
C	-1.903000	20.976000	-13.371000
O	-2.056000	21.977000	-12.707000
N	-1.779000	19.775000	-12.827000
C	-1.514000	19.586000	-11.409000
C	-2.274000	18.345000	-10.855000
C	-3.772000	18.642000	-10.804000
C	-1.966000	17.101000	-11.681000
C	-0.022000	19.349000	-11.256000
O	0.611000	18.951000	-12.215000
N	0.523000	19.598000	-10.055000
C	1.824000	19.063000	-9.647000
C	2.372000	19.971000	-8.578000
C	3.530000	19.327000	-7.932000
C	2.703000	21.330000	-9.249000
C	3.024000	22.441000	-8.308000
C	1.732000	17.631000	-9.091000
O	0.831000	17.316000	-8.286000
N	2.629000	16.756000	-9.535000
C	2.733000	15.386000	-9.021000
C	2.655000	14.379000	-10.186000
C	2.730000	12.913000	-9.724000
C	1.418000	14.664000	-11.022000
C	4.060000	15.230000	-8.252000
O	5.158000	15.618000	-8.734000
N	3.974000	14.698000	-7.044000
C	5.145000	14.370000	-6.254000
C	5.114000	15.158000	-4.932000
C	6.166000	14.681000	-3.977000
C	5.188000	16.651000	-5.168000
C	5.050000	12.863000	-5.922000
O	3.909000	12.320000	-5.758000
N	6.215000	12.197000	-5.873000
C	6.343000	10.827000	-5.379000
C	6.240000	9.813000	-6.491000
O	7.348000	9.850000	-7.391000
C	7.651000	10.675000	-4.595000
O	8.580000	11.413000	-4.826000
N	7.687000	9.753000	-3.630000
C	8.836000	9.584000	-2.738000
C	8.554000	10.200000	-1.351000
C	7.938000	9.266000	-0.314000
S	6.185000	8.773000	-0.645000
C	5.337000	10.300000	-0.160000
C	9.219000	8.105000	-2.591000
O	8.325000	7.195000	-2.632000
N	10.536000	7.854000	-2.406000
C	11.025000	6.568000	-1.912000
C	12.466000	6.231000	-2.435000
C	12.509000	6.017000	-3.924000
O	11.456000	5.909000	-4.576000
N	13.727000	5.957000	-4.499000
C	11.029000	6.544000	-0.407000
O	11.460000	7.522000	0.247000
N	10.490000	5.448000	0.154000

C	10.495000	5.231000	1.595000
C	9.085000	5.403000	2.218000
C	8.073000	4.410000	1.671000
C	7.865000	3.181000	2.279000
C	6.903000	2.243000	1.753000
C	6.168000	2.567000	0.634000
O	5.226000	1.657000	0.087000
C	6.365000	3.782000	0.009000
C	7.321000	4.709000	0.527000
C	11.030000	3.819000	1.837000
O	10.933000	2.973000	0.948000
N	11.606000	3.578000	3.017000
C	12.240000	2.304000	3.377000
C	12.952000	2.446000	4.715000
C	14.279000	3.310000	4.616000
C	14.839000	3.491000	6.034000
N	14.239000	4.645000	6.694000
C	14.474000	5.036000	7.951000
N	15.313000	4.366000	8.746000
N	13.870000	6.124000	8.407000
C	11.210000	1.191000	3.531000
O	10.143000	1.432000	4.068000
N	11.574000	-0.011000	3.104000
C	10.663000	-1.148000	3.050000
C	10.302000	-1.470000	1.566000
C	9.351000	-0.431000	1.008000
C	11.640000	-1.587000	0.665000
C	11.367000	-2.341000	3.742000
O	12.590000	-2.283000	3.979000
N	10.636000	-3.409000	4.056000
C	11.253000	-4.579000	4.685000
C	11.716000	-4.298000	6.095000
O	11.279000	-3.330000	6.719000
N	12.601000	-5.138000	6.635000
C	13.090000	-4.914000	7.996000
C	14.132000	-5.945000	8.382000
C	13.667000	-3.541000	8.155000
O	13.592000	-2.949000	9.223000
N	14.305000	-3.040000	7.118000
C	15.026000	-1.784000	7.266000
C	15.936000	-1.550000	6.058000
C	17.237000	-2.368000	5.927000
C	17.770000	-2.214000	4.489000
C	18.311000	-2.004000	7.015000
C	14.062000	-0.610000	7.405000
O	14.452000	0.484000	7.839000
N	12.792000	-0.837000	7.034000
C	11.769000	0.194000	7.174000
C	10.750000	-0.104000	8.254000
O	10.139000	0.818000	8.770000
N	10.593000	-1.357000	8.648000
C	9.532000	-1.687000	9.613000
C	8.317000	-2.223000	8.860000
C	7.688000	-1.202000	7.940000
C	6.673000	-0.371000	8.394000
C	6.070000	0.580000	7.552000
C	6.535000	0.721000	6.248000
C	7.551000	-0.096000	5.783000

C	8.119000	-1.068000	6.627000
C	9.905000	-2.590000	10.815000
O	9.056000	-2.861000	11.698000
N	11.178000	-3.012000	10.894000
C	11.661000	-3.761000	12.078000
C	13.202000	-3.774000	12.130000
C	13.869000	-4.624000	13.228000
C	14.275000	-5.948000	12.610000
C	15.107000	-3.928000	13.815000
C	11.158000	-3.087000	13.361000
O	11.251000	-1.880000	13.505000
N	10.648000	-3.868000	14.304000
C	10.124000	-3.287000	15.524000
C	8.612000	-3.029000	15.406000
C	10.456000	-4.045000	16.826000
O	10.280000	-5.258000	16.951000
N	10.948000	-3.266000	17.778000
C	11.183000	-3.702000	19.119000
C	12.676000	-3.569000	19.407000
C	13.414000	-4.721000	20.064000
C	12.703000	-6.050000	19.826000
C	14.812000	-4.715000	19.467000
C	10.422000	-2.629000	19.861000
O	10.981000	-1.556000	20.167000
N	9.123000	-2.885000	20.107000
C	8.452000	-4.184000	19.861000
C	6.968000	-3.818000	19.863000
C	6.918000	-2.633000	20.854000
C	8.181000	-1.835000	20.503000
C	8.682000	-1.022000	21.696000
O	9.153000	-1.607000	22.676000
N	8.610000	0.310000	21.596000
C	8.992000	1.178000	22.704000
C	10.462000	1.570000	22.757000
O	10.893000	2.280000	23.666000
N	11.242000	1.135000	21.769000
C	12.680000	1.400000	21.756000
C	13.398000	0.076000	21.621000
C	14.899000	0.218000	21.719000
O	15.478000	1.222000	21.282000
N	15.546000	-0.796000	22.279000
C	13.075000	2.269000	20.583000
O	13.019000	1.793000	19.453000
N	13.510000	3.528000	20.830000
C	13.847000	4.151000	22.122000
C	14.560000	5.416000	21.717000
C	14.094000	5.770000	20.396000
C	13.785000	4.429000	19.712000
C	14.935000	3.984000	18.798000
O	15.163000	4.585000	17.739000
N	15.654000	2.930000	19.177000
C	16.694000	2.392000	18.280000
C	17.728000	1.522000	19.043000
C	18.504000	2.267000	20.146000
C	19.221000	3.506000	19.615000
O	19.850000	3.424000	18.533000
O	19.149000	4.582000	20.258000
C	16.026000	1.610000	17.143000

O	16.588000	1.482000	16.057000
N	14.816000	1.115000	17.406000
C	14.006000	0.381000	16.405000
C	14.410000	-1.102000	16.347000
C	12.530000	0.503000	16.765000
O	11.939000	-0.422000	17.313000
N	11.932000	1.663000	16.494000
C	12.439000	2.932000	15.944000
C	11.318000	3.912000	16.219000
C	10.384000	3.289000	17.199000
C	10.605000	1.782000	17.091000
C	9.484000	1.130000	16.282000
O	8.423000	0.869000	16.847000
N	9.710000	0.868000	14.997000
C	8.683000	0.374000	14.101000
C	8.227000	1.500000	13.193000
O	8.456000	2.665000	13.490000
N	7.529000	1.166000	12.100000
C	6.881000	2.210000	11.272000
C	5.816000	2.965000	12.089000
C	4.628000	2.133000	12.427000
O	4.427000	1.013000	11.899000
N	3.806000	2.666000	13.344000
C	7.832000	3.258000	10.670000
O	7.403000	4.340000	10.256000
N	9.121000	2.968000	10.595000
C	10.035000	4.011000	10.094000
C	11.525000	3.571000	10.237000
C	11.935000	3.141000	11.655000
S	11.495000	1.479000	12.257000
C	12.564000	0.439000	11.240000
C	9.732000	4.439000	8.664000
O	9.830000	5.616000	8.332000
N	9.403000	3.478000	7.797000
C	8.970000	3.780000	6.416000
C	7.725000	4.661000	6.296000
O	7.589000	5.463000	5.362000
N	6.817000	4.543000	7.264000
C	5.640000	5.450000	7.330000
C	4.611000	4.896000	8.319000
C	3.773000	3.716000	7.761000
C	2.976000	3.101000	8.840000
C	2.858000	4.218000	6.671000
C	6.102000	6.879000	7.751000
O	5.540000	7.879000	7.273000
N	7.091000	6.971000	8.667000
C	7.680000	8.293000	9.007000
C	8.496000	8.235000	10.303000
C	7.646000	8.219000	11.559000
C	7.581000	7.089000	12.350000
C	6.797000	7.080000	13.506000
C	6.095000	8.210000	13.882000
C	6.175000	9.341000	13.128000
C	6.957000	9.337000	11.952000
C	8.501000	8.903000	7.864000
O	8.615000	10.116000	7.754000
N	9.057000	8.068000	6.980000
C	9.716000	8.572000	5.763000

C	10.345000	7.407000	4.960000
C	11.567000	6.760000	5.675000
O	12.049000	7.293000	6.689000
O	12.008000	5.672000	5.235000
C	8.674000	9.306000	4.881000
O	8.894000	10.456000	4.442000
N	7.571000	8.602000	4.569000
C	6.421000	9.189000	3.900000
C	5.239000	8.206000	3.922000
C	5.523000	6.874000	3.203000
C	4.330000	5.957000	3.310000
O	3.253000	6.453000	3.625000
N	4.481000	4.654000	3.017000
C	6.012000	10.510000	4.562000
O	5.867000	11.524000	3.881000
N	5.839000	10.500000	5.871000
C	5.351000	11.692000	6.587000
C	5.239000	11.429000	8.061000
C	4.277000	12.389000	8.755000
C	4.028000	11.996000	10.169000
O	2.972000	11.420000	10.531000
N	4.996000	12.253000	10.990000
C	6.259000	12.854000	6.433000
O	5.821000	13.990000	6.351000
N	7.551000	12.589000	6.437000
C	8.510000	13.688000	6.358000
C	9.903000	13.216000	6.816000
C	11.048000	14.242000	6.770000
C	10.741000	15.418000	7.713000
C	12.300000	13.528000	7.220000
C	8.544000	14.220000	4.941000
O	8.696000	15.410000	4.728000
N	8.338000	13.362000	3.948000
C	8.181000	13.862000	2.591000
C	8.055000	12.744000	1.609000
C	6.993000	14.781000	2.456000
O	7.054000	15.762000	1.718000
N	5.879000	14.437000	3.112000
C	4.646000	15.283000	3.119000
C	3.546000	14.613000	3.951000
C	3.048000	13.211000	3.561000
C	1.708000	12.876000	4.248000
C	2.937000	13.100000	2.057000
C	4.954000	16.649000	3.754000
O	4.473000	17.691000	3.313000
N	5.755000	16.640000	4.806000
C	6.193000	17.880000	5.431000
C	7.098000	17.511000	6.581000
C	6.722000	18.039000	7.925000
C	7.671000	17.429000	8.943000
O	8.862000	17.794000	8.967000
N	7.174000	16.454000	9.753000
C	7.020000	18.734000	4.463000
O	6.863000	19.971000	4.401000
N	7.901000	18.076000	3.705000
C	8.740000	18.748000	2.698000
C	9.659000	17.737000	1.995000
C	10.662000	18.382000	1.047000

C	11.983000	18.694000	1.319000
N	12.566000	19.292000	0.221000
C	11.636000	19.379000	-0.785000
C	11.759000	19.900000	-2.076000
C	10.653000	19.864000	-2.894000
C	9.424000	19.293000	-2.451000
C	9.307000	18.769000	-1.160000
C	10.422000	18.820000	-0.300000
C	7.832000	19.459000	1.693000
O	8.105000	20.581000	1.273000
N	6.725000	18.814000	1.306000
C	5.783000	19.455000	0.389000
C	4.678000	18.449000	-0.113000
C	3.587000	19.136000	-0.849000
C	5.296000	17.427000	-1.027000
C	5.174000	20.680000	1.074000
O	5.117000	21.741000	0.491000
N	4.730000	20.548000	2.318000
C	4.143000	21.685000	3.035000
C	3.760000	21.270000	4.459000
C	2.498000	20.413000	4.565000
C	1.313000	21.061000	3.893000
O	0.810000	22.072000	4.368000
N	0.907000	20.537000	2.745000
C	5.125000	22.898000	3.031000
O	4.756000	24.013000	2.664000
N	6.376000	22.682000	3.418000
C	7.356000	23.788000	3.446000
C	8.553000	23.393000	4.273000
C	8.182000	22.815000	5.589000
C	9.406000	22.748000	6.498000
C	9.210000	21.722000	7.617000
N	10.538000	21.352000	8.201000
C	7.865000	24.287000	2.094000
O	8.239000	25.451000	1.975000
N	7.886000	23.436000	1.069000
C	8.593000	23.785000	-0.161000
C	9.708000	22.771000	-0.411000
C	10.764000	22.811000	0.668000
O	11.553000	23.750000	0.738000
N	10.763000	21.821000	1.538000
C	7.791000	23.929000	-1.434000
O	8.279000	24.496000	-2.399000
N	6.576000	23.403000	-1.476000
C	5.901000	23.308000	-2.793000
C	4.808000	22.177000	-2.789000
C	3.500000	22.654000	-2.140000
C	4.670000	21.547000	-4.171000
C	5.801000	20.603000	-4.461000
C	5.391000	24.648000	-3.391000
O	5.162000	24.755000	-4.589000
N	5.197000	25.662000	-2.555000
C	4.831000	26.994000	-3.052000
C	4.616000	27.964000	-1.881000
C	5.915000	27.536000	-3.988000
O	5.604000	28.172000	-4.978000
N	7.179000	27.240000	-3.709000
C	8.285000	27.622000	-4.622000

C	9.604000	27.255000	-3.999000
C	8.207000	27.064000	-6.060000
O	8.676000	27.709000	-7.021000
N	7.599000	25.881000	-6.232000
C	7.439000	25.255000	-7.554000
C	7.502000	23.712000	-7.437000
C	8.803000	23.183000	-6.853000
C	9.080000	23.323000	-5.503000
C	10.267000	22.829000	-4.969000
C	11.222000	22.213000	-5.816000
C	10.972000	22.113000	-7.152000
C	9.743000	22.573000	-7.670000
C	6.093000	25.624000	-8.161000
O	5.755000	25.182000	-9.243000
N	5.302000	26.419000	-7.460000
C	3.990000	26.816000	-7.989000
C	2.794000	26.079000	-7.375000
O	1.643000	26.221000	-7.847000
N	3.044000	25.308000	-6.318000
C	2.026000	24.424000	-5.769000
C	1.268000	25.059000	-4.638000
O	1.789000	25.923000	-3.977000
N	0.034000	24.608000	-4.406000
C	-0.789000	25.034000	-3.262000
C	-2.243000	25.113000	-3.752000
C	-3.181000	25.710000	-2.728000
O	-2.767000	26.085000	-1.649000
N	-4.453000	25.798000	-3.072000
C	-0.733000	24.059000	-2.080000
O	-1.419000	23.039000	-2.097000
N	0.064000	24.358000	-1.035000
C	0.832000	25.561000	-0.671000
C	1.109000	25.359000	0.787000
C	1.102000	23.906000	1.020000
C	0.088000	23.343000	0.024000
C	-1.271000	23.079000	0.742000
O	-1.386000	22.115000	1.505000
N	-2.279000	23.923000	0.495000
C	-3.574000	23.786000	1.143000
C	-4.230000	25.151000	1.346000
C	-3.572000	26.011000	2.433000
C	-4.393000	27.278000	2.701000
C	-3.487000	28.399000	3.266000
N	-4.224000	29.646000	3.755000
C	-4.482000	22.941000	0.263000
O	-5.646000	22.763000	0.597000
N	-3.946000	22.464000	-0.861000
C	-4.697000	21.577000	-1.741000
C	-5.169000	22.341000	-2.977000
O	-5.970000	21.541000	-3.859000
C	-3.802000	20.411000	-2.122000
O	-3.290000	20.352000	-3.229000
N	-3.647000	19.458000	-1.201000
C	-2.804000	18.311000	-1.414000
C	-1.646000	18.333000	-0.394000
C	-0.876000	16.988000	-0.397000
C	-0.677000	19.466000	-0.835000
C	-3.603000	17.018000	-1.288000

O	-4.175000	16.774000	-0.238000
N	-3.597000	16.187000	-2.341000
C	-4.232000	14.841000	-2.303000
C	-5.158000	14.734000	-3.515000
C	-5.829000	13.402000	-3.613000
O	-6.160000	15.762000	-3.383000
C	-3.228000	13.685000	-2.328000
O	-2.408000	13.605000	-3.246000
N	-3.280000	12.788000	-1.335000
C	-2.454000	11.604000	-1.338000
C	-2.342000	11.003000	0.064000
C	-1.913000	11.940000	1.219000
C	-1.730000	11.165000	2.511000
C	-0.666000	12.718000	0.830000
C	-3.161000	10.594000	-2.196000
O	-4.373000	10.491000	-2.085000
N	-2.414000	9.827000	-3.007000
C	-2.978000	8.654000	-3.715000
C	-3.623000	9.018000	-5.093000
C	-2.665000	9.397000	-6.225000
C	-1.640000	10.307000	-6.058000
C	-0.811000	10.639000	-7.148000
C	-1.010000	10.112000	-8.389000
C	-2.080000	9.202000	-8.573000
C	-2.877000	8.879000	-7.524000
C	-2.005000	7.497000	-3.714000
O	-0.779000	7.752000	-3.566000
N	-2.499000	6.236000	-3.817000
C	-1.629000	5.070000	-3.646000
C	-2.322000	3.788000	-3.992000
O	-3.577000	3.746000	-4.058000
N	-1.552000	2.737000	-4.279000
C	-2.175000	1.458000	-4.606000
C	-2.040000	1.175000	-6.117000
C	-2.718000	-0.105000	-6.676000
C	-1.808000	-1.328000	-6.694000
O	-0.625000	-1.174000	-6.288000
O	-2.243000	-2.433000	-7.132000
C	-1.667000	0.332000	-3.695000
O	-0.506000	0.344000	-3.280000
N	-2.551000	-0.602000	-3.294000
C	-2.124000	-1.755000	-2.456000
C	-1.216000	-2.663000	-3.282000
O	-1.171000	-3.990000	-2.757000
C	-1.447000	-1.300000	-1.123000
O	-2.030000	-0.501000	-0.350000
N	-0.229000	-1.750000	-0.806000
C	0.418000	-1.197000	0.402000
C	1.844000	-1.761000	0.590000
C	0.446000	0.321000	0.404000
O	0.468000	0.978000	1.492000
N	0.525000	0.927000	-0.774000
C	0.528000	2.405000	-0.812000
C	-0.850000	2.981000	-0.410000
O	-0.955000	4.081000	0.180000
N	-1.904000	2.228000	-0.709000
C	-3.286000	2.642000	-0.332000
C	-4.360000	1.786000	-1.078000

C	-3.425000	2.459000	1.162000
O	-3.987000	3.326000	1.852000
N	-2.914000	1.321000	1.660000
C	-2.973000	1.056000	3.077000
C	-2.367000	-0.262000	3.413000
C	-2.258000	2.179000	3.794000
O	-2.789000	2.689000	4.791000
N	-1.086000	2.592000	3.296000
C	-0.382000	3.749000	3.886000
C	0.918000	4.079000	3.093000
O	1.794000	2.948000	3.003000
C	-1.262000	5.001000	3.902000
O	-1.332000	5.691000	4.900000
N	-1.900000	5.335000	2.775000
C	-2.750000	6.534000	2.672000
C	-3.488000	6.650000	1.254000
C	-4.652000	7.652000	1.323000
C	-2.529000	7.049000	0.118000
C	-3.813000	6.506000	3.788000
O	-4.030000	7.497000	4.491000
N	-4.393000	5.339000	4.006000
C	-5.441000	5.169000	5.035000
C	-6.157000	3.833000	4.836000
O	-5.369000	2.760000	5.288000
C	-4.927000	5.318000	6.480000
O	-5.655000	5.774000	7.331000
N	-3.661000	5.006000	6.738000
C	-3.083000	5.204000	8.096000
C	-1.817000	4.321000	8.271000
C	-2.086000	2.813000	8.356000
C	-0.819000	2.015000	8.178000
C	-2.808000	2.386000	9.641000
C	-2.709000	6.651000	8.306000
O	-2.722000	7.149000	9.426000
N	-2.337000	7.346000	7.238000
C	-2.168000	8.807000	7.351000
C	-1.623000	9.389000	6.045000
C	-0.157000	9.110000	5.815000
N	0.860000	9.749000	6.511000
C	2.030000	9.297000	6.090000
N	1.818000	8.387000	5.150000
C	0.459000	8.255000	4.959000
C	-3.507000	9.498000	7.743000
O	-3.515000	10.575000	8.363000
N	-4.642000	8.885000	7.377000
C	-5.947000	9.406000	7.810000
C	-7.096000	8.737000	7.045000
C	-7.278000	9.220000	5.604000
C	-8.219000	8.233000	4.866000
C	-7.855000	10.611000	5.527000
C	-6.141000	9.246000	9.314000
O	-6.865000	10.029000	9.944000
N	-5.518000	8.231000	9.897000
C	-5.602000	7.989000	11.334000
C	-5.517000	6.490000	11.628000
C	-6.492000	5.534000	10.932000
C	-6.410000	4.144000	11.470000
C	-7.862000	5.999000	11.098000

C	-4.492000	8.664000	12.142000
O	-4.597000	8.765000	13.357000
N	-3.412000	9.093000	11.503000
C	-2.257000	9.532000	12.278000
C	-0.923000	9.242000	11.557000
O	0.178000	9.742000	12.366000
C	-2.309000	10.994000	12.667000
O	-2.300000	11.878000	11.808000
N	-2.292000	11.279000	13.976000
C	-2.241000	10.431000	15.177000
C	-1.697000	11.395000	16.202000
C	-2.316000	12.707000	15.837000
C	-2.362000	12.698000	14.309000
C	-1.248000	13.561000	13.669000
O	-1.514000	14.699000	13.268000
N	-0.036000	13.039000	13.519000
C	1.050000	13.852000	12.958000
C	0.907000	14.153000	11.488000
O	1.499000	15.086000	11.016000
N	0.115000	13.362000	10.759000
C	-0.200000	13.611000	9.342000
C	-0.386000	12.271000	8.621000
O	0.795000	11.489000	8.737000
C	-1.439000	14.493000	9.050000
O	-1.702000	14.858000	7.896000
N	-2.206000	14.817000	10.077000
C	-3.526000	15.426000	9.912000
C	-4.187000	15.663000	11.294000
C	-5.526000	16.336000	11.232000
N	-6.704000	15.676000	11.512000
C	-7.722000	16.509000	11.375000
N	-7.246000	17.691000	11.028000
C	-5.876000	17.613000	10.945000
C	-3.436000	16.705000	9.119000
O	-4.259000	16.956000	8.261000
N	-2.424000	17.531000	9.386000
C	-2.397000	18.821000	8.730000
C	-2.121000	19.929000	9.755000
O	-0.730000	19.953000	10.040000
C	-1.429000	18.923000	7.528000
O	-1.202000	20.003000	7.043000
N	-0.925000	17.784000	7.048000
C	-0.053000	17.669000	5.892000
C	0.997000	16.585000	6.162000
C	1.811000	16.740000	7.436000
C	2.976000	15.754000	7.450000
C	2.301000	18.189000	7.471000
C	-0.775000	17.301000	4.577000
O	-0.113000	17.068000	3.547000
N	-2.106000	17.202000	4.594000
C	-2.827000	16.930000	3.326000
C	-2.598000	15.476000	2.870000
C	-3.274000	14.407000	3.729000
C	-2.817000	14.105000	4.982000
C	-3.414000	13.155000	5.753000
C	-4.488000	12.430000	5.234000
C	-4.947000	12.718000	3.971000
C	-4.354000	13.669000	3.229000

C	-4.320000	17.286000	3.407000
O	-4.800000	17.605000	4.481000
N	-5.014000	17.302000	2.274000
C	-6.428000	17.690000	2.220000
C	-6.646000	18.610000	1.030000
C	-8.075000	19.210000	1.019000
O	-5.668000	19.685000	1.045000
C	-7.354000	16.438000	2.021000
O	-8.320000	16.241000	2.776000
N	-7.054000	15.625000	1.010000
C	-7.973000	14.528000	0.597000
C	-8.728000	14.964000	-0.686000
C	-9.984000	15.719000	-0.392000
C	-10.874000	15.941000	-1.583000
N	-10.386000	17.082000	-2.290000
C	-10.571000	18.321000	-1.868000
N	-10.045000	19.324000	-2.545000
N	-11.313000	18.544000	-0.790000
C	-7.182000	13.235000	0.337000
O	-5.968000	13.270000	0.309000
N	-7.862000	12.101000	0.111000
C	-7.157000	10.850000	-0.054000
C	-6.988000	10.183000	1.319000
C	-7.876000	9.923000	-1.059000
O	-9.154000	9.896000	-1.128000
N	-7.061000	9.215000	-1.858000
C	-7.506000	8.219000	-2.873000
C	-7.073000	8.625000	-4.337000
C	-7.426000	7.517000	-5.402000
C	-7.559000	10.045000	-4.700000
C	-7.289000	10.464000	-6.126000
C	-6.852000	6.891000	-2.489000
O	-5.582000	6.810000	-2.307000
N	-7.668000	5.848000	-2.373000
C	-7.215000	4.499000	-2.047000
C	-7.942000	3.987000	-0.782000
C	-7.558000	4.632000	0.551000
C	-7.952000	6.088000	0.567000
C	-8.214000	3.933000	1.704000
C	-7.473000	3.466000	-3.143000
O	-8.599000	2.972000	-3.273000
N	-6.434000	3.092000	-3.909000
C	-6.564000	2.070000	-4.947000
C	-5.765000	2.487000	-6.191000
C	-6.092000	3.934000	-6.640000
C	-5.171000	4.564000	-7.731000
O	-5.193000	5.767000	-7.899000
N	-4.408000	3.758000	-8.473000
C	-6.155000	0.661000	-4.467000
O	-4.949000	0.368000	-4.124000
N	-7.134000	-0.254000	-4.472000
C	-6.901000	-1.625000	-4.000000
C	-6.181000	-2.478000	-5.062000
O	-6.778000	-2.361000	-6.346000
C	-6.117000	-1.759000	-2.687000
O	-5.082000	-2.440000	-2.658000
N	-6.543000	-1.108000	-1.613000
C	-5.919000	-1.419000	-0.340000

C	-6.464000	-0.523000	0.743000
O	-6.891000	0.575000	0.432000
N	-6.447000	-0.972000	1.991000
C	-6.764000	-0.095000	3.133000
C	-8.291000	-0.017000	3.294000
O	-8.837000	-1.338000	3.227000
C	-6.164000	-0.778000	4.344000
O	-5.904000	-1.980000	4.262000
N	-5.974000	-0.063000	5.465000
C	-5.275000	-0.663000	6.631000
C	-4.980000	0.396000	7.717000
C	-6.205000	0.792000	8.498000
C	-6.838000	2.008000	8.268000
C	-7.977000	2.337000	8.945000
C	-8.517000	1.440000	9.842000
C	-7.914000	0.229000	10.076000
C	-6.772000	-0.100000	9.397000
C	-6.021000	-1.845000	7.287000
O	-5.417000	-2.656000	7.941000
N	-7.332000	-1.940000	7.140000
C	-8.068000	-3.095000	7.672000
C	-9.569000	-2.791000	7.886000
C	-10.246000	-2.304000	6.622000
O	-9.647000	-1.551000	5.855000
N	-11.486000	-2.730000	6.388000
C	-7.912000	-4.344000	6.825000
O	-8.559000	-5.374000	7.096000
N	-7.077000	-4.307000	5.796000
C	-6.837000	-5.555000	5.025000
C	-6.053000	-5.248000	3.767000
C	-6.094000	-6.555000	5.942000
O	-5.389000	-6.139000	6.851000
N	-6.279000	-7.873000	5.757000
C	-7.130000	-8.585000	4.790000
C	-6.772000	-10.033000	5.004000
C	-6.173000	-10.162000	6.377000
C	-5.659000	-8.774000	6.751000
C	-4.108000	-8.792000	6.729000
O	-3.491000	-9.198000	7.716000
N	-3.502000	-8.338000	5.637000
C	-2.033000	-8.252000	5.527000
C	-1.619000	-8.478000	4.071000
C	-2.474000	-7.680000	3.077000
C	-3.565000	-8.142000	2.378000
N	-4.099000	-7.133000	1.604000
C	-3.346000	-5.995000	1.786000
C	-3.463000	-4.713000	1.192000
C	-2.571000	-3.731000	1.572000
C	-1.546000	-4.004000	2.477000
C	-1.408000	-5.290000	3.048000
C	-2.320000	-6.304000	2.702000
C	-1.425000	-6.936000	6.007000
O	-0.210000	-6.805000	5.973000
N	-2.217000	-5.941000	6.417000
C	-1.665000	-4.567000	6.527000
C	-2.679000	-3.526000	6.095000
C	-1.035000	-4.111000	7.835000
O	-0.269000	-3.137000	7.837000

N	-1.406000	-4.714000	8.959000
C	-0.864000	-4.273000	10.236000
C	-1.931000	-3.493000	11.055000
C	-1.376000	-3.080000	12.440000
C	-2.439000	-2.260000	10.268000
C	-0.395000	-5.495000	11.036000
O	-1.134000	-6.472000	11.176000
N	0.835000	-5.464000	11.527000
C	1.398000	-6.564000	12.347000
C	2.964000	-6.678000	12.173000
C	3.521000	-7.991000	12.731000
O	3.334000	-6.563000	10.787000
C	1.114000	-6.298000	13.829000
O	1.311000	-5.180000	14.315000
N	0.667000	-7.320000	14.559000
C	0.410000	-7.165000	16.006000
C	-0.338000	-8.370000	16.565000
O	0.580000	-9.430000	16.739000
C	1.723000	-7.053000	16.729000
O	2.751000	-7.460000	16.209000
N	1.708000	-6.486000	17.923000
C	2.956000	-6.310000	18.685000
C	2.692000	-5.523000	19.960000
C	3.227000	-4.098000	19.911000
C	3.557000	-3.645000	18.473000
C	2.254000	-3.154000	20.599000
C	3.678000	-7.637000	18.996000
O	4.897000	-7.701000	18.969000
N	2.913000	-8.686000	19.278000
C	3.476000	-10.015000	19.494000
C	2.419000	-11.041000	19.939000
C	3.042000	-12.356000	20.393000
C	3.925000	-12.391000	21.470000
C	4.503000	-13.575000	21.889000
C	4.204000	-14.750000	21.233000
O	4.780000	-15.935000	21.663000
C	3.335000	-14.746000	20.155000
C	2.759000	-13.552000	19.740000
C	4.221000	-10.558000	18.282000
O	5.375000	-10.948000	18.417000
N	3.580000	-10.617000	17.117000
C	4.293000	-11.086000	15.925000
C	3.409000	-11.060000	14.680000
C	1.981000	-11.367000	14.926000
C	1.110000	-11.001000	13.732000
O	0.877000	-9.782000	13.502000
O	0.651000	-11.941000	13.034000
C	5.506000	-10.218000	15.629000
O	6.533000	-10.709000	15.206000
N	5.362000	-8.913000	15.789000
C	6.458000	-8.012000	15.500000
C	6.058000	-6.557000	15.745000
C	7.723000	-8.361000	16.283000
O	8.794000	-8.424000	15.702000
N	7.599000	-8.564000	17.598000
C	8.756000	-8.920000	18.442000
C	8.429000	-8.832000	19.934000
C	8.007000	-7.416000	20.403000

C	8.620000	-6.938000	21.780000
N	8.824000	-8.031000	22.727000
C	10.011000	-8.403000	23.194000
N	11.101000	-7.742000	22.823000
N	10.103000	-9.428000	24.035000
C	9.263000	-10.311000	18.107000
O	10.451000	-10.498000	17.949000
N	8.366000	-11.281000	17.967000
C	8.784000	-12.613000	17.570000
C	7.576000	-13.547000	17.415000
C	7.922000	-15.058000	17.633000
O	8.313000	-15.465000	18.732000
N	7.747000	-15.880000	16.574000
C	9.608000	-12.542000	16.271000
O	10.598000	-13.279000	16.104000
N	9.228000	-11.647000	15.355000
C	9.898000	-11.578000	14.067000
C	9.017000	-10.904000	13.021000
C	7.919000	-11.804000	12.523000
C	6.829000	-11.020000	11.808000
N	5.611000	-11.822000	11.652000
C	4.568000	-11.453000	10.924000
N	4.567000	-10.300000	10.272000
N	3.524000	-12.245000	10.847000
C	11.244000	-10.892000	14.182000
O	12.201000	-11.287000	13.535000
N	11.312000	-9.867000	15.015000
C	12.560000	-9.148000	15.256000
C	12.316000	-7.954000	16.179000
C	13.622000	-7.263000	16.524000
O	11.419000	-7.025000	15.556000
C	13.650000	-10.044000	15.888000
O	14.806000	-9.977000	15.501000
N	13.281000	-10.874000	16.859000
C	14.249000	-11.791000	17.504000
C	13.706000	-12.362000	18.804000
C	13.058000	-11.391000	19.772000
C	12.410000	-12.198000	20.896000
C	14.067000	-10.357000	20.262000
C	14.567000	-12.958000	16.598000
O	15.725000	-13.370000	16.479000
N	13.537000	-13.501000	15.965000
C	13.746000	-14.460000	14.894000
C	12.416000	-14.808000	14.234000
C	11.628000	-15.858000	15.015000
O	12.103000	-16.373000	16.026000
N	10.436000	-16.193000	14.530000
C	14.770000	-14.002000	13.843000
O	15.520000	-14.811000	13.323000
N	14.793000	-12.724000	13.500000
C	15.780000	-12.243000	12.535000
C	15.379000	-10.875000	11.976000
C	16.345000	-10.227000	10.992000
C	16.308000	-10.966000	9.679000
C	16.031000	-8.751000	10.826000
C	17.165000	-12.170000	13.210000
O	18.184000	-12.504000	12.593000
N	17.179000	-11.733000	14.474000

C	18.365000	-11.729000	15.326000
C	17.988000	-11.348000	16.729000
C	18.951000	-13.116000	15.335000
O	20.054000	-13.341000	14.834000
N	18.203000	-14.063000	15.887000
C	18.655000	-15.446000	15.852000
C	17.534000	-16.426000	16.175000
C	17.968000	-17.880000	16.030000
C	17.345000	-18.782000	17.105000
C	18.176000	-20.071000	17.321000
N	18.156000	-20.547000	18.767000
C	19.333000	-15.798000	14.528000
O	20.513000	-16.141000	14.518000
N	18.607000	-15.683000	13.419000
C	19.111000	-16.054000	12.080000
C	18.000000	-15.913000	11.035000
C	16.870000	-16.936000	11.066000
C	15.580000	-16.335000	10.517000
C	17.277000	-18.157000	10.271000
C	20.341000	-15.292000	11.580000
O	20.968000	-15.691000	10.606000
N	20.684000	-14.183000	12.207000
C	21.843000	-13.440000	11.746000
C	21.522000	-11.946000	11.543000
C	20.689000	-11.730000	10.297000
O	20.834000	-11.449000	12.700000
C	22.922000	-13.542000	12.808000
O	23.909000	-12.791000	12.797000
N	22.711000	-14.457000	13.745000
C	23.595000	-14.577000	14.889000
C	23.648000	-13.339000	15.766000
O	24.679000	-13.044000	16.368000
N	22.543000	-12.620000	15.881000
C	22.574000	-11.384000	16.628000
C	21.989000	-10.218000	15.821000
S	23.146000	-9.352000	14.679000
C	21.861000	-11.523000	17.945000
O	21.749000	-10.545000	18.689000
N	21.387000	-12.723000	18.266000
C	20.710000	-12.877000	19.560000
C	20.319000	-14.327000	19.854000
O	19.238000	-14.754000	19.041000
C	21.550000	-12.301000	20.709000
O	22.754000	-12.530000	20.795000
N	20.902000	-11.528000	21.572000
C	21.529000	-10.916000	22.741000
C	21.839000	-9.441000	22.496000
C	23.300000	-9.144000	22.217000
C	23.935000	-10.280000	21.457000
N	25.098000	-9.856000	20.685000
C	25.639000	-10.597000	19.728000
N	26.683000	-10.166000	19.039000
N	25.108000	-11.771000	19.445000
C	20.540000	-11.010000	23.878000
O	19.495000	-11.654000	23.756000
N	20.852000	-10.341000	24.978000
C	19.965000	-10.343000	26.126000
C	20.710000	-10.866000	27.366000

C	22.084000	-10.175000	27.669000
C	23.121000	-10.247000	26.514000
O	23.542000	-9.162000	26.033000
O	23.524000	-11.371000	26.100000
C	19.419000	-8.928000	26.331000
O	18.235000	-8.730000	26.612000
N	20.300000	-7.947000	26.177000
C	19.939000	-6.548000	26.224000
C	21.174000	-5.757000	26.656000
C	20.933000	-4.266000	26.743000
O	20.224000	-3.690000	25.909000
N	21.556000	-3.624000	27.748000
C	19.487000	-6.192000	24.805000
O	20.219000	-6.437000	23.832000
N	18.272000	-5.659000	24.677000
C	17.666000	-5.444000	23.354000
C	16.178000	-5.078000	23.465000
C	15.298000	-6.262000	23.868000
C	13.820000	-5.916000	23.942000
O	13.465000	-4.723000	23.836000
O	13.011000	-6.855000	24.110000
C	18.396000	-4.420000	22.508000
O	18.391000	-4.520000	21.284000
N	19.031000	-3.441000	23.150000
C	19.771000	-2.420000	22.407000
C	20.134000	-1.216000	23.285000
C	20.748000	-0.103000	22.437000
O	18.958000	-0.729000	23.939000
C	21.024000	-3.020000	21.819000
O	21.630000	-2.442000	20.927000
N	21.394000	-4.190000	22.332000
C	22.564000	-4.926000	21.871000
C	23.026000	-5.931000	22.923000
C	23.009000	-5.420000	24.347000
C	24.385000	-5.072000	24.864000
O	24.902000	-5.831000	25.721000
O	24.947000	-4.044000	24.414000
C	22.188000	-5.675000	20.599000
O	22.979000	-5.779000	19.664000
N	20.977000	-6.206000	20.548000
C	20.546000	-6.822000	19.306000
C	19.142000	-7.433000	19.393000
C	18.804000	-8.123000	18.094000
C	19.063000	-8.458000	20.503000
C	17.739000	-9.145000	20.532000
C	20.557000	-5.795000	18.187000
O	21.096000	-6.051000	17.127000
N	19.968000	-4.627000	18.421000
C	19.905000	-3.605000	17.372000
C	19.088000	-2.337000	17.749000
C	19.037000	-1.392000	16.569000
C	17.655000	-2.689000	18.097000
C	17.010000	-3.677000	17.096000
C	21.295000	-3.171000	16.952000
O	21.569000	-2.982000	15.767000
N	22.162000	-2.984000	17.943000
C	23.523000	-2.572000	17.685000
C	24.248000	-2.367000	19.005000

C	25.223000	-1.239000	18.966000
C	26.557000	-1.678000	18.351000
C	27.467000	-2.346000	19.394000
N	27.897000	-1.372000	20.458000
C	24.206000	-3.626000	16.805000
O	24.873000	-3.296000	15.819000
N	24.005000	-4.892000	17.142000
C	24.502000	-5.965000	16.317000
C	24.066000	-7.313000	16.887000
S	24.680000	-8.725000	15.930000
C	23.969000	-5.874000	14.890000
O	24.724000	-5.966000	13.915000
N	22.649000	-5.729000	14.771000
C	22.000000	-5.779000	13.473000
C	20.479000	-5.835000	13.638000
C	19.876000	-7.227000	13.917000
C	18.442000	-7.110000	14.409000
C	19.947000	-8.157000	12.686000
C	22.456000	-4.617000	12.613000
O	22.426000	-4.693000	11.406000
N	22.931000	-3.554000	13.237000
C	23.422000	-2.430000	12.483000
C	23.509000	-1.212000	13.380000
C	22.173000	-0.592000	13.724000
C	22.319000	0.890000	13.973000
N	21.654000	1.159000	15.226000
C	22.260000	1.121000	16.410000
N	21.552000	1.349000	17.509000
N	23.573000	0.871000	16.499000
C	24.796000	-2.656000	11.886000
O	25.308000	-1.790000	11.196000
N	25.422000	-3.786000	12.202000
C	26.718000	-4.146000	11.627000
C	27.657000	-4.683000	12.697000
C	28.014000	-3.641000	13.702000
O	28.490000	-2.560000	13.339000
N	27.754000	-3.928000	14.981000
C	26.572000	-5.191000	10.565000
O	27.464000	-5.356000	9.739000
N	25.460000	-5.921000	10.586000
C	25.172000	-6.820000	9.480000
C	23.885000	-7.622000	9.696000
C	23.897000	-8.473000	10.952000
C	25.221000	-9.208000	11.134000
C	25.125000	-10.704000	10.823000
N	26.447000	-11.391000	11.064000
C	25.073000	-5.974000	8.238000
O	24.816000	-4.777000	8.296000
N	25.305000	-6.565000	7.086000
C	25.139000	-5.740000	5.941000
C	26.440000	-5.629000	5.155000
C	26.591000	-6.705000	4.114000
O	25.748000	-6.752000	3.173000
O	27.590000	-7.478000	4.222000
C	23.894000	-6.125000	5.125000
O	23.412000	-7.263000	5.177000
N	23.364000	-5.154000	4.386000
C	24.030000	-3.894000	4.019000

C	22.991000	-3.158000	3.231000
C	22.059000	-4.201000	2.730000
C	22.028000	-5.233000	3.854000
C	21.666000	-6.617000	3.339000
O	20.557000	-7.092000	3.613000
N	22.574000	-7.262000	2.601000
C	22.256000	-8.520000	1.943000
C	23.348000	-8.938000	0.965000
C	23.058000	-8.552000	-0.453000
C	21.784000	-9.197000	-0.991000
O	21.385000	-10.292000	-0.574000
N	21.134000	-8.509000	-1.918000
C	21.999000	-9.613000	2.943000
O	21.221000	-10.522000	2.668000
N	22.632000	-9.520000	4.114000
C	22.466000	-10.523000	5.178000
C	23.491000	-10.331000	6.307000
C	24.950000	-10.666000	5.982000
C	25.928000	-10.222000	7.111000
O	26.019000	-10.924000	8.157000
O	26.596000	-9.169000	6.949000
C	21.080000	-10.431000	5.809000
O	20.463000	-11.442000	6.158000
N	20.630000	-9.196000	6.003000
C	19.285000	-8.904000	6.476000
C	19.200000	-7.432000	6.846000
C	17.757000	-6.985000	6.982000
C	19.948000	-7.211000	8.165000
C	20.437000	-5.785000	8.400000
C	18.229000	-9.292000	5.437000
O	17.293000	-10.010000	5.752000
N	18.397000	-8.874000	4.188000
C	17.417000	-9.189000	3.156000
C	17.831000	-8.577000	1.821000
C	17.755000	-7.063000	1.729000
C	18.442000	-6.558000	0.462000
C	16.279000	-6.612000	1.790000
C	17.253000	-10.693000	2.981000
O	16.125000	-11.209000	2.757000
N	18.393000	-11.396000	3.061000
C	18.431000	-12.835000	2.807000
C	19.880000	-13.329000	2.648000
C	20.352000	-13.560000	1.191000
C	19.371000	-12.985000	0.104000
C	21.821000	-13.119000	0.931000
C	17.692000	-13.622000	3.879000
O	17.180000	-14.700000	3.618000
N	17.614000	-13.086000	5.088000
C	16.858000	-13.760000	6.144000
C	17.665000	-13.714000	7.448000
C	18.912000	-14.552000	7.371000
O	19.981000	-14.053000	7.064000
N	18.766000	-15.838000	7.589000
C	15.391000	-13.298000	6.383000
O	14.688000	-13.859000	7.219000
N	14.948000	-12.272000	5.662000
C	13.627000	-11.718000	5.881000
C	13.381000	-10.514000	4.948000

C	13.987000	-9.209000	5.460000
C	13.689000	-7.982000	4.586000
O	13.132000	-8.104000	3.479000
O	14.047000	-6.854000	5.012000
C	12.558000	-12.789000	5.698000
O	11.634000	-12.890000	6.505000
N	12.686000	-13.597000	4.649000
C	11.646000	-14.562000	4.294000
C	11.973000	-15.221000	3.000000
C	11.367000	-15.613000	5.371000
O	10.263000	-16.112000	5.461000
N	12.349000	-15.920000	6.213000
C	12.187000	-16.980000	7.219000
C	13.405000	-17.918000	7.250000
C	14.152000	-17.987000	5.970000
C	13.647000	-18.693000	4.902000
C	14.330000	-18.746000	3.701000
C	15.518000	-18.104000	3.578000
C	16.036000	-17.400000	4.634000
C	15.355000	-17.346000	5.831000
C	11.968000	-16.505000	8.655000
O	12.051000	-17.323000	9.571000
N	11.726000	-15.220000	8.901000
C	11.421000	-14.838000	10.290000
C	11.518000	-13.319000	10.543000
C	12.919000	-12.827000	10.309000
C	10.551000	-12.574000	9.662000
C	10.032000	-15.349000	10.704000
O	9.524000	-14.999000	11.767000
N	9.448000	-16.194000	9.858000
C	8.065000	-16.649000	9.961000
C	7.158000	-15.755000	9.029000
C	6.489000	-16.559000	7.926000
C	6.158000	-14.920000	9.833000
C	7.981000	-18.158000	9.587000
O	8.557000	-18.593000	8.568000
N	7.274000	-18.965000	10.406000
C	6.279000	-18.523000	11.405000
C	5.431000	-19.757000	11.634000
C	6.330000	-20.929000	11.314000
C	7.257000	-20.424000	10.204000
C	6.690000	-20.817000	8.842000
O	7.283000	-21.618000	8.125000
N	5.538000	-20.256000	8.500000
C	4.957000	-20.446000	7.185000
C	3.802000	-21.451000	7.254000
C	2.914000	-21.241000	8.458000
C	2.562000	-22.304000	9.296000
C	1.742000	-22.087000	10.420000
C	1.285000	-20.794000	10.701000
O	0.483000	-20.528000	11.797000
C	1.636000	-19.740000	9.882000
C	2.445000	-19.963000	8.777000
C	4.477000	-19.090000	6.656000
O	3.971000	-18.257000	7.408000
N	4.650000	-18.854000	5.367000
C	4.170000	-17.621000	4.784000
C	3.296000	-17.940000	3.590000

O	3.009000	-19.102000	3.297000
N	2.869000	-16.917000	2.878000
C	2.039000	-17.162000	1.724000
C	0.554000	-16.949000	2.083000
C	0.188000	-17.722000	3.330000
O	0.343000	-15.562000	2.360000
C	2.506000	-16.157000	0.695000
O	3.368000	-15.327000	1.012000
N	1.958000	-16.229000	-0.537000
C	1.095000	-17.313000	-1.039000
C	1.251000	-17.201000	-2.485000
C	1.497000	-15.724000	-2.764000
C	2.280000	-15.241000	-1.561000
C	1.869000	-13.808000	-1.200000
O	2.205000	-12.878000	-1.930000
N	1.181000	-13.632000	-0.075000
C	0.708000	-12.324000	0.372000
C	-0.801000	-12.355000	0.531000
C	-1.469000	-11.967000	-0.773000
C	-2.785000	-12.672000	-0.960000
C	-1.665000	-10.464000	-0.733000
C	1.335000	-11.892000	1.686000
O	0.899000	-10.930000	2.297000
N	2.380000	-12.591000	2.106000
C	3.056000	-12.286000	3.376000
C	4.202000	-13.271000	3.613000
O	3.715000	-14.435000	4.255000
C	3.616000	-10.878000	3.479000
O	4.284000	-10.408000	2.566000
N	3.386000	-10.240000	4.621000
C	3.986000	-8.961000	4.898000
C	2.922000	-7.884000	5.062000
C	3.494000	-6.644000	5.622000
C	2.250000	-7.608000	3.677000
C	4.814000	-9.145000	6.161000
O	4.378000	-8.837000	7.269000
N	6.017000	-9.676000	6.001000
C	6.797000	-10.051000	7.170000
C	8.078000	-10.777000	6.753000
C	7.788000	-12.177000	6.217000
O	8.491000	-12.691000	5.342000
N	6.724000	-12.791000	6.736000
C	7.089000	-8.869000	8.069000
O	6.997000	-9.012000	9.254000
N	7.447000	-7.717000	7.500000
C	7.742000	-6.508000	8.275000
C	9.208000	-6.084000	8.108000
C	10.166000	-7.054000	8.740000
C	10.423000	-7.006000	10.105000
C	11.276000	-7.965000	10.712000
C	11.850000	-8.977000	9.942000
C	11.590000	-9.044000	8.585000
C	10.729000	-8.091000	7.987000
C	6.822000	-5.379000	7.835000
O	7.016000	-4.826000	6.771000
N	5.842000	-5.029000	8.671000
C	4.840000	-4.002000	8.324000
C	4.498000	-2.988000	9.401000

O	5.104000	-2.945000	10.453000
N	3.528000	-2.134000	9.131000
C	2.906000	-1.954000	7.809000
C	1.916000	-0.833000	8.022000
C	1.713000	-0.675000	9.484000
C	3.017000	-1.190000	10.137000
C	2.698000	-1.892000	11.460000
O	2.283000	-3.066000	11.467000
N	2.894000	-1.207000	12.582000
C	2.539000	-1.787000	13.876000
C	3.766000	-2.333000	14.706000
C	4.225000	-3.631000	14.228000
O	4.849000	-1.393000	14.660000
C	1.989000	-0.667000	14.709000
O	2.241000	0.522000	14.403000
N	1.310000	-1.022000	15.797000
C	0.811000	-0.001000	16.711000
C	-0.374000	-0.546000	17.561000
C	-0.908000	0.502000	18.510000
C	-1.463000	-1.048000	16.634000
C	1.991000	0.392000	17.578000
O	2.365000	-0.345000	18.480000
N	2.605000	1.524000	17.286000
C	3.833000	1.914000	17.970000
C	4.809000	2.459000	16.931000
C	4.234000	3.645000	16.200000
O	2.994000	3.852000	16.313000
O	4.986000	4.389000	15.556000
C	3.629000	2.991000	19.077000
O	4.597000	3.377000	19.749000
N	2.407000	3.493000	19.273000
C	2.202000	4.585000	20.236000
C	2.717000	5.941000	19.758000
O	2.715000	6.914000	20.508000
N	3.129000	6.031000	18.488000
C	3.669000	7.289000	17.929000
C	5.184000	7.146000	17.739000
C	5.858000	8.447000	17.521000
O	5.161000	9.456000	17.404000
O	7.099000	8.475000	17.448000
C	2.962000	7.659000	16.619000
O	2.139000	8.561000	16.633000
N	3.288000	6.973000	15.498000
C	2.571000	7.169000	14.227000
C	3.185000	6.370000	13.065000
C	2.560000	6.677000	11.721000
C	1.476000	5.943000	11.250000
C	0.906000	6.213000	10.017000
C	1.395000	7.245000	9.262000
C	2.470000	8.001000	9.721000
C	3.044000	7.710000	10.936000
C	1.130000	6.700000	14.441000
O	0.200000	7.366000	14.006000
N	0.982000	5.577000	15.131000
C	-0.292000	4.931000	15.396000
C	-0.232000	3.519000	14.872000
C	-1.049000	3.227000	13.640000
C	-1.131000	1.691000	13.384000

C	-2.434000	3.840000	13.854000
C	-0.536000	4.804000	16.895000
O	0.156000	4.031000	17.588000
N	-1.545000	5.499000	17.404000
C	-1.787000	5.506000	18.826000
C	-2.292000	6.862000	19.258000
C	-1.508000	7.960000	18.526000
O	-3.686000	6.985000	18.920000
C	-2.675000	4.415000	19.429000
O	-2.710000	4.296000	20.638000
N	-3.347000	3.583000	18.640000
C	-4.187000	2.526000	19.200000
C	-5.473000	3.163000	19.761000
C	-6.303000	2.221000	20.699000
O	-7.260000	2.746000	21.328000
O	-6.044000	1.001000	20.817000
C	-4.490000	1.580000	18.041000
O	-4.222000	1.902000	16.900000
N	-5.041000	0.412000	18.311000
C	-5.313000	-0.513000	17.228000
C	-5.931000	-1.771000	17.794000
C	-5.002000	-2.339000	18.862000
S	-5.642000	-3.826000	19.644000
C	-6.105000	-4.832000	18.184000
C	-6.176000	0.175000	16.208000
O	-7.108000	0.882000	16.586000
N	-5.825000	0.062000	14.913000
C	-4.615000	-0.468000	14.246000
C	-4.864000	-0.171000	12.796000
C	-5.775000	1.017000	12.737000
C	-6.589000	0.970000	14.062000
C	-8.051000	0.546000	13.841000
O	-8.854000	1.362000	13.482000
N	-8.412000	-0.693000	14.064000
C	-9.816000	-1.055000	13.950000
C	-10.005000	-2.557000	14.200000
C	-9.176000	-3.062000	15.392000
O	-8.073000	-2.503000	15.599000
O	-9.599000	-4.027000	16.100000
C	-10.675000	-0.236000	14.945000
O	-11.834000	0.108000	14.668000
N	-10.091000	0.079000	16.098000
C	-10.783000	0.855000	17.146000
C	-10.088000	0.733000	18.551000
C	-10.927000	1.355000	19.644000
C	-9.891000	-0.739000	18.906000
C	-9.173000	-0.932000	20.241000
C	-10.945000	2.305000	16.748000
O	-12.053000	2.839000	16.873000
N	-9.861000	2.945000	16.274000
C	-9.932000	4.304000	15.707000
C	-8.545000	4.726000	15.158000
C	-7.481000	4.597000	16.258000
C	-6.058000	4.937000	15.757000
C	-7.815000	5.489000	17.448000
C	-11.037000	4.451000	14.611000
O	-11.835000	5.405000	14.620000
N	-11.074000	3.509000	13.676000

C	-12.065000	3.533000	12.600000
C	-11.783000	2.435000	11.561000
C	-12.707000	2.423000	10.313000
C	-12.625000	3.718000	9.548000
C	-12.444000	1.225000	9.383000
C	-13.493000	3.395000	13.140000
O	-14.327000	4.204000	12.843000
N	-13.758000	2.376000	13.942000
C	-15.106000	2.144000	14.526000
C	-15.055000	0.921000	15.435000
C	-16.362000	0.522000	16.057000
C	-17.386000	0.133000	15.039000
O	-18.597000	0.221000	15.341000
O	-16.995000	-0.278000	13.926000
C	-15.600000	3.385000	15.283000
O	-16.776000	3.728000	15.207000
N	-14.699000	4.100000	15.958000
C	-15.112000	5.123000	16.936000
C	-14.408000	4.953000	18.318000
C	-14.800000	3.639000	19.018000
C	-14.148000	3.438000	20.359000
C	-16.349000	3.543000	19.181000
C	-14.873000	6.494000	16.417000
O	-15.013000	7.456000	17.156000
N	-14.502000	6.598000	15.146000
C	-14.594000	7.874000	14.435000
C	-13.398000	8.767000	14.635000
O	-13.494000	9.988000	14.442000
N	-12.254000	8.181000	14.978000
C	-11.092000	8.978000	15.348000
C	-10.431000	8.323000	16.545000
C	-11.100000	8.629000	17.832000
C	-10.604000	9.965000	18.385000
O	-11.408000	10.807000	18.780000
N	-9.262000	10.163000	18.411000
C	-10.109000	9.011000	14.189000
O	-9.081000	8.298000	14.216000
N	-10.460000	9.788000	13.154000
C	-9.657000	9.974000	11.949000
C	-9.964000	8.878000	10.899000
C	-11.427000	8.623000	10.672000
C	-12.173000	9.431000	9.860000
C	-13.542000	9.178000	9.652000
C	-14.151000	8.102000	10.269000
C	-13.406000	7.284000	11.077000
C	-12.057000	7.554000	11.274000
C	-9.943000	11.353000	11.333000
O	-10.906000	12.061000	11.729000
N	-9.133000	11.732000	10.345000
C	-9.275000	13.012000	9.675000
C	-8.108000	13.186000	8.702000
C	-8.051000	14.537000	8.013000
C	-7.065000	14.496000	6.843000
C	-7.078000	15.777000	6.022000
N	-6.554000	16.955000	6.804000
C	-10.590000	13.055000	8.873000
O	-10.858000	12.171000	8.039000
N	-11.400000	14.078000	9.129000

C	-12.685000	14.265000	8.441000
C	-13.650000	15.059000	9.371000
C	-13.851000	14.354000	10.748000
C	-14.715000	13.099000	10.608000
C	-14.338000	11.968000	11.543000
N	-13.804000	12.430000	12.849000
C	-12.457000	14.989000	7.098000
O	-12.052000	16.174000	7.067000
N	-12.633000	14.255000	5.993000
C	-12.392000	14.764000	4.640000
C	-10.883000	14.951000	4.294000
C	-10.185000	13.609000	4.086000
O	-10.787000	15.696000	3.078000
C	-13.035000	13.792000	3.658000
O	-13.630000	12.775000	4.089000
N	-12.957000	14.074000	2.366000
C	-13.583000	13.192000	1.319000
C	-13.857000	13.984000	0.056000
C	-14.805000	15.183000	0.227000
C	-14.115000	16.388000	0.862000
O	-12.990000	16.770000	0.493000
N	-14.788000	16.985000	1.837000
C	-12.602000	12.125000	0.949000
O	-11.386000	12.384000	0.974000
N	-13.093000	10.939000	0.588000
C	-12.238000	9.857000	0.081000
C	-12.063000	8.665000	1.081000
C	-11.263000	9.085000	2.318000
C	-13.412000	8.115000	1.481000
C	-13.385000	6.805000	2.338000
C	-12.743000	9.251000	-1.230000
O	-13.948000	9.206000	-1.495000
N	-11.806000	8.739000	-2.025000
C	-12.121000	8.019000	-3.233000
C	-11.602000	8.835000	-4.440000
C	-11.971000	8.388000	-5.855000
C	-11.416000	9.306000	-6.917000
C	-11.396000	7.039000	-6.091000
C	-11.471000	6.612000	-3.103000
O	-10.215000	6.496000	-2.911000
N	-12.288000	5.554000	-3.169000
C	-11.810000	4.198000	-2.878000
C	-12.458000	3.650000	-1.558000
C	-11.870000	2.345000	-1.160000
C	-12.226000	4.600000	-0.385000
C	-12.196000	3.275000	-4.029000
O	-13.319000	3.405000	-4.585000
N	-11.334000	2.325000	-4.388000
C	-11.726000	1.383000	-5.449000
C	-10.998000	0.053000	-5.427000
O	-9.978000	-0.110000	-4.707000
N	-11.465000	-0.872000	-6.279000
C	-10.856000	-2.168000	-6.478000
C	-11.418000	-3.240000	-5.526000
C	-11.061000	-2.933000	-4.096000
C	-12.960000	-3.311000	-5.722000
C	-11.080000	-2.624000	-7.930000
O	-11.880000	-2.051000	-8.641000

N	-10.321000	-3.651000	-8.347000
C	-10.326000	-4.185000	-9.664000
C	-8.878000	-4.473000	-10.114000
C	-8.077000	-3.176000	-10.257000
O	-8.644000	-2.103000	-10.015000
N	-6.795000	-3.255000	-10.601000
C	-11.099000	-5.477000	-9.642000
O	-11.180000	-6.168000	-8.617000
N	-11.647000	-5.809000	-10.799000
C	-12.471000	-7.025000	-10.918000
C	-12.939000	-7.139000	-12.361000
C	-13.879000	-8.264000	-12.630000
C	-14.402000	-8.112000	-14.046000
C	-15.133000	-9.382000	-14.463000
N	-16.018000	-9.060000	-15.629000
C	-11.789000	-8.307000	-10.495000
O	-12.391000	-9.126000	-9.806000
N	-10.551000	-8.542000	-10.932000
C	-9.872000	-9.775000	-10.540000
C	-9.500000	-10.652000	-11.751000
C	-10.717000	-11.026000	-12.596000
O	-11.431000	-11.960000	-12.194000
O	-10.980000	-10.374000	-13.630000
C	-8.654000	-9.551000	-9.643000
O	-7.581000	-10.085000	-9.889000
N	-8.826000	-8.817000	-8.565000
C	-7.724000	-8.642000	-7.608000
C	-8.288000	-8.050000	-6.317000
C	-8.843000	-6.634000	-6.498000
C	-7.745000	-5.590000	-6.459000
O	-6.569000	-5.976000	-6.360000
O	-8.051000	-4.401000	-6.525000
C	-6.848000	-9.905000	-7.279000
O	-5.594000	-9.825000	-7.166000
N	-7.490000	-11.045000	-7.035000
C	-6.759000	-12.171000	-6.444000
C	-5.986000	-13.090000	-7.406000
O	-5.172000	-13.879000	-6.965000
N	-6.247000	-13.017000	-8.707000
C	-5.603000	-13.908000	-9.670000
C	-6.147000	-13.738000	-11.067000
C	-7.687000	-14.069000	-11.133000
O	-5.924000	-12.397000	-11.518000
C	-4.060000	-13.860000	-9.719000
O	-3.421000	-14.902000	-9.861000
N	-3.462000	-12.673000	-9.580000
C	-2.001000	-12.537000	-9.685000
C	-1.619000	-11.079000	-9.423000
C	-1.255000	-13.468000	-8.717000
O	-0.109000	-13.924000	-8.993000
N	-1.871000	-13.777000	-7.575000
C	-1.083000	-14.402000	-6.516000
C	-1.525000	-13.914000	-5.110000
C	-1.564000	-12.415000	-4.993000
C	-2.703000	-11.699000	-5.383000
C	-2.741000	-10.319000	-5.309000
C	-1.633000	-9.632000	-4.864000
C	-0.494000	-10.333000	-4.454000

C	-0.465000	-11.718000	-4.528000
C	-1.072000	-15.916000	-6.618000
O	-0.283000	-16.578000	-5.949000
N	-1.931000	-16.454000	-7.468000
C	-2.137000	-17.903000	-7.557000
C	-3.439000	-18.176000	-8.309000
C	-4.692000	-17.551000	-7.680000
C	-5.937000	-17.932000	-8.478000
C	-4.790000	-18.011000	-6.283000
C	-0.985000	-18.643000	-8.263000
O	-0.759000	-19.804000	-8.000000
N	-0.295000	-17.969000	-9.178000
C	0.860000	-18.557000	-9.871000
C	1.089000	-17.922000	-11.227000
C	-0.118000	-18.174000	-12.142000
C	1.389000	-16.381000	-11.063000
C	2.150000	-18.444000	-9.056000
O	3.221000	-18.826000	-9.541000
N	2.047000	-17.917000	-7.830000
C	3.174000	-17.912000	-6.885000
C	3.353000	-16.539000	-6.246000
C	3.823000	-15.472000	-7.213000
C	2.925000	-14.808000	-8.055000
C	3.375000	-13.800000	-8.974000
C	4.745000	-13.478000	-9.016000
O	5.236000	-12.509000	-9.886000
C	5.639000	-14.142000	-8.171000
C	5.183000	-15.128000	-7.296000
C	3.046000	-18.990000	-5.797000
O	3.514000	-18.805000	-4.661000
N	2.430000	-20.123000	-6.116000
C	2.469000	-21.212000	-5.166000
C	1.238000	-22.090000	-5.045000
O	1.242000	-23.039000	-4.248000
N	0.178000	-21.802000	-5.794000
C	-0.997000	-22.660000	-5.714000
C	-2.281000	-21.870000	-6.045000
C	-0.811000	-23.807000	-6.685000
O	-0.398000	-23.593000	-7.818000
N	-1.113000	-25.042000	-6.234000
C	-1.467000	-25.341000	-4.831000
C	-1.171000	-26.781000	-4.708000
C	-1.387000	-27.338000	-6.073000
C	-0.908000	-26.238000	-7.014000
C	-1.792000	-26.202000	-8.232000
O	-2.957000	-25.834000	-8.122000
N	-1.246000	-26.568000	-9.384000
C	-1.996000	-26.599000	-10.643000
C	-2.122000	-25.299000	-11.408000
O	-2.764000	-25.275000	-12.445000
N	-1.518000	-24.211000	-10.922000
C	-1.697000	-22.906000	-11.583000
C	-1.953000	-21.780000	-10.540000
C	-3.385000	-21.720000	-10.075000
C	-3.798000	-22.421000	-8.944000
C	-5.117000	-22.416000	-8.562000
C	-6.033000	-21.708000	-9.301000
C	-5.639000	-21.011000	-10.424000

C	-4.329000	-21.030000	-10.817000
C	-0.473000	-22.567000	-12.416000
O	0.629000	-22.766000	-11.940000
N	-0.653000	-22.023000	-13.620000
C	0.503000	-21.519000	-14.356000
C	1.104000	-22.629000	-15.250000
O	2.067000	-22.104000	-16.173000
C	0.133000	-20.347000	-15.223000
O	-0.953000	-20.326000	-15.800000
N	1.040000	-19.379000	-15.360000
C	0.812000	-18.292000	-16.295000
C	1.786000	-17.116000	-16.101000
C	3.248000	-17.385000	-16.561000
C	4.075000	-16.089000	-16.707000
C	5.412000	-16.353000	-17.468000
N	5.085000	-16.614000	-18.920000
C	0.866000	-18.841000	-17.723000
O	0.329000	-18.217000	-18.638000
N	1.451000	-20.032000	-17.901000
C	1.627000	-20.614000	-19.252000
C	3.008000	-21.259000	-19.367000
C	4.110000	-20.313000	-19.000000
O	4.810000	-20.586000	-17.999000
O	4.249000	-19.290000	-19.691000
C	0.600000	-21.667000	-19.709000
O	0.890000	-22.434000	-20.639000
N	-0.558000	-21.730000	-19.042000
C	-1.705000	-22.587000	-19.450000
C	-1.475000	-24.097000	-19.240000
C	-1.361000	-24.499000	-17.779000
O	-2.007000	-23.915000	-16.890000
N	-0.521000	-25.511000	-17.528000
C	-2.941000	-22.123000	-18.716000
O	-2.866000	-21.217000	-17.886000
N	-4.085000	-22.728000	-19.021000
C	-5.351000	-22.181000	-18.542000
C	-6.483000	-22.406000	-19.559000
C	-6.912000	-23.861000	-19.635000
O	-6.311000	-24.746000	-18.984000
N	-7.960000	-24.129000	-20.436000
C	-5.722000	-22.632000	-17.122000
O	-6.815000	-22.360000	-16.663000
N	-4.797000	-23.304000	-16.446000
C	-4.888000	-23.530000	-15.010000
C	-4.577000	-22.232000	-14.255000
O	-3.240000	-21.807000	-14.497000
C	-6.211000	-24.148000	-14.506000
O	-6.583000	-23.959000	-13.339000
N	-6.906000	-24.907000	-15.361000
C	-8.030000	-25.713000	-14.872000
C	-8.606000	-26.637000	-15.979000
C	-9.785000	-27.481000	-15.446000
C	-9.138000	-25.792000	-17.130000
C	-10.000000	-24.661000	-16.663000
C	-7.557000	-26.543000	-13.686000
O	-6.647000	-27.326000	-13.820000
N	-8.122000	-26.354000	-12.508000
C	-7.696000	-27.152000	-11.369000

C	-7.125000	-26.287000	-10.241000
C	-5.745000	-25.723000	-10.620000
C	-8.146000	-25.203000	-9.848000
C	-8.118000	-24.811000	-8.376000
C	-8.879000	-27.942000	-10.820000
O	-10.042000	-27.647000	-11.127000
N	-8.579000	-28.930000	-9.987000
C	-9.593000	-29.826000	-9.441000
C	-9.062000	-31.261000	-9.393000
C	-8.733000	-31.748000	-10.780000
O	-7.881000	-31.288000	-8.579000
C	-10.024000	-29.459000	-8.012000
O	-9.362000	-28.681000	-7.300000
N	-11.125000	-30.055000	-7.579000
C	-11.621000	-29.796000	-6.255000
C	-12.786000	-30.719000	-5.948000
C	-13.129000	-30.806000	-4.481000
C	-14.482000	-31.471000	-4.269000
N	-14.938000	-31.399000	-2.881000
C	-15.911000	-30.596000	-2.435000
N	-16.271000	-30.642000	-1.151000
N	-16.531000	-29.756000	-3.265000
C	-10.499000	-29.953000	-5.237000
O	-10.390000	-29.183000	-4.269000
N	-9.633000	-30.925000	-5.474000
C	-8.586000	-31.252000	-4.509000
C	-8.133000	-32.701000	-4.702000
C	-7.248000	-33.210000	-3.580000
C	-6.952000	-34.694000	-3.736000
C	-5.454000	-34.970000	-3.564000
N	-4.937000	-34.627000	-2.185000
C	-7.394000	-30.311000	-4.559000
O	-6.720000	-30.091000	-3.556000
N	-7.112000	-29.777000	-5.742000
C	-6.127000	-28.686000	-5.888000
C	-5.784000	-28.466000	-7.378000
C	-4.757000	-29.486000	-7.943000
C	-4.847000	-29.651000	-9.445000
O	-3.806000	-29.911000	-10.096000
O	-5.963000	-29.519000	-9.984000
C	-6.635000	-27.395000	-5.205000
O	-5.884000	-26.728000	-4.468000
N	-7.917000	-27.082000	-5.409000
C	-8.564000	-25.975000	-4.677000
C	-10.048000	-25.894000	-5.022000
C	-10.792000	-24.808000	-4.309000
C	-10.973000	-23.568000	-4.900000
C	-11.698000	-22.561000	-4.257000
C	-12.271000	-22.790000	-3.014000
C	-12.122000	-24.027000	-2.408000
C	-11.383000	-25.043000	-3.062000
C	-8.349000	-26.113000	-3.174000
O	-7.907000	-25.153000	-2.518000
N	-8.618000	-27.305000	-2.623000
C	-8.395000	-27.540000	-1.182000
C	-8.887000	-28.952000	-0.785000
C	-10.429000	-29.043000	-0.818000
C	-10.975000	-30.455000	-0.632000

O	-10.590000	-31.390000	-1.335000
N	-11.895000	-30.603000	0.297000
C	-6.957000	-27.291000	-0.732000
O	-6.693000	-26.736000	0.332000
N	-6.023000	-27.719000	-1.570000
C	-4.588000	-27.565000	-1.319000
C	-3.806000	-28.330000	-2.407000
C	-3.677000	-29.831000	-2.185000
C	-3.138000	-30.125000	-0.803000
O	-3.682000	-31.042000	-0.134000
O	-2.194000	-29.403000	-0.360000
C	-4.243000	-26.089000	-1.388000
O	-3.449000	-25.583000	-0.580000
N	-4.852000	-25.410000	-2.361000
C	-4.700000	-23.953000	-2.550000
C	-5.049000	-23.138000	-1.321000
O	-4.372000	-22.136000	-1.022000
N	-6.099000	-23.565000	-0.597000
C	-6.505000	-22.896000	0.648000
C	-7.861000	-23.365000	1.188000
C	-9.083000	-23.234000	0.286000
C	-10.322000	-23.779000	0.978000
C	-9.297000	-21.798000	-0.143000
C	-5.470000	-23.108000	1.717000
O	-5.207000	-22.195000	2.512000
N	-4.883000	-24.305000	1.766000
C	-3.776000	-24.501000	2.724000
C	-3.286000	-25.952000	2.743000
C	-4.268000	-26.870000	3.398000
C	-3.905000	-28.367000	3.256000
C	-4.755000	-29.029000	2.158000
N	-4.921000	-30.506000	2.339000
C	-2.618000	-23.522000	2.455000
O	-1.970000	-23.029000	3.389000
N	-2.368000	-23.244000	1.175000
C	-1.316000	-22.302000	0.776000
C	-1.090000	-22.289000	-0.754000
C	-0.086000	-21.191000	-1.139000
C	-0.620000	-23.658000	-1.289000
C	0.717000	-24.081000	-0.795000
C	-1.688000	-20.893000	1.227000
O	-0.874000	-20.200000	1.801000
N	-2.947000	-20.492000	1.008000
C	-3.362000	-19.135000	1.339000
C	-4.379000	-18.660000	0.298000
C	-3.724000	-18.239000	-0.986000
C	-3.068000	-17.016000	-1.068000
C	-2.419000	-16.630000	-2.229000
C	-2.397000	-17.488000	-3.326000
C	-3.035000	-18.717000	-3.251000
C	-3.696000	-19.087000	-2.074000
C	-3.831000	-18.899000	2.784000
O	-3.858000	-17.748000	3.257000
N	-4.183000	-19.985000	3.489000
C	-4.636000	-19.895000	4.878000
C	-6.176000	-20.062000	4.943000
C	-6.961000	-19.034000	4.112000
C	-7.447000	-17.853000	4.684000

C	-8.181000	-16.939000	3.924000
C	-8.414000	-17.176000	2.600000
C	-7.951000	-18.327000	2.012000
C	-7.225000	-19.252000	2.765000
C	-3.913000	-20.893000	5.842000
O	-4.563000	-21.741000	6.472000
N	-2.572000	-20.770000	5.980000
C	-1.850000	-19.719000	5.241000
C	-0.387000	-19.969000	5.614000
C	-0.436000	-20.633000	6.937000
C	-1.617000	-21.581000	6.777000
C	-2.102000	-22.086000	8.142000
O	-1.789000	-23.209000	8.563000
N	-2.867000	-21.297000	8.857000
C	-3.832000	-21.996000	9.713000
C	-4.298000	-21.140000	10.825000
O	-3.516000	-20.815000	11.708000
N	-5.567000	-20.779000	10.831000
C	-6.665000	-21.394000	10.077000
C	-6.943000	-20.813000	8.663000
C	-8.387000	-21.036000	8.366000
C	-6.662000	-19.297000	8.649000
C	-6.931000	-22.895000	10.238000
O	-6.385000	-23.766000	9.539000
N	-7.818000	-23.138000	11.205000
C	-8.356000	-24.425000	11.512000
C	-9.448000	-24.237000	12.555000
O	-10.607000	-23.662000	11.975000
C	-8.946000	-25.077000	10.276000
O	-9.215000	-24.437000	9.271000
N	-9.169000	-26.363000	10.363000
C	-9.769000	-27.112000	9.297000
C	-9.716000	-28.582000	9.715000
C	-9.868000	-29.570000	8.551000
C	-8.847000	-29.338000	7.402000
O	-7.598000	-29.213000	7.650000
O	-9.320000	-29.283000	6.237000
C	-11.224000	-26.673000	9.064000
O	-11.755000	-26.718000	7.935000
N	-11.885000	-26.293000	10.153000
C	-13.246000	-25.817000	10.077000
C	-13.866000	-25.720000	11.476000
C	-15.181000	-25.002000	11.497000
C	-15.273000	-23.715000	12.008000
C	-16.474000	-23.041000	11.993000
C	-17.588000	-23.639000	11.472000
C	-17.509000	-24.931000	10.959000
C	-16.320000	-25.604000	10.982000
C	-13.227000	-24.455000	9.351000
O	-14.008000	-24.227000	8.448000
N	-12.289000	-23.577000	9.721000
C	-12.068000	-22.318000	8.992000
C	-12.040000	-22.579000	7.492000
O	-12.832000	-21.993000	6.706000
N	-11.153000	-23.469000	7.074000
C	-11.004000	-23.753000	5.650000
C	-9.733000	-24.577000	5.363000
C	-8.433000	-23.907000	5.852000

C	-7.218000	-24.511000	5.161000
C	-6.522000	-25.538000	6.039000
N	-6.346000	-25.038000	7.420000
C	-12.190000	-24.428000	5.003000
O	-12.424000	-24.174000	3.833000
N	-12.914000	-25.311000	5.714000
C	-14.119000	-25.968000	5.127000
C	-14.727000	-27.061000	6.051000
C	-14.069000	-28.479000	5.988000
C	-14.626000	-29.531000	7.059000
O	-14.755000	-29.207000	8.278000
O	-14.931000	-30.696000	6.675000
C	-15.191000	-24.910000	4.884000
O	-15.978000	-24.975000	3.942000
N	-15.214000	-23.919000	5.758000
C	-16.240000	-22.891000	5.664000
C	-16.319000	-22.112000	6.967000
O	-15.335000	-21.091000	7.008000
C	-16.009000	-21.966000	4.478000
O	-16.967000	-21.529000	3.842000
N	-14.741000	-21.690000	4.136000
C	-14.458000	-21.008000	2.861000
C	-12.935000	-20.747000	2.647000
C	-12.730000	-20.193000	1.303000
C	-12.352000	-19.815000	3.723000
C	-10.831000	-19.776000	3.740000
C	-14.977000	-21.890000	1.706000
O	-15.658000	-21.432000	0.804000
N	-14.628000	-23.168000	1.703000
C	-15.052000	-24.027000	0.605000
C	-14.535000	-25.463000	0.809000
C	-14.920000	-26.570000	-0.196000
C	-13.835000	-26.720000	-1.265000
C	-15.072000	-27.870000	0.541000
C	-16.564000	-24.058000	0.447000
O	-17.065000	-23.963000	-0.653000
N	-17.290000	-24.273000	1.530000
C	-18.778000	-24.309000	1.503000
C	-19.304000	-24.402000	2.944000
C	-20.815000	-24.328000	3.066000
C	-21.447000	-23.139000	3.482000
C	-22.868000	-23.081000	3.593000
C	-23.660000	-24.247000	3.297000
C	-23.040000	-25.415000	2.890000
C	-21.613000	-25.466000	2.793000
C	-19.319000	-23.050000	0.864000
O	-20.181000	-23.096000	-0.019000
N	-18.824000	-21.900000	1.310000
C	-19.297000	-20.637000	0.747000
C	-18.698000	-19.423000	1.473000
C	-19.421000	-18.141000	1.195000
N	-20.608000	-17.802000	1.812000
C	-21.016000	-16.626000	1.366000
N	-20.137000	-16.190000	0.482000
C	-19.127000	-17.113000	0.362000
C	-19.038000	-20.545000	-0.756000
O	-19.937000	-20.216000	-1.530000
N	-17.819000	-20.853000	-1.180000

C	-17.420000	-20.513000	-2.549000
C	-15.969000	-19.986000	-2.580000
C	-15.808000	-18.517000	-2.236000
C	-15.799000	-17.536000	-3.240000
C	-15.639000	-16.158000	-2.915000
C	-15.474000	-15.788000	-1.605000
O	-15.341000	-14.457000	-1.281000
C	-15.498000	-16.749000	-0.595000
C	-15.643000	-18.105000	-0.915000
C	-17.595000	-21.626000	-3.581000
O	-17.178000	-21.476000	-4.727000
N	-18.190000	-22.751000	-3.201000
C	-18.295000	-23.845000	-4.166000
C	-17.288000	-24.996000	-3.910000
C	-15.855000	-24.463000	-3.816000
O	-17.618000	-25.659000	-2.693000
C	-19.692000	-24.409000	-4.325000
O	-19.867000	-25.526000	-4.783000
N	-20.695000	-23.634000	-3.957000
C	-22.035000	-23.969000	-4.391000
C	-23.073000	-23.440000	-3.422000
C	-24.215000	-24.400000	-3.268000
O	-24.787000	-24.785000	-4.317000
O	-24.509000	-24.800000	-2.112000
C	-22.326000	-23.506000	-5.829000
O	-23.024000	-22.511000	-6.066000
N	-21.773000	-24.254000	-6.784000
C	-21.915000	-23.971000	-8.200000
C	-21.296000	-25.090000	-8.993000
C	-20.008000	-25.500000	-8.491000
C	-19.600000	-26.771000	-8.231000
N	-18.302000	-26.760000	-7.785000
C	-17.855000	-25.464000	-7.752000
C	-16.633000	-24.955000	-7.376000
C	-16.477000	-23.609000	-7.434000
C	-17.505000	-22.771000	-7.859000
C	-18.714000	-23.270000	-8.233000
C	-18.907000	-24.646000	-8.186000
C	-23.358000	-23.920000	-8.646000
O	-24.213000	-24.662000	-8.121000
N	-23.587000	-23.100000	-9.673000
C	-24.904000	-22.857000	-10.232000
C	-25.491000	-21.590000	-9.625000
C	-25.745000	-21.757000	-8.115000
C	-24.538000	-20.423000	-9.906000
C	-24.776000	-22.472000	-11.694000
O	-25.133000	-21.342000	-12.033000
N	-24.277000	-23.366000	-12.555000
C	-23.892000	-24.711000	-12.144000
C	-25.136000	-25.512000	-11.759000
C	-24.811000	-26.949000	-11.403000
O	-23.654000	-27.377000	-11.654000
O	-25.708000	-27.646000	-10.874000
C	-23.131000	-25.458000	-13.239000
O	-22.180000	-26.202000	-12.957000
N	-23.551000	-25.227000	-14.482000
C	-23.132000	-26.049000	-15.614000
C	-22.585000	-25.169000	-16.766000

C	-23.610000	-24.938000	-17.912000
O	-24.731000	-25.473000	-17.837000
O	-23.278000	-24.218000	-18.897000
C	-22.138000	-27.186000	-15.249000
O	-22.375000	-28.358000	-15.569000
N	-21.042000	-26.852000	-14.567000
C	-19.857000	-27.736000	-14.518000
C	-20.231000	-29.165000	-14.084000
C	-20.012000	-29.465000	-12.626000
C	-20.852000	-28.566000	-11.759000
O	-22.063000	-28.459000	-11.944000
N	-20.215000	-27.897000	-10.818000
C	-19.248000	-27.825000	-15.914000
O	-19.098000	-28.944000	-16.418000
N	-18.775000	-26.728000	-16.526000
C	-18.020000	-25.600000	-15.935000
C	-18.850000	-24.613000	-15.106000
C	-19.686000	-23.683000	-16.004000
C	-19.916000	-22.296000	-15.415000
N	-18.911000	-21.293000	-15.821000
C	-17.970000	-20.783000	-15.015000
N	-17.870000	-21.175000	-13.745000
N	-17.123000	-19.871000	-15.476000
C	-16.695000	-26.042000	-15.314000
O	-16.541000	-26.080000	-14.090000
N	-15.724000	-26.380000	-16.174000
C	-15.805000	-26.222000	-17.634000
C	-14.782000	-27.201000	-18.121000
C	-13.713000	-27.228000	-17.072000
C	-14.403000	-26.844000	-15.750000
C	-13.574000	-25.781000	-15.020000
O	-12.708000	-26.126000	-14.225000
N	-13.814000	-24.503000	-15.299000
C	-13.006000	-23.440000	-14.679000
C	-12.786000	-22.275000	-15.661000
C	-14.015000	-21.433000	-15.956000
C	-15.002000	-22.091000	-16.929000
O	-16.178000	-21.641000	-16.989000
O	-14.611000	-23.055000	-17.627000
C	-13.601000	-22.957000	-13.352000
O	-13.157000	-21.941000	-12.801000
N	-14.601000	-23.698000	-12.840000
C	-15.315000	-23.369000	-11.581000
C	-16.332000	-24.476000	-11.211000
C	-17.752000	-24.252000	-11.797000
O	-18.097000	-23.188000	-12.341000
N	-18.601000	-25.292000	-11.660000
C	-14.357000	-23.172000	-10.397000
O	-14.382000	-22.156000	-9.709000
N	-13.513000	-24.163000	-10.171000
C	-12.595000	-24.119000	-9.053000
C	-12.034000	-25.505000	-8.782000
C	-12.985000	-26.430000	-8.075000
C	-13.279000	-26.256000	-6.729000
C	-14.140000	-27.098000	-6.070000
C	-14.694000	-28.151000	-6.749000
O	-15.528000	-29.000000	-6.095000
C	-14.417000	-28.364000	-8.084000

C	-13.562000	-27.504000	-8.741000
C	-11.435000	-23.122000	-9.245000
O	-10.980000	-22.524000	-8.287000
N	-10.967000	-22.960000	-10.480000
C	-9.922000	-21.962000	-10.795000
C	-9.532000	-22.063000	-12.265000
C	-8.475000	-21.014000	-12.705000
C	-8.338000	-20.943000	-14.216000
N	-9.399000	-20.144000	-14.823000
C	-9.869000	-20.333000	-16.061000
N	-9.359000	-21.304000	-16.819000
N	-10.854000	-19.573000	-16.543000
C	-10.450000	-20.559000	-10.505000
O	-9.781000	-19.755000	-9.888000
N	-11.681000	-20.291000	-10.934000
C	-12.333000	-19.022000	-10.668000
C	-13.607000	-18.888000	-11.474000
C	-13.335000	-18.760000	-12.925000
C	-14.558000	-18.309000	-13.678000
O	-14.495000	-18.223000	-14.922000
O	-15.589000	-18.030000	-13.025000
C	-12.653000	-18.787000	-9.227000
O	-12.555000	-17.641000	-8.743000
N	-13.035000	-19.849000	-8.524000
C	-13.377000	-19.688000	-7.107000
C	-14.082000	-20.947000	-6.520000
C	-12.152000	-19.331000	-6.267000
O	-12.275000	-18.549000	-5.339000
N	-10.982000	-19.898000	-6.571000
C	-9.792000	-19.588000	-5.754000
C	-8.660000	-20.602000	-5.962000
C	-7.507000	-20.558000	-4.957000
C	-7.968000	-20.821000	-3.509000
C	-6.397000	-21.545000	-5.352000
C	-9.297000	-18.140000	-5.881000
O	-8.846000	-17.540000	-4.899000
N	-9.375000	-17.574000	-7.078000
C	-9.052000	-16.158000	-7.256000
C	-10.000000	-15.217000	-6.540000
O	-9.575000	-14.252000	-5.877000
N	-11.302000	-15.509000	-6.643000
C	-12.299000	-14.766000	-5.877000
C	-13.701000	-15.185000	-6.257000
C	-14.078000	-14.662000	-7.623000
O	-13.659000	-13.514000	-7.956000
O	-14.754000	-15.383000	-8.387000
C	-12.064000	-14.851000	-4.388000
O	-12.002000	-13.829000	-3.720000
N	-11.899000	-16.049000	-3.844000
C	-11.513000	-16.135000	-2.437000
C	-11.055000	-17.570000	-2.068000
C	-10.434000	-17.599000	-0.722000
C	-12.209000	-18.525000	-2.086000
C	-10.386000	-15.136000	-2.114000
O	-10.474000	-14.361000	-1.159000
N	-9.309000	-15.186000	-2.899000
C	-8.111000	-14.454000	-2.515000
C	-6.810000	-14.884000	-3.321000

C	-5.584000	-13.944000	-2.955000
C	-6.476000	-16.407000	-3.083000
C	-8.399000	-12.976000	-2.684000
O	-8.014000	-12.175000	-1.832000
N	-9.064000	-12.601000	-3.765000
C	-9.380000	-11.188000	-3.959000
C	-10.338000	-10.634000	-2.900000
O	-10.099000	-9.566000	-2.311000
N	-11.443000	-11.354000	-2.644000
C	-12.477000	-10.839000	-1.697000
C	-13.649000	-11.816000	-1.662000
C	-14.376000	-11.857000	-2.964000
O	-15.242000	-12.754000	-3.127000
O	-14.105000	-10.976000	-3.834000
C	-11.877000	-10.715000	-0.318000
O	-11.985000	-9.693000	0.357000
N	-11.187000	-11.737000	0.130000
C	-10.629000	-11.647000	1.493000
C	-10.029000	-12.995000	1.889000
C	-9.298000	-13.072000	3.200000
C	-9.933000	-12.815000	4.408000
C	-9.246000	-12.949000	5.629000
C	-7.933000	-13.405000	5.610000
O	-7.232000	-13.558000	6.755000
C	-7.321000	-13.714000	4.432000
C	-8.009000	-13.563000	3.240000
C	-9.603000	-10.548000	1.676000
O	-9.597000	-9.853000	2.678000
N	-8.657000	-10.461000	0.753000
C	-7.492000	-9.569000	0.940000
C	-6.260000	-10.136000	0.204000
C	-5.639000	-11.266000	0.943000
O	-4.979000	-11.062000	1.979000
N	-5.874000	-12.487000	0.461000
C	-7.688000	-8.128000	0.516000
O	-7.001000	-7.247000	1.038000
N	-8.548000	-7.881000	-0.471000
C	-8.681000	-6.523000	-1.009000
C	-8.101000	-6.487000	-2.441000
C	-6.639000	-6.892000	-2.495000
C	-6.284000	-8.173000	-2.911000
C	-4.962000	-8.621000	-2.911000
C	-3.946000	-7.746000	-2.504000
C	-4.288000	-6.422000	-2.098000
C	-5.657000	-6.012000	-2.095000
C	-10.111000	-5.890000	-1.011000
O	-10.324000	-4.770000	-0.513000
N	-11.052000	-6.613000	-1.596000
C	-12.371000	-6.055000	-1.901000
C	-13.111000	-6.908000	-2.981000
C	-14.544000	-6.377000	-3.181000
C	-12.256000	-6.899000	-4.264000
C	-12.856000	-7.592000	-5.495000
C	-13.250000	-5.866000	-0.676000
O	-13.702000	-4.768000	-0.433000
N	-13.479000	-6.902000	0.130000
C	-14.312000	-6.706000	1.317000
C	-14.592000	-8.059000	1.967000

S	-15.560000	-9.123000	0.811000
C	-13.642000	-5.721000	2.326000
O	-14.315000	-4.858000	2.902000
N	-12.308000	-5.846000	2.551000
C	-11.309000	-6.893000	2.251000
C	-10.191000	-6.610000	3.271000
C	-10.254000	-5.184000	3.467000
C	-11.749000	-4.823000	3.412000
C	-11.934000	-3.399000	2.876000
O	-12.067000	-2.472000	3.684000
N	-11.896000	-3.178000	1.558000
C	-11.974000	-1.779000	1.065000
C	-11.558000	-1.658000	-0.420000
C	-13.430000	-1.314000	1.218000
O	-13.699000	-0.158000	1.534000
N	-14.374000	-2.212000	0.976000
C	-15.808000	-1.823000	1.127000
C	-16.691000	-2.934000	0.592000
C	-16.773000	-3.055000	-0.941000
C	-17.329000	-4.410000	-1.354000
C	-17.643000	-1.951000	-1.546000
C	-16.115000	-1.534000	2.598000
O	-16.848000	-0.601000	2.934000
N	-15.532000	-2.311000	3.506000
C	-15.829000	-2.120000	4.932000
C	-15.356000	-3.326000	5.750000
C	-15.838000	-3.265000	7.154000
C	-17.398000	-3.469000	7.247000
O	-18.075000	-3.887000	6.246000
O	-17.936000	-3.221000	8.339000
C	-15.205000	-0.814000	5.458000
O	-15.793000	-0.070000	6.242000
N	-13.997000	-0.519000	5.008000
C	-13.365000	0.767000	5.358000
C	-11.965000	0.869000	4.674000
C	-11.361000	2.220000	4.794000
C	-10.551000	2.557000	5.900000
C	-10.036000	3.872000	6.017000
C	-10.333000	4.824000	5.050000
C	-11.171000	4.495000	3.971000
C	-11.658000	3.192000	3.854000
C	-14.285000	1.898000	4.852000
O	-14.532000	2.900000	5.523000
N	-14.792000	1.753000	3.638000
C	-15.550000	2.827000	3.046000
C	-15.781000	2.620000	1.507000
C	-16.437000	3.835000	0.917000
O	-14.521000	2.444000	0.822000
C	-16.860000	3.046000	3.829000
O	-17.230000	4.206000	4.120000
N	-17.563000	1.967000	4.191000
C	-18.818000	2.108000	4.973000
C	-19.401000	0.752000	5.335000
C	-19.778000	-0.061000	4.143000
C	-20.273000	-1.435000	4.619000
C	-20.295000	-2.457000	3.475000
N	-20.078000	-3.875000	3.995000
C	-18.567000	2.824000	6.280000

O	-19.267000	3.787000	6.594000
N	-17.588000	2.343000	7.053000
C	-17.354000	2.904000	8.365000
C	-16.282000	2.106000	9.145000
C	-16.630000	0.671000	9.466000
C	-17.654000	0.563000	10.605000
C	-17.766000	-0.899000	11.069000
N	-18.894000	-1.054000	12.050000
C	-16.895000	4.354000	8.276000
O	-17.179000	5.155000	9.164000
N	-16.091000	4.672000	7.269000
C	-15.554000	6.029000	7.146000
C	-14.490000	6.108000	6.030000
C	-13.737000	7.458000	5.987000
C	-12.535000	7.625000	6.635000
C	-11.876000	8.878000	6.605000
C	-12.408000	9.939000	5.930000
C	-13.584000	9.779000	5.243000
C	-14.255000	8.535000	5.297000
C	-16.705000	6.949000	6.830000
O	-16.806000	8.048000	7.363000
N	-17.610000	6.503000	5.964000
C	-18.669000	7.370000	5.537000
C	-19.296000	6.833000	4.258000
O	-20.056000	5.693000	4.594000
C	-19.747000	7.532000	6.606000
O	-20.464000	8.510000	6.612000
N	-19.872000	6.581000	7.526000
C	-20.883000	6.704000	8.595000
C	-20.908000	5.466000	9.467000
C	-21.770000	4.392000	8.880000
C	-21.497000	3.045000	9.491000
O	-20.657000	2.950000	10.416000
O	-22.133000	2.059000	9.045000
C	-20.629000	7.869000	9.513000
O	-21.494000	8.212000	10.325000
N	-19.446000	8.456000	9.446000
C	-19.175000	9.624000	10.266000
C	-17.749000	9.583000	10.845000
C	-17.695000	8.598000	11.940000
C	-17.453000	7.267000	11.821000
N	-17.537000	6.661000	13.052000
C	-17.867000	7.599000	13.997000
C	-18.043000	7.468000	15.400000
C	-18.353000	8.599000	16.122000
C	-18.469000	9.855000	15.494000
C	-18.280000	9.989000	14.083000
C	-17.975000	8.839000	13.331000
C	-19.392000	10.931000	9.552000
O	-18.907000	11.931000	9.992000
N	-20.109000	10.944000	8.438000
C	-20.512000	12.208000	7.874000
C	-19.735000	12.681000	6.665000
O	-19.973000	13.767000	6.216000
N	-18.793000	11.913000	6.116000
C	-18.008000	12.456000	4.988000
C	-16.516000	12.287000	5.231000
C	-15.952000	13.341000	6.211000

O	-15.612000	12.978000	7.312000
N	-15.836000	14.652000	5.783000
C	-18.365000	11.834000	3.663000
O	-18.716000	10.651000	3.607000
N	-18.261000	12.624000	2.601000
C	-18.541000	12.127000	1.274000
C	-18.551000	13.289000	0.274000
C	-19.882000	14.030000	0.245000
O	-20.832000	13.660000	0.950000
N	-19.965000	15.059000	-0.593000
C	-17.439000	11.151000	0.879000
O	-16.239000	11.398000	1.153000
N	-17.840000	10.060000	0.244000
C	-16.939000	9.019000	-0.197000
C	-16.940000	7.864000	0.806000
C	-17.464000	8.532000	-1.534000
O	-18.690000	8.488000	-1.737000
N	-16.556000	8.144000	-2.415000
C	-16.878000	7.679000	-3.765000
C	-16.430000	8.743000	-4.795000
C	-17.014000	10.126000	-4.522000
C	-16.397000	11.003000	-3.642000
C	-16.966000	12.303000	-3.356000
C	-18.168000	12.674000	-3.995000
C	-18.789000	11.775000	-4.877000
C	-18.215000	10.528000	-5.145000
C	-16.216000	6.323000	-4.007000
O	-15.019000	6.161000	-3.725000
N	-16.956000	5.331000	-4.518000
C	-16.345000	3.979000	-4.673000
C	-17.044000	2.952000	-3.736000
C	-16.274000	1.689000	-3.533000
C	-16.309000	0.678000	-4.497000
C	-15.574000	-0.505000	-4.332000
C	-14.778000	-0.685000	-3.162000
C	-14.748000	0.314000	-2.213000
C	-15.502000	1.498000	-2.379000
C	-16.385000	3.519000	-6.146000
O	-17.435000	3.698000	-6.828000
N	-15.270000	2.973000	-6.682000
C	-15.304000	2.451000	-8.097000
C	-14.337000	3.179000	-9.056000
C	-12.855000	2.956000	-8.663000
C	-12.169000	3.878000	-7.872000
C	-10.750000	3.654000	-7.501000
C	-10.094000	2.532000	-7.984000
O	-8.745000	2.234000	-7.667000
C	-10.779000	1.655000	-8.836000
C	-12.156000	1.874000	-9.142000
C	-14.990000	0.958000	-8.180000
O	-14.311000	0.388000	-7.312000
N	-15.411000	0.339000	-9.277000
C	-15.090000	-1.045000	-9.548000
C	-16.376000	-1.901000	-9.531000
C	-16.190000	-3.426000	-9.690000
C	-15.462000	-4.169000	-8.750000
C	-15.345000	-5.577000	-8.851000
C	-15.976000	-6.225000	-9.883000

O	-15.865000	-7.605000	-9.996000
C	-16.706000	-5.505000	-10.831000
C	-16.818000	-4.127000	-10.734000
C	-14.522000	-0.996000	-10.971000
O	-15.261000	-0.777000	-11.953000
N	-13.210000	-1.215000	-11.082000
C	-12.518000	-1.132000	-12.366000
C	-11.058000	-0.707000	-12.082000
C	-10.211000	-0.520000	-13.319000
C	-10.161000	0.722000	-13.974000
C	-9.391000	0.902000	-15.125000
C	-8.624000	-0.136000	-15.609000
C	-8.615000	-1.364000	-14.946000
C	-9.440000	-1.568000	-13.816000
C	-12.577000	-2.496000	-13.108000
O	-12.102000	-3.491000	-12.617000
N	-13.184000	-2.550000	-14.289000
C	-13.328000	-3.841000	-14.923000
C	-14.801000	-4.334000	-14.865000
C	-15.790000	-3.239000	-14.966000
C	-17.275000	-3.670000	-14.951000
O	-17.695000	-4.529000	-14.160000
O	-18.027000	-3.063000	-15.723000
C	-12.750000	-3.842000	-16.322000
O	-13.190000	-4.625000	-17.144000
N	-11.725000	-3.028000	-16.614000
C	-11.127000	-3.104000	-17.950000
C	-11.017000	-1.720000	-18.601000
C	-10.308000	-1.754000	-19.922000
N	-10.910000	-2.204000	-21.078000
C	-10.038000	-2.158000	-22.072000
N	-8.892000	-1.699000	-21.601000
C	-9.027000	-1.449000	-20.259000
C	-9.765000	-3.833000	-17.982000
O	-8.852000	-3.498000	-17.225000
N	-9.660000	-4.870000	-18.815000
C	-8.381000	-5.558000	-19.055000
C	-8.597000	-7.047000	-19.361000
C	-7.328000	-7.765000	-19.765000
C	-7.502000	-9.277000	-19.735000
N	-6.412000	-9.934000	-20.447000
C	-5.457000	-10.691000	-19.895000
N	-5.408000	-10.950000	-18.571000
N	-4.533000	-11.219000	-20.684000
C	-7.548000	-4.900000	-20.182000
O	-7.960000	-4.858000	-21.341000
N	-6.372000	-4.403000	-19.814000
C	-5.451000	-3.764000	-20.751000
C	-4.114000	-3.561000	-20.060000
O	-3.264000	-2.739000	-20.838000
C	-5.262000	-4.628000	-22.000000
O	-5.097000	-5.850000	-21.895000
N	-5.312000	-4.010000	-23.175000
C	-5.076000	-4.733000	-24.435000
C	-5.466000	-3.876000	-25.646000
O	-4.696000	-2.680000	-25.691000
C	-3.601000	-5.158000	-24.527000
O	-3.239000	-6.027000	-25.314000

N	-2.756000	-4.583000	-23.686000
C	-1.364000	-4.935000	-23.699000
C	-0.537000	-3.642000	-23.621000
C	-0.836000	-2.643000	-24.742000
C	0.013000	-1.373000	-24.616000
C	1.509000	-1.724000	-24.421000
N	2.380000	-0.497000	-24.436000
C	-0.976000	-5.860000	-22.524000
O	0.223000	-6.031000	-22.228000
N	-1.943000	-6.459000	-21.832000
C	-1.580000	-7.228000	-20.657000
C	-2.850000	-7.609000	-19.875000
C	-2.929000	-7.570000	-18.328000
C	-2.544000	-8.875000	-17.719000
C	-2.141000	-6.479000	-17.698000
C	-0.744000	-8.462000	-21.068000
O	-1.160000	-9.232000	-21.923000
N	0.461000	-8.637000	-20.486000
C	1.154000	-7.619000	-19.682000
C	2.408000	-8.313000	-19.261000
C	2.719000	-9.284000	-20.340000
C	1.352000	-9.768000	-20.823000
C	0.991000	-11.087000	-20.103000
O	1.445000	-12.168000	-20.513000
N	0.190000	-10.997000	-19.036000
C	-0.314000	-12.175000	-18.311000
C	-0.812000	-11.711000	-16.956000
C	0.249000	-11.121000	-16.016000
C	0.497000	-9.793000	-15.778000
N	1.550000	-9.658000	-14.878000
C	1.937000	-10.916000	-14.465000
C	2.905000	-11.300000	-13.552000
C	3.102000	-12.643000	-13.352000
C	2.376000	-13.601000	-14.046000
C	1.397000	-13.227000	-14.974000
C	1.164000	-11.859000	-15.189000
C	-1.470000	-12.863000	-19.087000
O	-2.112000	-12.227000	-19.887000
N	-1.774000	-14.147000	-18.816000
C	-1.097000	-15.066000	-17.903000
C	-2.042000	-16.251000	-17.885000
C	-2.700000	-16.293000	-19.208000
C	-2.785000	-14.832000	-19.653000
C	-4.214000	-14.346000	-19.426000
O	-4.517000	-13.706000	-18.405000
N	-5.092000	-14.665000	-20.366000
C	-6.472000	-14.235000	-20.318000
C	-7.237000	-14.733000	-21.555000
C	-7.000000	-13.889000	-22.773000
C	-8.174000	-13.001000	-23.068000
O	-8.407000	-12.029000	-22.304000
O	-8.866000	-13.281000	-24.077000
C	-7.208000	-14.698000	-19.070000
O	-8.080000	-13.984000	-18.566000
N	-6.883000	-15.875000	-18.561000
C	-7.682000	-16.384000	-17.465000
C	-7.341000	-17.843000	-17.092000
C	-5.980000	-18.141000	-16.501000

C	-4.921000	-18.639000	-17.175000
N	-3.846000	-18.830000	-16.319000
C	-4.222000	-18.484000	-15.046000
C	-3.490000	-18.513000	-13.846000
C	-4.119000	-18.086000	-12.698000
C	-5.451000	-17.649000	-12.727000
C	-6.183000	-17.632000	-13.922000
C	-5.561000	-18.053000	-15.109000
C	-7.547000	-15.477000	-16.250000
O	-8.401000	-15.516000	-15.346000
N	-6.488000	-14.657000	-16.216000
C	-6.232000	-13.875000	-15.008000
C	-4.737000	-13.572000	-14.837000
C	-3.946000	-14.765000	-14.383000
S	-2.137000	-14.564000	-14.437000
C	-1.689000	-14.267000	-12.779000
C	-7.030000	-12.600000	-14.955000
O	-7.024000	-11.934000	-13.927000
N	-7.704000	-12.260000	-16.039000
C	-8.683000	-11.150000	-16.096000
C	-8.121000	-9.755000	-15.835000
O	-6.991000	-9.458000	-16.274000
N	-8.886000	-8.933000	-15.093000
C	-8.559000	-7.543000	-14.739000
C	-9.864000	-6.739000	-14.501000
C	-9.562000	-5.232000	-14.189000
C	-10.866000	-6.942000	-15.686000
C	-7.728000	-7.536000	-13.444000
O	-8.256000	-7.407000	-12.326000
N	-6.423000	-7.667000	-13.606000
C	-5.565000	-8.053000	-12.510000
C	-4.226000	-8.549000	-13.075000
C	-4.233000	-10.003000	-13.582000
S	-2.674000	-10.337000	-14.480000
C	-1.558000	-10.450000	-13.132000
C	-5.287000	-6.912000	-11.565000
O	-5.385000	-5.751000	-11.933000
N	-4.900000	-7.253000	-10.349000
C	-4.364000	-6.292000	-9.393000
C	-3.875000	-7.094000	-8.178000
C	-3.252000	-6.274000	-7.083000
N	-3.998000	-5.629000	-6.117000
C	-3.181000	-5.040000	-5.252000
N	-1.932000	-5.291000	-5.615000
C	-1.948000	-6.082000	-6.741000
C	-3.207000	-5.531000	-10.018000
O	-2.266000	-6.153000	-10.529000
N	-3.261000	-4.200000	-9.971000
C	-2.108000	-3.348000	-10.325000
C	-2.260000	-2.719000	-11.689000
O	-1.499000	-1.820000	-12.065000
N	-3.252000	-3.166000	-12.455000
C	-3.321000	-2.782000	-13.872000
C	-3.479000	-4.027000	-14.791000
C	-2.140000	-4.778000	-14.804000
C	-1.896000	-5.813000	-13.907000
C	-0.674000	-6.452000	-13.860000
C	0.359000	-6.038000	-14.705000

O	1.575000	-6.695000	-14.644000
C	0.168000	-4.996000	-15.595000
C	-1.078000	-4.354000	-15.636000
C	-4.354000	-1.694000	-14.168000
O	-4.721000	-1.443000	-15.315000
N	-4.780000	-1.010000	-13.119000
C	-5.513000	0.226000	-13.329000
C	-6.565000	0.414000	-12.237000
C	-6.123000	1.190000	-11.017000
C	-5.275000	0.341000	-10.026000
O	-5.062000	-0.876000	-10.296000
O	-4.832000	0.894000	-8.967000
C	-4.536000	1.404000	-13.318000
O	-4.848000	2.481000	-13.775000
N	-3.366000	1.183000	-12.747000
C	-2.438000	2.292000	-12.426000
C	-1.148000	1.765000	-11.676000
C	-0.132000	2.906000	-11.466000
C	-1.504000	1.102000	-10.336000
C	-0.356000	0.388000	-9.645000
C	-2.040000	3.039000	-13.674000
O	-2.102000	4.271000	-13.713000
N	-1.600000	2.315000	-14.701000
C	-1.257000	2.936000	-15.981000
C	-0.780000	1.907000	-16.987000
C	-1.773000	0.838000	-17.324000
C	-1.100000	-0.424000	-17.873000
O	-0.545000	-1.232000	-17.069000
O	-1.154000	-0.616000	-19.107000
C	-2.400000	3.809000	-16.548000
O	-2.159000	4.799000	-17.254000
N	-3.651000	3.476000	-16.217000
C	-4.756000	4.303000	-16.699000
C	-6.063000	3.515000	-16.671000
C	-6.184000	2.547000	-17.843000
C	-5.535000	1.327000	-17.820000
C	-5.607000	0.458000	-18.907000
C	-6.320000	0.827000	-20.017000
C	-6.954000	2.064000	-20.054000
C	-6.875000	2.908000	-18.976000
C	-4.861000	5.568000	-15.880000
O	-5.142000	6.647000	-16.394000
N	-4.640000	5.443000	-14.591000
C	-4.712000	6.602000	-13.724000
C	-4.552000	6.193000	-12.243000
C	-4.251000	7.427000	-11.348000
C	-5.840000	5.427000	-11.798000
C	-3.617000	7.592000	-14.122000
O	-3.892000	8.795000	-14.152000
N	-2.390000	7.085000	-14.421000
C	-1.234000	7.956000	-14.750000
C	0.081000	7.246000	-14.389000
C	0.475000	7.410000	-12.958000
C	0.055000	6.502000	-11.993000
C	0.385000	6.637000	-10.662000
C	1.212000	7.716000	-10.251000
C	1.641000	8.634000	-11.216000
C	1.257000	8.478000	-12.565000

C	-1.226000	8.487000	-16.220000
O	-0.400000	9.341000	-16.607000
N	-2.141000	7.980000	-17.040000
C	-2.360000	8.523000	-18.388000
C	-1.452000	7.944000	-19.465000
O	-1.285000	8.530000	-20.523000
N	-0.891000	6.776000	-19.231000
C	-0.029000	6.204000	-20.243000
C	0.628000	4.921000	-19.766000
C	1.501000	4.824000	-18.514000
C	2.597000	3.797000	-18.697000
C	2.007000	6.131000	-17.927000
C	-0.756000	5.934000	-21.561000
O	-0.161000	6.024000	-22.648000
N	-2.038000	5.571000	-21.479000
C	-2.704000	4.939000	-20.320000
C	-3.726000	4.013000	-20.996000
C	-4.075000	4.686000	-22.304000
C	-2.758000	5.341000	-22.752000
C	-3.037000	6.624000	-23.544000
O	-3.659000	6.569000	-24.611000
N	-2.642000	7.772000	-23.015000
C	-2.816000	9.027000	-23.746000
C	-2.867000	10.228000	-22.794000
C	-4.001000	10.169000	-21.753000
C	-3.999000	11.359000	-20.781000
C	-5.368000	10.012000	-22.423000
C	-1.722000	9.183000	-24.803000
O	-1.879000	9.884000	-25.784000
N	-0.612000	8.493000	-24.598000
C	0.443000	8.442000	-25.590000
C	1.769000	8.054000	-24.943000
C	2.854000	7.816000	-25.957000
C	3.330000	9.128000	-26.601000
O	2.877000	9.473000	-27.736000
O	4.143000	9.823000	-25.944000
C	0.055000	7.458000	-26.711000
O	0.159000	6.226000	-26.558000
N	-0.423000	8.032000	-27.821000
C	-0.821000	7.299000	-29.045000
C	-1.139000	8.278000	-30.178000
C	-2.437000	9.054000	-30.057000
C	-2.790000	9.736000	-31.416000
N	-3.675000	10.904000	-31.255000
C	-3.279000	12.178000	-31.330000
N	-2.007000	12.488000	-31.578000
N	-4.167000	13.146000	-31.163000
C	0.262000	6.344000	-29.558000
O	-0.023000	5.176000	-29.890000
N	1.489000	6.868000	-29.651000
C	2.649000	6.085000	-30.065000
C	3.938000	6.812000	-29.678000
C	4.357000	7.863000	-30.657000
C	4.946000	7.230000	-31.911000
N	4.979000	8.191000	-33.010000
C	3.955000	8.418000	-33.834000
N	2.809000	7.746000	-33.683000
N	4.080000	9.320000	-34.811000

C	2.683000	4.711000	-29.419000
O	3.364000	3.807000	-29.909000
N	1.936000	4.548000	-28.331000
C	2.174000	3.438000	-27.435000
C	2.137000	3.946000	-26.004000
C	3.359000	3.536000	-25.243000
O	4.325000	4.353000	-25.205000
O	3.376000	2.375000	-24.752000
C	1.327000	2.155000	-27.602000
O	1.388000	1.247000	-26.756000
N	0.561000	2.067000	-28.691000
C	-0.167000	0.824000	-28.993000
C	0.819000	-0.356000	-29.091000
C	1.121000	-0.855000	-30.535000
C	2.067000	0.067000	-31.341000
O	2.953000	0.727000	-30.775000
N	1.885000	0.095000	-32.671000
C	-1.353000	0.434000	-28.065000
O	-1.746000	-0.723000	-28.039000
N	-1.921000	1.375000	-27.310000
C	-3.207000	1.114000	-26.646000
C	-3.385000	2.035000	-25.442000
C	-2.522000	1.745000	-24.240000
C	-1.364000	2.465000	-24.001000
C	-0.585000	2.250000	-22.849000
C	-0.984000	1.303000	-21.934000
O	-0.230000	1.059000	-20.807000
C	-2.153000	0.581000	-22.159000
C	-2.903000	0.805000	-23.303000
C	-4.314000	1.409000	-27.675000
O	-4.074000	2.160000	-28.604000
N	-5.518000	0.843000	-27.497000
C	-6.623000	1.101000	-28.404000
C	-7.813000	0.115000	-28.205000
C	-7.411000	-1.345000	-28.352000
O	-8.377000	0.316000	-26.908000
C	-7.184000	2.511000	-28.205000
O	-6.905000	3.193000	-27.208000
N	-7.999000	2.949000	-29.161000
C	-8.705000	4.214000	-29.040000
C	-9.480000	4.487000	-30.338000
C	-10.120000	5.843000	-30.374000
C	-9.068000	6.902000	-30.539000
C	-9.592000	8.226000	-30.044000
N	-8.602000	9.352000	-30.255000
C	-9.680000	4.227000	-27.819000
O	-9.806000	5.229000	-27.120000
N	-10.341000	3.106000	-27.549000
C	-11.291000	3.062000	-26.467000
C	-12.070000	1.757000	-26.470000
C	-10.549000	3.244000	-25.165000
O	-11.097000	3.803000	-24.221000
N	-9.302000	2.795000	-25.126000
C	-8.451000	2.922000	-23.931000
C	-7.324000	1.902000	-23.989000
C	-7.829000	0.450000	-23.908000
C	-6.691000	-0.583000	-23.923000
O	-5.752000	-0.464000	-24.733000

O	-6.714000	-1.532000	-23.119000
C	-7.921000	4.338000	-23.725000
O	-7.887000	4.839000	-22.619000
N	-7.495000	5.007000	-24.786000
C	-7.181000	6.398000	-24.652000
C	-6.874000	6.971000	-26.036000
C	-6.696000	8.489000	-26.012000
C	-6.474000	9.146000	-27.397000
O	-6.175000	10.374000	-27.425000
O	-6.591000	8.462000	-28.445000
C	-8.355000	7.165000	-24.017000
O	-8.191000	8.014000	-23.140000
N	-9.563000	6.925000	-24.498000
C	-10.717000	7.677000	-23.987000
C	-12.022000	7.360000	-24.790000
C	-13.168000	8.233000	-24.277000
C	-11.852000	7.595000	-26.301000
C	-11.662000	9.081000	-26.648000
C	-10.992000	7.369000	-22.504000
O	-11.187000	8.289000	-21.672000
N	-11.007000	6.074000	-22.171000
C	-11.136000	5.633000	-20.764000
C	-10.973000	4.124000	-20.618000
C	-11.139000	3.621000	-19.166000
C	-12.464000	4.066000	-18.548000
C	-11.041000	2.069000	-19.136000
C	-10.076000	6.326000	-19.904000
O	-10.380000	6.915000	-18.881000
N	-8.827000	6.273000	-20.321000
C	-7.808000	6.894000	-19.489000
C	-6.410000	6.633000	-20.093000
O	-5.406000	7.191000	-19.276000
C	-8.096000	8.389000	-19.365000
O	-7.925000	9.015000	-18.311000
N	-8.532000	9.008000	-20.444000
C	-8.646000	10.474000	-20.399000
C	-9.006000	11.061000	-21.790000
C	-9.273000	12.588000	-21.791000
C	-7.954000	13.344000	-21.571000
N	-8.065000	14.753000	-21.141000
C	-8.147000	15.186000	-19.876000
N	-8.193000	14.346000	-18.839000
N	-8.204000	16.487000	-19.645000
C	-9.717000	10.844000	-19.376000
O	-9.619000	11.855000	-18.667000
N	-10.750000	10.017000	-19.303000
C	-11.876000	10.273000	-18.406000
C	-13.050000	9.423000	-18.861000
O	-14.060000	9.525000	-17.897000
C	-11.494000	10.020000	-16.904000
O	-11.770000	10.849000	-16.009000
N	-10.746000	8.940000	-16.652000
C	-10.210000	8.663000	-15.309000
C	-9.467000	7.290000	-15.306000
C	-8.629000	7.076000	-14.054000
C	-10.517000	6.141000	-15.341000
C	-9.808000	4.789000	-15.653000
C	-9.338000	9.800000	-14.775000

O	-9.496000	10.270000	-13.637000
N	-8.413000	10.252000	-15.612000
C	-7.488000	11.313000	-15.229000
C	-6.461000	11.604000	-16.420000
C	-5.723000	12.944000	-16.220000
C	-5.440000	10.428000	-16.569000
C	-8.254000	12.555000	-14.847000
O	-7.944000	13.240000	-13.854000
N	-9.292000	12.873000	-15.619000
C	-10.106000	14.042000	-15.296000
C	-11.071000	14.294000	-16.452000
C	-12.116000	15.307000	-16.174000
C	-11.498000	16.661000	-15.767000
C	-10.675000	17.304000	-16.899000
N	-10.309000	18.721000	-16.494000
C	-10.860000	13.864000	-13.964000
O	-10.886000	14.764000	-13.091000
N	-11.477000	12.693000	-13.779000
C	-12.201000	12.440000	-12.516000
C	-12.899000	11.073000	-12.564000
C	-14.085000	10.977000	-13.577000
C	-14.792000	9.608000	-13.436000
N	-15.917000	9.377000	-14.371000
C	-17.162000	9.802000	-14.151000
N	-18.138000	9.570000	-15.014000
N	-17.416000	10.499000	-13.067000
C	-11.239000	12.540000	-11.286000
O	-11.609000	13.098000	-10.275000
N	-10.034000	11.971000	-11.378000
C	-9.078000	11.952000	-10.251000
C	-7.860000	11.071000	-10.621000
C	-7.824000	9.608000	-10.061000
C	-6.801000	9.031000	-9.351000
N	-7.118000	7.730000	-9.026000
C	-8.365000	7.429000	-9.517000
C	-9.088000	6.233000	-9.466000
C	-10.328000	6.213000	-10.082000
C	-10.835000	7.344000	-10.738000
C	-10.109000	8.527000	-10.792000
C	-8.844000	8.576000	-10.187000
C	-8.622000	13.378000	-9.965000
O	-8.447000	13.782000	-8.820000
N	-8.428000	14.151000	-11.027000
C	-8.005000	15.537000	-10.907000
C	-7.629000	16.079000	-12.258000
C	-9.107000	16.396000	-10.303000
O	-8.861000	17.187000	-9.389000
N	-10.323000	16.246000	-10.811000
C	-11.449000	16.919000	-10.187000
C	-12.752000	16.720000	-10.973000
C	-12.852000	17.674000	-12.167000
O	-12.032000	18.605000	-12.302000
N	-13.805000	17.413000	-13.067000
C	-11.620000	16.561000	-8.736000
O	-11.923000	17.463000	-7.912000
N	-11.426000	15.285000	-8.392000
C	-11.464000	14.886000	-6.984000
C	-11.136000	13.392000	-6.758000

C	-11.238000	12.988000	-5.299000
C	-12.497000	12.957000	-4.675000
C	-12.627000	12.648000	-3.366000
C	-11.481000	12.364000	-2.600000
C	-10.193000	12.410000	-3.219000
C	-10.084000	12.720000	-4.532000
C	-10.448000	15.691000	-6.195000
O	-10.733000	16.213000	-5.137000
N	-9.230000	15.733000	-6.690000
C	-8.163000	16.390000	-5.921000
C	-6.832000	16.139000	-6.577000
C	-8.429000	17.880000	-5.796000
O	-8.294000	18.477000	-4.705000
N	-8.778000	18.515000	-6.907000
C	-9.037000	19.972000	-6.897000
C	-9.140000	20.523000	-8.325000
C	-7.903000	20.368000	-9.200000
C	-8.230000	20.653000	-10.669000
C	-6.930000	20.862000	-11.469000
N	-6.933000	20.315000	-12.900000
C	-10.310000	20.375000	-6.111000
O	-10.276000	21.341000	-5.332000
N	-11.401000	19.639000	-6.307000
C	-12.750000	20.117000	-5.902000
C	-13.634000	20.365000	-7.148000
C	-12.923000	21.190000	-8.202000
C	-12.777000	20.740000	-9.515000
C	-12.128000	21.521000	-10.483000
C	-11.598000	22.743000	-10.117000
O	-10.893000	23.513000	-11.025000
C	-11.710000	23.186000	-8.839000
C	-12.379000	22.412000	-7.879000
C	-13.508000	19.166000	-4.957000
O	-14.565000	19.536000	-4.476000
N	-12.986000	17.953000	-4.754000
C	-13.498000	16.984000	-3.817000
C	-14.629000	16.193000	-4.430000
O	-15.374000	15.515000	-3.719000
N	-14.732000	16.203000	-5.745000
C	-15.881000	15.587000	-6.410000
C	-16.913000	16.715000	-6.622000
C	-18.344000	16.240000	-6.655000
O	-18.653000	15.115000	-7.054000
N	-19.237000	17.140000	-6.322000
C	-15.485000	14.968000	-7.768000
O	-15.197000	15.715000	-8.694000
N	-15.469000	13.613000	-7.900000
C	-16.068000	12.580000	-7.021000
C	-15.578000	11.263000	-7.599000
C	-14.436000	11.598000	-8.613000
C	-14.801000	13.010000	-9.085000
C	-15.695000	12.941000	-10.294000
O	-16.046000	11.844000	-10.801000
N	-16.094000	14.099000	-10.768000
C	-17.065000	14.123000	-11.891000
C	-18.173000	15.179000	-11.686000
C	-18.961000	15.110000	-10.370000
C	-19.619000	13.750000	-10.114000

O	-19.962000	13.006000	-11.052000
N	-19.791000	13.411000	-8.847000
C	-16.278000	14.434000	-13.141000
O	-15.197000	15.038000	-13.077000
N	-16.798000	13.989000	-14.276000
C	-16.341000	14.473000	-15.558000
C	-15.994000	13.316000	-16.497000
C	-15.174000	13.765000	-17.680000
C	-16.001000	14.577000	-18.647000
O	-15.681000	15.750000	-18.949000
O	-17.018000	14.035000	-19.083000
C	-17.514000	15.286000	-16.094000
O	-18.497000	14.703000	-16.552000
N	-17.466000	16.617000	-15.972000
C	-18.699000	17.412000	-16.159000
C	-18.620000	18.718000	-15.357000
C	-18.440000	18.457000	-13.888000
O	-17.483000	19.455000	-15.799000
C	-19.072000	17.765000	-17.647000
O	-20.179000	18.243000	-17.916000
N	-18.168000	17.511000	-18.597000
C	-18.306000	18.027000	-19.959000
C	-16.971000	18.621000	-20.423000
C	-16.604000	19.895000	-19.661000
C	-15.182000	20.367000	-19.921000
O	-14.234000	19.570000	-19.993000
N	-15.019000	21.677000	-20.025000
C	-18.827000	17.057000	-21.022000
O	-19.423000	17.474000	-21.999000
N	-18.603000	15.769000	-20.870000
C	-18.946000	14.896000	-21.973000
C	-17.719000	14.098000	-22.421000
C	-16.604000	15.009000	-22.972000
O	-16.859000	16.147000	-23.377000
N	-15.366000	14.515000	-22.953000
C	-20.160000	14.032000	-21.676000
O	-20.182000	12.827000	-21.958000
N	-21.179000	14.681000	-21.117000
C	-22.388000	14.011000	-20.604000
C	-23.548000	14.046000	-21.619000
C	-23.404000	13.120000	-22.815000
C	-23.535000	13.892000	-24.131000
O	-24.366000	13.562000	-25.000000
N	-22.721000	14.947000	-24.270000
C	-22.126000	12.592000	-20.095000
O	-22.715000	11.615000	-20.566000
N	-21.225000	12.506000	-19.126000
C	-20.857000	11.255000	-18.513000
C	-19.455000	11.370000	-17.920000
O	-18.507000	11.602000	-18.955000
C	-21.863000	10.901000	-17.420000
O	-22.684000	11.719000	-17.030000
N	-21.799000	9.661000	-16.951000
C	-22.587000	9.220000	-15.812000
C	-22.425000	7.709000	-15.611000
C	-23.196000	7.271000	-14.416000
O	-22.935000	7.017000	-16.761000
C	-22.084000	9.982000	-14.581000

O	-20.875000	10.178000	-14.432000
N	-23.010000	10.485000	-13.759000
C	-22.652000	11.098000	-12.477000
C	-23.880000	11.726000	-11.852000
O	-24.000000	13.045000	-12.308000
C	-22.163000	10.037000	-11.499000
O	-22.663000	8.921000	-11.501000
N	-21.185000	10.365000	-10.672000
C	-20.690000	9.403000	-9.703000
C	-19.146000	9.518000	-9.629000
C	-18.441000	8.421000	-8.875000
C	-18.978000	7.562000	-7.943000
N	-17.997000	6.709000	-7.460000
C	-16.815000	6.990000	-8.107000
C	-15.546000	6.410000	-7.964000
C	-14.513000	6.937000	-8.730000
C	-14.722000	7.971000	-9.637000
C	-15.992000	8.553000	-9.776000
C	-17.058000	8.059000	-9.002000
C	-21.355000	9.816000	-8.399000
O	-21.078000	10.908000	-7.902000
N	-22.275000	8.970000	-7.847000
C	-22.703000	7.671000	-8.400000
C	-23.498000	7.073000	-7.271000
C	-24.090000	8.230000	-6.587000
C	-22.982000	9.273000	-6.601000
C	-22.109000	9.012000	-5.386000
O	-21.183000	8.220000	-5.493000
N	-22.376000	9.658000	-4.251000
C	-21.635000	9.330000	-3.060000
C	-21.741000	10.426000	-2.020000
C	-21.666000	11.770000	-2.688000
C	-23.043000	10.333000	-1.358000
C	-22.074000	7.954000	-2.534000
O	-23.248000	7.492000	-2.762000
N	-21.118000	7.286000	-1.877000
C	-21.311000	5.980000	-1.254000
C	-19.981000	5.184000	-1.299000
C	-20.028000	3.798000	-0.684000
C	-20.009000	3.624000	0.698000
C	-20.028000	2.360000	1.277000
C	-19.979000	1.242000	0.461000
C	-19.951000	1.394000	-0.919000
C	-19.982000	2.670000	-1.487000
C	-21.740000	6.202000	0.181000
O	-21.038000	6.883000	0.947000
N	-22.907000	5.671000	0.540000
C	-23.382000	5.733000	1.921000
C	-24.684000	6.517000	2.043000
C	-24.705000	7.933000	1.525000
C	-26.170000	8.375000	1.341000
C	-26.381000	9.220000	0.052000
N	-26.111000	10.705000	0.147000
C	-23.666000	4.326000	2.382000
O	-23.853000	3.440000	1.543000
N	-23.654000	4.123000	3.703000
C	-24.151000	2.879000	4.322000
C	-24.030000	2.920000	5.841000

O	-22.698000	2.663000	6.256000
C	-25.611000	2.916000	4.013000
O	-26.218000	3.995000	3.990000
N	-26.199000	1.760000	3.821000
C	-27.583000	1.684000	3.371000
C	-28.572000	2.906000	3.812000
C	-28.609000	3.091000	5.365000
O	-28.227000	4.159000	3.177000
C	-27.554000	1.341000	1.901000
O	-27.573000	0.153000	1.584000
N	-27.412000	2.322000	1.009000
C	-27.538000	2.014000	-0.426000
C	-28.019000	3.228000	-1.203000
C	-28.523000	4.349000	-0.318000
C	-28.572000	5.693000	-1.058000
O	-29.739000	6.118000	-1.330000
O	-27.476000	6.296000	-1.374000
C	-26.260000	1.504000	-1.063000
O	-26.297000	0.669000	-1.987000
N	-25.129000	2.039000	-0.610000
C	-23.845000	1.558000	-1.096000
C	-23.628000	0.160000	-0.534000
C	-23.451000	0.125000	0.973000
C	-23.755000	-1.256000	1.553000
O	-24.518000	-1.385000	2.506000
N	-23.214000	-2.280000	0.950000
C	-23.683000	1.566000	-2.627000
O	-23.386000	0.534000	-3.235000
N	-23.896000	2.720000	-3.250000
C	-23.776000	2.873000	-4.710000
C	-24.542000	4.116000	-5.202000
C	-26.062000	4.094000	-4.875000
C	-26.764000	5.305000	-5.471000
C	-28.271000	5.388000	-5.020000
N	-28.751000	6.741000	-5.422000
C	-22.299000	3.036000	-5.079000
O	-21.549000	3.717000	-4.340000
N	-21.921000	2.438000	-6.222000
C	-20.531000	2.490000	-6.750000
C	-19.700000	1.260000	-6.373000
C	-20.163000	-0.086000	-6.941000
C	-21.193000	-0.817000	-6.343000
C	-21.616000	-2.068000	-6.878000
C	-21.001000	-2.582000	-7.984000
O	-21.355000	-3.828000	-8.508000
C	-19.935000	-1.887000	-8.575000
C	-19.534000	-0.651000	-8.044000
C	-20.602000	2.668000	-8.247000
O	-21.654000	2.429000	-8.859000
N	-19.477000	3.055000	-8.829000
C	-19.407000	3.327000	-10.247000
C	-18.997000	4.813000	-10.461000
C	-18.718000	5.281000	-11.897000
C	-20.012000	5.418000	-12.651000
C	-18.006000	6.620000	-12.009000
C	-18.420000	2.367000	-10.949000
O	-17.269000	2.178000	-10.470000
N	-18.849000	1.773000	-12.074000

C	-18.003000	0.863000	-12.808000
C	-18.783000	-0.357000	-13.407000
C	-19.611000	-1.059000	-12.292000
O	-19.669000	0.081000	-14.448000
C	-17.209000	1.639000	-13.864000
O	-17.717000	2.603000	-14.424000
N	-15.940000	1.258000	-14.059000
C	-15.019000	1.939000	-14.995000
C	-13.744000	2.403000	-14.280000
C	-14.009000	3.371000	-13.122000
C	-12.719000	3.720000	-12.382000
C	-14.699000	4.584000	-13.624000
C	-14.672000	0.872000	-16.017000
O	-14.128000	-0.180000	-15.666000
N	-15.061000	1.119000	-17.266000
C	-14.848000	0.191000	-18.367000
C	-15.851000	-0.940000	-18.317000
C	-17.283000	-0.437000	-18.450000
O	-17.648000	0.070000	-19.500000
N	-18.108000	-0.579000	-17.384000
C	-14.940000	0.982000	-19.676000
O	-15.365000	2.130000	-19.649000
N	-14.510000	0.403000	-20.799000
C	-14.494000	1.170000	-22.063000
C	-13.538000	0.575000	-23.087000
C	-12.082000	0.692000	-22.609000
O	-13.882000	-0.800000	-23.304000
C	-15.868000	1.348000	-22.738000
O	-16.061000	2.279000	-23.547000
N	-16.823000	0.472000	-22.459000
C	-18.188000	0.822000	-22.866000
C	-19.059000	-0.401000	-23.016000
C	-18.286000	-1.619000	-23.442000
C	-18.319000	-2.687000	-22.365000
O	-18.323000	-2.308000	-21.164000
O	-18.372000	-3.898000	-22.720000
C	-18.789000	1.819000	-21.879000
O	-18.091000	2.736000	-21.419000
N	-20.056000	1.657000	-21.518000
C	-20.654000	2.664000	-20.636000
C	-22.135000	2.962000	-20.950000
O	-23.022000	2.006000	-20.339000
C	-20.503000	2.250000	-19.197000
O	-20.598000	1.058000	-18.851000
N	-20.261000	3.258000	-18.368000
C	-20.128000	3.059000	-16.965000
C	-19.168000	4.104000	-16.422000
C	-17.880000	4.139000	-17.339000
O	-19.808000	5.379000	-16.411000
C	-21.497000	3.072000	-16.252000
O	-22.369000	3.850000	-16.590000
N	-21.675000	2.212000	-15.257000
C	-22.940000	2.140000	-14.571000
C	-23.571000	0.764000	-14.774000
C	-23.131000	0.129000	-16.022000
C	-24.244000	-0.496000	-16.753000
N	-24.134000	-0.178000	-18.163000
C	-25.184000	0.041000	-18.932000

N	-26.395000	-0.036000	-18.396000
N	-25.025000	0.343000	-20.214000
C	-22.859000	2.404000	-13.087000
O	-21.809000	2.182000	-12.435000
N	-23.989000	2.867000	-12.555000
C	-24.187000	2.945000	-11.128000
C	-25.176000	4.055000	-10.743000
C	-25.400000	4.059000	-9.246000
C	-24.714000	5.438000	-11.205000
C	-23.245000	5.620000	-11.116000
C	-24.798000	1.595000	-10.695000
O	-25.876000	1.225000	-11.200000
N	-24.098000	0.877000	-9.788000
C	-24.596000	-0.343000	-9.140000
C	-23.664000	-1.492000	-9.422000
C	-23.138000	-1.453000	-10.802000
S	-24.288000	-2.303000	-11.845000
C	-23.659000	-3.970000	-11.610000
C	-24.614000	-0.160000	-7.644000
O	-24.174000	0.876000	-7.142000
N	-25.092000	-1.189000	-6.943000
C	-25.267000	-1.146000	-5.471000
C	-26.792000	-0.872000	-5.040000
C	-27.217000	0.551000	-5.428000
O	-27.661000	-1.813000	-5.722000
C	-24.787000	-2.455000	-4.827000
O	-24.919000	-3.527000	-5.423000
N	-24.164000	-2.348000	-3.657000
C	-23.843000	-3.503000	-2.820000
C	-25.136000	-4.145000	-2.266000
C	-25.955000	-3.134000	-1.432000
C	-27.134000	-3.745000	-0.691000
C	-28.085000	-2.653000	-0.243000
N	-29.367000	-3.231000	0.350000
C	-22.943000	-4.518000	-3.480000
O	-23.252000	-5.720000	-3.566000
N	-21.798000	-4.044000	-3.954000
C	-20.772000	-4.955000	-4.484000
C	-19.485000	-4.169000	-4.774000
C	-18.324000	-4.912000	-5.392000
C	-18.748000	-5.384000	-6.743000
C	-17.173000	-3.898000	-5.578000
C	-20.450000	-6.148000	-3.553000
O	-20.232000	-5.965000	-2.381000
N	-20.411000	-7.366000	-4.101000
C	-20.050000	-8.579000	-3.348000
C	-18.549000	-8.642000	-3.052000
C	-17.680000	-8.645000	-4.283000
C	-17.471000	-10.057000	-4.877000
N	-16.259000	-10.059000	-5.695000
C	-16.172000	-9.421000	-6.868000
N	-15.014000	-9.385000	-7.574000
N	-17.249000	-8.797000	-7.336000
C	-20.818000	-8.752000	-2.064000
O	-20.274000	-9.239000	-1.058000
N	-22.092000	-8.379000	-2.076000
C	-22.833000	-8.358000	-0.815000
C	-24.351000	-7.948000	-1.041000

C	-22.724000	-9.691000	-0.088000
O	-22.330000	-9.723000	1.080000
N	-23.047000	-10.802000	-0.762000
C	-23.084000	-12.121000	-0.052000
C	-23.566000	-13.242000	-0.983000
C	-25.064000	-13.541000	-0.987000
C	-25.327000	-15.016000	-1.314000
O	-26.129000	-15.676000	-0.656000
N	-24.617000	-15.541000	-2.312000
C	-21.691000	-12.507000	0.445000
O	-21.518000	-13.032000	1.529000
N	-20.688000	-12.239000	-0.386000
C	-19.315000	-12.637000	-0.100000
C	-18.434000	-12.414000	-1.338000
C	-18.806000	-13.346000	-2.496000
C	-19.783000	-12.715000	-3.486000
O	-19.633000	-12.883000	-4.665000
N	-20.759000	-11.970000	-3.003000
C	-18.768000	-11.918000	1.088000
O	-18.201000	-12.531000	1.976000
N	-18.917000	-10.599000	1.134000
C	-18.375000	-9.843000	2.258000
C	-18.380000	-8.371000	1.908000
S	-17.338000	-8.032000	0.471000
C	-19.064000	-10.099000	3.608000
O	-18.427000	-9.996000	4.677000
N	-20.363000	-10.438000	3.575000
C	-21.043000	-10.888000	4.795000
C	-22.514000	-11.267000	4.526000
C	-23.440000	-10.000000	4.494000
C	-25.002000	-10.291000	4.379000
N	-25.590000	-9.745000	3.147000
C	-26.033000	-10.496000	2.144000
N	-26.529000	-9.934000	1.060000
N	-25.981000	-11.822000	2.224000
C	-20.262000	-12.043000	5.385000
O	-20.044000	-12.101000	6.567000
N	-19.812000	-12.955000	4.543000
C	-18.958000	-14.048000	5.003000
C	-18.714000	-15.049000	3.858000
C	-17.768000	-16.158000	4.231000
C	-18.216000	-17.247000	5.024000
C	-17.344000	-18.248000	5.376000
C	-15.985000	-18.220000	4.927000
C	-15.541000	-17.171000	4.153000
C	-16.439000	-16.143000	3.789000
C	-17.613000	-13.614000	5.597000
O	-17.253000	-14.058000	6.671000
N	-16.860000	-12.770000	4.885000
C	-15.510000	-12.415000	5.320000
C	-14.684000	-11.784000	4.151000
C	-14.376000	-12.816000	3.096000
C	-14.933000	-12.923000	1.834000
N	-14.438000	-14.033000	1.178000
C	-13.545000	-14.670000	2.022000
C	-12.784000	-15.842000	1.819000
C	-11.981000	-16.272000	2.848000
C	-11.906000	-15.565000	4.076000

C	-12.641000	-14.384000	4.277000
C	-13.476000	-13.921000	3.230000
C	-15.586000	-11.499000	6.498000
O	-14.776000	-11.556000	7.419000
N	-16.596000	-10.637000	6.465000
C	-16.682000	-9.565000	7.434000
C	-17.232000	-8.301000	6.756000
C	-17.072000	-7.067000	7.634000
O	-16.481000	-8.091000	5.550000
C	-17.408000	-9.966000	8.717000
O	-16.950000	-9.657000	9.800000
N	-18.503000	-10.704000	8.643000
C	-19.160000	-11.025000	9.902000
C	-20.677000	-11.053000	9.769000
O	-21.121000	-9.797000	9.284000
C	-18.681000	-12.321000	10.484000
O	-18.243000	-12.335000	11.630000
N	-18.766000	-13.402000	9.712000
C	-18.389000	-14.726000	10.200000
C	-19.036000	-15.813000	9.371000
C	-18.503000	-17.146000	9.710000
C	-18.488000	-17.558000	11.043000
C	-17.980000	-18.775000	11.420000
C	-17.439000	-19.588000	10.468000
C	-17.422000	-19.186000	9.125000
C	-17.952000	-17.959000	8.759000
C	-16.875000	-15.100000	10.334000
O	-16.410000	-15.456000	11.434000
N	-16.129000	-15.071000	9.221000
C	-14.728000	-15.536000	9.200000
C	-14.058000	-15.281000	7.832000
C	-12.772000	-16.069000	7.626000
C	-12.810000	-17.405000	7.256000
C	-11.647000	-18.140000	7.093000
C	-10.433000	-17.546000	7.299000
C	-10.372000	-16.220000	7.684000
C	-11.530000	-15.484000	7.856000
C	-13.814000	-15.042000	10.346000
O	-13.015000	-15.817000	10.866000
N	-13.901000	-13.754000	10.728000
C	-14.825000	-12.711000	10.259000
C	-14.159000	-11.444000	10.718000
C	-13.321000	-11.772000	11.916000
C	-12.966000	-13.259000	11.775000
C	-13.179000	-13.951000	13.116000
O	-12.418000	-13.726000	14.064000
N	-14.220000	-14.775000	13.185000
C	-14.525000	-15.531000	14.380000
C	-16.014000	-15.878000	14.403000
C	-16.865000	-14.703000	14.800000
C	-18.319000	-14.996000	14.590000
C	-19.139000	-13.758000	14.814000
N	-20.561000	-13.976000	14.431000
C	-13.670000	-16.793000	14.489000
O	-13.164000	-17.105000	15.573000
N	-13.510000	-17.498000	13.363000
C	-12.775000	-18.770000	13.294000
C	-12.869000	-19.418000	11.868000

C	-14.256000	-19.310000	11.299000
C	-11.861000	-18.784000	10.918000
C	-11.278000	-18.685000	13.717000
O	-10.661000	-17.627000	13.899000
O	-10.602000	-19.703000	13.892000

5. Table S2, RMSD, RMSF, and Rg for protein-ligand complexes from MD simulations

Table S2. Root-mean-square displacement (RMSD), RMS fluctuations of alpha carbons of the protein backbone, and radius of gyration for complexes of BChE and ligands listed in the table, derived by molecular dynamics simulation of 100 ns.

Ligand	RMSD/Å		RMSF/Å		Rg/Å	
	average	min, max	average	min, max	average	min, max
**1a**	2.16	0.78, 2.67	0.83	0.34, 3.29	23.19	22.81, 23.41
**1b**	2.07	0.78, 2.59	0.85	0.35, 4.50	23.11	22.79, 23.34
**7**	2.02	0.81, 2.44	0.82	0.32, 6.59	23.06	22.81, 23.28

The MD simulation trajectory and topology for complex BChE-**1b** is freely available at https://data.fulir.irb.hr/islandora/object/irb:566 and MD-trajectory_prmtop-BChE-1b – Google disk

6. Complex formation of biometals with bioactive carbamates

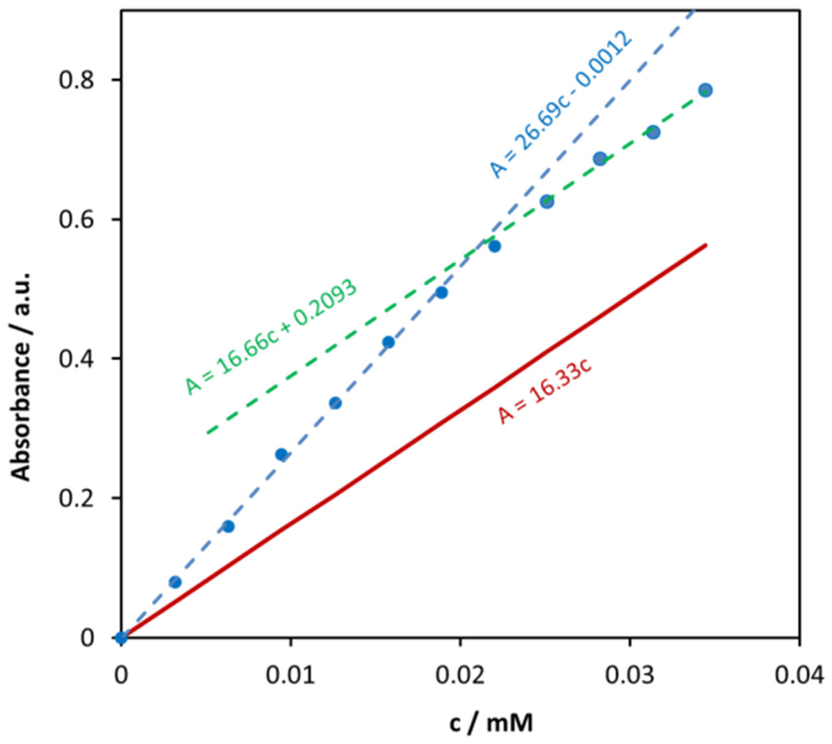

Figure S64. The enlarged version of the inset in Figure 9. It shows the changes of absorbance measured at 330 nm (blue) and the theoretical absorbance calculated for the ligand at the same wavelength (red) as a function of the concentration of **6**.

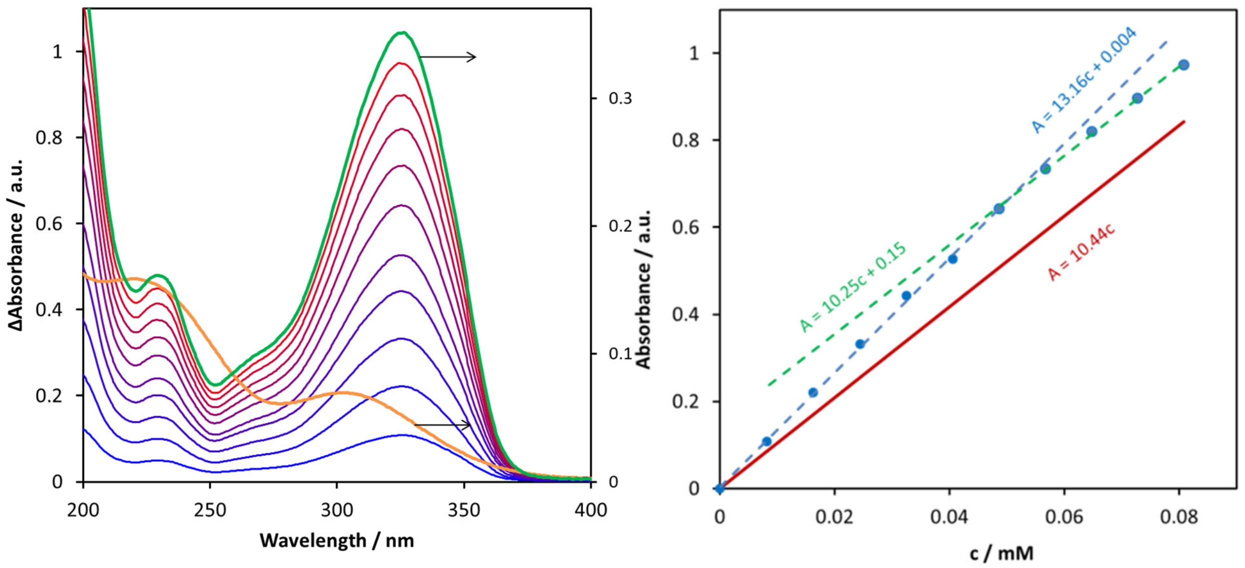

a) b)

Figure S65. a) Difference absorption spectra obtained by titration of 1.93×10^{-5} M of $Fe_2(SO_4)_3$ aqueous solution with compound **3** in the 0–0.35 mM concentration range. The orange curve is the spectrum of the starting pure $Fe_2(SO_4)_3$ solution, while the green spectrum belongs to the pure ligand. b) The changes of absorbance measured at 330 nm (blue) and the theoretical absorbance calculated for the ligand at the same wavelength (red) as a function of the concentration of **3**.

7. Dose – response curves for BChE inhibition by compounds 1–13

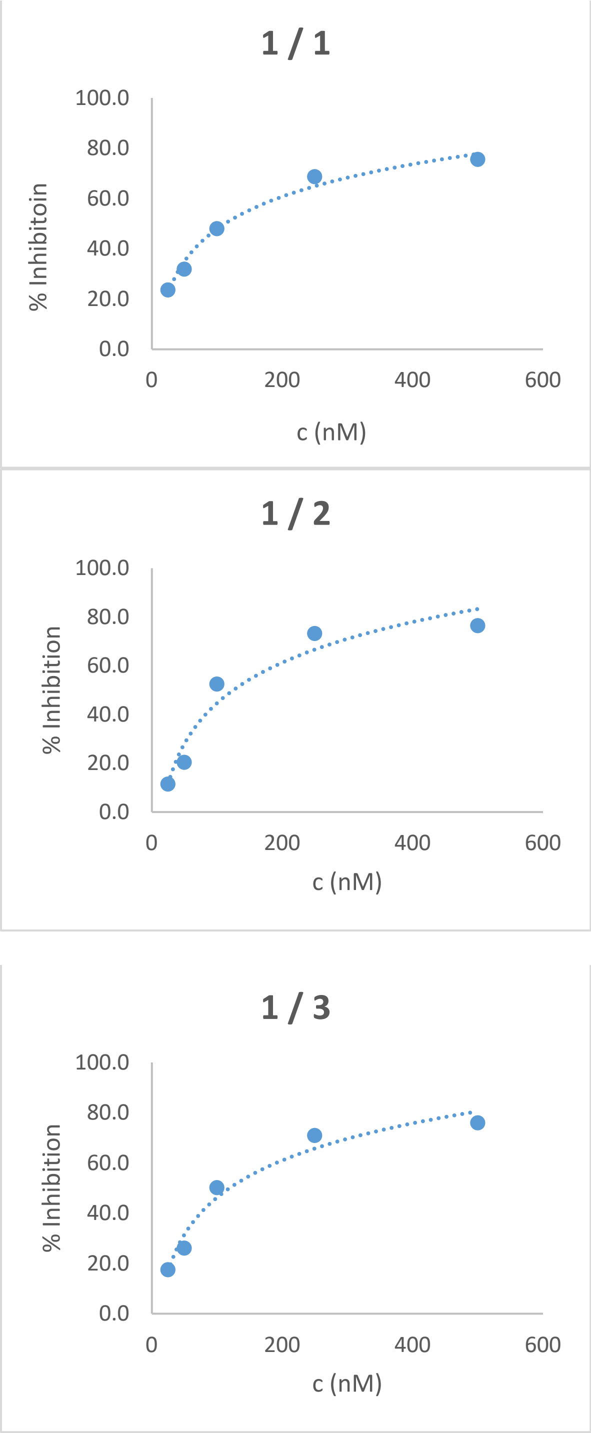

Figure S66. Dose – response curves for three measurements of BChE inhibition by compound **1**.

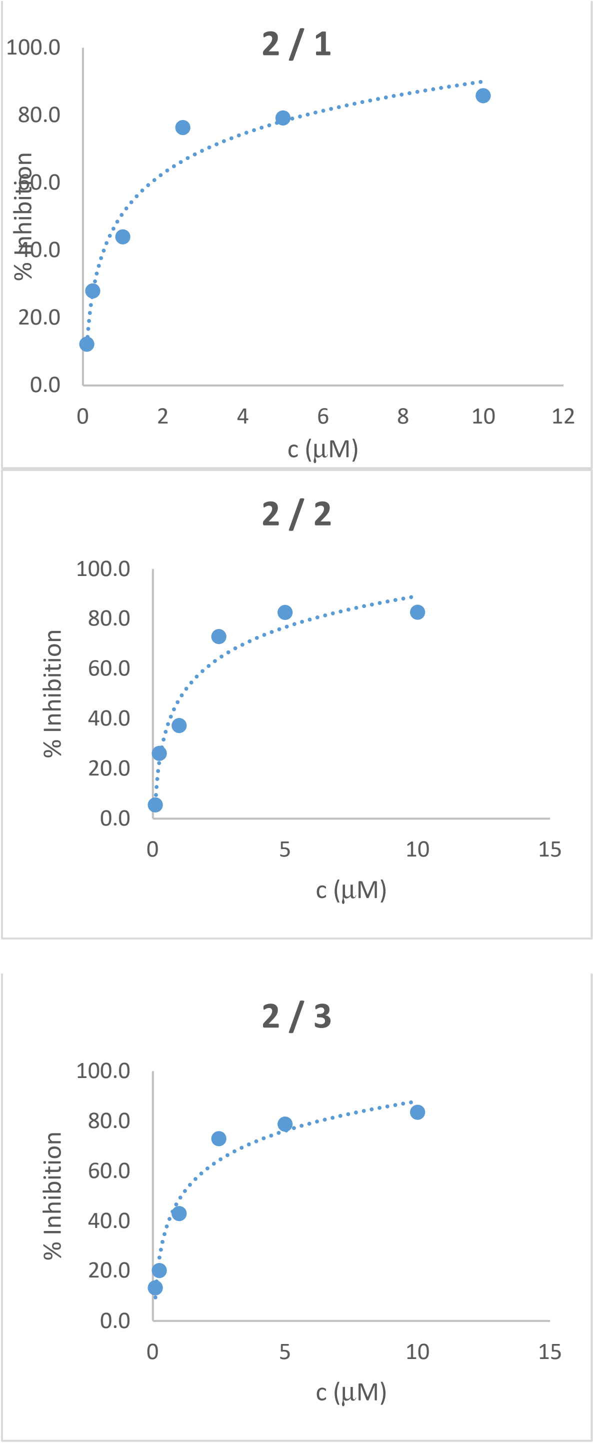

Figure S67. Dose – response curves for three measurements of BChE inhibition by compound **2**.

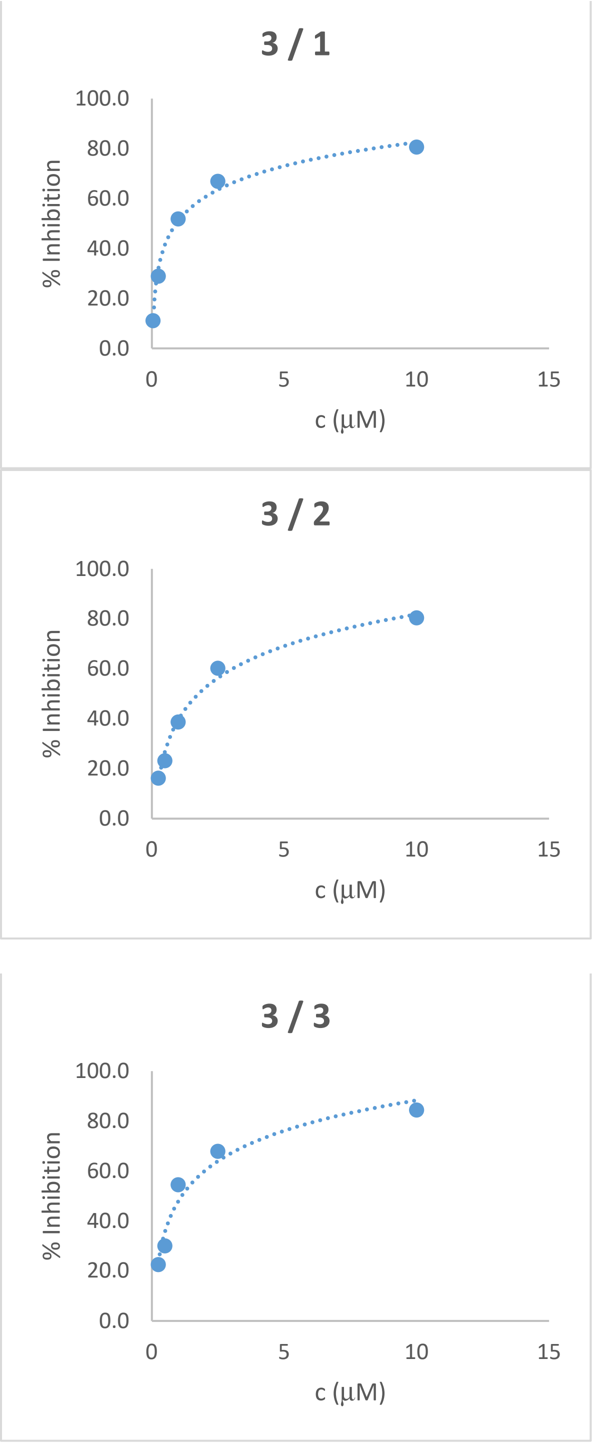

Figure S68. Dose – response curves for three measurements of BChE inhibition by compound **3**.

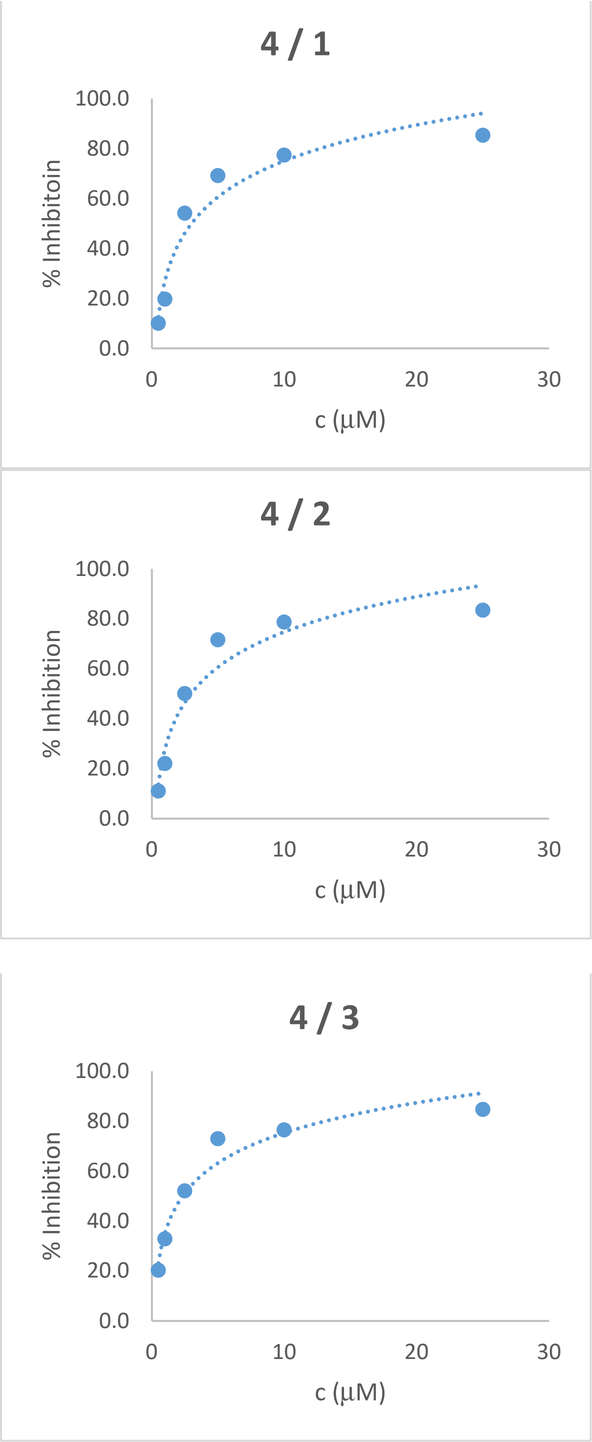

Figure S69. Dose – response curves for three measurements of BChE inhibition by compound **4**.

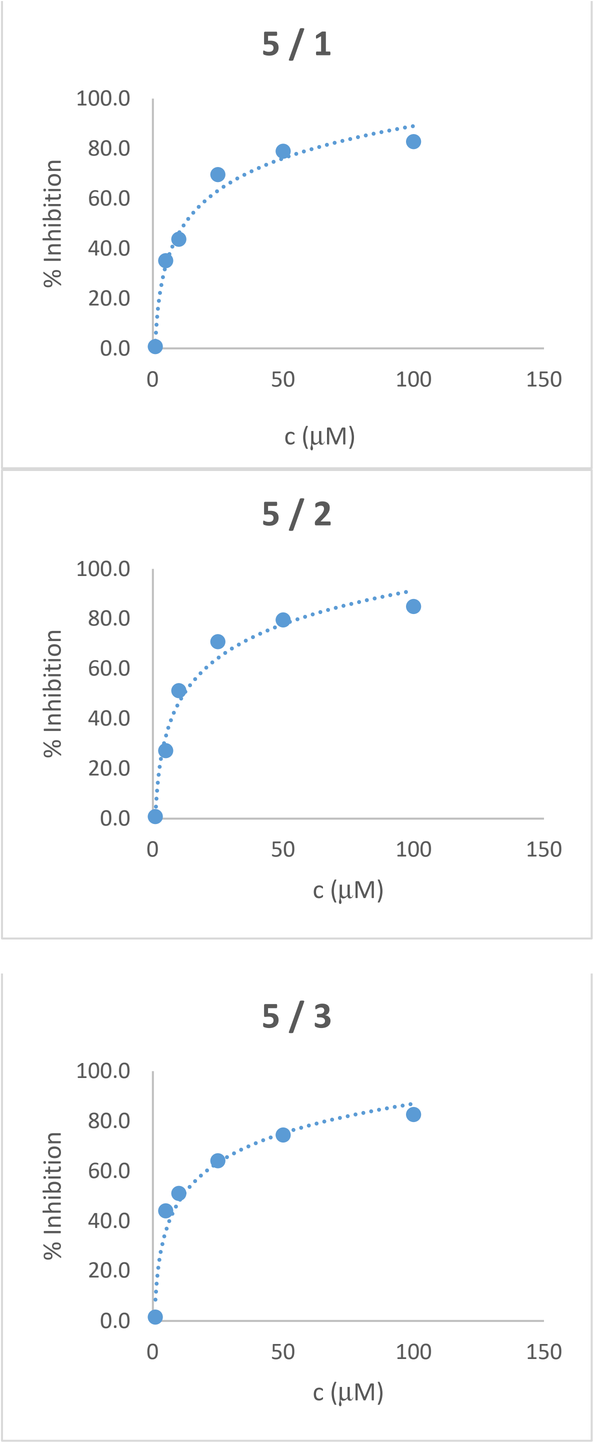

Figure S70. Dose – response curves for three measurements of BChE inhibiton by compound **5**.

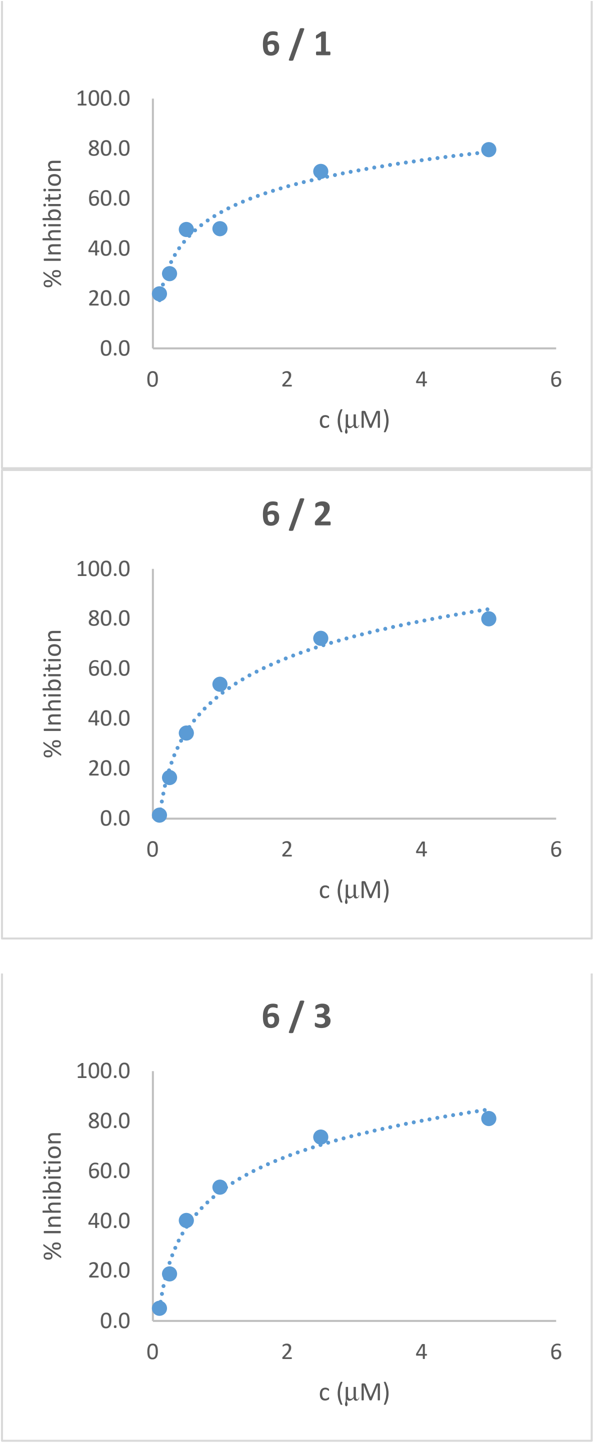

Figure S71. Dose – response curves for three measurements of BChE inhibiton by compound **6**.

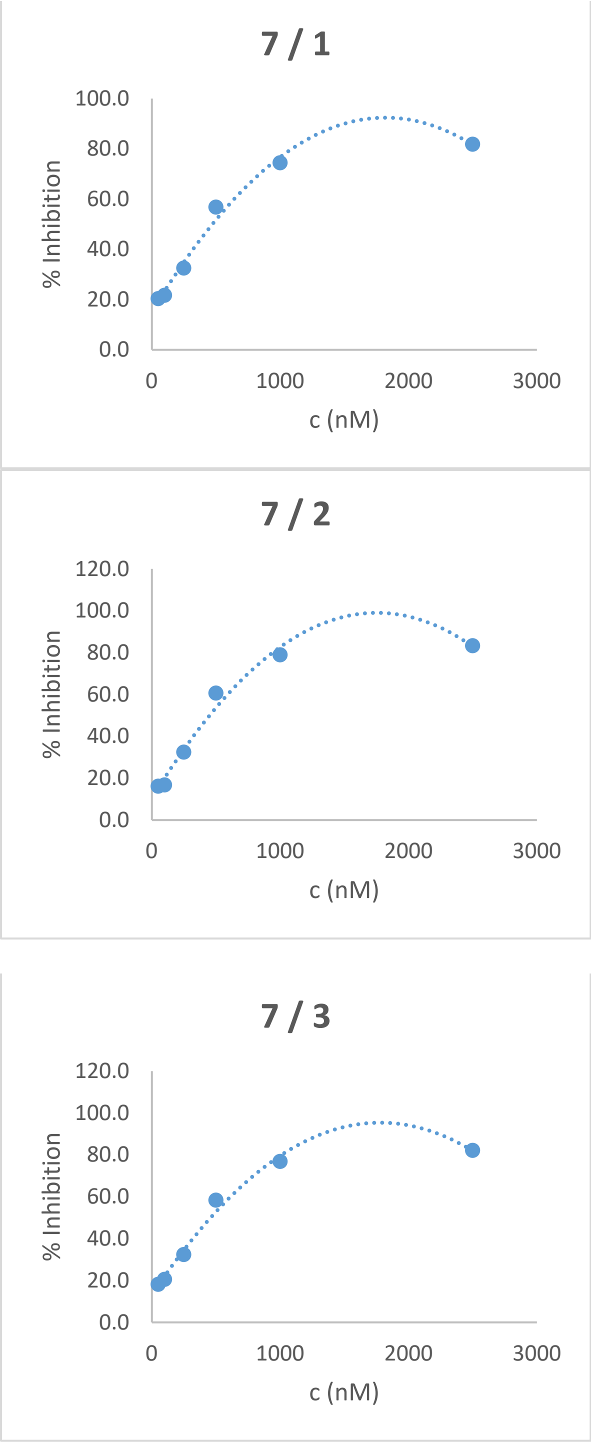

Figure S72. Dose – response curves for three measurements of BChE inhibiton by compound 7.

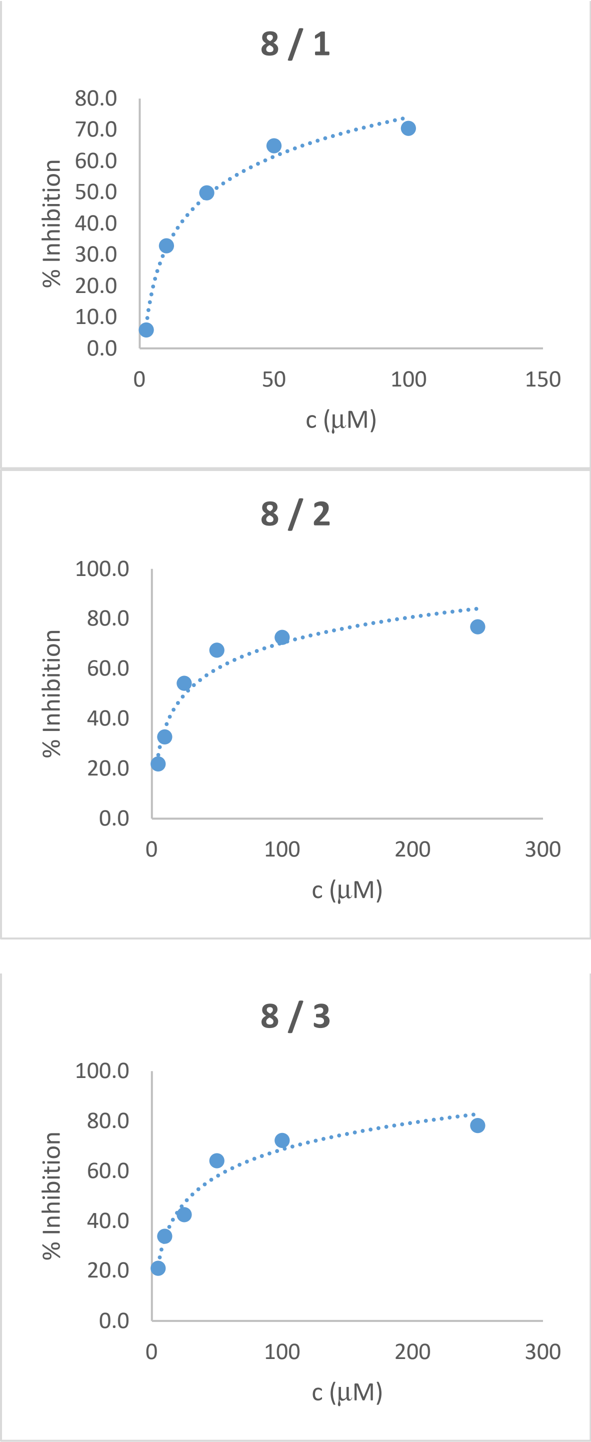

Figure S73. Dose – response curves for three measurements of BChE inhibiton by compound **8**.

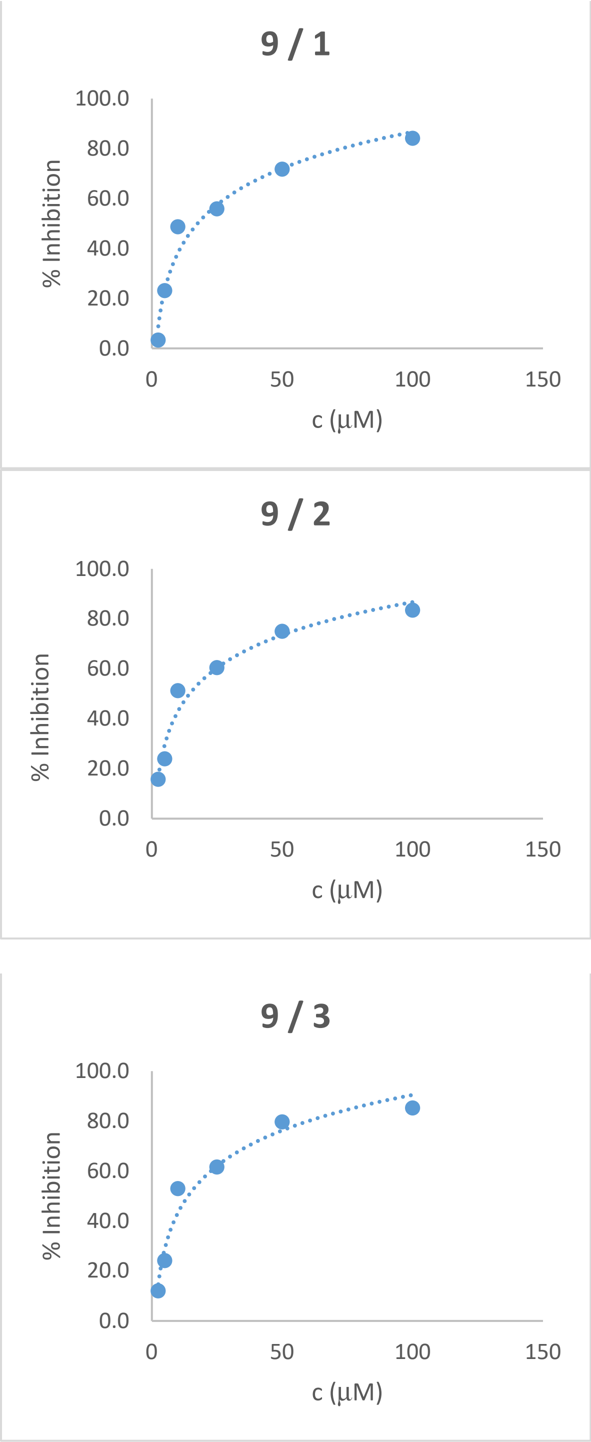

Figure S74. Dose – response curves for three measurements of BChE inhibiton by compound **9**.

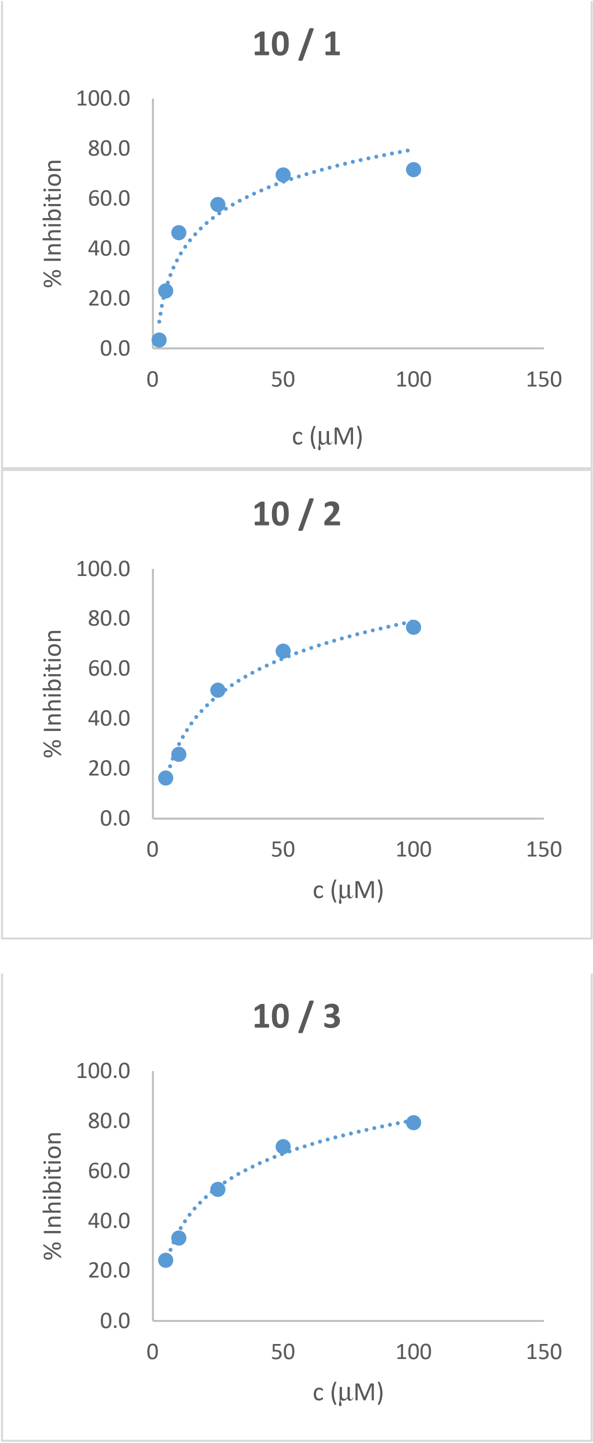

Figure S75. Dose – response curves for three measurements of BChE inhibiton by compound **10**.

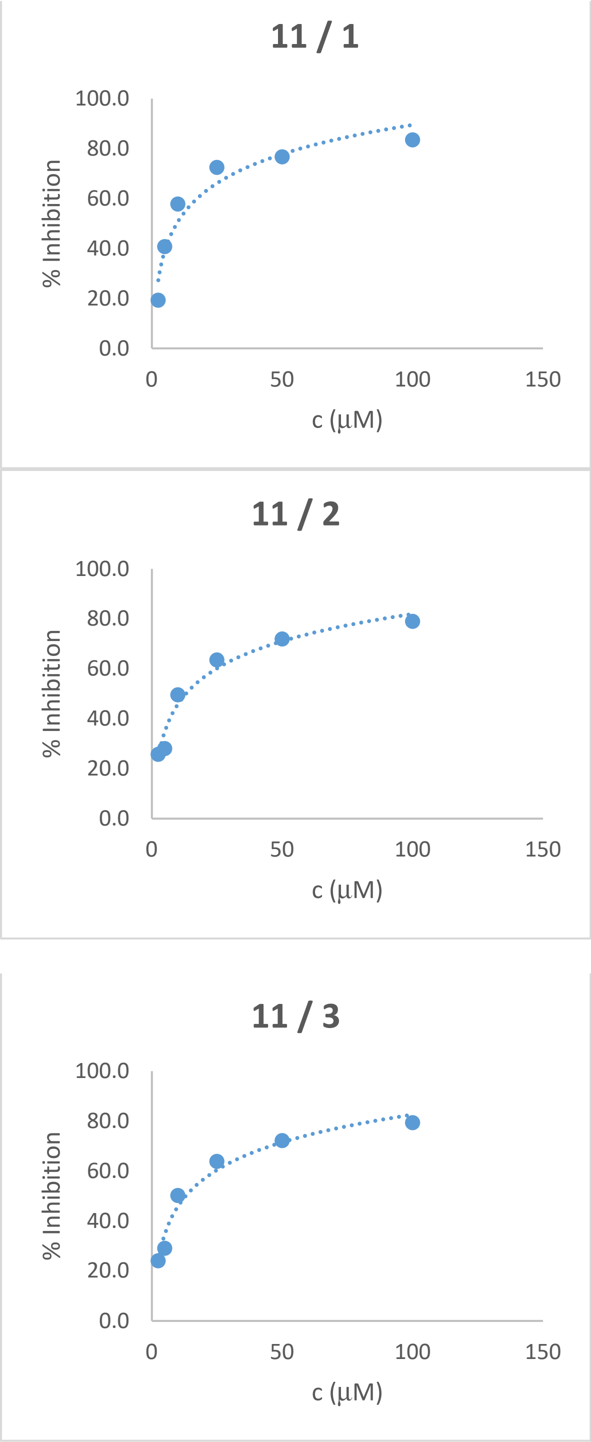

Figure S76. Dose – response curves for three measurements of BChE inhibiton by compound **11**.

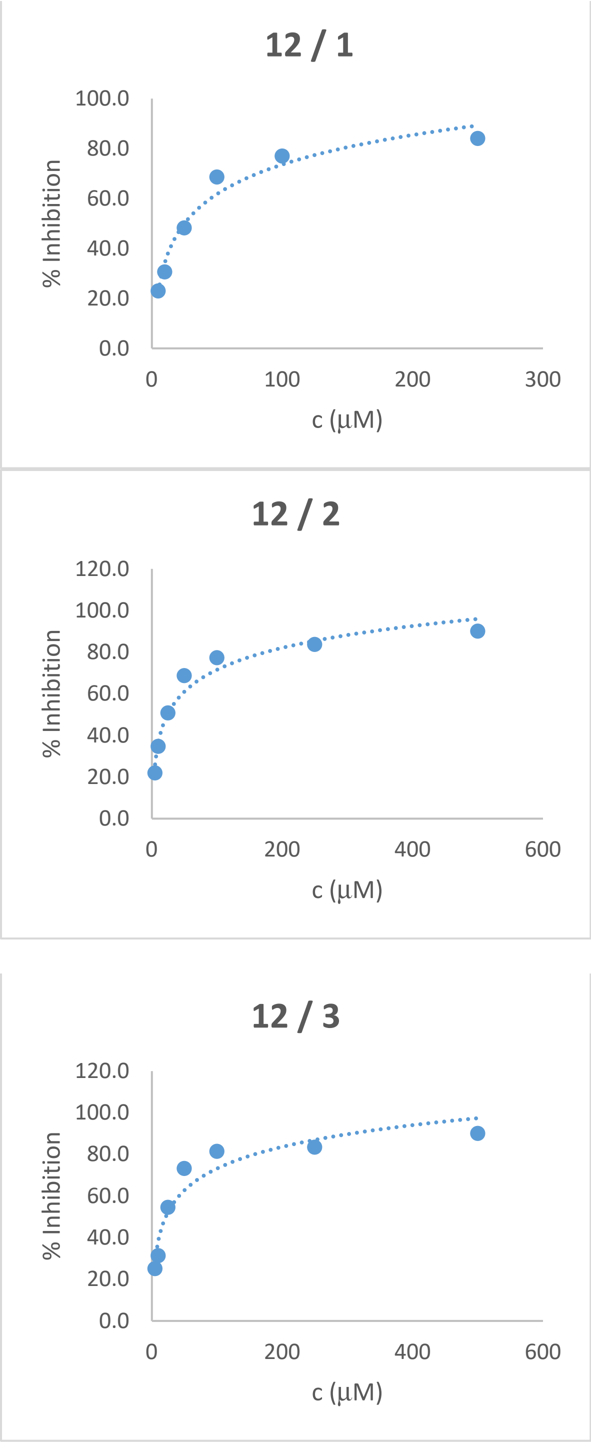

Figure S77. Dose – response curves for three measurements of BChE inhibiton by compound **12**.

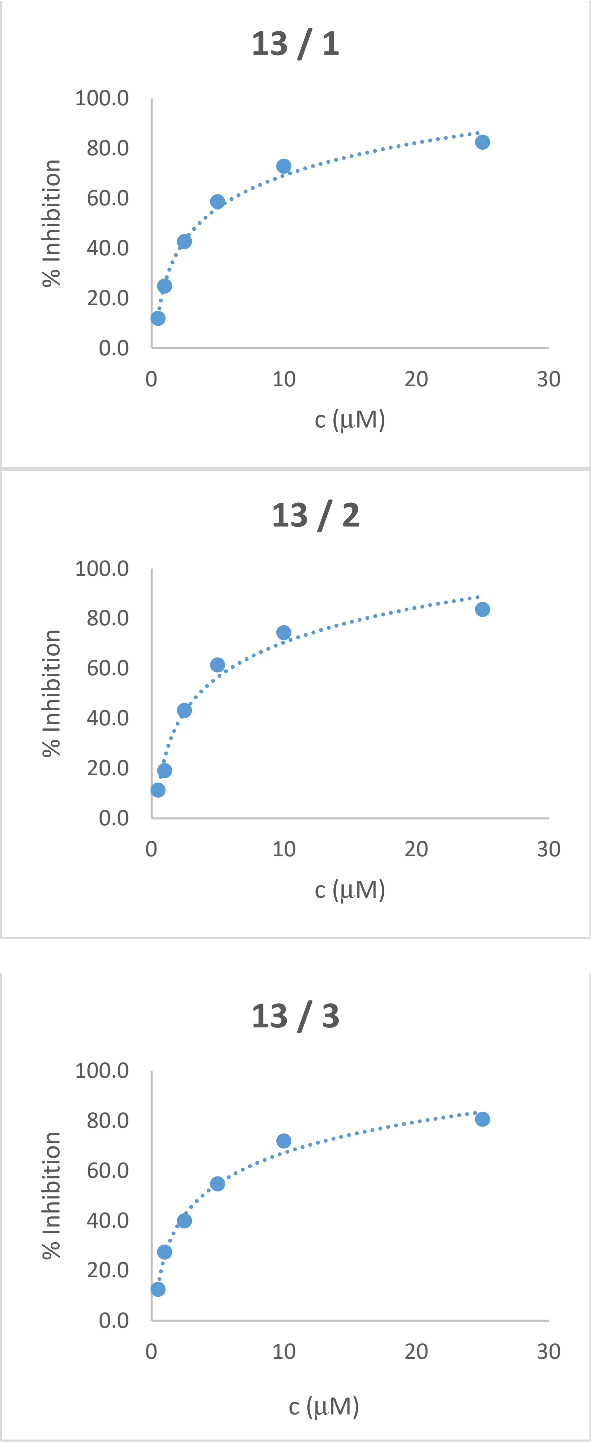

Figure S78. Dose – response curves for three measurements of BChE inhibiton by compound **13**.
